# Acute and chronic pesticide exposure trigger fundamentally different molecular responses in bumble bee brains

**DOI:** 10.1186/s12915-025-02169-z

**Published:** 2025-03-11

**Authors:** Alicja Witwicka, Federico López-Osorio, Andres Arce, Richard J. Gill, Yannick Wurm

**Affiliations:** 1 Biology Department, Mary University of London, London, Queen UK; 2 Department of Biology, Edge Hill University, Ormskirk, Lancashire UK; 3 Department of Life Sciences, Georgina Mace Centre for the Living Planet, Silwood Park Campus, Imperial College London, London, UK; 4 Digital Environment Research Institute, Queen Mary University of London, London, UK; 5 Alan Turing Institute, London, UK

**Keywords:** Neonicotinoids, *Bombus terrestris*, Insecticides, Transcriptomics, Ecotoxicology, Ttoxicogenomics, Safety assessments

## Abstract

**Background:**

Beneficial insects, including pollinators, encounter various pesticide exposure conditions, from brief high-concentration acute exposure to continuous low-level chronic exposure. To effectively assess the environmental risks of pesticides, it is critical to understand how different exposure schemes influence their effects. Unfortunately, this knowledge remains limited. To clarify whether different exposure schemes disrupt the physiology of pollinators in a similar manner, we exposed bumble bees to acute or chronic treatments of three different pesticides: acetamiprid, clothianidin, or sulfoxaflor. Genome-wide gene expression profiling enabled us to compare the effects of these treatments on the brain in a high-resolution manner.

**Results:**

There were two main findings: First, acute and chronic exposure schemes largely affected non-overlapping sets of genes. Second, different pesticides under the same exposure scheme showed more comparable effects than the same pesticide under different exposure schemes. Each exposure scheme induced a distinct gene expression profile. Acute exposure mainly caused upregulation of genes linked to the stress response mechanisms, like *peroxidase* and detoxification genes, while chronic exposure predominantly affected immunity and energy metabolism.

**Conclusions:**

Our findings show that the mode of exposure is critical in determining the molecular effects of pesticides. These results signal the need for safety testing practices to better consider mode-of-exposure dependent effects and suggest that transcriptomics can support such improvements.

**Supplementary Information:**

The online version contains supplementary material available at 10.1186/s12915-025-02169-z.

## Background

Safeguarding insect pollinators is critical for ecosystem stability, food production, and human welfare [41, 55, 58, 59]. Agricultural pesticides significantly contribute to pollinator declines [15, 27, 50], including through indirect sublethal effects [67]. These declines have prompted calls for a better understanding of how different exposure schemes affect physiology and behavior [24, 36, 37]. Indeed, exposure in natural environments ranges from low to high concentrations over variable periods [38], leading to distinct physiological and behavioral outcomes (e.g., [2, 62, 67]). For example, chronic long-term exposure to low doses of neonicotinoid pesticides impairs development, foraging, learning, and immunity [14, 68, 73]. By contrast, acute short-term exposure to higher neonicotinoid concentrations disrupts feeding and coordination while increasing locomotor activity [25, 77]. Additionally, locomotive behaviors of neonicotinoid-exposed bees can transition from hyperactive movement after acute exposure to hypoactivity when exposed for a more extended period [36, 37]. This variability in effect challenges the accuracy of risk assessments [20] and our ability to forecast population responses. Consequently, it is essential to understand how different exposure schemes impact organisms differently. So far, the extent to which different exposure schemes have distinctive molecular and physiological effects is unknown.

Focusing assessment of pesticide toxicity on a few selected phenotypes can result in contradictory measures of their negative effects [74]. In contrast, advances in transcriptomics enable us to measure the genome-wide effects of pesticide exposure [28, 42]. Indeed, simultaneously quantifying the expression levels of thousands of genes provides high-dimensional data that enables assessment of many aspects of insect physiology. Applying higher-resolution approaches could enable new insights into the impacts and risks of pesticide exposure. Several recent studies have provided insights into transcriptomic profiles induced by pesticide exposure, including genes related to macronutrient metabolism, immunity, learning, and memory [3, 4, 10, 46, 48, 74], showing that this technique can detect subtle, informative gene regulatory responses. Nevertheless, a critical knowledge gap remains regarding the molecular changes that underpin acute and chronic responses to different pesticides in pollinating insects.

**Fig. 1:**
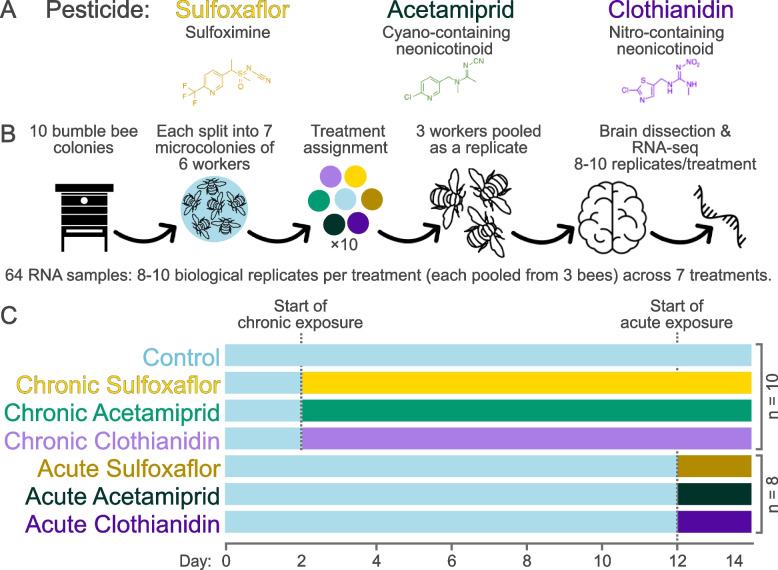
Experimental design.** A** Microcolonies were exposed to a control treatment or to one of three pesticides: sulfoxaflor, acetamiprid, and clothianidin. **B** Seven microcolonies of six callow workers were obtained from each of the ten bumble bee source colonies and assigned to one of the seven treatments; RNA extractions focused on pooled brains of three workers per microcolony. **C** After a 2-day adjustment period, we began chronic exposure; acute exposure began after 12 days to control for age of workers and day of sampling

Here, we aimed to test whether different pesticide exposure schemes disrupt physiology of a beneficial pollinator species in similar manners. For this, we exposed microcolonies of *Bombus terrestris* bumble bees to “acute”, i.e., short-term high-concentration and “chronic”, i.e., long-term low-concentration pesticide treatments, and measured activity levels of all genes. Across all treatments we used microcolonies from the same ten source colonies to increase power and control for genetic backgrounds (Additional File 1: Fig. S1). To investigate a range of outcomes, we performed experiments with three widely used pesticides: The neonicotinoid pesticides acetamiprid and clothianidin and the sulfoximine pesticide sulfoxaflor (Fig. 1A–C). Acetamiprid is a widely used neonicotinoid globally; clothianidin, albeit restricted in the European Union, is still used worldwide and exported from the EU in large quantities [17]; sulfoxaflor’s use has been increasing [8, 65] despite recent restrictions within the EU and reports of its high toxicity to bees [19]. We measured brain gene expression because each of these pesticides target nicotinic acetylcholine receptors (nAChRs) which are common in the brain [79], and because the brain governs behavioral responses. We hypothesized that exposure to different pesticides would lead to distinct gene expression responses, and that acute exposure to a pesticide would primarily affect the same genes as chronic exposure, but with a greater intensity.

## Results

### Acute exposure causes stronger and broader changes than chronic exposure

**Fig. 2:**
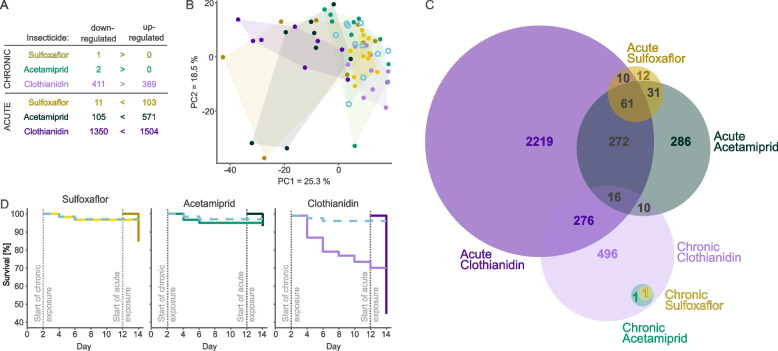
Gene expression changes and survival rates under chronic and acute exposure schemes. **A** Numbers of genes differentially expressed in each treatment compared to the control. **B** Principal component analysis of all microcolonies. The first principal component separates chronic and acute treatments; for the first two principal components, chronic treatments overlap with the control (blue). **C** Euler diagram showing relative numbers of differentially expressed genes and overlaps between treatments (DESeq2 Wald test cut-off of FDR 0.05). **D** Survival rates; Dashed blue lines indicate the control

We exposed bumble bee microcolonies to acute (21.5 ppb, 48 h) and chronic treatments (4.4 ppb, 12 days) of acetamiprid, clothianidin, and sulfoxaflor, and the control, and sequenced RNA from pools of brains of three workers per microcolony (Fig. 1A–C). Acute exposure was always more disruptive than chronic exposure, resulting in greater numbers of differentially expressed genes. For example, acute exposure to clothianidin resulted in 3.5 times more differentially expressed genes than chronic exposure (Fig. 2A). Similarly, changes in expression amplitude among the 20 genes with the most pronounced changes after acute exposure were 2.7 times greater than after chronic exposure (*t*-test, *p* < 10^−6^). In line with these patterns, principal component analyses showed that the expression profiles of chronically exposed microcolonies were more similar to control colonies than to acutely exposed colonies (Fig. 2B).

The effects of chronic exposure were also more consistent across the microcolonies than the effects of acute exposure (20% lower standard deviation; GLMM *p* < 10^−16^). The difference in variation may be because the organism’s physiology has more time to adjust over the 12 days of chronic exposure. Alternatively, genetic variation between colonies may affect the thresholds that trigger a response, as well as the rate at which the pesticide is taken up by the hemolymph and reaches the brain (Additional File 1: Fig. S2 and S3).

### Different exposure schemes drive different types of changes

There were major differences in the magnitude of effects between pesticides: Acute exposure to clothianidin and acetamiprid respectively changed the expression of 25 and 6 times more genes than acute exposure to sulfoxaflor. Chronic exposure to clothianidin similarly caused substantially more changes than acetamiprid or sulfoxaflor (Fig. 2A). Nevertheless, we observed discrepancies in the molecular responses between acute and chronic treatments. Expression levels of a set of 61 genes changed consistently in response to all acute treatments (40 times more than expected by chance, SE = 0.015; hypergeometric test, *p* < 10^−5^, Fig. 2C). Moreover, 89% of genes differentially expressed under acute exposure to sulfoxaflor were differentially expressed in at least one other acute treatment. However, none of these overlapping genes were differentially expressed in chronic exposure treatments.

**Fig. 3:**
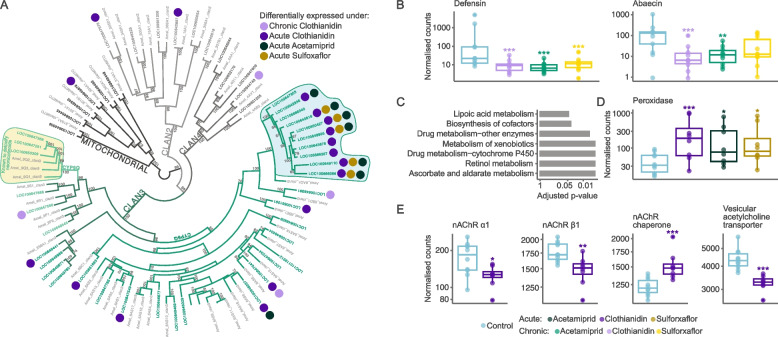
Selected genes affected by chronic and acute exposure schemes.** A** Maximum-likelihood tree of all cytochromes P450 in bumble bee (51 genes) and honey bee (49 genes). Filled circles highlight differentially expressed genes. Yellow area highlights CYP9Q genes known to detoxify neonicotinoids [29],none of these were upregulated. Blue area highlights genes consistently differentially expressed under acute exposure. Numbers indicate bootstrap values of nodes. **B** Antimicrobial peptide genes were downregulated under chronic treatments. **C** KEGG pathways enriched in genes differentially expressed under all acute treatments. **D** *Peroxidase* was upregulated in all acute treatments. **E** After acute clothianidin exposure, two nAChR-subunits whose *Drosophila* orthologs have particularly high affinity to clothianidin are downregulated, as is the vesicular acetylcholine transporter, while a chaperone which assists nAChR folding is upregulated. Boxplot significance codes: * < 0.05; ** < 0.01; *** < 0.001

Although chronic exposure affected considerably fewer genes than acute exposure, the effects of different pesticides also overlapped (Fig. 2A). In particular, expression of the antimicrobial peptide *defensin* was on average 32-fold lower in chronic treatments, while expression of another antimicrobial peptide, *abaecin*, was 11-fold lower after exposure to clothianidin and acetamiprid (Fig. 3B). The other genes most significantly affected by chronic clothianidin exposure included the pathogen response genes *kynurenine/alpha-aminoadipate aminotransferase* and *antichymotrypsin-2*, which were respectively downregulated tenfold and threefold relative to the controls. Changes in expression levels of immune genes may explain the decreased immunocompetence of bees exposed to neonicotinoids [56]. Intriguingly, expression of these immune genes was unaffected by acute exposure.

### Acute exposure to every pesticide caused upregulation of detoxification and stress-response genes

Among the 61 genes differentially expressed by all acute treatments, 91% were upregulated rather than downregulated (*X*^*2*^ = 23.8, *p* < 10^−6^). These included well-known detoxification genes (e.g., P450 cytochromes, ATP-binding cassette transporters, and UDP-glycosyltransferases). Surprisingly, none of the cytochrome P450 genes from the CYP9Q subfamily were upregulated. Genes in this subfamily are conserved across bee species and can determine neonicotinoid sensitivity [29, 45, 72]. The three bumble bee CYP9Q genes most able to metabolize neonicotinoids have high baseline expression in the brain (representing 33% of the total expression of 51 cytochrome P450s,*X*^*2*^ = 21.5, *p* < 10^−6^). The lack of upregulation in these genes, combined with their generally high baseline activity, suggests that detoxification mechanisms are constantly operational rather than being activated by exposure to specific compounds.

In contrast, expression of a subset of cytochrome P450 genes in the CYP6Q subfamily increased by an average of 248-fold in acute treatments. This subset of genes likely represents a recent expansion in the bumble bee (*Bombus* spp.) lineage because a phylogenetic reconstruction indicates a lack of orthologs in the honey bee and relatively short branch lengths (Fig. 3A, Additional File 1: Table S1). The dramatic upregulation of these genes may help metabolize the pesticides or their metabolites, or it may contribute to a generic stress response. Indeed, other stress-response components included upregulation of ascorbic and lipoic acid metabolism and *peroxidase* (Fig. 3C, D) likely indicating responses to oxidative stress which results from calcium ion influx after nAChR overstimulation [30, 75]. Furthermore, acute exposure to acetamiprid and clothianidin caused upregulation of peptide and fatty acid metabolism. This upregulation may help compensate for the detrimental impacts of cellular stress, as indicated by analogous changes in mice [80].

### Acute and chronic clothianidin exposure schemes trigger mortality through different mechanisms

Although we primarily compared the effects of insecticides on bumble bee brains, we also measured survival, enabling us to examine the links between gene expression and mortality. Acute and chronic exposure to the most toxic compound, clothianidin, led to the death of 54% and 29% of bees, respectively (Cox proportional hazard models; both *p* < 10^−6^; Fig. 2D). The cumulative doses of clothianidin consumed under both exposure schemes were comparable. This indicates that the effects of clothianidin do not cause stronger effects over time and that bees are better able to tolerate long-term lower-level exposure than acute exposure.

Both acute and chronic clothianidin treatments affected a set of 292 overlapping genes, more than expected by chance (hypergeometric test, *p* < 10^−6^). However, most of the affected genes (90% and 64%, respectively) were treatment specific. Intriguingly, acute exposure upregulated apoptosis-inducing genes, suggesting immediate lethal effects, while such links were absent after chronic exposure. Acute exposure to clothianidin also caused changes in the expression of genes important for nerve function, including upregulation of synaptic vesicle proteins involved in neurotransmitter transport and release, and downregulation of neuroligins which are crucial for synapse stability. Acute clothianidin exposure also downregulated nAChR subunits α1 and β1, and the vesicular acetylcholine transporter, and upregulated the acetylcholine receptor chaperone which supports assembly of nAChRs (Fig. 3E). Different changes stood out after chronic exposure to clothianidin, including downregulation of genes associated with the respiratory transport chain and ATP synthase complex (Fisher’s exact test, all *p*-values < 0.005).

## Discussion

Contrary to our initial hypothesis, the pesticide exposure scheme was a stronger determinant of bumble bee brain gene expression profiles than the type of pesticide. Indeed, we found more overlap among affected genes after acute exposure to different pesticides than between acute and chronic exposure to individual pesticides. This indicates that despite differences between pesticides in baseline toxicity and in chemical structures, the effects of exposure are strongly determined by exposure duration and intensity. Moreover, the pesticides tested here do not show time-reinforced toxicity; the strength of effects does not increase over time. Instead, the effects differ altogether. Acute treatments cause severe and immediate stress responses, while chronic exposure gradually impairs immunity and energy metabolism. Thus, different exposure schemes pose distinct challenges for pollinators.

Our results indicate potential links between molecular alterations and previously observed phenotypical changes. For instance, chronic clothianidin exposure downregulated genes related to the respiratory transport chain and ATP production, while acute exposure disrupted nerve function and neurotransmitter genes. These alterations parallel observed changes in locomotor behaviors: acute exposure increases locomotor activity, potentially due to synaptic overstimulation, while chronic exposure reduces locomotor activity, likely due to changes in energy metabolism [36, 71]. Acute exposure to clothianidin caused downregulation on α1 and β1 nAChR subunit. Remarkably, orthologs of these subunits in the fruit fly have particularly high affinity to clothianidin [44]. These changes in gene expression suggest that some nAChRs in the brain may be reassembled after acute exposure, potentially to prevent further toxic effects. Moreover, downregulation of key immunity genes under chronic exposure to all three pesticides may explain the lower immunocompetence of bees exposed to neonicotinoids [56].

In agricultural settings, pollinators may survive pesticide exposure but suffer from sub-lethal disruptions that jeopardize their long-term abilities to reproduce and to pollinate crops and wild plants. Estimating the risks of such diverse detrimental impacts is essential for ensuring the environmental safety of pesticides. Traditionally, the acute LD_50_ dose which kills 50% of individuals over 24 or 48 h has often been used as basis for calculating the risk posed by pesticides in a variety of exposure scenarios (OECD Test no. 213, [53]; OECD Test no. 214, [52]). Subsequent developments have included chronic 10-day pesticide exposure assays that explicitly record behavioral abnormalities (OECD Test no. 245, [54]). These behavioral abnormalities are used as a proxy for sublethal effects and have recently been integrated into EU regulatory guidance [20]. While these improvements toward including sub-lethal effects are commendable, the new protocols are still unlikely to capture the full spectrum of changes detected here by gene expression profiling.

By comparing two neonicotinoids and a sulfoximine, we reveal gene expression disruptions that likely represent general patterns relevant to nAChR-targeting insecticides. Such compounds account for 24% of the global insecticide market, the largest share among all chemical classes [70]. Although regulators have banned clothianidin and restricted sulfoxaflor throughout most of the native European range of *B. terrestris*, these compounds can persist and accumulate in soils for years [26]. For example, a recent study reported that the banned neonicotinoid imidacloprid ranked among the most common compounds found in colony pollen stores of bumble bees in Europe [50]. Moreover, clothianidin [18] is still commonly used worldwide [63]. EU countries also export large amounts of clothianidin globally. For example, in 2022, 656 tonnes of clothianidin were shipped to non-EU countries where its use is still permitted in some form (e.g., USA, Canada, China, Brazil, India, Indonesia, [17]). Furthermore, clothianidin is a breakdown product of thiamethoxam, which is still used under emergency derogations in Europe. A Greenpeace/Unearthed investigation found that 2 years after the 2018 ban, EU countries issued at least 67 different “emergency authorisations” for outdoor use of these chemicals. Sulfoxaflor’s use has been increasing [8, 65] despite recent restrictions within the EU and reports of its high toxicity to bees when applied during flowering [19, 33].

Despite general recommendations to not spray pesticides onto flowering plants that have been in place since 2013, honey bees still had high levels of these compounds in their hives [47], indicating their spread and persistence in the environment [32]. Neonicotinoids are also applied as a seed treatment. Therefore, although not applied during the flowering period, the systemic nature of the compound means it permeates plant tissues, including pollen and nectar, which are accessible to bees. The residues persist years after application [82], creating long-term exposure hazards for pollinators. For example, hibernating underground bumble bee queens prefer neonicotinoid-contaminated soils, which puts them at risk of exposure [60]. Moreover, bumble bees, including *B. terrestris*, are globally used as commercial pollinators in greenhouses [22], making them vulnerable to exposure to compounds that may be restricted within their geographic range.

Therefore, even with certain compounds facing restrictions across different geo-political regions, understanding their differential impacts remains essential, as exposure risks persist through international trade, environmental contamination, and the continued development of similar cholinergic pesticides. Furthermore, our results highlight how length of exposure can affect an organism in fundamentally different ways. For many pollinators, including worker bumble bees, 12 days of exposure constitutes a significant portion of their adult life. By examining how environmental stressors influence metabolic processes during this period, we can deepen our understanding of how stress affects individual pollinators and in turn how it may affect colony health and resilience overall. In the early days of examining gene expression to human diseases, only little information existed to help link changes in individual genes to tangible health outcomes [31]. Similarly, we cannot yet directly link exact changes in gene expression to the ability of pollinators to survive, reproduce, or pollinate. However, based on our understanding of cellular processes, changes in the expression levels of many genes undoubtedly indicate substantial disruption to basic biological functions.

## Conclusions

Our findings show how measuring expression levels of thousands of genes can reveal previously unknown impacts of pesticides on beneficial species. Our work also highlights the complexities of such effects. We show that different exposure schemes can cause different effects in the brain. However, it is important to note that effects of exposure also vary substantially across body parts [78]. Here, we focused on two neonicotinoids and a sulfoximine which all target nicotinic acetylcholine receptors (nAChRs). The use and diversity of nAChR-targeting pesticides continue to increase [26, 66], and we look forward to work clarifying how the patterns we observed here extend to pesticides with different modes of action, to other stressors, and to other beneficial insect species. Broader use of high-resolution approaches could significantly expand our understanding of how environmental stressors impact insect health. Just as gene expression profiling revolutionized the diagnosis and understanding of human disease, we anticipate it will play a pivotal role in the development and regulatory evaluation of novel pesticides.

## Materials and methods

### Pesticide solution preparation

Bumble bees were exposed to three pesticides that target nicotinic acetylcholine receptors (nAChRs): clothianidin, acetamiprid, or sulfoxaflor (Sigma Aldrich, UK). We used the same concentrations for each compound to allow for direct comparisons. We based the chronic concentration on the residues found in pollen and nectar [6, 12, 57, 61, 64]. We established the acute concentration based on the reports on clothianidin, the most toxic compound among these tested here to ensure survival of the bees and adequate replication levels for RNA sequencing. Reports suggest that 24 h-LD_50_ for clothianidin varies greatly between colonies of *A. mellifera ligustica*, ranging from 9.69 to 41.96 ppb [40], we decided on a dose in the middle of that range. We established the chronic concentration at 5 μg/L and the acute at five times higher, 25 μg/L. We prepared pesticide stock solutions by dissolving pesticides in acetone to a concentration of 2.5 and 0.5 mg/mL and stored in darkness at − 20℃. Subsequently, we diluted the stock solutions using 30% sucrose solution to 5 and 25 μg/L feeding solutions. Given that the weight of a liter of 30% sucrose solution is 1130 g, the final concentrations were 4.4 and 22.1 ppb. To avoid pesticide degradation, we stored feeding solutions in darkness at 4℃.

### Microcolony setup and sampling

We acquired ten source colonies of *Bombus terrestris audax* from a commercial breeder (Agralan Growers UK). We transferred the queen, existing brood and 20 workers to wooden boxes (30 cm long/20 cm wide/15 cm deep) separated into two equal-sized chambers (foraging and nesting area). We provided each colony with ad libitum 30% sucrose solution and organic honeybee-collected pollen (General Food Merchants LTD). We marked all twenty workers using water-based markers (POSCA, UK) and screened the colonies daily for the emergence of new workers. All source colonies were 2 weeks old when we started setting up the microcolonies. Six callow workers that emerged within 24 h were used to assemble microcolonies. Over 14 days, we obtained seven microcolonies from each source colony in a staggered manner, depending on the pace of worker production (Additional File 1: Fig. S4).

Each microcolony was kept in a single-chamber wooden box (12 cm long/12 cm wide/10 cm deep) and provided with organic pollen ad libitum and a single dose of nest substrate (5 parts organic pollen to 1 part 30% sucrose solution in a 2.5-cm Petri dish). Each microcolony received 10 mL of 30% control sucrose solution across two 5-mL syringes. We assigned the control treatment or one of the six pesticide treatments to all microcolonies in a randomized fashion. Our design ensured that treatments were assigned to microcolonies created at various stages of the source colony development. We replaced the control syringes with two syringes containing pesticide solution (a) 4.4 ppb after 2 days for chronic exposure or (b) 22.1 ppb after 12 days for short-term, acute exposure, so that all workers were of the same age at the time of sampling. We changed pollen and sugar solution every 48 h between 11:00 am and 1:00 pm. All colonies remained in darkness at 24℃, and we used red light during feeding and sampling. All sampling took place between 1:00 pm and 2:00 pm to minimize the effects of circadian rhythms on gene expression profiles. We placed individual workers in 2-mL screw-cap cryovial tubes, rapidly submerged them in liquid nitrogen, and stored the samples at − 80℃. We obtain ten replicates per treatment except for acute clothianidin were we obtained eight replicates because the mortality rates in two source colonies were continuously too high. Our experimental design builds on insights from previous similar studies [4, 10] in three key ways: (I) We used microcolonies to ensure that bees from different genetic backgrounds were exposed to each treatment; (II) We pooled RNA from three individuals (from the same microcolony and treatment) to reduce the impacts of between-individual variation in food intake, physiology, and behavior; (III) We used more than twice the number of biological replicates as in the previous study.

### Survival analysis

Because we had to collect enough living bees per microcolony to establish a sufficient sample size for RNA-seq, we did not measure the LD50 values for each treatment. However, we collected data on the survival of individual bees throughout the experiment during feeding. We fitted Cox proportional hazards regression models to conduct pairwise comparisons of survival between each treatment and the control utilizing *survfit* function from the survival v.3.5–7 R library. For chronic treatments, we compared the data from the entire experiment duration (14 days). For the acute treatments, we only compared survival data from the last 2 days of the experiments when the microcolonies assigned to acute treatments experienced exposure.

### RNA extraction, library preparation, and high-throughput sequencing

In the absence of a queen, one bumble bee worker can become dominant, which induces development of bigger ovaries. We dissected abdomens to examine ovarian development and excluded dominant workers from further steps as significant ovarian development may change gene expression patterns. We dissected brains on dry ice and immediately placed them in a homogenization tube. We randomly selected three non-dominant workers per microcolony and pooled brains for further steps. We homogenized the dissected brains in TRIzol using a FastPrep96 (45 s at 1800 RPM). We isolated RNA using chloroform and purified it with Genaxxon RNA Mini Spin Kit, applying DNase I on-column digestion. We prepared cDNA libraries using the NEBNext® Ultra™ II Directional RNA Library Prep Kit for Illumina. We altered the standard protocol using one-third volumes of enzymes and buffers and 300 ng of total RNA input with 14 PCR cycles. Library size was quantified using TapeStation 2200 (Agilent, UK) and Qubit 2.0 fluorometer. Libraries were sequenced at QMUL Genome Centre on NextSeq 500 generating a mean of 36 million 40 bp paired-end reads per sample (from 22.8 million to 52.2 million reads).

### Quality assessment of raw reads

Initially, we assessed the quality of raw reads using FastQC v0.11.9 [1]. To evaluate alignment qualities, we respectively aligned RNA-seq samples to the *B. terrestris* genome and transcriptome (Ensembl Metazoa release 52) using STAR v2.7 [16] and kallisto v0.46 [7]. We processed STAR alignments using the RNA-seq module of Qualimap v2.2.1 [51] and summarized the results from FastQC and Qualimap using MultiQC v1.9 [21]. Four samples failed our quality-control checks due to poor alignment (< 65%) and unusual GC content, which can result from PCR errors or contamination [11]. We retained 64 samples in total, including 10 replicates for controls and all chronic treatments and 8 replicates for each acute treatment (Additional File 1: Table S2).

### Differential gene expression analysis

We summarized kallisto pseudo-aligned transcript abundances to the gene level with tximport v1.14 [69] using transcript-to-gene tables retrieved with biomaRt v2.48.3. We excluded genes with low expression levels applying a cut-off of at least eight samples with a count of 10 transcripts or higher, retaining 9735 out of 10,661 genes with mapped reads. We transformed counts using the variance stabilizing transformation (VST, *blind* = *FALSE*) and performed principal component analysis on all remaining genes to assess the relationships of the samples. To detect differentially expressed genes between exposure treatments and the control, we applied Wald tests on median-of-ratios normalized counts in DESeq2 v1.32.0 [43]. We included treatment and source colony as factors in the model design. We report genes as differentially expressed using a significance cut-off of 0.05 after false discovery rate adjustment [5] of the Wald test *p*-values (FDR).

### Gene Ontology enrichment analysis

To test which Gene Ontology terms were overrepresented in response to the treatments, we performed gene ontology enrichment analysis using g:GOSt option of gprofiler2 v0.2.1 [39]. We sorted differentially expressed genes (Wald test’s FDR < 0.05) for each treatment by adjusted *p*-values change values and set the custom background genes to all genes expressed in control brain samples. We report Gene Ontology terms as enriched applying corrected for multiple testing g:SCS threshold of 0.05 derived from Fisher’s exact test.

### Cytochrome P450 expression analysis and phylogeny

We identified 51 putative cytochrome P450 orthologs for *B. terrestris* searching the proteome for the Pfam PF00067.25 domain and extracted them using HMMER v3.1b2 applying the *–cut_ga* option, which removes domains with conditional E-values greater than the internally established threshold. We selected the longest isoforms of the 51 *B. terrestris* and 49 *A. mellifera* P450 [13] cytochromes P450, and used the *mafft-linsi* option of MAFFT v7.480 [35] to perform multiple sequence alignment of amino acid sequences. We trimmed the aligned sequences using Jalview v2.11 [76] and TrimAl using *-automated1* option [9]. We used IQ-TREE v2.0.3 [49] to perform maximum likelihood phylogenetic inference using the *-MFP* option [34].

### Variable number of replicates in treatment groups

To examine whether the statistical power of detection of the differentially expressed genes changes due to the uneven number of replicates across exposure groups (8 in acute treatments and 10 in chronic treatments), we ran the analyses again using eight replicates per treatment. We randomly selected 8 samples for the control and chronic treatment and repeated the Wald test as implemented in DESeq2. We ran this analysis over 50 iterations making sure that the random exclusion of samples is unique for each iteration. We identified genes as differentially expressed applying a cut-off for false discovery rate (FDR) < 0.05. We built a distribution of the number of differentially expressed genes detected at each iteration to assess the probability of obtaining a higher number of differentially expressed genes for each of the acute treatments when the number of replicates is equal in all groups. None of the trends we report here changed under the alternate scenarios. Our analysis indicates that reducing the number of replicates in chronic treatments does not increase the power of detection of differentially expressed genes for acute treatments (permutation test, all *p* > 0.09). Therefore, we decided to retain all the suitable samples in downstream analysis to allow for a better estimation of the gene expression levels.

### Significance of overlaps of differentially expressed genes between treatments

To determine if the overlap between differentially expressed genes across various treatments was statistically significant, we used a simulation-based method, anchored in a hypergeometric testing framework. Specifically, we created 10,000 unique samples by randomly selecting genes from a combined pool of all expressed genes without replacing them once drawn. The number of genes drawn was determined by the actual number of differentially expressed genes under each treatment. For each comparison, we assessed the extent of overlap between treatments and compared to the actual observed overlap in the real data. We calculated the *p*-value as the proportion of simulated overlaps that were at least as extreme as the observed overlap, adjusted for the observed case itself.

### Food intake measurements

Every other day, we measured the food intake of each microcolony. We calculated the median pesticide dose [μg] per bee over exposure time for both acute and chronic exposures. Microcolonies exposed to acute clothianidin treatment, consumed a median of 0.017 μg of clothianidin, while microcolonies exposed to clothianidin at chronic regime consumed 0.019 μg. For acetamiprid, microcolonies consumed on average 0.036 and 0.039 μg of the compound under acute and chronic exposure respectively. Microcolonies exposed to sulfoxaflor consumed a median of 0.038 and 0.040 μg of the compound under acute and chronic exposure respectively.

We used a linear model to assess differences in food intake between the control and chronic treatment groups. Specifically, we used the average daily food intake per bee within a microcolony as the response variable and included the queenright colony of origin and the interaction between the day of exposure and treatment as explanatory variables. Our model returned a negative coefficient of − 0.063 with a small standard error of 0.015 for an interaction term between the day of exposure and chronic clothianidin treatment, indicating that the effect of chronic clothianidin exposure on per day food intake decreased by 0.063 mL for each consecutive measurement compared to the control (*p* < 10 − 5). We observed a similar trend applying a linear model with the same variables to food intake data from microcolonies exposed to acute doses of clothianidin (coefficient = − 0.51, SE = 0.11 *p* < 10 − 5). In this model, we only used measurements from days 12 (last day before exposure) and 14 (sampling day post exposure). We did not detect significant differences in the food intake between the control microcolonies and microcolonies exposed to sulfoxaflor and acetamiprid at either acute or chronic treatments.

The bees exposed to clothianidin consumed less compound compared to sulfoxaflor and acetamiprid. However, even at a lower total dose this compound caused the most changes. Most importantly, the accumulative dose consumed under acute and chronic exposures were comparable for all compounds. It is important to note that some of the changes detected in the differential gene expression analysis between control samples and bees exposed to chronic clothianidin may have been caused by lower food intake rather than exposure alone. Unfortunately, we were unable to differentiate between these two factors. Nonetheless, the observed lower food intake was a direct result of the exposure to clothianidin.

### Quantification of differences between source colonies

To detect differentially expressed genes between the source colonies, we used the same DESeq2 model that was used for the detection of differentially expressed genes between treatments, which included treatment and source colonies as explanatory variables. We report genes as differentially expressed using a significance cut-off of FDR < 0.05 (Additional File 1: Fig. S2). After mapping reads obtained from all microcolonies to the reference genome using STAR, we mapped variants using freeBayes v1.3.1 [23] and applied principal component analysis using SNPRelate v3.18 [81] to see whether there are differences in the genetic makeup between the source colonies. All colonies were separated by the first two principal components showing that the source colonies varied in their genetic background (Additional File 1: Fig. S3).

### Variable responses in bees exposed to acute pesticide treatments

We observed that the variance in gene expression between replicates in acute treatments is higher than in chronic and control treatments. To check if this pattern is statistically significant, we built a generalized mixed-effects model using lme4 R library. We used the standard deviation of genes as a response variable and treatment (acute or chronic) and differential expression status (differentially expressed or unchanged) of each gene as fixed explanatory variables; we assigned the gene as a random effect to account for a non-independence of the observations. To address issues related to heteroscedasticity and non-normality, we applied variance stabilizing transformation (VST) before calculating the standard deviation of gene expression. The use of VST helped to mitigate the presence of extreme values that were observed when standard deviation was calculated on un-normalized counts, which resulted in a skewed distribution of the residuals, and hindered our ability to fit a generalized linear model. Standard deviation of gene expression values after VST is more consistent across genes, as VST reduces the dependence of variance on the mean expression level. Therefore, if we still see differences in standard deviation between acute and chronic groups calculated using VST-normalized counts, the differences in standard deviation between genes in non-normalized counts should be greater. To ensure the best model fit for continuous data with a right-skewed distribution, we applied gamma distribution.

The model’s output showed that the interaction term between treatment and the expression status variables has a significant effect on the response variable, with a coefficient of 0.19, *t*-value of − 13.91, and *p* of < 10^−16^, suggesting standard deviation is higher for the acute group than for the chronic group when genes are differentially expressed. In the context of GLMM with a gamma distribution, the coefficient is on the log scale. Therefore, the standard deviation in the acute treatment group is approximately 20% higher on average compared to the chronic group.

### Differences in the scale of treatment effects

To assess whether differentially expressed genes under acute exposure are more likely to exhibit substantial changes in expression levels compared to those under chronic exposure, we calculated the proportion of genes showing at least a fourfold change in expression relative to the control. A chi-square test was utilized to determine whether this proportion was significantly higher in the acute treatment group. The findings indicated that 3.61% of differentially expressed genes under acute clothianidin treatment experienced at least a fourfold expression change, in contrast to only 0.75% of differentially expressed genes under chronic treatment (*χ*^2^ = 16.7, *p* < 0.00001). Moreover, the most extreme expression change under acute exposure to clothianidin was a 1487-fold increase, which is substantially larger than the 31-fold decrease seen with chronic exposure. Thus, we selected 20 genes with the most extreme changes under acute and chronic clothianidin treatments and ran the Welch two-sample *t*-test to compare the log2Fold changes between the gene sets. On average, the top 20 affected genes had an increase of expression 2.65 times higher under acute clothianidin exposure compared to the chronic exposure (*t* = 6.2, df = 26.4, *p* < 10^−6^).

## Supplementary Information

Additional file 1: Fig. S1. The power to detect differentially expressed genes depends on the agreement between the biological replicates within treatment groups and the similarities in the genetic background of individuals between groups. We controlled for the genetic background of the bumble bees by applying a microcolony-based experimental design, where replicates in all treatments came from the same ten source colonies and were, therefore, genetically related. To show this effect, we randomly selected control samples from five source colonies and clothianidin samples from the remaining five source colonies, so the bees in both treatments were not related. We iterated this process over 100 different selection combinations and built a distribution of the number of differentially expressed genes detected (blue distribution). Next, we repeated this process, but we randomly selected control and clothianidin samples from the same five source colonies, so the bees in both treatments were related (yellow distribution). We compared both distributions and tested whether it is more likely to obtain a higher number of differentially expressed genes when controlling for the source colony effect. When controlling for the colony effect, the median number of differentially expressed genes was 543 compared to 225 when the colony was not accounted for. The distribution when the colony was accounted for was also less positively skewed (skewness = 1.99) than when the colony was not accounted for (skewness = 0.53). We applied the Kolmogorov-Smirnov test to compare the distributions. We detected high numbers of differentially expressed genes using both strategies. However, we conclude that the two distributions are statistically significantly different (D = 0.21, *p*-value = 0.02). Therefore, more differentially expressed genes are detected by the DESeq2 algorithms when we control for the genetic background of the bees. Fig. S2. Heatmap of the number of differentially expressed genes (FDR < 0.05) detected between workers from the ten source colonies used to establish the microcolonies. We observed many differentially expressed genes between the 10 source colonies, with colonies two and three being particularly dissimilar. On average, we detected 445 differentially expressed genes between other colonies and colonies 2 or 3, compared to average 97 differentially expressed genes between other colony pairs. Importantly, microcolonies established using worker bees from each source colony were equally distributed among the treatments used. All colonies were purchased from a commercial breeder and were healthy. During the experiment all bees were kept under controlled conditions. Therefore, we expected the variation in gene expression to be driven by baseline-biological differences between the source colonies. We decided not to exclude colonies two and three from the analysis since the potential impact of these colonies on our results was mitigated by the presence of representative individuals from these source colonies in each treatment group. Fig. S3. Principal component analysis of the SNPs detected between the 10 source colonies. Each data point indicates a pool of three workers from all microcolonies assembled using the corresponding source colony. All microcolonies used in the differential gene expression analysis were used here. All source colonies were clearly separated indicating underlying genomic differences that were controlled for by including the source colony in the DESeq2 model design. Fig. S4. Experimental design. We obtained microcolonies from 10 source colonies and assigned to one of the seven treatments. All source colonies were two weeks old when we started arranging the microcolonies. We created microcolonies using callow workers. Workers within each microcolony emerged within 24h. Because of the differences in the pace of worker production, we created microcolonies in a staggered manner. The order in which we assigned treatments to the microcolonies was randomized. Our design ensured that treatments were assigned to microcolonies created at various stages of source colony development. Table S1. Differentially expressed cytochromes P450 under acute treatments and chronic clothianidin treatment. The direction of the arrows indicates up- or down-regulation of the differentially expressed genes. Table S2. Summary of all samples used in differential gene expression analysis.

Additional file 2: Table S3. Differentially expressed genes detected under the acute and chronic exposure treatments.

## Data Availability

Sequence data underlying this work are in the National Center for Biotechnology Information Short Read Archive, accession PRJNA1076820 (available upon publication). Analysis scripts are available on GitHub at https://github.com/wurmlab/2022–09-Bter-chronic-acute-exposure-alicja.
