# Supplementary material for: Acute and chronic pesticide exposure trigger fundamentally different molecular responses in bumble bee brains
Source: BMC Biol. 2025 Mar 11;23:72. doi: 10.1186/s12915-025-02169-z (PMC11900027; doi:10.1186/s12915-025-02169-z)
Supplement: Supplementary file 1 — Additional file 1: Fig. S1. The power to detect differentially expressed genes depends on the agreement between the biological replicates within treatment groups and the similarities in the genetic background of individuals between groups. We controlled for the genetic background of the bumble bees by applying a microcolony-based experimental design, where replicates in all treatments came from the same ten source colonies and were, therefore, genetically related. To show this effect, we randomly selected control samples from five source colonies and clothianidin samples from the remaining five source colonies, so the bees in both treatments were not related. We iterated this process over 100 different selection combinations and built a distribution of the number of differentially expressed genes detected (blue distribution). Next, we repeated this process, but we randomly selected control and clothianidin samples from the same five source colonies, so the bees in both treatments were related (yellow distribution). We compared both distributions and tested whether it is more likely to obtain a higher number of differentially expressed genes when controlling for the source colony effect. When controlling for the colony effect, the median number of differentially expressed genes was 543 compared to 225 when the colony was not accounted for. The distribution when the colony was accounted for was also less positively skewed (skewness = 1.99) than when the colony was not accounted for (skewness = 0.53). We applied the Kolmogorov-Smirnov test to compare the distributions. We detected high numbers of differentially expressed genes using both strategies. However, we conclude that the two distributions are statistically significantly different (D = 0.21, p-value = 0.02). Therefore, more differentially expressed genes are detected by the DESeq2 algorithms when we control for the genetic background of the bees. Fig. S2. Heatmap of the number of differentially expressed genes ( [file 12915_2025_2169_MOESM1_ESM.docx]

SUPPLEMENTAL INFORMATION

**Acute and chronic pesticide exposure trigger fundamentally different molecular responses in bumble bee brains**

Alicja Witwicka^1^, Federico López-Osorio^1^, Andres Arce^2^, Richard J Gill^3^, Yannick Wurm^1,4,5^

^1^ Biology Department, Queen Mary University of London, London, UK

^2^ Department of Biology, Edge Hill University, Ormskirk, Lancashire, UK

^3^ Georgina Mace Centre for the Living Planet, Department of Life Sciences, Silwood Park Campus, Imperial College London, UK

^4^ Digital Environment Research Institute, Queen Mary University of London, London, UK

^5^ Alan Turing Institute, London, UK

Figure S1. The power to detect differentially expressed genes depends on the agreement between the biological replicates within treatment groups and the similarities in the genetic background of individuals between groups. We controlled for the genetic background of the bumble bees by applying a microcolony-based experimental design, where replicates in all treatments came from the same ten source colonies and were, therefore, genetically related. To show this effect, we randomly selected control samples from five source colonies and clothianidin samples from the remaining five source colonies, so the bees in both treatments were not related. We iterated this process over 100 different selection combinations and built a distribution of the number of differentially expressed genes detected (blue distribution). Next, we repeated this process, but we randomly selected control and clothianidin samples from the same five source colonies, so the bees in both treatments were related (yellow distribution). We compared both distributions and tested whether it is more likely to obtain a higher number of differentially expressed genes when controlling for the source colony effect. When controlling for the colony effect, the median number of differentially expressed genes was 543 compared to 225 when the colony was not accounted for. The distribution when the colony was accounted for was also less positively skewed (skewness = 1.99) than when the colony was not accounted for (skewness = 0.53). We applied the Kolmogorov-Smirnov test to compare the distributions. We detected high numbers of differentially expressed genes using both strategies. However, we conclude that the two distributions are statistically significantly different (D = 0.21, p-value = 0.02). Therefore, more differentially expressed genes are detected by the DESeq2 algorithms when we control for the genetic background of the bees.

Figure S2. Heatmap of the number of differentially expressed genes (FDR < 0.05) detected between workers from the ten source colonies used to establish the microcolonies. We observed many differentially expressed genes between the 10 source colonies, with colonies two and three being particularly dissimilar. On average, we detected 445 differentially expressed genes between other colonies and colonies 2 or 3, compared to average 97 differentially expressed genes between other colony pairs. Importantly, microcolonies established using worker bees from each source colony were equally distributed among the treatments used. All colonies were purchased from a commercial breeder and were healthy. During the experiment all bees were kept under controlled conditions. Therefore, we expected the variation in gene expression to be driven by baseline-biological differences between the source colonies. We decided not to exclude colonies two and three from the analysis since the potential impact of these colonies on our results was mitigated by the presence of representative individuals from these source colonies in each treatment group.


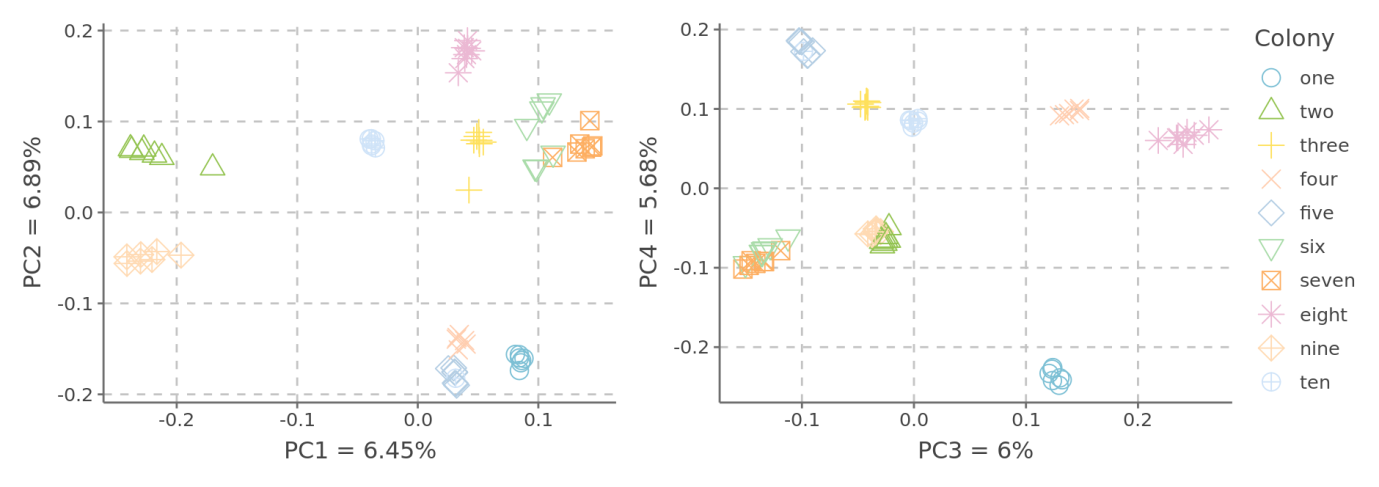


Figure S3. Principal component analysis of the SNPs detected between the 10 source colonies. Each data point indicates a pool of three workers from all microcolonies assembled using the corresponding source colony. All microcolonies used in the differential gene expression analysis were used here. All source colonies were clearly separated indicating underlying genomic differences that were controlled for by including the source colony in the DESeq2 model design.

Figure S4. Experimental design. We obtained microcolonies from 10 source colonies and assigned to one of the seven treatments. All source colonies were two weeks old when we started arranging the microcolonies. We created microcolonies using callow workers. Workers within each microcolony emerged within 24h. Because of the differences in the pace of worker production, we created microcolonies in a staggered manner. The order in which we assigned treatments to the microcolonies was randomized. Our design ensured that treatments were assigned to microcolonies created at various stages of source colony development.

Table S1. Differentially expressed cytochromes P450 under acute treatments and chronic clothianidin treatment. The direction of the arrows indicates up- or down-regulation of the differentially expressed genes.

Table S2. Summary of all samples used in differential gene expression analysis.
