# Supplementary material for: Acute and chronic pesticide exposure trigger fundamentally different molecular responses in bumble bee brains
Source: BMC Biol. 2025 Mar 11;23:72. doi: 10.1186/s12915-025-02169-z (PMC11900027; doi:10.1186/s12915-025-02169-z)
Supplement: Supplementary file 2 — Additional file 2: Table S3. Differentially expressed genes detected under the acute and chronic exposure treatments. [file 12915_2025_2169_MOESM2_ESM.pdf]

Table S3. Differentially expressed genes detected under the acute and chronic exposure treatments.

CHRONIC SULFOXAFLOR

Gene ID	log2Fold Change	Adjusted P-value (FDR)	Expression pattern	Gene annotation
Def	-4.588027081	6.82E-07	Down-regulated	defensin

CHRONIC ACETAMIPRID

Gene ID	log2Fold Change	Adjusted P-value (FDR)	Expression pattern	Gene annotation
Def	-5.375462956	7.00E-05	Down-regulated	defensin
LOC100631078	-3.606816832	3.33E-06	Down-regulated	abaecin

CHRONIC CLOTHIANIDIN

Gene ID	log2Fold Change	Adjusted P-value (FDR)	Expression pattern	Gene annotation
LOC100644602	1.105317594	1.42E-06	Up-regulated	mediator of RNA polymerase II transcription subunit 20
LOC100646142	1.098209721	1.42E-06	Up-regulated	glycine receptor subunit alpha-1
LOC100651332	0.424654415	2.55E-06	Up-regulated	cAMP-dependent protein kinase type I regulatory subunit
LOC100648391	0.882745706	6.83E-06	Up-regulated	cytochrome P450 6k1 LOC100648391
LOC100645349	0.526227088	2.28E-05	Up-regulated	spermine oxidase
LOC110119952	1.205450069	2.28E-05	Up-regulated	uncharacterized LOC110119952
LOC100643335	-0.347188444	3.04E-05	Down-regulated	acid phosphatase type 7
LOC100648049	-0.282523579	4.84E-05	Down-regulated	protein MTO1 homolog
LOC100651942	1.044583694	5.29E-05	Up-regulated	cilia- and flagella-associated protein 58
LOC100646587	0.631374562	6.86E-05	Up-regulated	phospholipase B1
Def	-4.945798782	8.18E-05	Down-regulated	defensin
LOC100643272	-0.562979132	8.18E-05	Down-regulated	HIG1 domain family member 1C
LOC105665823	0.470377632	8.18E-05	Up-regulated	secretion-regulating guanine nucleotide exchange factor
LOC100652251	0.721227661	9.74E-05	Up-regulated	RYamide receptor
LOC100642530	0.753426681	1.59E-04	Up-regulated	sodium-independent sulfate anion transporter
LOC105666345	-1.050735548	2.76E-04	Down-regulated	uncharacterized LOC105666345
LOC100645828	0.896643305	2.84E-04	Up-regulated	dystroglycan 1
LOC100647277	0.762472873	2.84E-04	Up-regulated	IQ and AAA domain-containing protein 1-like
LOC100652150	1.173818013	2.84E-04	Up-regulated	BMP-binding endothelial regulator protein
LOC100649555	-0.739893188	3.36E-04	Down-regulated	protein ABHD11
LOC100644025	0.634964523	3.41E-04	Up-regulated	endoribonuclease CG2145
LOC100643082	1.066659197	4.28E-04	Up-regulated	UPF0489 protein C5orf22 homolog
LOC100643009	-0.418442236	4.36E-04	Down-regulated	protein stunted
LOC100645024	1.34883417	4.36E-04	Up-regulated	apyrase
LOC100645386	0.589767747	4.36E-04	Up-regulated	protein 5NUC
LOC100647889	0.378983168	4.36E-04	Up-regulated	vascular endothelial growth factor A-A
LOC100648079	-1.445480114	4.36E-04	Down-regulated	endoglucanase E-4
LOC100650431	0.675454426	4.38E-04	Up-regulated	jmjC domain-containing histone demethylation protein 1
LOC100649251	0.504580104	4.50E-04	Up-regulated	vitellogenin
LOC100644732	0.460746193	4.93E-04	Up-regulated	protein eiger
LOC100644229	0.489846481	7.70E-04	Up-regulated	heat shock 70 kDa protein cognate 4
LOC100643329	-0.373647579	8.75E-04	Down-regulated	asparagine synthetase domain-containing protein 1
LOC100650043	0.385187145	9.10E-04	Up-regulated	calcium-activated potassium channel slowpoke
LOC100642296	2.386706376	9.65E-04	Up-regulated	keratin-associated protein 19-2
LOC100645663	0.319209247	9.65E-04	Up-regulated	protein ELYS
LOC100647817	0.427457087	9.65E-04	Up-regulated	choline/ethanolamine kinase
LOC100648632	-0.225270919	9.82E-04	Down-regulated	heat shock protein 75 kDa
LOC100644938	0.3403012	0.001021412	Up-regulated	organic cation transporter protein
LOC100647466	-0.418194935	0.001135407	Down-regulated	NADH dehydrogenase [ubiquinone] 1 beta subcomplex subunit 8
LOC110119161	-0.443888256	0.001181786	Down-regulated	28S ribosomal protein S31
LOC100642714	0.217590022	0.00118775	Up-regulated	formin-binding protein 4
LOC100644442	-0.317171821	0.00118775	Down-regulated	uncharacterized LOC100644442
LOC100644476	0.343845158	0.00118775	Up-regulated	transforming growth factor-beta-induced protein ig-h3
LOC100646771	-0.36099043	0.00118775	Down-regulated	protein pelota
LOC100647762	0.314301321	0.001219427	Up-regulated	sorting nexin-24
LOC100651076	0.572242814	0.001226942	Up-regulated	voltage-dependent calcium channel subunit alpha-2/delta-3
LOC100646048	0.416777187	0.001298041	Up-regulated	aminopeptidase N
LOC100645015	-0.372511777	0.001300619	Down-regulated	snRNA-activating protein complex subunit 3
LOC100648383	0.773314036	0.001317764	Up-regulated	dynein heavy chain 5
LOC100649002	0.275299135	0.001317764	Up-regulated	poly(U)-binding-splicing factor half pint
LOC100631078	-3.476382657	0.001363934	Down-regulated	abaecin
LOC100643697	0.247078466	0.001399214	Up-regulated	rabenosyn-5
LOC100647229	-1.711996812	0.001399214	Down-regulated	troponin C
LOC100647656	0.327067058	0.001399214	Up-regulated	uncharacterized LOC100647656
LOC100648275	0.609054511	0.001399214	Up-regulated	DNA topoisomerase 2-binding protein 1
LOC100651278	-0.309535479	0.001399214	Down-regulated	sialin
LOC105666438	0.762783411	0.001399214	Up-regulated	uncharacterized LOC105666438
LOC110120273	-0.616754213	0.001399214	Down-regulated	uncharacterized LOC110120273
LOC100644651	0.277459332	0.00159968	Up-regulated	NAD-dependent protein deacetylase sirtuin-7
LOC100649276	-0.448331366	0.001840918	Down-regulated	activator of 90 kDa heat shock protein ATPase homolog 1
LOC100643661	-0.31569364	0.001856009	Down-regulated	translation factor GUF1 homolog
LOC100647285	1.025989696	0.001856009	Up-regulated	facilitated trehalose transporter Tret1
LOC100650397	-0.353545996	0.001856009	Down-regulated	uncharacterized protein C4B3.18
LOC100649962	-0.403666481	0.001970047	Down-regulated	ribosomal protein 63
LOC100647941	0.785918012	0.00250464	Up-regulated	uncharacterized protein DDB_G0283697
LOC100643507	-0.338585945	0.002575966	Down-regulated	trypsin-3
LOC100646243	1.198342743	0.002575966	Up-regulated	uncharacterized LOC100646243
LOC105666757	0.795902605	0.002575966	Up-regulated	uncharacterized LOC105666757
LOC100643514	-0.748297628	0.002627095	Down-regulated	ejaculatory bulb-specific protein 3
LOC100644174	0.787729383	0.002627095	Up-regulated	flocculation protein FLO11
LOC100645134	-0.353762714	0.002627095	Down-regulated	transmembrane protein 19

LOC100650598	-0.510757179	0.002635299	Down-regulated	zinc finger protein 41
LOC100651466	-0.358288819	0.002635299	Down-regulated	nonsense-mediated mRNA decay factor SMG9
LOC100643597	-0.347149823	0.002649081	Down-regulated	synembryn-A
LOC100643565	0.30541956	0.002658711	Up-regulated	leucine-rich repeat-containing protein 19
LOC100643722	0.415433712	0.002912379	Up-regulated	protein eva-1
LOC100644112	0.633746933	0.002979536	Up-regulated	fructose-bisphosphate aldolase
LOC100647716	-0.458995078	0.002979536	Down-regulated	uncharacterized LOC100647716
LOC100649524	0.461152855	0.002979536	Up-regulated	40S ribosomal protein S6
LOC110120252	0.377609924	0.002979536	Up-regulated	uncharacterized LOC110120252
LOC100644393	0.406626455	0.003256589	Up-regulated	peptidyl-prolyl cis-trans isomerase G
LOC100644831	-0.618698191	0.003323637	Down-regulated	asparagine synthetase [glutamine-hydrolyzing]
LOC100649384	0.494298935	0.003323637	Up-regulated	neprilysin-4
LOC100647758	-0.254764035	0.003384463	Down-regulated	uncharacterized LOC100647758
LOC100644065	0.204796319	0.003412852	Up-regulated	myotubularin-related protein 9
LOC100651274	0.320130018	0.003450417	Up-regulated	bromodomain-containing protein 4
LOC100650813	0.374762224	0.003531124	Up-regulated	BTB/POZ domain-containing protein 6-B
LOC100644259	-0.422545203	0.003565295	Down-regulated	LDLR chaperone boca
LOC100645257	0.692395117	0.003623884	Up-regulated	mucin-3A
LOC100646909	2.334598763	0.003623884	Up-regulated	uncharacterized LOC100646909
LOC100642347	-0.344323381	0.003642011	Down-regulated	transcription factor MafG
LOC100644435	-0.444393692	0.003704942	Down-regulated	E3 ubiquitin-protein ligase RNF14
LOC100645702	-3.326307033	0.003704942	Down-regulated	kynurenine/alpha-aminoadipate aminotransferase
LOC100648622	0.304548377	0.003704942	Up-regulated	histone-lysine N-methyltransferase EHMT2
LOC100652279	1.00194302	0.003704942	Up-regulated	uncharacterized LOC100652279
LOC100644971	-0.42548833	0.003759973	Down-regulated	elongation factor Ts
LOC100646282	-0.546020351	0.003832383	Down-regulated	transmembrane protein 42
LOC100642370	0.418283535	0.003984809	Up-regulated	uncharacterized protein C1orf112 homolog
LOC100644613	0.22193073	0.004067097	Up-regulated	cholinesterase
LOC100643346	0.232668704	0.004125988	Up-regulated	PAXIP1-associated glutamate-rich protein 1
LOC100651986	0.262271498	0.004176112	Up-regulated	chromodomain-helicase-DNA-binding protein 1
LOC100644984	0.387091515	0.00434131	Up-regulated	myb-like protein X
LOC100645710	0.637055947	0.00434131	Up-regulated	uncharacterized LOC100645710
LOC100642939	-0.280442754	0.004438353	Down-regulated	sulfide:quinone oxidoreductase
LOC100651542	-0.877224013	0.004475281	Down-regulated	dendritic arbor reduction protein 1
LOC110120083	0.992727865	0.004646466	Up-regulated	uncharacterized LOC110120083
LOC100651555	-0.408960041	0.004677982	Down-regulated	DNA-directed RNA polymerase II subunit RPB11
LOC100644531	0.274846547	0.004686835	Up-regulated	rho GTPase-activating protein 44
LOC100650704	0.469545251	0.004695436	Up-regulated	fructose-1
LOC100645265	-0.569091079	0.004841278	Down-regulated	transcription termination factor 5
LOC100647930	0.461593524	0.005137964	Up-regulated	cyclin-dependent kinase-like 4
LOC100652057	-0.329339537	0.005217538	Down-regulated	probable proline--tRNA ligase
LOC100642548	0.71569837	0.00523367	Up-regulated	hyaluronan mediated motility receptor-like
LOC100649548	-0.318751877	0.00523367	Down-regulated	trans-1
LOC100651203	0.273856049	0.00523367	Up-regulated	ubiquitin carboxyl-terminal hydrolase 31
LOC105665756	-0.249091612	0.00523367	Down-regulated	ubiquinone biosynthesis monooxygenase COQ6
LOC100649178	0.613821938	0.005268382	Up-regulated	ras-like protein family member 11B
LOC100650118	-0.430927989	0.00562443	Down-regulated	pyrimidodiazepine synthase
LOC100642205	0.40843529	0.005637916	Up-regulated	protein krueppel
LOC100647510	0.232581528	0.005746526	Up-regulated	uncharacterized LOC100647510
LOC100644444	0.454223785	0.005851474	Up-regulated	pregnancy zone protein
LOC100645292	-0.287147993	0.005851474	Down-regulated	dehydrogenase/reductase SDR family member 4
LOC100646070	0.482684594	0.005851474	Up-regulated	40S ribosomal protein S6
LOC100646089	-0.751290547	0.005851474	Down-regulated	uncharacterized LOC100646089
LOC100647736	0.168630398	0.005851474	Up-regulated	histone-lysine N-methyltransferase E(z)
LOC100651060	1.404778121	0.005851474	Up-regulated	uncharacterized LOC100651060
LOC100651222	-0.359139287	0.005851474	Down-regulated	probable 28S ribosomal protein S16
LOC105666304	-0.814067945	0.005851474	Down-regulated	pre-mRNA-splicing factor CWC22 homolog
LOC100644715	0.558628065	0.005851545	Up-regulated	polypeptide N-acetylgalactosaminyltransferase 2
LOC100650928	-0.586495237	0.005857715	Down-regulated	uncharacterized LOC100650928
LOC100650903	0.952465577	0.005965877	Up-regulated	cadherin-89D
LOC100650034	-0.412619513	0.005994101	Down-regulated	recQ-mediated genome instability protein 1
LOC100647283	-0.375810994	0.006069364	Down-regulated	bidirectional sugar transporter N3
LOC100651461	-0.630141276	0.006088987	Down-regulated	dihydrofolate reductase
LOC105666713	0.644277533	0.006123398	Up-regulated	uncharacterized LOC105666713
LOC100649426	0.774633302	0.006287053	Up-regulated	probable chitinase 2
LOC100642744	0.378783691	0.006350214	Up-regulated	45 kDa calcium-binding protein
LOC100643576	-0.477384713	0.006350214	Down-regulated	S-adenosylmethionine mitochondrial carrier protein
LOC100644910	-0.256683024	0.006350214	Down-regulated	phosphatidylinositol transfer protein
LOC100649210	0.576504735	0.006350214	Up-regulated	insulin-like growth factor-binding protein complex acid labile subunit
LOC100650456	0.31985509	0.006350214	Up-regulated	protein N-terminal glutamine amidohydrolase
LOC100645809	-0.264380466	0.006796063	Down-regulated	aspartate--tRNA ligase
LOC100647840	0.42168283	0.006796063	Up-regulated	diuretic hormone receptor
LOC100650912	0.274468171	0.006796063	Up-regulated	RNA-binding protein squid
LOC100651221	1.007661713	0.006796063	Up-regulated	probable chitinase 10
LOC100644083	-0.148729794	0.006876646	Down-regulated	NADH dehydrogenase [ubiquinone] 1 alpha subcomplex subunit 9
LOC100649224	-0.542777312	0.006876646	Down-regulated	UPF0598 protein CG30010
LOC100650181	0.29616149	0.007166526	Up-regulated	acidic leucine-rich nuclear phosphoprotein 32 family member B
LOC100642945	0.414554331	0.007248921	Up-regulated	papilin
LOC100646185	-0.221320938	0.007248921	Down-regulated	SUMO-activating enzyme subunit 2
LOC100643728	-0.341905766	0.007345542	Down-regulated	laccase-1
LOC110119143	-0.666374756	0.007365788	Down-regulated	D-aminoacyl-tRNA deacylase 1

LOC100647029	0.3583159	0.007383404	Up-regulated	protein kinase C-binding protein 1
LOC100644315	-0.454910972	0.007519786	Down-regulated	mitochondrial ornithine transporter 1
LOC100644264	-0.295264299	0.007605992	Down-regulated	cytochrome b-c1 complex subunit 6
LOC100646557	-0.211724429	0.007605992	Down-regulated	ATP synthase subunit d
LOC100652139	-0.350029816	0.007605992	Down-regulated	PRADC1-like protein
LOC100647617	-0.821327845	0.007661375	Down-regulated	N-acetylglucosamine-6-sulfatase
LOC100649886	-0.367001581	0.00773698	Down-regulated	mitochondrial potassium channel ATP-binding subunit
LOC100651592	0.280480734	0.00773698	Up-regulated	polyhomeotic-like protein 2
LOC100652253	0.318231249	0.00773698	Up-regulated	potassium voltage-gated channel subfamily H member 6
LOC110120345	-0.805051989	0.00773698	Down-regulated	origin recognition complex subunit 5
LOC100643939	1.040369849	0.007827234	Up-regulated	uncharacterized LOC100643939
LOC100650432	0.517001475	0.007887483	Up-regulated	TNF receptor-associated factor 4
LOC100645612	0.3780396	0.007954184	Up-regulated	uncharacterized LOC100645612
LOC100643797	0.253779085	0.008021359	Up-regulated	phospholipase ABHD3
LOC100647295	-0.594913488	0.008021359	Down-regulated	neuroparsin-A
LOC100648547	0.604833642	0.008021359	Up-regulated	excitatory amino acid transporter
LOC100642401	-0.264753119	0.008031563	Down-regulated	cytochrome c oxidase subunit 5A
LOC100648791	-0.251344993	0.008031563	Down-regulated	acylphosphatase-1
LOC100631058	0.683655944	0.008050871	Up-regulated	small heat shock protein
LOC100651873	0.552770971	0.008050871	Up-regulated	RNA-binding protein Rsf1
LOC100649313	-0.205401902	0.008053103	Down-regulated	NADH dehydrogenase [ubiquinone] flavoprotein 2
LOC100651269	-0.503541321	0.008053103	Down-regulated	39S ribosomal protein L42
LOC100650415	0.602306678	0.008229012	Up-regulated	DNA helicase MCM9
LOC100650477	-0.456589072	0.008272945	Down-regulated	calcyclin-binding protein
LOC100642899	-0.266825003	0.008389169	Down-regulated	protein artichoke
LOC100645209	0.24110215	0.008389169	Up-regulated	ATP-binding cassette sub-family C member 4
LOC105666721	-0.396367549	0.008389169	Down-regulated	uncharacterized LOC105666721
LOC100642445	-0.282163932	0.008414155	Down-regulated	tRNA-specific adenosine deaminase 1
LOC100644734	0.542480445	0.008414155	Up-regulated	single Ig IL-1-related receptor
LOC110120082	0.892439373	0.00842798	Up-regulated	uncharacterized LOC110120082
LOC100649382	-0.645637309	0.008523002	Down-regulated	alpha-N-acetylglucosaminidase
LOC105667138	-0.252041262	0.008523002	Down-regulated	acyl carrier protein
LOC100644568	-0.260654543	0.008757614	Down-regulated	ATP synthase-coupling factor 6
LOC100644766	0.569017784	0.008757614	Up-regulated	soluble guanylate cyclase 89Da
LOC100646920	-0.689227766	0.008757614	Down-regulated	uncharacterized LOC100646920
LOC100648347	0.221664097	0.00878591	Up-regulated	zinc finger RNA-binding protein
LOC100647103	0.673886774	0.008790148	Up-regulated	uncharacterized LOC100647103
LOC100647314	0.435113821	0.008790148	Up-regulated	uncharacterized LOC100647314
LOC100651339	-0.311434256	0.008933119	Down-regulated	leucine-rich repeat-containing protein 57
LOC100644265	-0.516477833	0.009254914	Down-regulated	succinate dehydrogenase assembly factor 3
LOC100649126	-0.26933576	0.009254914	Down-regulated	cytochrome c oxidase assembly factor 3
LOC100647062	-0.352066954	0.009282215	Down-regulated	39S ribosomal protein L40
LOC100643220	0.133704435	0.00930785	Up-regulated	R3H and coiled-coil domain-containing protein 1
LOC110120209	0.762627001	0.00930785	Up-regulated	uncharacterized LOC110120209
LOC100643580	0.506569188	0.009435401	Up-regulated	purine nucleoside phosphorylase
LOC100644099	-1.201218395	0.009435401	Down-regulated	venom acid phosphatase Acph-1
LOC100647010	-0.234548071	0.009435401	Down-regulated	elongation factor G
LOC100648474	0.170364101	0.009435401	Up-regulated	SAP30-binding protein
LOC100645102	0.288095368	0.009546956	Up-regulated	uncharacterized LOC100645102
LOC105666410	-0.433466375	0.009546956	Down-regulated	translation machinery-associated protein 7 homolog
LOC100642230	-0.35291558	0.009594797	Down-regulated	ubiquinol-cytochrome-c reductase complex assembly factor 1
LOC100642901	-0.236404291	0.009729653	Down-regulated	NADH-quinone oxidoreductase subunit I
LOC100642535	-0.65368269	0.009962336	Down-regulated	probable nuclear hormone receptor HR38
LOC100646882	0.438799428	0.009967993	Up-regulated	protein I'm not dead yet
LOC100650941	-0.573487213	0.009967993	Down-regulated	peptidyl-prolyl cis-trans isomerase A
LOC100652248	-0.401210357	0.009967993	Down-regulated	uncharacterized LOC100652248
LOC100644950	0.292501107	0.01005147	Up-regulated	CXXC-type zinc finger protein 1
LOC100651676	0.184832763	0.01005147	Up-regulated	E3 ubiquitin-protein ligase RNF38
LOC100649363	0.304873725	0.010098148	Up-regulated	uncharacterized LOC100649363
LOC100650004	-0.31674473	0.010139666	Down-regulated	cytidine deaminase
LOC100650814	0.359116739	0.010139666	Up-regulated	uncharacterized LOC100650814
LOC105666736	-0.278880607	0.01019078	Down-regulated	probable phosphatase phospho2
LOC100631088	0.939434645	0.010207937	Up-regulated	phosphoenolpyruvate carboxykinase
LOC100642965	1.020874758	0.010217884	Up-regulated	uncharacterized LOC100642965
LOC100651927	-0.348073116	0.010234858	Down-regulated	28S ribosomal protein S28
LOC105667088	0.47045172	0.010234858	Up-regulated	uncharacterized LOC105667088
LOC100642932	0.457567034	0.010341719	Up-regulated	microtubule-associated protein futsch
LOC100644007	-0.426860514	0.010341719	Down-regulated	alpha-methylacyl-CoA racemase
LOC100644498	0.272465998	0.010341719	Up-regulated	C-mannosyltransferase dpy-19
LOC100646760	0.443683627	0.010341719	Up-regulated	uncharacterized LOC100646760
LOC100652171	0.291866528	0.010341719	Up-regulated	UPF0430 protein CG31712
LOC100642973	1.298967061	0.010367037	Up-regulated	pro-resilin
LOC100644995	-1.324545842	0.010381	Down-regulated	uncharacterized LOC100644995
LOC100646594	0.288165185	0.010427753	Up-regulated	nuclear pore complex protein Nup98-Nup96
LOC110119463	-0.286229751	0.010613321	Down-regulated	uncharacterized LOC110119463
LOC100647673	-0.438282772	0.010653299	Down-regulated	insulin-like growth factor-binding protein complex acid labile subunit
Mpk	-0.612867598	0.010699046	Down-regulated	phosphomevalonate kinase
LOC100643012	0.534139191	0.010929824	Up-regulated	serine/threonine-protein kinase fused
LOC100648272	0.431174258	0.010935698	Up-regulated	DNA (cytosine-5)-methyltransferase 3B
LOC100648408	0.183294749	0.010935698	Up-regulated	lethal(3)malignant brain tumor-like protein 3
LOC100648731	0.836282256	0.011145393	Up-regulated	angiopoietin-2

LOC100646121	0.369335712	0.011173177	Up-regulated	nuclear pore complex protein DDB_G0274915
LOC100647094	0.323019415	0.011173177	Up-regulated	zinc transporter 1
LOC100643812	0.250769744	0.011200885	Up-regulated	zinc finger protein 37
LOC100649792	-0.548086853	0.011200885	Down-regulated	N(4)-(Beta-N-acetylglucosaminyl)-L-asparaginase
LOC100650758	-0.169820921	0.011200885	Down-regulated	succinate dehydrogenase [ubiquinone] iron-sulfur subunit
LOC100651213	-0.283493175	0.011200885	Down-regulated	transmembrane emp24 domain-containing protein 5
LOC100650808	-0.689978909	0.011225162	Down-regulated	annulin
LOC100645585	0.795017556	0.011319787	Up-regulated	uncharacterized LOC100645585
LOC100646385	-0.592240573	0.011319787	Down-regulated	lipid storage droplets surface-binding protein 1
LOC100642713	-0.56711957	0.011562734	Down-regulated	peptidyl-prolyl cis-trans isomerase FKBP4
LOC100644661	0.72833391	0.011562734	Up-regulated	facilitated trehalose transporter Tret1
LOC100645679	0.531973289	0.011562734	Up-regulated	peptide transporter family 1
LOC100647373	-0.280001387	0.011562734	Down-regulated	NFU1 iron-sulfur cluster scaffold homolog
LOC100649041	-0.563418578	0.011654071	Down-regulated	uncharacterized LOC100649041
LOC100644674	0.228366664	0.011670857	Up-regulated	U3 small nucleolar RNA-interacting protein 2
LOC100648691	1.550788212	0.011863589	Up-regulated	sodium-dependent nutrient amino acid transporter 1
LOC100646745	-0.361812484	0.011902781	Down-regulated	uncharacterized LOC100646745
LOC100648675	-0.31557201	0.012008796	Down-regulated	sodium/hydrogen exchanger 8
LOC105666132	0.437008935	0.012008796	Up-regulated	DNA mismatch repair protein MLH3
LOC100648722	0.301270943	0.01216629	Up-regulated	negative elongation factor A
LOC100647442	-0.356410773	0.0122004	Down-regulated	mitochondrial 2-oxodicarboxylate carrier
LOC100643845	0.422690667	0.012253072	Up-regulated	carbohydrate-responsive element-binding protein
LOC100646833	0.829683555	0.012273098	Up-regulated	histone demethylase UTY
LOC100647476	-0.35294601	0.012273098	Down-regulated	cytochrome c oxidase copper chaperone
LOC105667024	0.323099554	0.012273098	Up-regulated	glutathione S-transferase D7
LOC100647355	-0.426975787	0.012326758	Down-regulated	uncharacterized LOC100647355
LOC100643021	-0.448074308	0.012485851	Down-regulated	enoyl-CoA hydratase domain-containing protein 3
LOC100642253	-0.374480404	0.012884439	Down-regulated	succinate--hydroxymethylglutarate CoA-transferase
LOC100647093	-0.303001123	0.012884439	Down-regulated	probable nuclear transport factor 2
LOC100648549	-0.219963017	0.012884439	Down-regulated	cytochrome c
LOC100644757	-0.318722126	0.01290782	Down-regulated	tubulin--tyrosine ligase-like protein 12
LOC100649739	-0.29670011	0.013126763	Down-regulated	glutamate--cysteine ligase catalytic subunit
LOC100649732	-0.280766924	0.013174236	Down-regulated	WD repeat-containing protein 13
LOC100642448	-0.406729654	0.013189468	Down-regulated	transferrin receptor protein 1
LOC100649237	-0.378062256	0.013194628	Down-regulated	uncharacterized protein F13E9.13
LOC100651314	-0.259825863	0.013196864	Down-regulated	ras-related protein Rab-30
LOC100647134	0.321673668	0.013431458	Up-regulated	synaptotagmin-6
LOC100651403	-0.19887873	0.013431458	Down-regulated	carboxypeptidase D
LOC100652056	-0.371246909	0.013431458	Down-regulated	3-hydroxyisobutyryl-CoA hydrolase
LOC100650224	0.342053762	0.013515207	Up-regulated	protein sneaky
LOC100644237	-0.414969583	0.013547334	Down-regulated	cytochrome b-c1 complex subunit 9
LOC100644699	-0.318356502	0.01368142	Down-regulated	insulin-degrading enzyme
LOC100642579	0.270256734	0.013773392	Up-regulated	prohormone-4
LOC100642912	0.542388142	0.013787955	Up-regulated	probable phospholipid hydroperoxide glutathione peroxidase
LOC100650442	0.22519492	0.013787955	Up-regulated	zinc finger protein on ecdysone puffs
LOC100649770	0.218264941	0.013939219	Up-regulated	transcriptional repressor p66-beta
LOC100650426	1.132939178	0.014262464	Up-regulated	loricrin
LOC100651434	0.471581321	0.014262464	Up-regulated	40S ribosomal protein S3-like
LOC100644607	0.249175196	0.014278607	Up-regulated	KAT8 regulatory NSL complex subunit 1
LOC100648420	-0.317628551	0.01429732	Down-regulated	vacuolar protein sorting-associated protein 29
LOC100651046	1.026608867	0.014350121	Up-regulated	uncharacterized LOC100651046
LOC100648346	-0.276508331	0.014409094	Down-regulated	aldehyde dehydrogenase 1A1
LOC100645942	0.236196569	0.014474419	Up-regulated	double-strand-break repair protein rad21 homolog
LOC100645810	0.361449068	0.014570185	Up-regulated	telomere attrition and p53 response 1 protein
LOC100646379	0.162915146	0.01458227	Up-regulated	mitochondrial import receptor subunit TOM22 homolog
LOC105666933	-0.702580746	0.01458227	Down-regulated	E3 ubiquitin-protein ligase MARCHF3-like
LOC100643029	-0.382279848	0.014682456	Down-regulated	multifunctional methyltransferase subunit TRM112-like protein
LOC100643422	0.382208873	0.014682456	Up-regulated	arginine-glutamic acid dipeptide repeats protein
LOC100646773	0.215657242	0.014778791	Up-regulated	afadin
LOC100647923	-0.221820979	0.014800557	Down-regulated	zinc finger CCCH domain-containing protein 15 homolog
LOC100647540	1.835047438	0.014906198	Up-regulated	uncharacterized LOC100647540
LOC100650975	0.261708678	0.015103687	Up-regulated	methionine aminopeptidase 2
LOC105665953	-0.19786281	0.015111699	Down-regulated	uncharacterized LOC105665953
LOC100644557	0.69513162	0.015116681	Up-regulated	sushi
LOC100644102	0.315095923	0.015155844	Up-regulated	uncharacterized LOC100644102
LOC100644441	-0.487595092	0.015172716	Down-regulated	tubulin delta chain
LOC100642739	0.760741408	0.015366332	Up-regulated	facilitated trehalose transporter Tret1
LOC100648263	-0.20698586	0.015397008	Down-regulated	acyl-CoA-binding protein
LOC100643425	-0.418543969	0.015428205	Down-regulated	uncharacterized LOC100643425
LOC100650486	-0.244023883	0.015428205	Down-regulated	lysosomal alpha-glucosidase
LOC100644308	-0.170410092	0.015918106	Down-regulated	NAD-dependent protein deacylase sirtuin-5
LOC100652286	-0.314183821	0.01612916	Down-regulated	uncharacterized LOC100652286
LOC100650355	0.250211265	0.016146635	Up-regulated	host cell factor
LOC100644377	-1.463904415	0.016237089	Down-regulated	uncharacterized LOC100644377
LOC100648342	-1.194040824	0.016237089	Down-regulated	probable 28S ribosomal protein S26
LOC100650938	0.717195667	0.016237089	Up-regulated	general odorant-binding protein 83a
LOC100644916	0.283632931	0.016263182	Up-regulated	hydroxylysine kinase
LOC100649773	-0.340334344	0.016309504	Down-regulated	vacuolar protein sorting-associated protein 26C
LOC100646396	-0.192642734	0.01631586	Down-regulated	UPF0415 protein C7orf25 homolog
LOC100643731	-1.022116362	0.016324968	Down-regulated	maltase 1
LOC100645630	-0.590878286	0.016324968	Down-regulated	RISC-loading complex subunit TARBP2

LOC100645002	-0.368572252	0.016347271	Down-regulated	dnaJ protein homolog 1
LOC100647296	0.248935297	0.016462171	Up-regulated	translation elongation factor 2
LOC100643538	-0.187775914	0.016553297	Down-regulated	cytochrome c1
LOC100652047	1.268087146	0.016553297	Up-regulated	chitin deacetylase 1
LOC100644234	-0.350218055	0.016648342	Down-regulated	biogenesis of lysosome-related organelles complex 1 subunit 4
LOC110119486	0.356713598	0.016963963	Up-regulated	uncharacterized LOC110119486
LOC100644505	-0.336413643	0.017123955	Down-regulated	arginine-hydroxylase NDUFAF5
LOC100650203	0.711641196	0.017123955	Up-regulated	heat shock 70 kDa protein cognate 4
LOC100645617	-0.312596079	0.017126224	Down-regulated	mitochondrial import inner membrane translocase subunit Tim10
LOC100647566	0.276216879	0.017298775	Up-regulated	cytochrome P450 9e2 LOC100647566
LOC100642791	-0.296625287	0.017306852	Down-regulated	ER membrane protein complex subunit 8/9 homolog
LOC100643526	-0.274625936	0.017418415	Down-regulated	thioredoxin-2
LOC100651197	-0.34873968	0.017418415	Down-regulated	aldo-keto reductase family 1 member A1
LOC100644091	-0.250670887	0.017446772	Down-regulated	DNA-directed RNA polymerases I
LOC100644207	-0.401019525	0.017446772	Down-regulated	zinc finger protein 569
LOC100645222	-0.245417844	0.017446772	Down-regulated	aminoacylase-1
LOC100646598	0.305438297	0.017446772	Up-regulated	pyrroline-5-carboxylate reductase 2
LOC100649168	-0.358724325	0.017446772	Down-regulated	28S ribosomal protein S31
LOC100649233	0.299820498	0.017446772	Up-regulated	sodium-independent sulfate anion transporter
LOC100651186	-0.535411337	0.017446772	Down-regulated	6-pyruvoyl tetrahydrobiopterin synthase
LOC100651615	-0.220609332	0.017446772	Down-regulated	peptidyl-prolyl cis-trans isomerase-like 1
LOC105666150	0.607507735	0.017446772	Up-regulated	histone demethylase UTY
LOC110119575	0.453026188	0.017446772	Up-regulated	peroxiredoxin-2-like
LOC100645929	-0.291951786	0.017497558	Down-regulated	CDGSH iron-sulfur domain-containing protein 2 homolog
LOC100647322	1.332282774	0.017497558	Up-regulated	gustatory receptor for sugar taste 64f-like
LOC100652262	0.196790635	0.017666979	Up-regulated	NADPH--cytochrome P450 reductase
LOC100647980	-0.447126741	0.017761139	Down-regulated	UPF0545 protein C22orf39 homolog
LOC100649993	0.186321078	0.017761139	Up-regulated	heterogeneous nuclear ribonucleoprotein R
LOC100651079	-0.352762815	0.017761139	Down-regulated	glucosamine-6-phosphate isomerase
LOC100652235	-0.495579165	0.017761139	Down-regulated	checkpoint protein HUS1
LOC100646641	1.627679806	0.01792468	Up-regulated	alpha-glucosidase-like
LOC100650552	-0.28897875	0.018230907	Down-regulated	39S ribosomal protein L19
LOC100648599	0.49409982	0.018252605	Up-regulated	proton-coupled amino acid transporter 1
LOC100649167	0.675567391	0.018252605	Up-regulated	coiled-coil domain-containing protein 170
LOC100652118	0.278393346	0.018252605	Up-regulated	protein Wnt-1
LOC100645934	-0.234475757	0.018291612	Down-regulated	alpha-ketoglutarate dehydrogenase component 4
LOC100643604	-0.427659484	0.01842567	Down-regulated	uncharacterized LOC100643604
LOC100645850	0.244614939	0.018735142	Up-regulated	immunoglobulin domain-containing protein oig-4
LOC100646308	0.295026006	0.018735142	Up-regulated	protein bunched
LOC100646352	0.190575797	0.019352261	Up-regulated	phosphatidylinositol 4
LOC100643075	0.311176776	0.019357638	Up-regulated	WD repeat-containing protein 20
LOC100644957	0.146200043	0.019413492	Up-regulated	apoptotic chromatin condensation inducer in the nucleus
LOC100644959	0.177845553	0.019413492	Up-regulated	ubiquitin-conjugating enzyme E2-17 kDa
LOC100645776	-0.895059154	0.019413492	Down-regulated	pupal cuticle protein C1B
LOC100646107	0.472608322	0.019413492	Up-regulated	sodium-independent sulfate anion transporter
LOC100646695	-0.2751008	0.019413492	Down-regulated	V-type proton ATPase 21 kDa proteolipid subunit c"
LOC100646872	0.217246047	0.019413492	Up-regulated	palmitoyltransferase ZDHHC8
LOC100648281	0.664994152	0.019413492	Up-regulated	uncharacterized LOC100648281
LOC100648833	0.199316905	0.019413492	Up-regulated	antichymotrypsin-2
LOC105666586	-0.341007334	0.019413492	Down-regulated	U3 small nucleolar ribonucleoprotein protein IMP3
LOC110119243	-0.442735485	0.019413492	Down-regulated	uncharacterized LOC110119243
LOC100649369	0.533742798	0.019506455	Up-regulated	uncharacterized LOC100649369
LOC105666076	0.439813329	0.019758404	Up-regulated	uncharacterized LOC105666076
LOC100645792	0.383873479	0.019995813	Up-regulated	uncharacterized LOC100645792
LOC100647020	-0.248486981	0.019995813	Down-regulated	myb-like protein V
LOC100647553	0.170579822	0.02008434	Up-regulated	SWI/SNF complex subunit SMARCC2
LOC100651721	-0.402552127	0.020113649	Down-regulated	ionotropic receptor 21a
LOC100642360	0.564734577	0.020185248	Up-regulated	uncharacterized LOC100642360
LOC100650422	0.212808428	0.020185248	Up-regulated	actin-related protein 8
LOC100649347	-0.38394129	0.020192172	Down-regulated	protein jagunal
LOC100646021	-0.198444563	0.020217296	Down-regulated	periodic tryptophan protein 2 homolog
LOC100651847	0.563227361	0.020217296	Up-regulated	cytosolic carboxypeptidase 2
LOC100645030	-0.338966515	0.020291884	Down-regulated	ribonuclease P protein subunit p20
LOC100649762	-0.462834279	0.020354934	Down-regulated	ovarian-specific serine/threonine-protein kinase Lok
LOC110120070	-0.533371203	0.020354934	Down-regulated	leucine-rich repeat-containing protein 15-like
LOC100651452	-0.176494032	0.020423485	Down-regulated	cytochrome b-c1 complex subunit 7
LOC100643359	-0.236507745	0.02045601	Down-regulated	X-ray repair cross-complementing protein 6
LOC100644113	-0.221954723	0.02045601	Down-regulated	28S ribosomal protein S30
LOC100644743	0.395779801	0.02045601	Up-regulated	cytochrome P450 4c3 LOC100644743
LOC100646402	0.333404055	0.02045601	Up-regulated	voltage-gated potassium channel subunit beta-2
LOC100646433	-0.428203912	0.02045601	Down-regulated	ATPase WRNIP1
LOC100646571	0.195226406	0.02045601	Up-regulated	adenomatous polyposis coli protein
LOC100647095	-0.364777229	0.02045601	Down-regulated	protein MIX23
LOC100649719	-0.35479625	0.02045601	Down-regulated	glutamate--cysteine ligase regulatory subunit
LOC110119976	-0.488260432	0.02045601	Down-regulated	GPI mannosyltransferase 1
LOC100649136	0.201645014	0.02068858	Up-regulated	tectonin beta-propeller repeat-containing protein
LOC100649012	-0.149969376	0.020751081	Down-regulated	small glutamine-rich tetratricopeptide repeat-containing protein alpha
LOC100647597	-0.205185372	0.0207694	Down-regulated	uncharacterized LOC100647597
LOC100648241	-0.38882188	0.0207694	Down-regulated	HAUS augmin-like complex subunit 3
LOC100649266	0.214606528	0.0207694	Up-regulated	single-stranded DNA-binding protein 3
LOC105665673	0.285664391	0.0207694	Up-regulated	uncharacterized LOC105665673

LOC100644737	0.590066026	0.021391789	Up-regulated	facilitated trehalose transporter Tret1-like
LOC100649430	0.446360793	0.021397534	Up-regulated	E3 ubiquitin-protein ligase MIB1
LOC100649484	0.217485968	0.021397534	Up-regulated	moesin/ezrin/radixin homolog 1
LOC100645645	0.334201878	0.021483852	Up-regulated	FAD synthase
LOC100647259	0.516173866	0.021586474	Up-regulated	uncharacterized LOC100647259
LOC100642375	-0.326475041	0.021690553	Down-regulated	nuclear pore complex protein Nup93
LOC100642867	0.367360145	0.021690553	Up-regulated	serine/threonine-protein kinase OSR1
LOC100646058	-0.148793426	0.021690553	Down-regulated	T-complex protein 1 subunit alpha
LOC100648787	0.666732508	0.021690553	Up-regulated	dynein regulatory complex subunit 5
LOC100651211	0.130210533	0.021690553	Up-regulated	striatin
LOC105665927	-0.232737702	0.021690553	Down-regulated	MAPK regulated corepressor interacting protein 2
LOC100644463	0.937804569	0.021754383	Up-regulated	4-hydroxybutyrate coenzyme A transferase
LOC100644719	0.27720289	0.021754383	Up-regulated	DNA polymerase alpha subunit B
LOC100645389	0.346950667	0.021754383	Up-regulated	retinal homeobox protein Rx2
LOC100646324	0.35316588	0.021754383	Up-regulated	thyrotropin-releasing hormone-degrading ectoenzyme
LOC100648603	0.330334866	0.021754383	Up-regulated	putative phosphoenolpyruvate synthase
LOC100650655	-0.186842109	0.021754383	Down-regulated	mitochondrial chaperone BCS1
LOC100648864	-0.239748851	0.021769587	Down-regulated	conserved oligomeric Golgi complex subunit 6
LOC100643020	-2.695996208	0.022007983	Down-regulated	putative aminopeptidase-2
LOC100644620	-0.310559967	0.022007983	Down-regulated	translin
LOC100647247	0.222274999	0.022007983	Up-regulated	uncharacterized LOC100647247
LOC100647321	-0.224481472	0.022007983	Down-regulated	ectonucleoside triphosphate diphosphohydrolase 5
LOC100651343	0.316465092	0.022007983	Up-regulated	chaoptin
LOC105666834	0.978987817	0.022007983	Up-regulated	uncharacterized LOC105666834
LOC100643709	0.626922137	0.022035598	Up-regulated	neuropeptide CCHamide-1 receptor
LOC100645362	-0.186059179	0.022288583	Down-regulated	uncharacterized LOC100645362
LOC100647429	0.303326534	0.022288583	Up-regulated	uncharacterized LOC100647429
LOC100647741	-0.505198601	0.022288583	Down-regulated	uncharacterized LOC100647741
LOC100643056	0.331943717	0.022288944	Up-regulated	amyloid beta A4 precursor protein-binding family B member 1-interacting protein
LOC100650022	0.195756433	0.022288944	Up-regulated	probable ATP-dependent RNA helicase kurz
LOC100650358	-0.304869379	0.022288944	Down-regulated	1-acyl-sn-glycerol-3-phosphate acyltransferase alpha
LOC100650791	-0.350656722	0.022288944	Down-regulated	U6 snRNA-associated Sm-like protein LSm8
LOC105666363	0.896126266	0.022288944	Up-regulated	amine oxidase [flavin-containing]
LOC100644000	-0.468673755	0.022304957	Down-regulated	probable oligoribonuclease
LOC100645156	0.126484618	0.022304957	Up-regulated	uncharacterized LOC100645156
LOC100648630	-0.427258531	0.022304957	Down-regulated	cytochrome c oxidase assembly factor 5
LOC100649562	-0.535735222	0.022304957	Down-regulated	mismatch repair endonuclease PMS2
LOC100651957	0.691694308	0.022304957	Up-regulated	uncharacterized LOC100651957
LOC100646979	-0.332728251	0.022391917	Down-regulated	viral IAP-associated factor homolog
LOC100649617	0.443269796	0.0224294	Up-regulated	glucose dehydrogenase [FAD
LOC100651128	0.606774564	0.0224294	Up-regulated	uncharacterized LOC100651128
LOC100647243	0.410714197	0.022566698	Up-regulated	tyrosine-protein kinase Btk29A
LOC100647965	0.63855623	0.022566698	Up-regulated	monocarboxylate transporter 12-B
LOC105666149	0.51613497	0.022667149	Up-regulated	histone demethylase UTY
LOC100651350	0.550119298	0.022723143	Up-regulated	uncharacterized LOC100651350
LOC100642522	0.209523564	0.022733848	Up-regulated	neurexin-4
LOC105666259	-0.256980845	0.022733848	Down-regulated	zinc finger protein 706
LOC100642849	-0.363880828	0.022736553	Down-regulated	Werner Syndrome-like exonuclease
LOC100642975	-0.282860623	0.022736553	Down-regulated	bridging integrator 3
LOC100643135	-0.162185504	0.022736553	Down-regulated	protein l(2)37Cc
LOC100646943	0.197288616	0.022736553	Up-regulated	fatty-acid amide hydrolase 2-A
LOC100648705	-0.43255907	0.022736553	Down-regulated	tubulointerstitial nephritis antigen-like
LOC100651776	-0.345865304	0.022736553	Down-regulated	uncharacterized LOC100651776
LOC100651846	-0.280504375	0.022736553	Down-regulated	RAD50-interacting protein 1
LOC100642201	-0.360149904	0.022815589	Down-regulated	glycerol 2-dehydrogenase (NADP(+))
LOC100651534	0.219813151	0.022815589	Up-regulated	uncharacterized LOC100651534
LOC100647856	0.559506687	0.02286176	Up-regulated	uncharacterized LOC100647856
LOC110119584	0.552548219	0.022881532	Up-regulated	uncharacterized LOC110119584
LOC105666886	1.100838786	0.022913633	Up-regulated	facilitated trehalose transporter Tret1-like
LOC100649172	0.123152664	0.022983975	Up-regulated	replication protein A 70 kDa DNA-binding subunit
LOC100651127	0.189296093	0.023436157	Up-regulated	broad-complex core protein
LOC100643778	0.147899046	0.023470883	Up-regulated	RCC1 and BTB domain-containing protein 1
LOC100652200	-0.330412294	0.023480873	Down-regulated	39S ribosomal protein L14
LOC100649399	-0.248412789	0.02348791	Down-regulated	T-cell activation inhibitor
LOC100645193	0.178954955	0.023601492	Up-regulated	DNA polymerase iota
LOC100647232	-0.610330986	0.023660039	Down-regulated	uncharacterized LOC100647232
LOC100650774	-0.33109048	0.023746564	Down-regulated	surfeit locus protein 1
LOC100646329	-0.384041152	0.023903685	Down-regulated	peptidyl-prolyl cis-trans isomerase NIMA-interacting 4
LOC100651671	0.503033692	0.023903685	Up-regulated	putative inorganic phosphate cotransporter
LOC100650337	-0.195657906	0.024117439	Down-regulated	Golgi apparatus protein 1
LOC100645886	-0.33705658	0.024604904	Down-regulated	39S ribosomal protein L20
LOC100650317	-0.853765782	0.024604904	Down-regulated	tubulin alpha-2/alpha-4 chain
LOC100643053	0.326736287	0.024634551	Up-regulated	flotillin-2
LOC100645667	-0.216502104	0.024789758	Down-regulated	haloacid dehalogenase-like hydrolase domain-containing 5
LOC105666531	-0.433428857	0.024789758	Down-regulated	COMM domain-containing protein 2
LOC100651530	-0.749686937	0.024894037	Down-regulated	uncharacterized LOC100651530
LOC105665661	-0.675755788	0.024894037	Down-regulated	cylicin-1
LOC100645898	0.916435142	0.024909292	Up-regulated	T-box transcription factor TBX10
LOC100651656	-0.281491693	0.024909292	Down-regulated	myosin-2 essential light chain
LOC100652263	0.422546666	0.024909292	Up-regulated	G-protein coupled receptor dmsr-1
LOC100650339	0.393447861	0.025025052	Up-regulated	chloride channel protein 2

LOC100643494	-0.476106938	0.025031563	Down-regulated	ubiquitin-associated domain-containing protein 1
LOC100650619	-0.285180148	0.025148572	Down-regulated	DNA-directed RNA polymerases I
LOC100643825	0.290482408	0.025323469	Up-regulated	uncharacterized LOC100643825
LOC100644001	-0.414927645	0.025323469	Down-regulated	caltractin
LOC100644198	-0.334408083	0.025323469	Down-regulated	NADH dehydrogenase [ubiquinone] 1 subunit C2
LOC100644972	0.405474047	0.025654358	Up-regulated	glycine-rich protein DOT1
LOC100645281	-0.188282919	0.025654358	Down-regulated	NADH dehydrogenase [ubiquinone] iron-sulfur protein 3
LOC100645841	-1.204006923	0.025654358	Down-regulated	N-lysine methyltransferase SMYD2
LOC100645511	-0.399334069	0.02604049	Down-regulated	inositol-3-phosphate synthase 1-B
LOC105666154	-0.366132277	0.026077828	Down-regulated	WD repeat-containing protein WRAP73
LOC100647265	-0.43407028	0.026112378	Down-regulated	globin
LOC100649253	0.680402259	0.026112378	Up-regulated	collagen alpha-1(I) chain
LOC100643190	-0.233326029	0.026196272	Down-regulated	ATP synthase subunit O
LOC100646864	-0.305052195	0.026196272	Down-regulated	RNA polymerase II subunit A C-terminal domain phosphatase SSU72
LOC100648965	0.474836437	0.026196272	Up-regulated	NPC intracellular cholesterol transporter 1
LOC100648466	-0.170611506	0.026257771	Down-regulated	geranylgeranyl transferase type-1 subunit beta
LOC100644208	0.484920598	0.02652541	Up-regulated	uncharacterized LOC100644208
LOC100648047	-0.488073686	0.026557891	Down-regulated	ribonuclease H2 subunit C
LOC100648688	0.396242417	0.026569682	Up-regulated	aromatic-L-amino-acid decarboxylase
LOC100650683	-0.191330656	0.026756912	Down-regulated	nuclear FMR1 interacting protein 1
LOC100644618	1.279478224	0.026766035	Up-regulated	facilitated trehalose transporter Tret1
LOC100644777	-0.285611022	0.026833148	Down-regulated	breast cancer metastasis-suppressor 1-like protein
LOC100648928	-0.195458387	0.026833148	Down-regulated	ATP synthase subunit b
LOC100650884	0.436218077	0.026897667	Up-regulated	serine/threonine-protein phosphatase 4 regulatory subunit 1
LOC105666201	0.258944293	0.026897667	Up-regulated	protein Spindly
LOC100644746	0.617205103	0.026942086	Up-regulated	outer dynein arm-docking complex subunit 4
LOC100644432	0.529610911	0.02702864	Up-regulated	uncharacterized LOC100644432
LOC100642518	-0.342956027	0.027080713	Down-regulated	DNA polymerase interacting tetratricopeptide repeat-containing
LOC100646650	-0.291252325	0.027080713	Down-regulated	uncharacterized LOC100646650
LOC100647713	-0.386753303	0.027080713	Down-regulated	UPF0605 protein CG18335
LOC105666049	0.219236867	0.02735615	Up-regulated	cleavage stimulation factor subunit 2
LOC100644153	0.41808511	0.027374105	Up-regulated	mitochondrial carrier protein Rim2
LOC105666300	-0.495330586	0.027389808	Down-regulated	COA8 family protein CG14806
LOC100644823	0.169505459	0.027412338	Up-regulated	V-type proton ATPase subunit B
LOC100646289	0.463707118	0.027412338	Up-regulated	protein scalloped
LOC100648726	-0.187664959	0.027412338	Down-regulated	ubiquitin carboxyl-terminal hydrolase MINDY-3 homolog
LOC100642590	-0.255175707	0.027629511	Down-regulated	COMM domain-containing protein 8
LOC100645460	-0.400806202	0.027629511	Down-regulated	uncharacterized LOC100645460
LOC100644036	0.71403791	0.027636706	Up-regulated	protein Skeletor
LOC100647470	0.479111867	0.027636706	Up-regulated	zwei Ig domain protein zig-8
LOC100652008	-0.311039797	0.027636706	Down-regulated	uncharacterized LOC100652008
LOC100646700	0.294145998	0.027722942	Up-regulated	SEC14-like protein 2
LOC100647391	-0.131873575	0.027742523	Down-regulated	NECAP-like protein CG9132
LOC100644135	0.137299	0.027829841	Up-regulated	MOG interacting and ectopic P-granules protein 1
LOC105666911	-0.524332089	0.027829841	Down-regulated	palmitoyltransferase ZDHHC22
LOC100644395	-0.231664917	0.027874325	Down-regulated	uncharacterized LOC100644395
LOC100648482	-1.72904511	0.028097047	Down-regulated	antichymotrypsin-2
LOC100643965	-0.398823394	0.02836837	Down-regulated	39S ribosomal protein L13
LOC100644738	-0.528343005	0.028707292	Down-regulated	glyoxylate/hydroxypyruvate reductase A-like
LOC100646108	-0.207277472	0.028903153	Down-regulated	polyadenylate-binding protein 2-B
LOC110120267	-1.087464464	0.028968595	Down-regulated	uncharacterized LOC110120267
LOC100645499	-0.267381597	0.029190633	Down-regulated	palmitoyl-protein thioesterase 1
LOC100642627	0.23703816	0.029771113	Up-regulated	rho guanine nucleotide exchange factor 7
LOC100646159	-0.28599992	0.029771113	Down-regulated	RING finger protein 37
LOC100647138	-0.187897949	0.029771113	Down-regulated	39S ribosomal protein L15
LOC100648371	-0.456754247	0.029771113	Down-regulated	pre-mRNA-splicing factor CWC22 homolog
LOC100650616	0.374414024	0.029771113	Up-regulated	dedicator of cytokinesis protein 3
LOC100652304	-0.30086698	0.029843458	Down-regulated	NADH dehydrogenase [ubiquinone] iron-sulfur protein 6
LOC100650685	0.487049346	0.029868576	Up-regulated	rho guanine nucleotide exchange factor 10
LOC100643592	-0.306835532	0.03027074	Down-regulated	uncharacterized protein C7orf50 homolog
LOC100644492	0.319795532	0.03027074	Up-regulated	neurogenic protein big brain
LOC100643460	-0.91382105	0.030416199	Down-regulated	UNC93-like protein
LOC100648344	-0.38194588	0.030452435	Down-regulated	crossover junction endonuclease EME1
LOC100645554	0.568118557	0.030474966	Up-regulated	heparan sulfate glucosamine 3-O-sulfotransferase 5
LOC100650332	0.445152859	0.030669383	Up-regulated	general odorant-binding protein 69a
LOC100651632	-0.332974715	0.030669383	Down-regulated	protein kish-A
LOC100643200	0.501569131	0.031030467	Up-regulated	growth hormone secretagogue receptor type 1
LOC100645505	-0.169274796	0.031030467	Down-regulated	protein phosphatase 1 regulatory subunit 14B
LOC100652007	0.30372465	0.031030467	Up-regulated	carbohydrate sulfotransferase 11
LOC100650960	0.319294014	0.031051384	Up-regulated	fat-like cadherin-related tumor suppressor homolog
LOC105666944	0.257938086	0.031051384	Up-regulated	nucleoporin Nup35
LOC100642258	-0.305871153	0.031139543	Down-regulated	uncharacterized protein F21D5.5
LOC100643279	-0.287027356	0.031182237	Down-regulated	phosphatidylinositol-glycan biosynthesis class F protein
LOC100645908	-0.284004296	0.031182237	Down-regulated	elongation of very long chain fatty acids protein 4
LOC105665772	-0.342756521	0.031182237	Down-regulated	39S ribosomal protein L17
LOC100645051	0.375259949	0.031276608	Up-regulated	protein slit
LOC100646450	-0.294310965	0.031377783	Down-regulated	intraflagellar transport protein 81 homolog
LOC105666139	-1.190923221	0.031377783	Down-regulated	15-hydroxyprostaglandin dehydrogenase [NAD(+)]
LOC100645648	0.490526341	0.031491575	Up-regulated	laccase-1
LOC100645746	0.177742021	0.031491575	Up-regulated	kinase suppressor of Ras 2
LOC100646371	-0.362736882	0.031491575	Down-regulated	probable arginine--tRNA ligase

LOC100647665	-0.226880705	0.031491575	Down-regulated	probable 39S ribosomal protein L49
LOC100649007	0.640957936	0.031491575	Up-regulated	cholesterol 7-desaturase nvd
LOC100647000	0.205013124	0.031491746	Up-regulated	dedicator of cytokinesis protein 1
LOC100652179	-0.172678415	0.031670964	Down-regulated	WD and tetratricopeptide repeats protein 1
LOC100645345	0.38615146	0.031698924	Up-regulated	cationic amino acid transporter 4
LOC100643742	0.418533051	0.031727588	Up-regulated	CAD protein
LOC100651993	-0.374292856	0.031814972	Down-regulated	39S ribosomal protein L54
LOC100648976	-0.24441868	0.031939071	Down-regulated	protein zer-1 homolog
LOC100646873	-1.210171159	0.032349933	Down-regulated	uncharacterized LOC100646873
LOC100647705	0.380938656	0.032349933	Up-regulated	uncharacterized LOC100647705
LOC100648433	0.883270015	0.032349933	Up-regulated	uncharacterized aarF domain-containing protein kinase 2
LOC100650980	-0.481154671	0.032349933	Down-regulated	protein POLR1D
LOC100644473	-0.159562383	0.032378516	Down-regulated	neutral alpha-glucosidase AB
LOC100645155	0.292785568	0.032378516	Up-regulated	U4/U6 small nuclear ribonucleoprotein Prp4
LOC100648330	0.156542637	0.032378516	Up-regulated	pumilio homolog 2
LOC100649278	-0.875944407	0.032378516	Down-regulated	hemocyte protein-glutamine gamma-glutamyltransferase
LOC100649416	-0.290454265	0.032378516	Down-regulated	zinc finger HIT domain-containing protein 3
LOC100649952	0.218612026	0.032378516	Up-regulated	regulator of chromosome condensation
LOC100648975	-0.373521402	0.032799028	Down-regulated	protein immune deficiency
LOC100650646	0.540353407	0.032966853	Up-regulated	SPARC-related modular calcium-binding protein 2
LOC100646345	0.57218222	0.033016051	Up-regulated	leucine-rich repeat-containing protein 24
LOC100649398	-0.22015191	0.033063708	Down-regulated	NADH dehydrogenase [ubiquinone] 1 beta subcomplex subunit 3
LOC100651598	-0.282515406	0.033221602	Down-regulated	protein LTV1 homolog
LOC100643706	0.266515329	0.033277182	Up-regulated	pre-mRNA-splicing factor RBM22
LOC100644162	0.392520146	0.033301767	Up-regulated	sodium/calcium exchanger regulatory protein 1
LOC100646705	0.419559393	0.033301767	Up-regulated	protein Wnt-7b
LOC100651737	-0.358227841	0.033557971	Down-regulated	28S ribosomal protein S18c
LOC100648412	0.304881896	0.033697915	Up-regulated	putative GPI-anchored protein pfl2
LOC100644876	0.386393349	0.033749134	Up-regulated	LIM domain only protein 3
LOC100642504	-0.346836919	0.03408185	Down-regulated	trichohyalin
LOC100643811	0.307423037	0.03408185	Up-regulated	ADP-ribosylhydrolase ARH3
LOC100647483	0.366888745	0.03408185	Up-regulated	A-kinase anchor protein 200
LOC105665631	-0.278831545	0.03408185	Down-regulated	iron-sulfur protein NUBPL
LOC100646000	-0.282837912	0.034105294	Down-regulated	gem-associated protein 5
LOC100650801	-0.232084334	0.034105294	Down-regulated	protein sarah
LOC110119545	0.413315748	0.034105294	Up-regulated	zinc finger protein 596-like
LOC100649531	-0.397663837	0.034189812	Down-regulated	dolichyl-diphosphooligosaccharide--protein glycosyltransferase subunit 4
LOC100648774	-0.222599311	0.034365235	Down-regulated	aspartate aminotransferase
LOC100651159	-0.267916818	0.034365235	Down-regulated	U6 snRNA-associated Sm-like protein LSm5
LOC105666028	0.43884515	0.034365235	Up-regulated	protein suppressor of hairy wing
LOC110120352	-0.516328653	0.034365235	Down-regulated	uncharacterized LOC110120352
LOC100650895	-0.230746076	0.034599861	Down-regulated	reactive oxygen species modulator 1
LOC100644304	-0.169854194	0.034627909	Down-regulated	protein germ cell-less
LOC100648636	-0.389191276	0.034627909	Down-regulated	2-amino-3-ketobutyrate coenzyme A ligase
LOC100651266	-0.198649581	0.034627909	Down-regulated	S-adenosylmethionine mitochondrial carrier protein
LOC100646627	-0.23481888	0.034645416	Down-regulated	biogenesis of lysosome-related organelles complex 1 subunit 5
LOC100650725	-0.405131107	0.034645416	Down-regulated	regulator of telomere elongation helicase 1 homolog
LOC100651627	-0.280363911	0.034645416	Down-regulated	PHD finger-like domain-containing protein 5A
LOC100644846	0.409173502	0.034733646	Up-regulated	sodium- and chloride-dependent glycine transporter 1
LOC100648911	-0.168417176	0.034818834	Down-regulated	COP9 signalosome complex subunit 5
LOC100650228	-0.353410845	0.034818834	Down-regulated	uncharacterized LOC100650228
LOC100643214	0.509736256	0.035343738	Up-regulated	4-coumarate--CoA ligase 1
LOC105667070	0.721798829	0.035343738	Up-regulated	uncharacterized LOC105667070
LOC100642282	-0.189779622	0.035556943	Down-regulated	transmembrane protein 256 homolog
LOC100642821	-0.219553322	0.035556943	Down-regulated	ATP synthase subunit e
LOC100646225	-0.228548297	0.035556943	Down-regulated	telomere length and silencing protein 1 homolog
LOC100647463	0.273976595	0.035556943	Up-regulated	zinc finger protein 384
LOC100652075	-0.226246644	0.035556943	Down-regulated	3-hydroxyacyl-CoA dehydrogenase type-2
LOC100645625	-0.271714864	0.035748387	Down-regulated	tax1-binding protein 3 homolog
LOC100647693	0.325491351	0.035748387	Up-regulated	head-specific guanylate cyclase
LOC100649653	0.477561478	0.0357655	Up-regulated	valacyclovir hydrolase
LOC100644236	-0.261554092	0.036067694	Down-regulated	NADH dehydrogenase [ubiquinone] 1 alpha subcomplex subunit 12
LOC100645876	0.453222546	0.036117769	Up-regulated	uncharacterized LOC100645876
LOC100649065	-0.413851447	0.036117769	Down-regulated	DNA repair protein XRCC3
LOC100651775	-0.310102927	0.036117769	Down-regulated	rRNA methyltransferase 3
LOC100652146	-0.423963999	0.036196408	Down-regulated	uncharacterized LOC100652146
LOC100644958	0.30800896	0.036250033	Up-regulated	sialin
LOC100646556	0.220050171	0.036920697	Up-regulated	rac GTPase-activating protein 1
LOC100644547	0.147361132	0.03738713	Up-regulated	rho GTPase-activating protein 190
LOC100645241	0.389910256	0.03738713	Up-regulated	estradiol 17-beta-dehydrogenase 2
LOC100643594	0.472716872	0.037415705	Up-regulated	uncharacterized LOC100643594
LOC100643044	-0.186333551	0.03742622	Down-regulated	probable serine/threonine-protein kinase roco9
LOC100643401	0.226567639	0.037430282	Up-regulated	WW domain-binding protein 4
LOC100648815	0.252769387	0.037430282	Up-regulated	TRPL translocation defect protein 14
LOC100646460	-0.269251809	0.03792396	Down-regulated	protein lin-37 homolog
LOC100650206	0.209702584	0.038049319	Up-regulated	GSK-3-binding protein
LOC105665678	-0.343771259	0.038138111	Down-regulated	complex I assembly factor ACAD9
LOC105666791	0.269741401	0.038138111	Up-regulated	uncharacterized LOC105666791
LOC100647106	0.17705188	0.038256551	Up-regulated	CCHC-type zinc finger nucleic acid binding protein
LOC100650919	-0.160041082	0.038256551	Down-regulated	peroxiredoxin-5
LOC100650983	-0.245337667	0.038256551	Down-regulated	CUE domain-containing protein 1

LOC100643557	0.547134596	0.038331949	Up-regulated	facilitated trehalose transporter Tret1
LOC100647833	0.730659793	0.038530462	Up-regulated	protein Wnt-6
LOC100650119	-0.274744532	0.039008441	Down-regulated	protein cueball
LOC100647687	-0.30354037	0.039130461	Down-regulated	1-phosphatidylinositol 4
LOC100652258	-0.415380228	0.039130461	Down-regulated	aldo-keto reductase family 1 member B1
LOC100646414	0.292778557	0.039192475	Up-regulated	beta-1
LOC100649115	0.233876483	0.039192475	Up-regulated	putative uncharacterized protein DDB_G0286901
LOC100646232	0.424714336	0.039240779	Up-regulated	putative uncharacterized protein DDB_G0271606
LOC110119981	-0.305004975	0.039240779	Down-regulated	uncharacterized LOC110119981
LOC100642752	-0.241146714	0.039260737	Down-regulated	uncharacterized LOC100642752
LOC100651201	0.159802903	0.03971656	Up-regulated	tafazzin
LOC100651296	0.16880973	0.03971656	Up-regulated	uncharacterized LOC100651296
LOC100650520	0.155921849	0.040263582	Up-regulated	histone H4 transcription factor
LOC100644768	-0.235454015	0.040367505	Down-regulated	calcineurin B homologous protein 1
LOC100651261	0.331309599	0.040367505	Up-regulated	endothelin-converting enzyme homolog
LOC100648317	-0.339991992	0.040413756	Down-regulated	deoxynucleoside kinase
LOC100650735	0.145198088	0.040413756	Up-regulated	tyrosine-protein phosphatase non-receptor type 23
LOC100644515	-0.247532195	0.040567586	Down-regulated	ras-like GTP-binding protein RhoL
LOC100646467	0.400427883	0.040567586	Up-regulated	uncharacterized LOC100646467
LOC100647879	0.67889032	0.040567586	Up-regulated	cyclic nucleotide-gated cation channel alpha-3
LOC100648472	-1.040422849	0.040567586	Down-regulated	endocuticle structural glycoprotein SgAbd-1
LOC100650114	-0.162309413	0.040567586	Down-regulated	vacuolar protein sorting-associated protein 35
LOC100650820	-0.329322501	0.040567586	Down-regulated	lambda-crystallin homolog
LOC100651841	-0.19673114	0.04064153	Down-regulated	vam6/Vps39-like protein
LOC100643089	-0.243262792	0.040681095	Down-regulated	probable cytosolic oligopeptidase A
LOC100649497	-0.305263801	0.040808145	Down-regulated	complex I intermediate-associated protein 30
LOC100645285	-0.198605939	0.041006295	Down-regulated	DNA polymerase delta catalytic subunit
LOC100643671	0.565136005	0.041025064	Up-regulated	FMRFamide-related peptides
LOC100646140	-0.378346305	0.041025064	Down-regulated	39S ribosomal protein L9
LOC100645579	-0.490059807	0.041051483	Down-regulated	stimulator of interferon genes protein homolog
LOC100642451	-0.284380303	0.041257977	Down-regulated	adrenodoxin-like protein 2
LOC100644943	0.100970747	0.041257977	Up-regulated	interleukin enhancer-binding factor 2
LOC100643817	0.421252705	0.041459201	Up-regulated	dynactin subunit 5
LOC100642466	-0.442637335	0.04157102	Down-regulated	transmembrane protein 17
LOC100643559	0.205014259	0.04157102	Up-regulated	inositol 1
LOC100643808	-0.357260916	0.04157102	Down-regulated	acyl-coenzyme A thioesterase 13
LOC100644467	-0.291009468	0.04157102	Down-regulated	WD repeat-containing protein 74
LOC100644801	-0.175329315	0.04157102	Down-regulated	TBC1 domain family member 23
LOC100651713	0.606311072	0.04157102	Up-regulated	venom acid phosphatase Acph-1
LOC100642260	0.186756153	0.041669153	Up-regulated	calcium homeostasis endoplasmic reticulum protein
LOC100644376	-0.179986877	0.041669153	Down-regulated	cystathionine beta-synthase
LOC100646123	-0.266787891	0.041669153	Down-regulated	transmembrane protein 185A
LOC100647551	-0.166243642	0.041669153	Down-regulated	thioredoxin domain-containing protein 15
LOC100650307	0.3848926	0.041669153	Up-regulated	uncharacterized LOC100650307
LOC105666163	-0.370084357	0.041669153	Down-regulated	zinc transporter 2
LOC105666264	-0.406800217	0.041669153	Down-regulated	28 kDa ribonucleoprotein
LOC100644389	0.372841431	0.041777131	Up-regulated	monocarboxylate transporter 2
LOC105667104	0.271994763	0.041908245	Up-regulated	protein lethal(2)essential for life
LOC100646035	0.196162167	0.042129587	Up-regulated	RNA-binding protein 26
LOC100644671	-0.990558835	0.04225666	Down-regulated	uncharacterized LOC100644671
LOC100646584	0.227899464	0.04225666	Up-regulated	phosphatidylcholine:ceramide cholinephosphotransferase 2
LOC100646646	-0.166987047	0.04225666	Down-regulated	modular serine protease
LOC100648982	0.802831507	0.04225666	Up-regulated	uncharacterized LOC100648982
LOC100648034	-0.212301637	0.042301111	Down-regulated	F-box only protein 9
LOC100651735	-0.232509402	0.042587636	Down-regulated	
LOC100652088	-0.404268324	0.042732956	Down-regulated	sodium-coupled monocarboxylate transporter 1
LOC100645857	-0.258604449	0.042768003	Down-regulated	centromere-associated protein E
LOC100649815	0.366071839	0.042768003	Up-regulated	tyrosine-protein kinase Drl
LOC100644354	-0.249551376	0.042902743	Down-regulated	uncharacterized LOC100644354
LOC100645228	0.3695051	0.042902743	Up-regulated	uncharacterized LOC100645228
LOC100647411	-0.333150295	0.042902743	Down-regulated	60S ribosomal protein L11
LOC105667074	0.302312959	0.042902743	Up-regulated	glutathione S-transferase 1
LOC100649409	-0.322089605	0.043693041	Down-regulated	kynurenine/alpha-aminoadipate aminotransferase
LOC100648022	0.339726617	0.044086293	Up-regulated	N-acetylgalactosamine kinase
LOC100650911	-0.299415409	0.044086293	Down-regulated	uncharacterized LOC100650911
LOC100646742	-0.314592129	0.044143305	Down-regulated	pyrimidodiazepine synthase
LOC100648411	-0.132076511	0.044143305	Down-regulated	histone acetyltransferase KAT2A
LOC100649391	-0.302846923	0.044143305	Down-regulated	mitochondrial glycine transporter
LOC100651398	-0.531045148	0.044143305	Down-regulated	leucine-rich repeat-containing protein 28
LOC105666200	-0.219109811	0.044143305	Down-regulated	transmembrane protein 222
LOC100651423	-0.18445133	0.044231647	Down-regulated	cystinosin homolog
LOC105665646	-0.260534879	0.044231647	Down-regulated	GPI mannosyltransferase 3
LOC100649294	0.208988767	0.044240757	Up-regulated	transmembrane 7 superfamily member 3
LOC100645172	-0.161283011	0.044393776	Down-regulated	probable phosphoglycerate kinase
LOC100646037	0.322711017	0.044401485	Up-regulated	F-box/LRR-repeat protein 2
LOC100651867	-0.367606119	0.044401485	Down-regulated	signal recognition particle 9 kDa protein
LOC100644184	-0.349300843	0.044571871	Down-regulated	mitochondrial assembly of ribosomal large subunit protein 1
LOC100647007	-0.356668257	0.044591219	Down-regulated	uncharacterized LOC100647007
LOC100642994	-0.200946782	0.044889888	Down-regulated	palmitoyltransferase ZDHHC6
LOC100644663	0.318642939	0.044889888	Up-regulated	putative uncharacterized protein DDB_G0271606
LOC100645495	-0.431486832	0.044889888	Down-regulated	bolA-like protein DDB_G0274169

LOC100645831	-0.711571111	0.044889888	Down-regulated	elongation of very long chain fatty acids protein 1
LOC100647821	0.331964346	0.044889888	Up-regulated	tubulin monoglutamylase TTLL4
LOC100649081	0.267582758	0.044889888	Up-regulated	DNA polymerase subunit gamma-1
LOC100650369	-0.458014648	0.044889888	Down-regulated	uncharacterized LOC100650369
LOC100650964	-0.433430105	0.044889888	Down-regulated	uncharacterized LOC100650964
LOC100651070	-0.325081513	0.044889888	Down-regulated	uncharacterized LOC100651070
LOC100651224	0.747681614	0.044889888	Up-regulated	uncharacterized LOC100651224
LOC100643351	-0.22452459	0.044961856	Down-regulated	uncharacterized protein C1orf50 homolog
LOC100645004	0.210314359	0.044985662	Up-regulated	mitochondrial import receptor subunit TOM70
LOC100650613	-0.174754915	0.045013356	Down-regulated	protein real-time
LOC100646127	0.439348322	0.045054434	Up-regulated	odorant receptor 47a
LOC100648843	-0.208940175	0.045279029	Down-regulated	digestive cysteine proteinase 1
LOC100646400	0.729518514	0.045522099	Up-regulated	ionotropic receptor 25a
LOC105666394	-0.413090803	0.045573787	Down-regulated	peptidoglycan recognition protein 3
LOC100649835	0.462779286	0.045689581	Up-regulated	hemicentin-1
LOC100643216	-0.408097158	0.045886834	Down-regulated	mitochondrial import receptor subunit TOM7 homolog
LOC100643485	-0.735609659	0.045930876	Down-regulated	zinc finger protein 668
LOC100649018	-0.44080627	0.045930876	Down-regulated	transmembrane protein 70 homolog
LOC100650597	0.467126348	0.045930876	Up-regulated	pyrokinin-1 receptor
LOC100643634	0.571697743	0.045986001	Up-regulated	DDB1- and CUL4-associated factor 11-like
LOC100646974	0.227325002	0.046034394	Up-regulated	RNA-binding protein 25
LOC100643055	-0.174028971	0.046331478	Down-regulated	proteasome adapter and scaffold protein ECM29
LOC100644145	-0.47742558	0.046331478	Down-regulated	L-threonine 3-dehydrogenase
LOC100644704	-0.340598081	0.046331478	Down-regulated	ubiquitin-fold modifier-conjugating enzyme 1
LOC100649996	-0.430771454	0.046331478	Down-regulated	uncharacterized LOC100649996
VSP	-0.542651846	0.046331478	Down-regulated	venom serine protease
LOC100649121	-0.127754637	0.046723147	Down-regulated	sorting nexin-2
LOC100648247	-0.21287954	0.046766321	Down-regulated	DNA polymerase epsilon subunit 2
LOC100652192	0.42480241	0.04684539	Up-regulated	N-glycosylase/DNA lyase
LOC100643927	0.311119425	0.046938132	Up-regulated	uncharacterized LOC100643927
LOC100648531	-0.426720015	0.047121447	Down-regulated	X-linked retinitis pigmentosa GTPase regulator
LOC100645167	0.296711127	0.047365475	Up-regulated	protein unc-13 homolog 4B
LOC100646930	-0.445216096	0.047365475	Down-regulated	uncharacterized LOC100646930
LOC100647182	-0.260733429	0.047365475	Down-regulated	cytoplasmic tRNA 2-thiolation protein 2
LOC105666060	0.585896706	0.047365475	Up-regulated	uncharacterized LOC105666060
LOC100647286	0.209503419	0.047417395	Up-regulated	uncharacterized LOC100647286
LOC100646408	0.376374829	0.04741949	Up-regulated	protein timeless homolog
LOC100651885	0.215007304	0.047474768	Up-regulated	mediator of RNA polymerase II transcription subunit 12
LOC100650805	0.242078402	0.047491601	Up-regulated	ankyrin repeat domain-containing protein 17
LOC100646113	-0.239074752	0.047548911	Down-regulated	prefoldin subunit 5
LOC100646374	0.348444385	0.047548911	Up-regulated	battenin
LOC100646802	-0.128080392	0.047548911	Down-regulated	lipoma-preferred partner homolog
LOC100647520	0.581536564	0.047548911	Up-regulated	uncharacterized LOC100647520
LOC105666659	0.327265451	0.047548911	Up-regulated	ejaculatory bulb-specific protein 3-like
LOC100646681	-0.300213462	0.047827123	Down-regulated	uncharacterized LOC100646681
LOC100642516	0.384362646	0.047837049	Up-regulated	uncharacterized LOC100642516
LOC100648188	-0.158171311	0.047837049	Down-regulated	uncharacterized LOC100648188
LOC100643814	-0.186977932	0.047904044	Down-regulated	drebrin-like protein
LOC100649998	0.139971396	0.047904044	Up-regulated	mediator of RNA polymerase II transcription subunit 23
LOC100645524	-0.578464685	0.048289986	Down-regulated	heat shock protein 83
LOC100647301	0.300461313	0.048289986	Up-regulated	acetylcholine receptor subunit alpha-L1
LOC100645093	-0.291619045	0.048378667	Down-regulated	arginyl-tRNA--protein transferase 1
LOC100650174	0.225172209	0.048378667	Up-regulated	band 7 protein AGAP004871
LOC100647041	-0.813001801	0.048412256	Down-regulated	probable cytochrome P450 6a14 LOC100647041
LOC100650729	0.114393231	0.048426263	Up-regulated	PHD finger protein 14
LOC100651089	0.327828145	0.048426263	Up-regulated	uncharacterized LOC100651089
LOC100652164	-0.373804589	0.048426263	Down-regulated	probable 28S ribosomal protein S25
LOC105666872	0.310847397	0.048426263	Up-regulated	centrosomal protein of 135 kDa
LOC100646011	0.623879554	0.048523398	Up-regulated	dynein axonemal heavy chain 2
LOC100643795	-0.157139936	0.04863353	Down-regulated	nucleolar complex protein 4 homolog B
LOC100646583	-0.313314342	0.04863353	Down-regulated	multidrug resistance-associated protein 1
LOC100648113	0.186778407	0.04863353	Up-regulated	inositol hexakisphosphate and diphosphoinositol-pentakisphosphate kinase 2
LOC100646694	-0.132157682	0.048666453	Down-regulated	isocitrate dehydrogenase [NAD] subunit beta
LOC100648825	0.254947682	0.048781126	Up-regulated	protein KIAA0100
LOC100646432	0.155137208	0.048912864	Up-regulated	copper-transporting ATPase 1
LOC105666305	-0.853400703	0.048912864	Down-regulated	leucine-rich repeat-containing protein 23
LOC100642962	-0.239497793	0.049132059	Down-regulated	DENN domain-containing protein 10
LOC100648987	0.289038139	0.049166714	Up-regulated	acetylcholine receptor subunit alpha-like
LOC105667120	0.281984278	0.049166714	Up-regulated	Bardet-Biedl syndrome 1 protein homolog
LOC100642332	0.191048118	0.049183363	Up-regulated	casein kinase I
LOC100646837	-0.350801631	0.049183363	Down-regulated	malonyl-CoA decarboxylase
LOC100642720	0.303634344	0.049387751	Up-regulated	basic salivary proline-rich protein 2
LOC100650852	-0.209021669	0.049776368	Down-regulated	inositol polyphosphate 1-phosphatase
LOC100645243	0.230915542	0.049805774	Up-regulated	putative 28S ribosomal protein S5
LOC100646109	-0.181935885	0.049805774	Down-regulated	ubiquitin carboxyl-terminal hydrolase 14
LOC100646568	-0.267769949	0.049805774	Down-regulated	DNA-directed RNA polymerase III subunit RPC7-like
LOC100648994	-0.336628502	0.049805774	Down-regulated	gamma-secretase subunit pen-2
LOC100649966	-0.518378731	0.049805774	Down-regulated	Sjoegren syndrome nuclear autoantigen 1 homolog
LOC100650460	0.460624584	0.049805774	Up-regulated	transferrin
LOC100651813	-0.318656219	0.049805774	Down-regulated	solute carrier family 25 member 35
LOC100645479	0.545912664	0.04998147	Up-regulated	ADAMTS-like protein 3

ACUTE CLOTHIANIDIN

Gene ID	log2Fold Change	Adjusted P-value (FDR)	Expression pattern	Gene annotation
LOC100644102	0.846336545	1.29E-14	Up-regulated	uncharacterized LOC100644102
LOC100643615	1.45582978	5.43E-14	Up-regulated	glycerol-3-phosphate dehydrogenase [NAD(+)]
LOC100652121	1.512235856	5.43E-14	Up-regulated	calcium-independent phospholipase A2-gamma
LOC100644880	4.473781016	9.05E-13	Up-regulated	uncharacterized LOC100644880
LOC100642680	2.517071206	3.47E-12	Up-regulated	leucine-rich repeat-containing protein 20
LOC105667011	2.221744653	6.54E-12	Up-regulated	neuropeptide CCHamide-2 receptor
LOC105667070	1.980968243	6.54E-12	Up-regulated	uncharacterized LOC105667070
LOC100649207	3.789349915	7.33E-12	Up-regulated	flagellar attachment zone protein 1
LOC100648995	10.53783313	7.56E-12	Up-regulated	cytochrome P450 6k1 LOC100648995
LOC100643073	4.588363003	1.19E-11	Up-regulated	uncharacterized LOC100643073
LOC100645035	3.254179407	1.31E-11	Up-regulated	mpv17-like protein 2
LOC100650553	2.785269066	1.37E-11	Up-regulated	glutamic acid-rich protein
LOC110119170	1.914627019	1.37E-11	Up-regulated	uncharacterized LOC110119170
LOC100650495	4.405873247	2.15E-11	Up-regulated	troponin C
LOC100649589	0.923763439	2.95E-11	Up-regulated	dynein light chain Tctex-type protein 2B
LOC100651154	2.84789726	3.31E-11	Up-regulated	uncharacterized LOC100651154
LOC100648579	3.685176164	3.85E-11	Up-regulated	uncharacterized LOC100648579
LOC100644883	2.203438244	3.85E-11	Up-regulated	uncharacterized LOC100644883
LOC100651728	3.621302829	3.85E-11	Up-regulated	SET domain-containing protein SmydA-8
LOC100649908	0.570979355	4.46E-11	Up-regulated	uncharacterized LOC100649908
LOC100642937	3.447276016	4.76E-11	Up-regulated	actin-binding Rho-activating protein
LOC100642816	8.584165526	6.13E-11	Up-regulated	cytochrome P450 6k1 LOC100642816
LOC100646450	-0.761549328	6.73E-11	Down-regulated	intraflagellar transport protein 81 homolog
LOC100651605	1.484507809	9.47E-11	Up-regulated	microtubule-associated protein futsch
LOC100649961	1.84465295	1.23E-10	Up-regulated	glycogen-binding subunit 76A
LOC100645156	0.299466058	1.27E-10	Up-regulated	uncharacterized LOC100645156
LOC105666366	6.806932067	2.61E-10	Up-regulated	cytochrome P450 6k1 LOC105666366
LOC100648003	6.045585629	3.46E-10	Up-regulated	UDP-glycosyltransferase UGT5
LOC100649328	3.175445817	3.46E-10	Up-regulated	obscurin
LOC100650962	1.186028492	7.77E-10	Up-regulated	F-actin-monooxygenase Mical
LOC100644985	1.492502042	8.01E-10	Up-regulated	ATP-binding cassette sub-family G member 1
LOC100647853	0.721410779	8.04E-10	Up-regulated	GTP-binding protein 2
LOC100649151	3.017088944	1.06E-09	Up-regulated	zinc finger CCHC domain-containing protein 24
LOC100646597	0.82633744	1.12E-09	Up-regulated	transmembrane GTPase Marf
LOC100649306	3.960741888	1.12E-09	Up-regulated	chitin deacetylase 1
LOC100652247	3.632976953	1.39E-09	Up-regulated	cAMP-dependent protein kinase catalytic subunit PRKX
LOC100649033	3.156820849	1.46E-09	Up-regulated	cyclin-dependent kinase-like 4
LOC105665950	3.071231466	1.54E-09	Up-regulated	titin
LOC100645759	0.651304508	1.69E-09	Up-regulated	uncharacterized LOC100645759
LOC105666762	2.046686736	2.04E-09	Up-regulated	bone morphogenetic protein 2
LOC100646099	0.569119621	2.21E-09	Up-regulated	F-box only protein 21
LOC100649678	4.234245087	2.36E-09	Up-regulated	PDZ and LIM domain protein Zasp
LOC100652273	3.964815302	2.66E-09	Up-regulated	synaptic vesicle glycoprotein 2C
LOC100643658	1.062680206	2.66E-09	Up-regulated	calcium uniporter protein
LOC100643406	2.960977327	2.86E-09	Up-regulated	titin homolog
LOC100650582	3.192863169	2.86E-09	Up-regulated	uncharacterized LOC100650582
LOC100652204	1.905305729	2.91E-09	Up-regulated	alpha-actinin
LOC100642776	3.83152256	2.96E-09	Up-regulated	uncharacterized LOC100642776
LOC100647418	1.424173867	2.96E-09	Up-regulated	ERAD-associated E3 ubiquitin-protein ligase HRD1B
LOC100651289	3.709906595	3.42E-09	Up-regulated	uncharacterized LOC100651289
LOC100649392	0.623397501	4.32E-09	Up-regulated	flavin-containing monooxygenase FMO GS-OX-like 2
LOC100647631	0.566410763	5.23E-09	Up-regulated	iron-sulfur cluster assembly 1 homolog
LOC100651156	2.222848194	5.66E-09	Up-regulated	uncharacterized LOC100651156
LOC100645530	3.574810242	6.80E-09	Up-regulated	E3 ubiquitin-protein ligase lubel
LOC105665642	1.070157438	7.09E-09	Up-regulated	vinexin-like
LOC110119633	2.225811464	8.39E-09	Up-regulated	myb-like protein X
LOC100650653	1.106686495	9.64E-09	Up-regulated	uncharacterized LOC100650653
LOC100651791	1.677175136	1.04E-08	Up-regulated	NAD(+) hydrolase sarm1
LOC105665934	2.308281362	1.10E-08	Up-regulated	uncharacterized LOC105665934
LOC100643627	1.299189698	1.11E-08	Up-regulated	tubulin alpha-1 chain-like
LOC100642428	0.505372721	1.24E-08	Up-regulated	OTU domain-containing protein 7B
LOC110119541	1.536630137	1.52E-08	Up-regulated	microtubule-associated protein futsch-like
LOC100645266	0.507014756	1.56E-08	Up-regulated	DNA repair protein complementing XP-C cells homolog
LOC100649426	1.348591893	1.62E-08	Up-regulated	probable chitinase 2
LOC100642610	3.345684671	1.73E-08	Up-regulated	PDZ and LIM domain protein Zasp
LOC105666016	2.121051791	1.73E-08	Up-regulated	uncharacterized LOC105666016
LOC100652088	-0.945988994	1.91E-08	Down-regulated	sodium-coupled monocarboxylate transporter 1
LOC100643462	3.434554946	2.07E-08	Up-regulated	RING finger protein nhl-1
LOC100647623	0.805207109	2.07E-08	Up-regulated	adenylate cyclase type 2
LOC100643217	1.302147147	2.08E-08	Up-regulated	forkhead box protein O
LOC105666314	0.43567625	2.19E-08	Up-regulated	poly(ADP-ribose) glycohydrolase
LOC100651332	0.473298215	2.30E-08	Up-regulated	cAMP-dependent protein kinase type I regulatory subunit
LOC100649769	0.754056214	2.53E-08	Up-regulated	mucin-1
LOC100649554	2.621050654	2.94E-08	Up-regulated	histone-lysine N-methyltransferase SETMAR
LOC100650836	2.616985291	3.22E-08	Up-regulated	uncharacterized LOC100650836
LOC100647704	-0.388716157	3.36E-08	Down-regulated	AP-2 complex subunit mu
LOC105665610	0.350739512	3.36E-08	Up-regulated	ATPase family protein 2 homolog
LOC100645301	1.986606768	3.66E-08	Up-regulated	filamin-A
LOC100644261	0.518034035	3.67E-08	Up-regulated	complex III assembly factor LYRM7

LOC100652071	8.923823218	3.67E-08	Up-regulated	venom carboxylesterase-6
LOC100647220	2.66044656	3.72E-08	Up-regulated	esterase FE4
LOC100647419	0.464898008	4.06E-08	Up-regulated	RNA polymerase II-associated protein 3
LOC100651413	0.707585173	4.37E-08	Up-regulated	U6 snRNA-associated Sm-like protein LSm7
LOC100648770	-0.617742861	4.40E-08	Down-regulated	progestin and adipoQ receptor family member 4
LOC100643066	3.43262079	4.87E-08	Up-regulated	bestrophin-4
LOC100650665	2.508878797	5.66E-08	Up-regulated	uncharacterized LOC100650665
LOC100647860	2.749240105	5.69E-08	Up-regulated	uncharacterized LOC100647860
LOC100642853	0.678234567	6.43E-08	Up-regulated	uridine-cytidine kinase-like 1
LOC100650906	2.203086866	6.55E-08	Up-regulated	esterase FE4
LOC100645503	1.398424244	7.15E-08	Up-regulated	protein phosphatase 1 regulatory subunit 3C-B
LOC100647520	1.322924268	7.87E-08	Up-regulated	uncharacterized LOC100647520
LOC100644069	0.615990049	8.16E-08	Up-regulated	phosphorylase b kinase gamma catalytic chain
LOC105665975	3.310114477	8.75E-08	Up-regulated	golgin subfamily A member 4
LOC100644153	0.860792431	1.04E-07	Up-regulated	mitochondrial carrier protein Rim2
LOC100645400	0.427047115	1.20E-07	Up-regulated	protein misato
LOC100647053	1.808095744	1.20E-07	Up-regulated	hippocalcin-like protein 4
LOC100643507	-0.516719184	1.30E-07	Down-regulated	trypsin-3
LOC100647703	0.886999961	1.30E-07	Up-regulated	uncharacterized LOC100647703
LOC100652298	0.434123726	1.30E-07	Up-regulated	heparan-alpha-glucosaminide N-acetyltransferase
LOC100646914	1.439441318	1.61E-07	Up-regulated	circadian clock-controlled protein daywake
LOC100644902	1.800767794	1.62E-07	Up-regulated	uncharacterized LOC100644902
LOC100644931	2.221415348	1.66E-07	Up-regulated	uncharacterized LOC100644931
LOC100649005	2.206170457	1.69E-07	Up-regulated	homeobox protein ceh-17
LOC100646816	0.368699062	1.75E-07	Up-regulated	uncharacterized LOC100646816
LOC100648377	0.242770559	1.75E-07	Up-regulated	sorting nexin-14
LOC100650328	1.044961276	1.75E-07	Up-regulated	phenoloxidase-activating factor 1
LOC100646999	-0.585256178	1.93E-07	Down-regulated	Kv channel-interacting protein 2
LOC100650149	-0.413653742	2.06E-07	Down-regulated	chitinase-3-like protein 2
LOC100651217	2.701228678	2.17E-07	Up-regulated	sarcoplasmic calcium-binding protein 1
LOC100642312	-0.438498583	2.29E-07	Down-regulated	ubiquitin-like-conjugating enzyme ATG3
LOC100645351	-0.479160935	2.46E-07	Down-regulated	procollagen-lysine
LOC100646382	2.575495131	2.46E-07	Up-regulated	ras-related and estrogen-regulated growth inhibitor
LOC100649115	0.495224736	2.46E-07	Up-regulated	putative uncharacterized protein DDB_G0286901
LOC110119180	-0.491486073	2.46E-07	Down-regulated	protein THEM6
LOC110120273	-0.900685406	2.46E-07	Down-regulated	uncharacterized LOC110120273
LOC100643495	0.653618434	2.52E-07	Up-regulated	eukaryotic translation initiation factor 4E-binding protein Mextli
LOC100647540	3.32141874	2.61E-07	Up-regulated	uncharacterized LOC100647540
LOC100647812	1.062613689	3.38E-07	Up-regulated	ATP synthase subunit s
LOC100642842	-0.543982719	3.41E-07	Down-regulated	heterogeneous nuclear ribonucleoprotein M
LOC100646249	-0.418379142	3.63E-07	Down-regulated	uncharacterized LOC100646249
LOC100644974	-0.47146661	3.90E-07	Down-regulated	probable multidrug resistance-associated protein lethal(2)03659
LOC100642958	-0.56128949	3.98E-07	Down-regulated	uncharacterized LOC100642958
LOC100646897	0.48085608	3.98E-07	Up-regulated	uncharacterized LOC100646897
LOC100649873	0.495516544	4.08E-07	Up-regulated	ubiquitin-conjugating enzyme E2 G1
LOC100649404	0.238913745	4.42E-07	Up-regulated	mitochondrial-processing peptidase subunit alpha
LOC100645782	0.876844362	4.91E-07	Up-regulated	branched-chain-amino-acid aminotransferase
LOC100652068	1.553214951	5.24E-07	Up-regulated	protein lethal(2)essential for life
LOC100649174	3.648398577	5.48E-07	Up-regulated	calcium-dependent protein kinase 16
LOC105666782	1.524666509	5.48E-07	Up-regulated	uncharacterized LOC105666782
LOC100649035	0.758114619	5.69E-07	Up-regulated	BCL2/adenovirus E1B 19 kDa protein-interacting protein 3
LOC100644802	0.581393415	6.05E-07	Up-regulated	twisted gastrulation protein homolog 1-B
LOC105666696	1.137837952	6.71E-07	Up-regulated	uncharacterized LOC105666696
LOC100652272	0.429611942	6.85E-07	Up-regulated	ribosome biogenesis protein WDR12 homolog
LOC100646297	-0.473472927	7.16E-07	Down-regulated	clusterin-associated protein 1
LOC100645227	0.420912451	7.41E-07	Up-regulated	glycerophosphocholine phosphodiesterase GPCPD1
LOC100651796	1.612140848	7.75E-07	Up-regulated	organic cation transporter protein
LOC100647290	1.502593844	8.45E-07	Up-regulated	SET and MYND domain-containing protein 4
LOC105666419	0.336945643	8.46E-07	Up-regulated	ornithine decarboxylase antizyme 1
LOC100642504	-0.689812167	8.58E-07	Down-regulated	trichohyalin
LOC100649208	1.445605667	8.58E-07	Up-regulated	homeobox protein SIX1
LOC100645505	-0.334136948	8.67E-07	Down-regulated	protein phosphatase 1 regulatory subunit 14B
LOC100646544	2.462352422	8.76E-07	Up-regulated	uncharacterized LOC100646544
LOC100650427	8.201778073	8.92E-07	Up-regulated	cytochrome P450 6k1 LOC100650427
LOC100650077	3.047002304	9.17E-07	Up-regulated	translation initiation factor IF-2
LOC100646948	0.4375974	9.48E-07	Up-regulated	tyrosine-protein phosphatase non-receptor type 2
LOC100646042	0.600222738	9.50E-07	Up-regulated	protein krasavietz
LOC100649179	-0.449025534	9.99E-07	Down-regulated	EH domain-binding protein 1
LOC100652215	0.432430408	1.01E-06	Up-regulated	ubiquitin-conjugating enzyme E2 R2
LOC100650136	0.626697179	1.04E-06	Up-regulated	adenylosuccinate synthetase
LOC100643938	-0.678530533	1.05E-06	Down-regulated	Kv channel-interacting protein 1
LOC100650291	1.522609911	1.11E-06	Up-regulated	four and a half LIM domains protein 2
LOC100646800	-0.467087866	1.17E-06	Down-regulated	PI-PLC X domain-containing protein 2
LOC100643338	1.431784233	1.23E-06	Up-regulated	leucine-rich repeat extensin-like protein 5
LOC100645831	-1.519033567	1.24E-06	Down-regulated	elongation of very long chain fatty acids protein 1
LOC100650368	0.292019746	1.24E-06	Up-regulated	NF-X1-type zinc finger protein NFXL1
LOC100648065	0.905158819	1.29E-06	Up-regulated	centrosomin
LOC100646448	1.238474332	1.34E-06	Up-regulated	intraflagellar transport protein 80 homolog
LOC100646293	0.503872773	1.46E-06	Up-regulated	TLD domain-containing protein 2
LOC100652292	0.547632496	1.46E-06	Up-regulated	prolyl 3-hydroxylase OGFOD1
LOC105665830	0.86061025	1.46E-06	Up-regulated	uncharacterized LOC105665830

LOC100650066	0.583878527	1.48E-06	Up-regulated	ABC transporter F family member 4
LOC100642622	0.400916342	1.48E-06	Up-regulated	DNA-directed RNA polymerase II subunit RPB2
LOC100648768	-0.373395831	1.48E-06	Down-regulated	sodium-independent sulfate anion transporter
LOC100650785	1.250330265	1.50E-06	Up-regulated	facilitated trehalose transporter Tret1
LOC100645330	-0.401042443	1.52E-06	Down-regulated	synaptic vesicle 2-related protein
LOC100650807	1.037149928	1.52E-06	Up-regulated	uncharacterized LOC100650807
LOC100648814	1.618232185	1.53E-06	Up-regulated	uncharacterized LOC100648814
LOC110119183	0.954722033	1.57E-06	Up-regulated	sodium/potassium-transporting ATPase subunit beta-2
LOC100646909	3.31597846	1.57E-06	Up-regulated	uncharacterized LOC100646909
LOC100651497	-0.454876119	1.57E-06	Down-regulated	uncharacterized LOC100651497
LOC110120039	1.605317112	1.57E-06	Up-regulated	uncharacterized LOC110120039
LOC100648339	0.319113939	1.65E-06	Up-regulated	nuclear distribution protein nudE-like 1
LOC100652140	-0.563045682	1.67E-06	Down-regulated	aldo-keto reductase family 1 member B1
LOC110119222	0.429593483	1.67E-06	Up-regulated	zinc finger and BTB domain-containing protein 41-like
LOC100645959	1.879497907	1.73E-06	Up-regulated	uncharacterized LOC100645959
LOC110120173	-1.008190411	1.81E-06	Down-regulated	uncharacterized LOC110120173
LOC100651076	0.747860376	1.86E-06	Up-regulated	voltage-dependent calcium channel subunit alpha-2/delta-3
LOC100648708	0.455152007	1.93E-06	Up-regulated	DNA excision repair protein ERCC-5 homolog
LOC105666138	1.973752365	1.93E-06	Up-regulated	15-hydroxyprostaglandin dehydrogenase [NAD(+)]-like
LOC100650692	-0.641299136	2.00E-06	Down-regulated	beta-1
LOC100646026	1.969431867	2.03E-06	Up-regulated	serine/arginine repetitive matrix protein 1
LOC100648802	0.908512877	2.14E-06	Up-regulated	MORN repeat-containing protein 5
LOC100647118	-0.469639525	2.16E-06	Down-regulated	uncharacterized LOC100647118
LOC100646216	0.592739596	2.22E-06	Up-regulated	homeodomain-interacting protein kinase 2
LOC100652051	-0.614907715	2.30E-06	Down-regulated	DNA fragmentation factor subunit beta
LOC110119437	-1.039186296	2.30E-06	Down-regulated	lipase member H
LOC100642961	-0.588951636	2.36E-06	Down-regulated	lysocardiolipin acyltransferase 1
LOC100643893	0.416771649	2.37E-06	Up-regulated	AFG3-like protein 2
LOC100651572	0.390935461	2.37E-06	Up-regulated	klaroid protein
LOC100643293	1.273598	2.54E-06	Up-regulated	probable inactive protein kinase DDB_G0270444
LOC100645349	0.541570149	2.54E-06	Up-regulated	spermine oxidase
LOC100645462	0.452204799	2.54E-06	Up-regulated	uncharacterized LOC100645462
LOC100643909	0.513126547	2.54E-06	Up-regulated	upstream activation factor subunit spp27
LOC100649558	-0.299045636	2.60E-06	Down-regulated	dynamin-like 120 kDa protein
LOC100648165	0.87607436	2.61E-06	Up-regulated	phytanoyl-CoA dioxygenase domain-containing protein 1 homolog
LOC100644051	0.309664552	2.68E-06	Up-regulated	DDB1- and CUL4-associated factor 8
LOC100647387	0.784671786	2.72E-06	Up-regulated	arfGAP with SH3 domain
LOC100644118	2.388316678	2.78E-06	Up-regulated	twitchin
LOC100649087	3.1961474	2.91E-06	Up-regulated	uncharacterized LOC100649087
LOC105666200	-0.441526828	2.93E-06	Down-regulated	transmembrane protein 222
LOC100643207	-0.529406213	2.98E-06	Down-regulated	methylthioribose-1-phosphate isomerase
LOC100649433	0.306830975	3.18E-06	Up-regulated	transmembrane protein 184C
LOC100644990	1.306403804	3.46E-06	Up-regulated	uncharacterized LOC100644990
LOC100651679	0.381863239	3.46E-06	Up-regulated	coiled-coil and C2 domain-containing protein 1-like
LOC110120152	1.266084679	3.46E-06	Up-regulated	vinexin-like
LOC100642241	1.310353116	3.47E-06	Up-regulated	uncharacterized LOC100642241
LOC100652163	-0.45361722	3.47E-06	Down-regulated	cyclin-dependent kinase 14
LOC100644310	0.995933604	3.63E-06	Up-regulated	TATA box-binding protein-associated factor RNA polymerase I subunit B
LOC100650381	-0.516327251	3.63E-06	Down-regulated	RNA-binding protein spenito
LOC100646601	0.323317239	3.75E-06	Up-regulated	protein kintoun
LOC100649549	1.057633469	3.85E-06	Up-regulated	mucin-5AC
LOC100652201	-0.476219395	3.86E-06	Down-regulated	stress response protein NST1
LOC100648822	-0.407480428	3.93E-06	Down-regulated	RRP15-like protein
LOC100651498	0.810663366	4.16E-06	Up-regulated	MAP kinase-interacting serine/threonine-protein kinase 1
LOC100647646	3.031195763	4.37E-06	Up-regulated	UDP-glycosyltransferase UGT5-like
LOC100643706	0.493431087	4.39E-06	Up-regulated	pre-mRNA-splicing factor RBM22
LOC100650746	-0.55862499	4.39E-06	Down-regulated	apoptosis-resistant E3 ubiquitin protein ligase 1
LOC100648428	-0.489938246	4.51E-06	Down-regulated	F-box only protein 7
LOC100651554	1.447031551	4.98E-06	Up-regulated	uncharacterized serine-rich protein C215.13
LOC100646995	2.323416764	5.05E-06	Up-regulated	uncharacterized LOC100646995
LOC100644899	0.325264064	5.24E-06	Up-regulated	protein tramtrack
LOC100645193	0.312260615	5.27E-06	Up-regulated	DNA polymerase iota
LOC100649778	0.216959273	5.27E-06	Up-regulated	spermatogenesis-associated protein 20
LOC100645580	0.419373712	5.30E-06	Up-regulated	guanine nucleotide-binding protein subunit gamma-1
LOC100645543	0.330313002	5.36E-06	Up-regulated	importin-5
LOC105665957	-0.387640433	5.39E-06	Down-regulated	syntaxin-6
LOC100645933	2.482095458	5.42E-06	Up-regulated	homeobox protein six1
LOC100647396	0.437006441	5.44E-06	Up-regulated	protein CLEC16A homolog
LOC100648429	-0.708333994	5.51E-06	Down-regulated	titin
LOC100649730	0.654196407	5.51E-06	Up-regulated	uncharacterized LOC100649730
LOC105666125	0.690783163	5.54E-06	Up-regulated	MRN complex-interacting protein
LOC100642931	0.631505568	5.68E-06	Up-regulated	glycogen phosphorylase
LOC100649226	0.490804394	5.70E-06	Up-regulated	protein unc-45 homolog B
LOC100650040	-0.379234212	6.30E-06	Down-regulated	uncharacterized LOC100650040
LOC100649474	-0.484630839	6.31E-06	Down-regulated	EF-hand domain-containing protein D2 homolog
LOC100649546	1.397745015	6.45E-06	Up-regulated	uncharacterized LOC100649546
LOC100648899	-0.267277649	6.69E-06	Down-regulated	vacuolar protein sorting-associated protein 8 homolog
LOC105666795	-0.925553157	7.00E-06	Down-regulated	tigger transposable element-derived protein 2-like
LOC100649083	-0.338651829	7.66E-06	Down-regulated	glycosaminoglycan xylosylkinase
LOC105665724	1.331460894	7.66E-06	Up-regulated	transcription factor kayak
LOC100649110	4.791181046	7.67E-06	Up-regulated	cytochrome P450 6k1 LOC100649110

LOC100646507	0.36973817	7.93E-06	Up-regulated	bromodomain-containing protein DDB_G0280777
LOC100647689	-0.623805925	8.12E-06	Down-regulated	ras-related protein Rap-2a
LOC100652095	-0.484804139	8.12E-06	Down-regulated	rab proteins geranylgeranyltransferase component A 1
LOC100647225	1.398743574	8.35E-06	Up-regulated	retinol dehydrogenase 10-A
LOC100647695	0.686071215	8.35E-06	Up-regulated	circadian locomoter output cycles protein kaput
LOC100645081	0.382805179	8.35E-06	Up-regulated	nucleobindin-2
LOC100644182	0.52334092	8.37E-06	Up-regulated	uncharacterized LOC100644182
LOC100645524	-1.11598847	8.37E-06	Down-regulated	heat shock protein 83
LOC100650912	0.39290043	8.37E-06	Up-regulated	RNA-binding protein squid
LOC100647217	-0.528063197	8.76E-06	Down-regulated	neurotrimin
LOC100651390	-1.135581619	8.78E-06	Down-regulated	gamma-aminobutyric acid receptor alpha-like
LOC100643882	0.419126676	9.51E-06	Up-regulated	U4/U6.U5 tri-snRNP-associated protein 1
LOC100651528	0.318596559	9.51E-06	Up-regulated	4'-phosphopantetheine phosphatase
LOC100647185	-1.157398004	9.51E-06	Down-regulated	one cut domain family member 3
LOC100651658	2.764818582	9.60E-06	Up-regulated	uncharacterized LOC100651658
LOC100643394	1.488934445	9.92E-06	Up-regulated	F-box/LRR-repeat protein fbxl-1
LOC100646488	-1.485517406	9.96E-06	Down-regulated	uncharacterized LOC100646488
LOC100652179	-0.308036962	1.01E-05	Down-regulated	WD and tetratricopeptide repeats protein 1
LOC100644627	1.017877381	1.07E-05	Up-regulated	heat shock protein beta-1
LOC105666613	-1.827516146	1.08E-05	Down-regulated	uncharacterized LOC105666613
LOC100645084	0.613952623	1.11E-05	Up-regulated	glycerate kinase
LOC105665825	2.153572676	1.12E-05	Up-regulated	treacle protein
LOC100649149	1.24553129	1.12E-05	Up-regulated	facilitated trehalose transporter Tret1
LOC100648876	-0.361894	1.13E-05	Down-regulated	DNA topoisomerase 3-beta-1
LOC100647790	0.29065914	1.13E-05	Up-regulated	uncharacterized LOC100647790
LOC100646594	0.431482876	1.14E-05	Up-regulated	nuclear pore complex protein Nup98-Nup96
LOC100646745	-0.555521005	1.22E-05	Down-regulated	uncharacterized LOC100646745
LOC100651738	0.662027097	1.22E-05	Up-regulated	uncharacterized LOC100651738
LOC100650403	0.497392545	1.22E-05	Up-regulated	probable protein phosphatase 2C T23F11.1
LOC105666994	0.794517949	1.22E-05	Up-regulated	copia protein
LOC100651954	-0.475846671	1.24E-05	Down-regulated	protein nervous wreck
LOC100651507	-0.453300357	1.24E-05	Down-regulated	39S ribosomal protein L42
LOC100644954	-0.323928226	1.27E-05	Down-regulated	AP-1 complex subunit beta-1
LOC100649704	-0.367957326	1.34E-05	Down-regulated	calmodulin
LOC100651339	-0.457681887	1.34E-05	Down-regulated	leucine-rich repeat-containing protein 57
LOC105666322	-0.492829882	1.34E-05	Down-regulated	protein lethal(2)essential for life
LOC100650517	-0.513254891	1.42E-05	Down-regulated	nitric oxide synthase
LOC100642328	-0.647261163	1.44E-05	Down-regulated	sushi
LOC100645908	-0.499010995	1.48E-05	Down-regulated	elongation of very long chain fatty acids protein 4
LOC100649314	-0.633797035	1.48E-05	Down-regulated	eyes absent homolog 4
LOC100650096	-0.231995435	1.49E-05	Down-regulated	PRELI domain-containing protein 1
LOC100650696	-0.335563182	1.50E-05	Down-regulated	protein ROP
LOC100644488	0.583210632	1.52E-05	Up-regulated	homeobox protein 14
LOC100652041	1.444447006	1.57E-05	Up-regulated	uncharacterized LOC100652041
LOC105665951	-0.372765304	1.58E-05	Down-regulated	inositol monophosphatase 1
LOC100646983	1.715180658	1.59E-05	Up-regulated	uncharacterized LOC100646983
LOC100643134	0.928064221	1.61E-05	Up-regulated	U11/U12 small nuclear ribonucleoprotein 48 kDa protein
LOC100642903	2.382050847	1.62E-05	Up-regulated	myosin regulatory light chain 2
LOC100646089	-1.09628954	1.67E-05	Down-regulated	uncharacterized LOC100646089
LOC100643316	0.348413251	1.71E-05	Up-regulated	SWI/SNF-related matrix-associated actin-dependent regulator of chromatin
LOC100651601	2.205187896	1.72E-05	Up-regulated	tropomyosin-2
LOC100650822	0.24492317	1.73E-05	Up-regulated	hsp70-binding protein 1
LOC100646016	-0.379237463	1.73E-05	Down-regulated	HIRA-interacting protein 3
LOC105665914	0.706494014	1.73E-05	Up-regulated	glomulin
LOC100646275	-0.765104518	1.75E-05	Down-regulated	cilia- and flagella-associated protein 157
LOC100648923	-0.427491823	1.76E-05	Down-regulated	synaptotagmin 1
LOC100643251	-0.315362684	1.77E-05	Down-regulated	kinesin heavy chain
LOC100647931	0.588749767	1.77E-05	Up-regulated	rab11 family-interacting protein 4A
LOC100651946	0.435321685	1.78E-05	Up-regulated	ras-related protein Rab-39B
LOC100648861	0.350260302	1.79E-05	Up-regulated	ribonucleoside-diphosphate reductase large subunit
LOC100642607	-0.508865816	1.85E-05	Down-regulated	peroxidasin homolog
LOC100646449	-0.736825684	1.85E-05	Down-regulated	beta-1
LOC100651597	0.385250828	1.90E-05	Up-regulated	elongator complex protein 3
LOC100644016	-0.541352784	1.92E-05	Down-regulated	transmembrane protein 62
LOC100649043	0.519411697	1.97E-05	Up-regulated	mediator of RNA polymerase II transcription subunit 9
LOC100645655	-0.458376901	2.00E-05	Down-regulated	lateral signaling target protein 2 homolog
LOC100644288	0.768937551	2.00E-05	Up-regulated	papilin
LOC105666149	0.835521997	2.06E-05	Up-regulated	histone demethylase UTY
LOC100650827	1.037544994	2.08E-05	Up-regulated	uncharacterized LOC100650827
LOC105667097	0.735719553	2.08E-05	Up-regulated	mitochondrial pyruvate carrier 4
LOC100645176	0.450133911	2.13E-05	Up-regulated	origin recognition complex subunit 1
LOC100651024	0.948042749	2.17E-05	Up-regulated	glycerol-3-phosphate dehydrogenase
LOC100643050	2.235729897	2.19E-05	Up-regulated	sodium channel protein Nach
LOC100645452	1.569919169	2.20E-05	Up-regulated	apolipoprotein D
LOC100649267	0.440957931	2.21E-05	Up-regulated	uncharacterized LOC100649267
LOC100643291	2.480653031	2.22E-05	Up-regulated	RNA-binding protein 24
LOC100650162	0.675111162	2.22E-05	Up-regulated	5'-nucleotidase domain-containing protein 3
LOC100643749	0.309145028	2.25E-05	Up-regulated	serine-rich adhesin for platelets
LOC100646793	1.633834133	2.25E-05	Up-regulated	PDZ and LIM domain protein 3
LOC100645233	-0.411770693	2.28E-05	Down-regulated	uncharacterized LOC100645233
LOC100642663	-0.360557647	2.30E-05	Down-regulated	quinone oxidoreductase-like protein 2

LOC105666145	1.379860643	2.35E-05	Up-regulated	uncharacterized LOC105666145
LOC100645482	0.645019515	2.42E-05	Up-regulated	ribonuclease P protein subunit p25-like protein
LOC100644616	0.638441712	2.43E-05	Up-regulated	ankyrin repeat and SOCS box protein 16
LOC100642200	-0.65228214	2.47E-05	Down-regulated	long-chain fatty acid transport protein 4
LOC100647732	-0.625789469	2.47E-05	Down-regulated	uncharacterized LOC100647732
LOC100649427	-0.646056619	2.47E-05	Down-regulated	uncharacterized LOC100649427
LOC100652251	0.724451307	2.47E-05	Up-regulated	RYamide receptor
LOC100649399	-0.405347175	2.48E-05	Down-regulated	T-cell activation inhibitor
LOC100646420	-0.674753315	2.49E-05	Down-regulated	neurexin-4
LOC100644683	1.892618368	2.52E-05	Up-regulated	uncharacterized LOC100644683
LOC100650992	-0.32296974	2.54E-05	Down-regulated	protein abrupt
LOC100645338	2.993400203	2.54E-05	Up-regulated	ATP-binding cassette sub-family C member 4
LOC100649981	-0.556406469	2.56E-05	Down-regulated	1-phosphatidylinositol 4
LOC100650220	-0.539506446	2.59E-05	Down-regulated	multiple inositol polyphosphate phosphatase 1
LOC100649977	1.097064011	2.70E-05	Up-regulated	uncharacterized LOC100649977
LOC100651811	-0.669733856	2.72E-05	Down-regulated	palmitoyltransferase ZDHHC5-A
LOC100650398	0.262581941	2.78E-05	Up-regulated	uncharacterized LOC100650398
LOC100642349	0.36949257	2.78E-05	Up-regulated	cytosolic purine 5'-nucleotidase
LOC100643646	0.692236726	2.94E-05	Up-regulated	growth arrest-specific protein 1
LOC100647190	-0.648960806	2.94E-05	Down-regulated	transcription factor Sox-19b
LOC100646396	-0.294961653	2.96E-05	Down-regulated	UPF0415 protein C7orf25 homolog
LOC100645346	-0.427707109	2.96E-05	Down-regulated	vesicle-associated membrane protein 2
LOC100631054	0.316073892	3.04E-05	Up-regulated	dorsal
LOC100652042	-0.419442148	3.06E-05	Down-regulated	lysophosphatidylcholine acyltransferase
LOC100642652	1.859899321	3.10E-05	Up-regulated	calcium-transporting ATPase sarcoplasmic/endoplasmic reticulum type
LOC100646486	0.97823286	3.10E-05	Up-regulated	cilia- and flagella-associated protein 61
LOC100645320	0.736595013	3.11E-05	Up-regulated	mitochondrial import inner membrane translocase subunit TIM14
LOC100647747	-0.572958558	3.17E-05	Down-regulated	uncharacterized LOC100647747
LOC100650457	-0.555189066	3.29E-05	Down-regulated	uncharacterized oxidoreductase YjmC
LOC100645183	-0.262195017	3.34E-05	Down-regulated	myotubularin-related protein 13
LOC100652234	0.301349575	3.34E-05	Up-regulated	zinc finger protein 330 homolog
LOC105665620	-0.350027443	3.40E-05	Down-regulated	serine--tRNA synthetase-like protein Slimp
LOC110119185	0.320707582	3.41E-05	Up-regulated	novel acetylcholine receptor chaperone
LOC100647119	-0.412103567	3.42E-05	Down-regulated	ATP-dependent RNA helicase dbp2
LOC100643874	0.404396871	3.45E-05	Up-regulated	zinc finger protein OZF
LOC100652148	0.672896595	3.45E-05	Up-regulated	sterol regulatory element-binding protein 1
LOC100647226	0.353824352	3.53E-05	Up-regulated	UDP-glucuronic acid decarboxylase 1
LOC100647240	0.314350684	3.53E-05	Up-regulated	succinate--CoA ligase [ADP-forming] subunit beta
LOC100645694	-0.313222192	3.55E-05	Down-regulated	phospholipid scramblase 3
LOC100643816	-0.855237311	3.62E-05	Down-regulated	frizzled-2
LOC100643512	2.602243964	3.72E-05	Up-regulated	lysosomal acid glucosylceramidase
LOC100642644	-0.586327763	3.78E-05	Down-regulated	uncharacterized LOC100642644
LOC100648973	0.641237848	3.79E-05	Up-regulated	protein GDAP2 homolog
LOC100646226	-0.467955974	3.82E-05	Down-regulated	TBC1 domain family member 16
LOC100645716	1.419289376	3.84E-05	Up-regulated	uncharacterized LOC100645716
LOC105666201	0.418560163	3.90E-05	Up-regulated	protein Spindly
LOC100642980	1.634864912	3.94E-05	Up-regulated	hemocytin
LOC105666988	0.926640448	3.96E-05	Up-regulated	bromodomain-containing protein DDB_G0280777
LOC100645915	-0.43097438	3.98E-05	Down-regulated	potassium/sodium hyperpolarization-activated cyclic nucleotide-gated channel 2
LOC100646091	-0.740968794	4.00E-05	Down-regulated	cilia- and flagella-associated protein 91
LOC100643770	0.367999109	4.07E-05	Up-regulated	trans-Golgi network integral membrane protein 1
LOC100650003	-0.403638205	4.07E-05	Down-regulated	uncharacterized LOC100650003
LOC100643620	-0.325965981	4.09E-05	Down-regulated	inactive selenide
LOC100648073	1.086311296	4.12E-05	Up-regulated	dehydrogenase/reductase SDR family member 11
LOC100649106	0.648066718	4.17E-05	Up-regulated	putative glutathione-specific gamma-glutamylcyclotransferase 2
LOC100651748	-0.910654226	4.17E-05	Down-regulated	phospholipase A1
LOC100642483	0.583178812	4.26E-05	Up-regulated	TBC1 domain family member 1
LOC100651200	-0.255816763	4.31E-05	Down-regulated	apoptosis-inducing factor 1
LOC100646706	-0.309377462	4.46E-05	Down-regulated	von Willebrand factor A domain-containing protein 8
LOC105666938	-1.134223683	4.89E-05	Down-regulated	uncharacterized LOC105666938
LOC100651326	-0.280395898	4.91E-05	Down-regulated	RING finger and CHY zinc finger domain-containing protein 1
LOC100644475	-0.483057174	4.92E-05	Down-regulated	lachesin
LOC105665677	1.007156588	4.92E-05	Up-regulated	TNF receptor-associated factor 5
LOC100643575	-0.533137742	4.92E-05	Down-regulated	microtubule-associated protein futsch
LOC100642886	-0.405054877	4.93E-05	Down-regulated	leucine-zipper-like transcriptional regulator 1
LOC100645663	0.351688105	4.97E-05	Up-regulated	protein ELYS
LOC100650361	-0.838368921	4.97E-05	Down-regulated	uncharacterized LOC100650361
LOC100647629	0.448301752	5.01E-05	Up-regulated	HMG box transcription factor BBX
LOC100651141	0.330325196	5.02E-05	Up-regulated	serine/threonine-protein kinase grp
LOC100651197	-0.519892321	5.23E-05	Down-regulated	aldo-keto reductase family 1 member A1
LOC100650928	-0.775982578	5.25E-05	Down-regulated	uncharacterized LOC100650928
LOC100648431	0.567509714	5.39E-05	Up-regulated	protein mothers against dpp
LOC100646271	-0.404218087	5.49E-05	Down-regulated	RCC1 domain-containing protein 1
LOC100648152	0.439842079	5.49E-05	Up-regulated	brain protein I3
LOC100649186	0.442371979	5.59E-05	Up-regulated	structural maintenance of chromosomes protein 6
LOC100647749	0.447669212	5.59E-05	Up-regulated	zinc transporter ZIP9
LOC100642474	-0.87154328	5.60E-05	Down-regulated	protein giant-lens
LOC100645149	-0.433993577	5.60E-05	Down-regulated	RIMS-binding protein 2
LOC105666087	2.857583462	5.60E-05	Up-regulated	uncharacterized LOC105666087
LOC100643044	-0.316402972	5.61E-05	Down-regulated	probable serine/threonine-protein kinase roco9
LOC100650415	0.804158859	5.64E-05	Up-regulated	DNA helicase MCM9

LOC100646432	0.273383665	5.74E-05	Up-regulated	copper-transporting ATPase 1
LOC100642205	0.520243403	5.77E-05	Up-regulated	protein krueppel
LOC100645087	0.364607124	5.87E-05	Up-regulated	transcription initiation factor TFIID subunit 9
LOC100651600	0.22782439	5.99E-05	Up-regulated	probable maleylacetoacetate isomerase 2
LOC100644780	0.451993912	6.11E-05	Up-regulated	thioredoxin
LOC100648852	-0.532815668	6.11E-05	Down-regulated	endonuclease G
LOC100648381	-0.378070685	6.15E-05	Down-regulated	potassium channel subfamily T member 2
LOC100651027	0.436332122	6.15E-05	Up-regulated	alpha-ketoglutarate-dependent dioxygenase alkB homolog 4
LOC100645803	1.457601732	6.19E-05	Up-regulated	nuclear factor 1 X-type
LOC105666345	-1.055914664	6.23E-05	Down-regulated	uncharacterized LOC105666345
LOC100649183	0.555165645	6.28E-05	Up-regulated	mRNA turnover protein 4 homolog
LOC100643834	-0.753973426	6.39E-05	Down-regulated	uncharacterized LOC100643834
LOC100648679	-0.446363287	6.51E-05	Down-regulated	protein FAM166B
LOC100645598	3.127677526	6.53E-05	Up-regulated	synaptic vesicle glycoprotein 2B
LOC110120040	2.165790651	6.53E-05	Up-regulated	uncharacterized LOC110120040
LOC100642965	1.386624326	6.63E-05	Up-regulated	uncharacterized LOC100642965
LOC110119952	1.066331849	6.79E-05	Up-regulated	uncharacterized LOC110119952
LOC100644256	-0.329014538	6.84E-05	Down-regulated	mitogen-activated protein kinase kinase kinase 12
LOC100651335	-0.373847566	6.86E-05	Down-regulated	sodium-dependent phosphate transporter 2
LOC100649105	0.314637191	6.96E-05	Up-regulated	forkhead box protein N3
LOC100650884	0.684132317	7.02E-05	Up-regulated	serine/threonine-protein phosphatase 4 regulatory subunit 1
LOC100648442	0.421221627	7.07E-05	Up-regulated	N-alpha-acetyltransferase 30
LOC100644046	0.498086253	7.13E-05	Up-regulated	GTP-binding protein Rheb homolog
LOC100650642	-0.559865523	7.23E-05	Down-regulated	probable multidrug resistance-associated protein lethal(2)03659
LOC100645404	0.460021705	7.37E-05	Up-regulated	protein mago nashi homolog
LOC100648383	0.857988985	7.43E-05	Up-regulated	dynein heavy chain 5
LOC100642345	-0.420717357	7.65E-05	Down-regulated	glycolipid transfer protein
LOC100646390	-0.473934268	7.65E-05	Down-regulated	zwei Ig domain protein zig-8
LOC100649094	-0.444135419	7.79E-05	Down-regulated	protein strawberry notch
LOC105666150	0.88072533	7.89E-05	Up-regulated	histone demethylase UTY
LOC100643390	0.338238101	8.01E-05	Up-regulated	30S ribosomal protein S11
LOC100648892	-0.621226402	8.01E-05	Down-regulated	quinone oxidoreductase
LOC100648754	-0.408135515	8.04E-05	Down-regulated	protein lin-7 homolog C
LOC105665758	0.797407554	8.04E-05	Up-regulated	neurochondrin homolog
LOC100651705	-0.279289574	8.19E-05	Down-regulated	dnaJ homolog subfamily C member 8
LOC100646666	1.833923514	8.22E-05	Up-regulated	receptor-type tyrosine-protein phosphatase kappa
LOC100647835	0.992585607	8.25E-05	Up-regulated	vanin-like protein 1
LOC105666538	0.7106587	8.37E-05	Up-regulated	uncharacterized LOC105666538
LOC100644945	0.317862855	8.43E-05	Up-regulated	mitogen-activated protein kinase kinase kinase 15
LOC100648414	-0.660814388	8.50E-05	Down-regulated	uncharacterized LOC100648414
LOC100645645	0.498602445	8.51E-05	Up-regulated	FAD synthase
LOC100644306	0.552079455	8.52E-05	Up-regulated	CKLF-like MARVEL transmembrane domain-containing protein 4
LOC100650119	-0.455355241	8.55E-05	Down-regulated	protein cueball
LOC100644749	-0.721671685	8.64E-05	Down-regulated	alkaline phosphatase 4
LOC100648227	0.707935085	8.68E-05	Up-regulated	uncharacterized LOC100648227
LOC100647457	1.057857873	8.68E-05	Up-regulated	leupaxin
LOC100643821	1.006213482	8.73E-05	Up-regulated	protein CREG1
LOC100646138	0.252874063	8.73E-05	Up-regulated	protein PRRC1-B
LOC100647451	-0.335471784	8.74E-05	Down-regulated	retinoblastoma-binding protein 5 homolog
LOC100650845	0.282514965	8.81E-05	Up-regulated	transport and Golgi organization protein 1
LOC100647556	-0.975717245	8.82E-05	Down-regulated	lysyl oxidase homolog 3A
LOC100650636	-0.454102666	8.86E-05	Down-regulated	tolloid-like protein 1
LOC100644977	-0.447480525	8.91E-05	Down-regulated	uncharacterized LOC100644977
LOC100647029	0.459575613	8.92E-05	Up-regulated	protein kinase C-binding protein 1
LOC100648765	1.183761417	8.93E-05	Up-regulated	neprilysin-2
LOC100646337	0.760286461	8.98E-05	Up-regulated	CBP80/20-dependent translation initiation factor
LOC100646205	1.742654821	9.11E-05	Up-regulated	uncharacterized LOC100646205
LOC100642502	-0.487394257	9.13E-05	Down-regulated	biotin--protein ligase
LOC100645561	0.375910689	9.14E-05	Up-regulated	transcription factor GAGA
LOC100644497	2.019780669	9.19E-05	Up-regulated	uncharacterized LOC100644497
LOC100651532	-0.258210231	9.19E-05	Down-regulated	serine/threonine-protein kinase 3
LOC100650208	0.331545057	9.49E-05	Up-regulated	uncharacterized LOC100650208
LOC110119181	0.749843446	9.81E-05	Up-regulated	pyrimidine-specific ribonucleoside hydrolase RihA
LOC100648720	1.035041676	9.89E-05	Up-regulated	elongation of very long chain fatty acids protein AAEL008004
LOC105665619	-0.406796919	9.89E-05	Down-regulated	zinc finger protein 425
LOC100642994	-0.342546825	9.92E-05	Down-regulated	palmitoyltransferase ZDHHC6
LOC105666637	1.08087902	1.07E-04	Up-regulated	calcium-independent protein kinase C
LOC100651943	1.991247447	1.07E-04	Up-regulated	UDP-glucosyltransferase 2-like
LOC100643007	2.039925762	1.08E-04	Up-regulated	uncharacterized LOC100643007
LOC100647746	0.349368123	1.08E-04	Up-regulated	ATP-dependent RNA helicase p62
LOC100649700	-0.533336677	1.08E-04	Down-regulated	excitatory amino acid transporter 1
LOC100650471	-0.530655555	1.08E-04	Down-regulated	regulating synaptic membrane exocytosis protein 1
LOC100649441	-1.079273008	1.09E-04	Down-regulated	probable cytochrome P450 LOC100649441
LOC100648793	-0.277582258	1.15E-04	Down-regulated	ATP-dependent 6-phosphofructokinase
LOC105666747	0.437235753	1.16E-04	Up-regulated	transmembrane emp24 domain-containing protein 7
LOC100651991	0.575153701	1.17E-04	Up-regulated	uncharacterized LOC100651991
LOC100643194	-0.680865879	1.17E-04	Down-regulated	regulator of G-protein signaling 17
LOC100644161	0.456617309	1.17E-04	Up-regulated	uncharacterized LOC100644161
LOC100644376	-0.297335056	1.18E-04	Down-regulated	cystathionine beta-synthase
LOC100645934	-0.341947113	1.18E-04	Down-regulated	alpha-ketoglutarate dehydrogenase component 4
LOC100648303	3.478948375	1.19E-04	Up-regulated	putative fatty acyl-CoA reductase CG5065

LOC100643811	0.485449849	1.20E-04	Up-regulated	ADP-ribosylhydrolase ARH3
LOC100651488	0.240749982	1.20E-04	Up-regulated	ubiquitin thioesterase OTU1
LOC100642730	-0.642103265	1.20E-04	Down-regulated	innexin inx1
LOC100648162	1.084823789	1.20E-04	Up-regulated	uncharacterized LOC100648162
LOC100643063	-0.447636409	1.21E-04	Down-regulated	D-aspartate oxidase
LOC100644120	-0.233507337	1.21E-04	Down-regulated	protein phosphatase 1H
LOC100643845	0.570282741	1.24E-04	Up-regulated	carbohydrate-responsive element-binding protein
LOC100643931	-0.930752719	1.24E-04	Down-regulated	zinc transporter ZIP3
LOC100649994	-0.225719572	0.000126354	Down-regulated	pre-mRNA-processing factor 39
LOC100650705	0.727270758	0.000126354	Up-regulated	basic proline-rich protein
LOC100646034	-0.661591113	1.27E-04	Down-regulated	von Hippel-Lindau disease tumor suppressor
LOC100642292	-0.4262999	1.29E-04	Down-regulated	heparan sulfate glucosamine 3-O-sulfotransferase 6
LOC100644415	0.287781537	1.29E-04	Up-regulated	syntenin-1
LOC100645507	1.633855871	1.30E-04	Up-regulated	uncharacterized LOC100645507
LOC100643864	-0.371969034	1.33E-04	Down-regulated	uncharacterized LOC100643864
LOC100646833	1.115240401	1.33E-04	Up-regulated	histone demethylase UTY
LOC100650024	-0.221161947	1.33E-04	Down-regulated	ceramide transfer protein
LOC100647965	0.937775735	1.34E-04	Up-regulated	monocarboxylate transporter 12-B
LOC105666352	1.56101945	1.34E-04	Up-regulated	nuclease SbcCD subunit C
LOC100646220	1.356768032	1.35E-04	Up-regulated	dual oxidase maturation factor 1
LOC100647519	-0.408701343	1.36E-04	Down-regulated	TM2 domain-containing protein CG11103
LOC105666930	0.339244361	1.36E-04	Up-regulated	vacuolar-sorting protein SNF8
LOC100649894	-0.247551219	1.38E-04	Down-regulated	tetratricopeptide repeat protein 7B
LOC100643955	1.485192415	1.38E-04	Up-regulated	Usher syndrome type-1G protein homolog
LOC100648707	0.245216398	1.38E-04	Up-regulated	vacuolar protein sorting-associated protein 28 homolog
LOC110120215	-0.951814377	1.39E-04	Down-regulated	uncharacterized LOC110120215
LOC100643045	0.337281397	1.41E-04	Up-regulated	rhythmically expressed gene 2 protein
LOC100651573	-0.583923279	1.41E-04	Down-regulated	adenylate cyclase type 3
LOC100645567	2.786601834	1.41E-04	Up-regulated	UDP-glucosyltransferase 2
LOC100648732	2.172420175	1.42E-04	Up-regulated	aquaporin AQPcic
LOC100651225	0.556082512	1.44E-04	Up-regulated	coronin-1C-A
LOC100644275	0.221950704	1.44E-04	Up-regulated	TBC1 domain family member 15
LOC100642895	-0.378842687	1.49E-04	Down-regulated	glutamine-dependent NAD(+) synthetase
LOC100648599	0.69575387	1.49E-04	Up-regulated	proton-coupled amino acid transporter 1
LOC100644273	0.271879767	1.51E-04	Up-regulated	protein RRP5 homolog
LOC100648128	1.495068151	1.51E-04	Up-regulated	uncharacterized LOC100648128
LOC100646908	0.326878921	1.51E-04	Up-regulated	ataxin-3
LOC100642912	0.733699749	1.52E-04	Up-regulated	probable phospholipid hydroperoxide glutathione peroxidase
LOC100643814	-0.312782224	1.52E-04	Down-regulated	drebrin-like protein
LOC100645630	-0.819573229	1.52E-04	Down-regulated	RISC-loading complex subunit TARBP2
LOC105666719	0.352846021	1.53E-04	Up-regulated	soma ferritin
LOC100647594	0.266165734	1.53E-04	Up-regulated	serine/threonine-protein kinase/endoribonuclease IRE1
LOC100645121	-0.517699994	1.53E-04	Down-regulated	probable G-protein coupled receptor Mth-like 5
LOC100643752	1.23946966	1.54E-04	Up-regulated	histone H2A-like
LOC100646227	2.794154351	1.54E-04	Up-regulated	peroxidase
LOC100650209	2.194423447	1.54E-04	Up-regulated	photoreceptor-specific nuclear receptor
LOC100647264	0.391386197	1.55E-04	Up-regulated	paraplegin
LOC100649461	-0.176121169	1.56E-04	Down-regulated	myeloid leukemia factor
LOC100644884	-0.975323836	1.64E-04	Down-regulated	uncharacterized LOC100644884
LOC100647848	0.31558168	1.64E-04	Up-regulated	putative E3 ubiquitin-protein ligase UBR7
LOC100648396	-0.473441528	1.65E-04	Down-regulated	uncharacterized LOC100648396
LOC100644029	0.246993571	1.65E-04	Up-regulated	DENN domain-containing protein Crag
LOC100643970	-0.361238523	1.66E-04	Down-regulated	SAC3 domain-containing protein 1
LOC100651347	-0.416448571	1.67E-04	Down-regulated	cyclin-G-associated kinase
LOC105667204	0.370051731	1.67E-04	Up-regulated	pyruvate kinase
LOC100646532	-0.370393544	1.67E-04	Down-regulated	TATA box-binding protein-like 1
LOC100646163	-0.249262739	1.67E-04	Down-regulated	zinc finger CCCH domain-containing protein 10
LOC100648836	0.357565119	1.67E-04	Up-regulated	uncharacterized LOC100648836
LOC100650193	0.638531517	1.67E-04	Up-regulated	FMRFamide receptor
LOC100651676	0.238665877	1.67E-04	Up-regulated	E3 ubiquitin-protein ligase RNF38
LOC100647423	-0.321205587	1.71E-04	Down-regulated	transmembrane protein 164
LOC100643327	1.51244296	1.72E-04	Up-regulated	solute carrier family 26 member 6
LOC100644925	0.476710387	1.72E-04	Up-regulated	ribosomal L1 domain-containing protein 1
LOC100648970	-0.843648969	1.72E-04	Down-regulated	uncharacterized LOC100648970
LOC100651813	-0.532496824	1.72E-04	Down-regulated	solute carrier family 25 member 35
LOC100643214	0.792204452	1.73E-04	Up-regulated	4-coumarate--CoA ligase 1
LOC100647616	-0.671598928	1.73E-04	Down-regulated	PAX-interacting protein 1
LOC105665676	-0.68113003	1.74E-04	Down-regulated	chaoptin
LOC100646049	0.261238415	1.74E-04	Up-regulated	uncharacterized LOC100646049
LOC100643129	2.079355307	1.75E-04	Up-regulated	fork head domain-containing protein FD4
LOC100644572	0.470571419	1.75E-04	Up-regulated	chloride channel CLIC-like protein 1
LOC100650355	0.34208807	1.75E-04	Up-regulated	host cell factor
LOC100647765	1.851664997	1.77E-04	Up-regulated	toll-like receptor 3
LOC100646094	-0.640434001	1.77E-04	Down-regulated	peptide methionine sulfoxide reductase
LOC100643589	1.680038583	1.77E-04	Up-regulated	troponin T
LOC100652261	-0.271360247	1.82E-04	Down-regulated	sorting nexin-7-like
LOC100645837	-0.59243144	1.83E-04	Down-regulated	synaptic vesicle glycoprotein 2A
LOC100652243	-0.377775293	1.85E-04	Down-regulated	innexin inx2
LOC100651910	1.452577433	1.86E-04	Up-regulated	chitin synthase chs-2
LOC100651188	-0.276537642	1.90E-04	Down-regulated	short coiled-coil protein B
LOC100649117	0.466524981	1.91E-04	Up-regulated	E3 ubiquitin-protein ligase parkin

LOC100644098	-0.660709774	1.92E-04	Down-regulated	transcription factor Sox-21-A-like	
LOC100651143	0.388455176	1.93E-04	Up-regulated	phosphoglycerate mutase 2	
LOC100644463	1.329495875	1.94E-04	Up-regulated	4-hydroxybutyrate coenzyme A transferase	
LOC110119150	0.847207016	1.94E-04	Up-regulated	sperm-associated antigen 6-like	
LOC100647479	0.20504202	1.98E-04	Up-regulated	uncharacterized protein DDB_G0287625	
LOC100649539	-0.330927365	1.98E-04	Down-regulated	uncharacterized LOC100649539	
LOC100649562	-0.771137555	1.98E-04	Down-regulated	mismatch repair endonuclease PMS2	
LOC100651462	-0.501524951	1.98E-04	Down-regulated	guanine nucleotide-binding protein subunit gamma-e	
LOC105666438	0.803619349	1.98E-04	Up-regulated	uncharacterized LOC105666438	
LOC105666898	-0.565083102	1.98E-04	Down-regulated	pre-rRNA-processing protein TSR1 homolog	
LOC100651420	0.46680278	2.01E-04	Up-regulated	nuclear receptor-binding factor 2	
LOC100650573	1.574869221	2.01E-04	Up-regulated	endothelin-converting enzyme homolog	
LOC100649849	0.781449014	2.02E-04	Up-regulated	phosphotriesterase-related protein	
LOC100644731	-0.996747848	2.02E-04	Down-regulated	villin-like protein quail	
LOC100644525	0.293680081	2.03E-04	Up-regulated	arf-GAP domain and FG repeat-containing protein 1	
LOC100649975	0.762850116	2.04E-04	Up-regulated	uncharacterized LOC100649975	
LOC105666630	0.627297154	2.09E-04	Up-regulated	uncharacterized LOC105666630	
LOC100645810	0.481751012	2.09E-04	Up-regulated	telomere attrition and p53 response 1 protein	
LOC100645942	0.314735351	2.09E-04	Up-regulated	double-strand-break repair protein rad21 homolog	
LOC100646790	-0.44461494	2.09E-04	Down-regulated	protein windpipe	
LOC100643780	-0.513768023	2.10E-04	Down-regulated	lysosomal thioesterase PPT2 homolog	
LOC100648099	0.427444311	2.10E-04	Up-regulated	tudor domain-containing protein 1	
LOC105666120	0.280821365	2.10E-04	Up-regulated	ferritin subunit	
LOC100647962	0.539616869	2.14E-04	Up-regulated	DDB1- and CUL4-associated factor 6	
LOC100648748	-0.339199835	2.14E-04	Down-regulated	sodium- and chloride-dependent GABA transporter 1	
LOC100645416	0.298723261	2.15E-04	Up-regulated	uncharacterized LOC100645416	
LOC100642983	1.029437349	2.16E-04	Up-regulated	protein bicaudal C homolog 1-B	
LOC100643710	0.552936509	2.16E-04	Up-regulated	stabilizer of axonemal microtubules 1	
LOC100645898	1.318182506	2.16E-04	Up-regulated	T-box transcription factor TBX10	
LOC100651920	1.132451258	2.16E-04	Up-regulated	uncharacterized LOC100651920	
LOC100643335	-0.290397028	2.17E-04	Down-regulated	acid phosphatase type 7	
LOC100645826	0.824030047	2.21E-04	Up-regulated	uncharacterized LOC100645826	
LOC100645102	0.36156997	2.24E-04	Up-regulated	uncharacterized LOC100645102	
LOC100648030	0.23052426	2.27E-04	Up-regulated	heterogeneous nuclear ribonucleoprotein U	
LOC100647028	0.329817312	2.27E-04	Up-regulated	hamartin	
LOC100652206	0.902369219	2.28E-04	Up-regulated	elongation of very long chain fatty acids protein	
LOC100646799	-0.584011481	2.29E-04	Down-regulated	ankyrin repeat and death domain-containing protein 1A	
LOC100645988	-0.679410813	2.30E-04	Down-regulated	putative mediator of RNA polymerase II transcription subunit 26	
LOC100651051	0.27527884	2.33E-04	Up-regulated	isocitrate dehydrogenase [NAD] subunit gamma	
LOC100647709	3.078406723	2.34E-04	Up-regulated	uncharacterized LOC100647709	
LOC100645868	0.32697755	2.35E-04	Up-regulated	putative leucine-rich repeat-containing protein DDB_G0290503	
LOC100652123	-0.343751782	2.35E-04	Down-regulated	guanine nucleotide exchange factor DBS	
LOC100652167	-0.443537628	2.36E-04	Down-regulated	monocarboxylate transporter 10	
LOC100649108	0.368049889	2.37E-04	Up-regulated	stromal interaction molecule homolog	
LOC100650544	-0.217177013	2.37E-04	Down-regulated	uncharacterized LOC100650544	
LOC100649217	0.2771072	2.37E-04	Up-regulated	cytochrome c oxidase subunit 6b-3	
LOC100647153	-0.597810758	2.37E-04	Down-regulated	persulfide dioxygenase ETHE1	
LOC100650778	0.34858062	2.40E-04	Up-regulated	immunoglobulin-binding protein 1	
LOC100646722	-0.338917062	2.41E-04	Down-regulated	obg-like ATPase 1	
LOC100644626	0.697316385	2.44E-04	Up-regulated	40S ribosomal protein S28	
LOC100645203	2.606224737	2.48E-04	Up-regulated	uncharacterized LOC100645203	
LOC100651128	0.855627019	2.48E-04	Up-regulated	uncharacterized LOC100651128	
LOC100642755	0.180292863	2.49E-04	Up-regulated	N-terminal kinase-like protein	
LOC100643012	0.677586732	2.49E-04	Up-regulated	serine/threonine-protein kinase fused	
LOC100649667	-0.536735466	2.50E-04	Down-regulated	protein big brother	
LOC100647858	-0.736149788	2.51E-04	Down-regulated	trichohyalin	
LOC105666871	-0.626752309	2.51E-04	Down-regulated	thyrotropin-releasing hormone receptor	
LOC100643441	0.416241742	2.53E-04	Up-regulated	lipoyltransferase 1	
LOC100645459	-0.428899952	2.53E-04	Down-regulated	short-chain dehydrogenase/reductase family 16C member 6	
LOC100643975	0.574211726	2.54E-04	Up-regulated	vitamin K-dependent protein C	
LOC100642758	0.278918237	2.54E-04	Up-regulated	FHIP family protein AAEL005291	
LOC100648025	-1.455545587	2.58E-04	Down-regulated	mitochondrial pyruvate carrier 2	
LOC100650918	0.638399167	2.60E-04	Up-regulated	mpv17-like protein	
LOC100650951	3.049120263	2.63E-04	Up-regulated	uncharacterized LOC100650951	
LOC100651664	0.512848317	2.66E-04	Up-regulated	SUZ domain-containing protein 1	
LOC100651530	-1.116036185	2.67E-04	Down-regulated	uncharacterized LOC100651530	
LOC100649788	0.45812715	2.68E-04	Up-regulated	DNA replication licensing factor MCM4	
LOC100652192	0.671813681	2.70E-04	Up-regulated	N-glycosylase/DNA lyase	
LOC105667135	0.205097367	2.73E-04	Up-regulated	E3 ubiquitin-protein ligase RNF185	
LOC100650135	-0.983933325	2.74E-04	Down-regulated	uncharacterized LOC100650135	
LOC100649708	-0.480943194	2.74E-04	Down-regulated	serine-enriched protein	
LOC100650166	0.455561889	2.74E-04	Up-regulated		1
LOC100644217	1.370470902	2.82E-04	Up-regulated	odorant receptor 9a	
LOC100649733	0.285599536	2.82E-04	Up-regulated	negative elongation factor E	
LOC100644265	-0.646938028	2.82E-04	Down-regulated	succinate dehydrogenase assembly factor 3	
LOC100648395	0.288948541	2.83E-04	Up-regulated	pyruvate dehydrogenase [acetyl-transferring]-phosphatase 1	
LOC100650372	1.947334735	2.87E-04	Up-regulated	endothelin-converting enzyme 1	
LOC100652258	-0.641991389	2.87E-04	Down-regulated	aldo-keto reductase family 1 member B1	
LOC100643165	-0.654531226	2.87E-04	Down-regulated	cell adhesion molecule 2	
LOC100646801	0.249781519	2.91E-04	Up-regulated	TATA element modulatory factor	
LOC100647437	-0.602589225	2.91E-04	Down-regulated	neurotactin	

LOC100646476	-0.699079699	2.92E-04	Down-regulated	LIM/homeobox protein Lhx9
LOC100647982	0.624718366	2.94E-04	Up-regulated	chaperone protein DnaJ
LOC100642708	0.391205463	2.95E-04	Up-regulated	holocytochrome c-type synthase
LOC100647673	-0.55346252	2.95E-04	Down-regulated	insulin-like growth factor-binding protein complex acid labile subunit
LOC100651195	-0.380741187	2.95E-04	Down-regulated	uncharacterized LOC100651195
LOC100643262	2.003451352	2.96E-04	Up-regulated	glycine-rich cell wall structural protein
LOC100643798	0.209442985	2.97E-04	Up-regulated	prosaposin
LOC100650477	-0.553641896	2.97E-04	Down-regulated	calcyclin-binding protein
LOC100645125	-1.018798873	2.98E-04	Down-regulated	uncharacterized LOC100645125
LOC100651797	-0.444608471	2.98E-04	Down-regulated	protein shifted
LOC100646403	-0.36200214	2.99E-04	Down-regulated	probable G-protein coupled receptor B0563.6
LOC100647218	-0.598011206	3.04E-04	Down-regulated	platelet endothelial aggregation receptor 1
LOC100642999	-0.587986308	3.06E-04	Down-regulated	microprocessor complex subunit DGCR8
LOC100651648	1.121095563	3.07E-04	Up-regulated	probable 2-oxoglutarate dehydrogenase E1 component DHKTD1
LOC100644469	0.273542289	3.11E-04	Up-regulated	coiled-coil domain-containing protein 50
LOC100642602	-0.432574176	3.11E-04	Down-regulated	inorganic pyrophosphatase
LOC100651716	0.493765952	3.12E-04	Up-regulated	protein toll
LOC100647187	-0.481045586	3.14E-04	Down-regulated	kinesin-like protein costa
LOC100643324	1.31229964	3.17E-04	Up-regulated	nose resistant to fluoxetine protein 6
LOC100650566	0.940011331	3.19E-04	Up-regulated	protein henna
LOC105667024	0.410329057	3.19E-04	Up-regulated	glutathione S-transferase D7
LOC100644232	1.503606404	3.26E-04	Up-regulated	uncharacterized LOC100644232
LOC100647625	-0.517224417	3.26E-04	Down-regulated	protein disabled
LOC100643636	-0.460420588	3.26E-04	Down-regulated	bestrophin-2
LOC100645442	1.062396828	3.26E-04	Up-regulated	calmodulin-lysine N-methyltransferase
LOC100643188	-0.819606623	3.28E-04	Down-regulated	glycine receptor subunit alpha-3
LOC100643662	-0.342753144	3.32E-04	Down-regulated	transcription initiation factor TFIID subunit 6
LOC100645595	1.405762359	3.32E-04	Up-regulated	retinoid-inducible serine carboxypeptidase
LOC100644414	-0.376494739	3.33E-04	Down-regulated	uncharacterized LOC100644414
LOC100651214	0.755950358	3.35E-04	Up-regulated	probable chitinase 10
LOC100646524	-0.55102189	3.36E-04	Down-regulated	uncharacterized LOC100646524
LOC100649182	0.40544082	3.37E-04	Up-regulated	adenylyl cyclase-associated protein 2
LOC100650782	-0.417792822	3.39E-04	Down-regulated	multiple coagulation factor deficiency protein 2 homolog
LOC100644259	-0.462635416	3.43E-04	Down-regulated	LDLR chaperone boca
LOC105666872	0.487916988	3.48E-04	Up-regulated	centrosomal protein of 135 kDa
LOC100650998	-0.461330182	3.52E-04	Down-regulated	uncharacterized LOC100650998
LOC100642754	0.493063638	3.53E-04	Up-regulated	protein yippee-like 1
LOC100642819	-0.220096029	3.53E-04	Down-regulated	probable ATP-dependent RNA helicase DDX17
LOC100647077	-0.167272792	3.53E-04	Down-regulated	protein shuttle craft
LOC100649561	0.533125834	3.53E-04	Up-regulated	laminin subunit alpha-1
LOC100647727	0.953843149	3.56E-04	Up-regulated	uncharacterized LOC100647727
LOC100646955	-0.268094574	3.62E-04	Down-regulated	A-kinase anchor protein 17A
LOC100643514	-0.79216547	3.66E-04	Down-regulated	ejaculatory bulb-specific protein 3
LOC100650316	-0.501741977	3.70E-04	Down-regulated	endoribonuclease Dicer
LOC100651907	0.174648904	3.71E-04	Up-regulated	PHD finger protein 20
LOC100644302	-0.374882583	3.78E-04	Down-regulated	phosphorylase b kinase gamma catalytic chain
LOC100644391	-0.665925133	3.81E-04	Down-regulated	uncharacterized LOC100644391
LOC105665832	-0.443399033	3.81E-04	Down-regulated	lachesin
LOC110120257	0.509556075	3.83E-04	Up-regulated	putative uncharacterized transmembrane protein DDB_G0285049
LOC100644090	-0.409494556	3.84E-04	Down-regulated	mediator of RNA polymerase II transcription subunit 4
LOC100650319	0.738480927	3.84E-04	Up-regulated	dual specificity protein phosphatase 10
LOC100644317	0.310705054	3.86E-04	Up-regulated	39S ribosomal protein L51
LOC100646400	1.123485139	3.88E-04	Up-regulated	ionotropic receptor 25a
LOC105665615	0.412628695	3.92E-04	Up-regulated	DNA repair protein REV1
LOC100650977	-0.462724143	3.96E-04	Down-regulated	cyclin-dependent kinase 4
LOC105666482	-0.623826745	3.96E-04	Down-regulated	CTD small phosphatase-like protein 2
LOC100644148	-0.377400401	3.97E-04	Down-regulated	allatostatin-A receptor
LOC100646298	-0.256303177	4.06E-04	Down-regulated	guanine nucleotide-binding protein subunit beta-5
LOC105666838	-0.537642241	4.06E-04	Down-regulated	titin homolog
LOC100644345	-0.791918411	4.08E-04	Down-regulated	semaphorin-5A
LOC100646351	0.259080808	4.09E-04	Up-regulated	diphthine--ammonia ligase
LOC100649169	-0.320941465	4.11E-04	Down-regulated	protein saal1
LOC100647116	1.546963475	4.13E-04	Up-regulated	aminomethyltransferase
LOC100647507	1.143378519	4.25E-04	Up-regulated	arginase
LOC100644719	0.374345051	4.30E-04	Up-regulated	DNA polymerase alpha subunit B
LOC100648686	-0.327208736	4.33E-04	Down-regulated	galactosylgalactosylxylosylprotein 3-beta-glucuronosyltransferase P
LOC100647940	-0.548199788	4.33E-04	Down-regulated	5-hydroxytryptamine receptor
LOC100650089	-0.33887492	4.39E-04	Down-regulated	zinc finger protein 271
LOC100642957	-0.480780666	4.39E-04	Down-regulated	transmembrane protein 8B
LOC100651503	0.545793817	4.39E-04	Up-regulated	prefoldin subunit 6
LOC105665752	-0.591435575	4.39E-04	Down-regulated	uncharacterized LOC105665752
LOC100650112	-0.464947983	4.41E-04	Down-regulated	polypeptide N-acetylgalactosaminyltransferase 13
LOC100642477	-0.386036589	4.42E-04	Down-regulated	tubulin-specific chaperone D
LOC100648372	-0.384401974	4.46E-04	Down-regulated	vesicle-fusing ATPase 1
LOC100646433	-0.572696922	4.47E-04	Down-regulated	ATPase WRNIP1
LOC100645930	-0.483393642	4.49E-04	Down-regulated	protein qui-1
LOC100649997	0.567461239	4.49E-04	Up-regulated	probable medium-chain specific acyl-CoA dehydrogenase
LOC100647756	-0.431528751	4.49E-04	Down-regulated	transmembrane protein 53
LOC100644981	-0.812462997	4.53E-04	Down-regulated	CUGBP Elav-like family member 4
LOC100647476	-0.440816667	4.54E-04	Down-regulated	cytochrome c oxidase copper chaperone
LOC100643103	1.557369359	4.56E-04	Up-regulated	homeotic protein proboscipedia

LOC100647614	-0.341309531	4.56E-04	Down-regulated	lysosomal acid phosphatase
LOC100648008	-0.240912583	4.56E-04	Down-regulated	trehalase
LOC100649859	-0.476325227	4.56E-04	Down-regulated	copper homeostasis protein cutC homolog
LOC105665840	0.30061824	4.56E-04	Up-regulated	protein ABHD18
LOC100645210	-0.749698574	4.57E-04	Down-regulated	slit homolog 3 protein
LOC100644703	-0.476709138	4.59E-04	Down-regulated	gamma-aminobutyric acid receptor subunit beta-like
LOC100648714	0.288090811	4.64E-04	Up-regulated	protein FAM8A1
LOC100646189	-0.503990795	4.67E-04	Down-regulated	transformer-2 protein homolog beta
LOC100648727	-0.319808402	4.74E-04	Down-regulated	uncharacterized LOC100648727
LOC100649414	-0.511226289	4.74E-04	Down-regulated	Bardet-Biedl syndrome 2 protein homolog
LOC100647608	-0.685459494	4.82E-04	Down-regulated	uncharacterized LOC100647608
LOC105666853	-0.771789889	4.88E-04	Down-regulated	probable ATP-dependent RNA helicase ddx42
LOC100646626	-0.424481505	4.88E-04	Down-regulated	14-3-3 protein epsilon
LOC100645441	0.374810767	4.89E-04	Up-regulated	eukaryotic translation initiation factor 1A
LOC100644478	-0.215525319	4.95E-04	Down-regulated	NAD-dependent protein deacetylase Sirt6
LOC105666409	-0.586952533	4.96E-04	Down-regulated	adenosine 5'-monophosphoramidase HINT1
LOC100647659	-0.491845526	5.08E-04	Down-regulated	cysteine and histidine-rich protein 1 homolog
LOC100649519	-0.339944261	5.19E-04	Down-regulated	uncharacterized LOC100649519
LOC100646259	0.71412699	5.21E-04	Up-regulated	trichohyalin
LOC100644048	0.225168554	5.24E-04	Up-regulated	heat shock 70 kDa protein cognate 5
LOC100646556	0.319858583	5.32E-04	Up-regulated	rac GTPase-activating protein 1
LOC100645512	1.624410108	5.32E-04	Up-regulated	late histone H1
LOC100646893	-0.403980778	5.32E-04	Down-regulated	heat shock 70 kDa protein 4
LOC100643713	-0.34918255	5.35E-04	Down-regulated	microtubule-associated protein 4
LOC100642651	-0.288799742	5.38E-04	Down-regulated	protein spire
LOC100651377	-0.370746824	5.38E-04	Down-regulated	exportin-5
LOC100650429	-0.227112209	5.50E-04	Down-regulated	spliceosome-associated protein CWC15 homolog
LOC100648193	0.20539809	5.52E-04	Up-regulated	serine/threonine-protein kinase Pink1
LOC100648070	-0.811208386	5.54E-04	Down-regulated	enhancer of split mbeta protein-like
LOC100644590	0.357283411	5.62E-04	Up-regulated	uncharacterized LOC100644590
LOC100649187	0.301572589	5.62E-04	Up-regulated	E3 ubiquitin-protein ligase znrf2
LOC100646566	1.085258301	5.68E-04	Up-regulated	drebrin-like protein
LOC100643699	-0.317475187	5.72E-04	Down-regulated	arylalkylamine N-acetyltransferase 1
LOC100646760	0.528702559	5.73E-04	Up-regulated	uncharacterized LOC100646760
LOC100642951	-0.667294246	5.77E-04	Down-regulated	solute carrier family 35 member F2
LOC100651400	-0.487296431	5.77E-04	Down-regulated	L-2-hydroxyglutarate dehydrogenase
LOC105667137	2.07323315	5.77E-04	Up-regulated	cGMP-dependent protein kinase 1
LOC100645373	0.30675552	0.000579733	Up-regulated	actin-interacting protein 1
LOC100645554	0.791716428	5.80E-04	Up-regulated	heparan sulfate glucosamine 3-O-sulfotransferase 5
LOC100649824	-0.73379372	5.83E-04	Down-regulated	arrestin domain-containing protein 17
LOC100650981	0.437923942	5.83E-04	Up-regulated	uncharacterized LOC100650981
LOC105666798	-0.731604404	5.83E-04	Down-regulated	uncharacterized LOC105666798
LOC100651714	0.290709597	0.000587097	Up-regulated	dnaJ homolog dnj-5
LOC100651510	-0.443208756	5.92E-04	Down-regulated	GTP-binding protein 128up
LOC100646377	-0.394078377	5.94E-04	Down-regulated	uncharacterized LOC100646377
LOC100651613	-0.453407037	5.94E-04	Down-regulated	pre-piRNA 3'-exonuclease trimmer
LOC100651873	0.635022081	5.96E-04	Up-regulated	RNA-binding protein Rsf1
LOC100643178	-0.595046425	5.98E-04	Down-regulated	epsilon-sarcoglycan
LOC100643775	-0.401459682	5.98E-04	Down-regulated	15-hydroxyprostaglandin dehydrogenase [NAD(+)]
LOC100650366	-0.507706881	5.98E-04	Down-regulated	xanthine dehydrogenase
LOC105666517	0.895398007	5.98E-04	Up-regulated	uncharacterized LOC105666517
LOC100642713	-0.684387747	5.98E-04	Down-regulated	peptidyl-prolyl cis-trans isomerase FKBP4
LOC100650376	-0.299043081	6.02E-04	Down-regulated	ubiquitin carboxyl-terminal hydrolase 22
LOC100646057	-0.73128413	6.08E-04	Down-regulated	uncharacterized LOC100646057
LOC100647468	-0.573077039	6.29E-04	Down-regulated	extracellular sulfatase SULF-1 homolog
LOC100649739	-0.363823823	6.29E-04	Down-regulated	glutamate--cysteine ligase catalytic subunit
LOC100645357	0.33994859	6.30E-04	Up-regulated	nischarin
LOC100647483	0.518881597	6.32E-04	Up-regulated	A-kinase anchor protein 200
LOC100631090	-0.259700896	6.37E-04	Down-regulated	histone 3
LOC100646902	0.174166447	6.37E-04	Up-regulated	zinc finger protein-like 1
LOC105667110	1.244035008	6.37E-04	Up-regulated	organic cation transporter protein
LOC100649351	-0.836443895	6.43E-04	Down-regulated	transcription factor Sox-10
LOC105666713	0.712085788	6.52E-04	Up-regulated	uncharacterized LOC105666713
LOC100645004	0.31193222	6.57E-04	Up-regulated	mitochondrial import receptor subunit TOM70
LOC100651509	2.417934862	6.62E-04	Up-regulated	putative fatty acyl-CoA reductase CG5065
LOC105666300	-0.68078048	6.66E-04	Down-regulated	COA8 family protein CG14806
LOC100651412	-0.296516317	6.66E-04	Down-regulated	uncharacterized LOC100651412
LOC100645882	0.190489808	6.76E-04	Up-regulated	probable tRNA (uracil-O(2)-)-methyltransferase
LOC100643485	1.071129394	6.81E-04	Up-regulated	zinc finger protein 668
LOC100646729	3.041802935	6.82E-04	Up-regulated	fatty acyl-CoA reductase 1-like
LOC100651314	-0.31888766	6.82E-04	Down-regulated	ras-related protein Rab-30
LOC100652248	-0.474894144	6.82E-04	Down-regulated	uncharacterized LOC100652248
LOC105665784	0.210824762	6.82E-04	Up-regulated	zinc finger protein 420
LOC110120360	1.173755686	6.82E-04	Up-regulated	uncharacterized LOC110120360
LOC100649903	0.380508888	6.85E-04	Up-regulated	WD repeat-containing protein 43
LOC100643538	-0.235680935	6.97E-04	Down-regulated	cytochrome c1
LOC100649596	0.347330986	7.01E-04	Up-regulated	MICOS complex subunit MIC19
LOC100646933	-0.764336706	7.15E-04	Down-regulated	uncharacterized LOC100646933
LOC105666272	0.740687116	7.18E-04	Up-regulated	piggyBac transposable element-derived protein 4-like
LOC100644825	0.263577383	7.39E-04	Up-regulated	probable peroxisomal acyl-coenzyme A oxidase 1
LOC105666575	-0.562852878	7.45E-04	Down-regulated	isoaspartyl peptidase/L-asparaginase

LOC100644324	-0.195701062	7.49E-04	Down-regulated	sorting nexin-12
LOC100643782	-0.668022855	7.61E-04	Down-regulated	uncharacterized LOC100643782
LOC100650052	0.376148333	7.61E-04	Up-regulated	kanadaptin
LOC100645624	0.487614146	7.64E-04	Up-regulated	geminin
LOC100644174	0.791019758	7.68E-04	Up-regulated	flocculation protein FLO11
LOC100651423	-0.271073546	7.70E-04	Down-regulated	cystinosin homolog
LOC100644595	0.336853612	7.70E-04	Up-regulated	protein Pixie
LOC100649555	-0.643114276	7.70E-04	Down-regulated	protein ABHD11
LOC100647575	0.378185983	7.70E-04	Up-regulated	RING-type E3 ubiquitin-protein ligase PPIL2
LOC100648445	-0.276493717	7.70E-04	Down-regulated	B-cell CLL/lymphoma 7 protein family member B
LOC105666565	0.213608881	7.71E-04	Up-regulated	nucleoplasmin-like protein
LOC100642535	-0.757927481	7.72E-04	Down-regulated	probable nuclear hormone receptor HR38
LOC100643856	-0.414719776	7.92E-04	Down-regulated	cysteine protease ATG4D
LOC100650898	-0.564906956	7.92E-04	Down-regulated	chondroitin sulfate synthase 1
LOC100643350	0.349631794	7.92E-04	Up-regulated	myotubularin-related protein 6
LOC100651213	-0.335262282	7.92E-04	Down-regulated	transmembrane emp24 domain-containing protein 5
LOC100644277	0.594161541	8.01E-04	Up-regulated	probable dolichyl pyrophosphate Glc1Man9GlcNAc2 alpha-1
LOC105666449	1.898796771	8.09E-04	Up-regulated	facilitated trehalose transporter Tret1-like
LOC100642529	-0.267411467	8.11E-04	Down-regulated	zinc finger protein 341
LOC100643601	0.664852637	8.12E-04	Up-regulated	uncharacterized LOC100643601
LOC100644519	0.307100489	8.15E-04	Up-regulated	RNA-binding protein 34
LOC100647208	2.861543543	8.15E-04	Up-regulated	putative fatty acyl-CoA reductase CG5065
LOC100651362	-0.511588177	8.15E-04	Down-regulated	calumenin-B
LOC100651921	-0.430389798	8.15E-04	Down-regulated	locomotion-related protein Hikaru genki
LOC100646031	0.310790425	8.18E-04	Up-regulated	transcription initiation factor TFIID subunit 9-like
LOC105666086	-0.482359978	8.22E-04	Down-regulated	PI-PLC X domain-containing protein 1
LOC100644254	0.363348944	8.26E-04	Up-regulated	nuclear pore complex protein Nup153
LOC100647699	1.233481828	8.29E-04	Up-regulated	two pore potassium channel protein sup-9
LOC100644008	0.377774424	8.35E-04	Up-regulated	uncharacterized LOC100644008
LOC100649516	0.861417292	8.44E-04	Up-regulated	uncharacterized protein YFR016C
LOC100649139	-0.303155697	8.44E-04	Down-regulated	alpha-tubulin N-acetyltransferase
LOC110120154	-0.717278542	8.44E-04	Down-regulated	uncharacterized LOC110120154
LOC100649120	0.954652558	8.46E-04	Up-regulated	protein Hook homolog 1
LOC100650715	-0.577255674	8.51E-04	Down-regulated	cathepsin O
LOC100645122	-0.556957785	8.56E-04	Down-regulated	coiled-coil and C2 domain-containing protein 2A
LOC100646568	-0.402065206	8.68E-04	Down-regulated	DNA-directed RNA polymerase III subunit RPC7-like
LOC100644748	0.89961903	8.72E-04	Up-regulated	relaxin receptor 1
LOC100642553	0.314952663	8.86E-04	Up-regulated	mitochondrial coenzyme A transporter SLC25A42
LOC100646573	-0.667138775	8.86E-04	Down-regulated	serine/threonine-protein kinase 32A
LOC100650843	-0.320090269	8.90E-04	Down-regulated	dosage compensation regulator
LOC100646435	0.271400995	9.10E-04	Up-regulated	nucleolar protein 11
LOC110120136	-0.353943067	9.15E-04	Down-regulated	piggyBac transposable element-derived protein 4
LOC100642305	-0.358904987	9.15E-04	Down-regulated	guanine nucleotide-binding protein G(o) subunit alpha
LOC100651448	-0.194408827	9.23E-04	Down-regulated	abl interactor 2
LOC100645225	0.591713021	9.24E-04	Up-regulated	60S acidic ribosomal protein P2
LOC100644592	2.343721121	9.40E-04	Up-regulated	uncharacterized LOC100644592
LOC100644092	-0.307485901	9.47E-04	Down-regulated	glycoprotein-N-acetylgalactosamine 3-beta-galactosyltransferase 1
LOC100647384	0.945565647	9.52E-04	Up-regulated	uncharacterized LOC100647384
LOC100642833	0.536184968	9.56E-04	Up-regulated	pleckstrin homology-like domain family B member 1
LOC100650563	-0.331204371	9.57E-04	Down-regulated	protein arginine N-methyltransferase 5
LOC100648663	1.640879431	9.62E-04	Up-regulated	YLP motif-containing protein 1-like
LOC100651124	2.965769867	9.73E-04	Up-regulated	uncharacterized LOC100651124
LOC110119351	-0.377336334	9.73E-04	Down-regulated	uncharacterized protein DDB_G0272718-like
LOC110119995	0.536156892	9.73E-04	Up-regulated	uncharacterized LOC110119995
LOC100651239	-0.528429377	9.75E-04	Down-regulated	apoptotic protease-activating factor 1
LOC100644311	-0.243041566	9.78E-04	Down-regulated	regulator of G-protein signaling 7
LOC100646270	0.84444255	9.78E-04	Up-regulated	maternal protein exuperantia
LOC100644067	0.311743543	9.83E-04	Up-regulated	serine/threonine-protein kinase RIO1
LOC100644744	0.280540561	9.92E-04	Up-regulated	ATP-binding cassette sub-family G member 1
LOC100650502	-0.395973125	9.93E-04	Down-regulated	cGMP-specific 3'
LOC100642192	0.276526561	9.95E-04	Up-regulated	uncharacterized LOC100642192
LOC100647494	0.251615789	9.95E-04	Up-regulated	CD109 antigen
LOC100643818	0.447934996	9.99E-04	Up-regulated	mRNA cap guanine-N7 methyltransferase
LOC100645470	1.320074579	0.001002273	Up-regulated	ABC transporter G family member 20
LOC100645780	0.348569468	0.001002273	Up-regulated	cathepsin L
LOC105666394	-0.595013172	0.001003874	Down-regulated	peptidoglycan recognition protein 3
LOC100643118	-0.274776144	0.001012274	Down-regulated	FACT complex subunit spt16
LOC100642888	-0.232061327	0.001023464	Down-regulated	nucleolin
LOC100645031	-0.231600253	0.00102348	Down-regulated	polypeptide N-acetylgalactosaminyltransferase 35A
LOC100644733	0.971752519	0.00102978	Up-regulated	uncharacterized LOC100644733
LOC100643272	-0.444973531	0.001030137	Down-regulated	HIG1 domain family member 1C
LOC100645621	0.508877476	0.001033627	Up-regulated	pyruvate dehydrogenase E1 component subunit beta
LOC100645649	0.411030693	0.001033627	Up-regulated	INO80 complex subunit C
LOC100643530	-0.821973651	0.001041857	Down-regulated	zinc finger and SCAN domain-containing protein 21
LOC100643801	0.839167484	0.001041857	Up-regulated	protein grainyhead
LOC100649904	0.510103033	0.00104471	Up-regulated	CD63 antigen
LOC100648478	1.000984611	0.001051488	Up-regulated	uncharacterized LOC100648478
LOC100649118	0.268569206	0.001053037	Up-regulated	serine/threonine-protein kinase 10
LOC100651475	-0.404224418	0.001061422	Down-regulated	uncharacterized LOC100651475
LOC100642322	0.761619177	0.001073417	Up-regulated	uncharacterized LOC100642322
LOC100646305	0.348488875	0.001073417	Up-regulated	protein FAM13B

LOC100646039	1.290260283	0.00107476	Up-regulated	uncharacterized LOC100646039
LOC100650013	-0.314047658	0.001085793	Down-regulated	uncharacterized LOC100650013
LOC100645879	2.157317222	0.001100847	Up-regulated	probable cytochrome P450 12a5 LOC100645879
LOC100648298	-0.483423719	0.001102502	Down-regulated	RING finger protein 207
LOC100644776	-0.487315601	0.001108293	Down-regulated	diacylglycerol lipase-alpha
LOC100646269	-0.361573735	0.001110256	Down-regulated	GMP synthase [glutamine-hydrolyzing]
LOC100645201	0.301783794	0.001110394	Up-regulated	RING finger protein 10
LOC105666829	-0.450514053	0.001110574	Down-regulated	5-hydroxytryptamine receptor 1
LOC100643281	0.278331236	0.001116115	Up-regulated	membrane-bound transcription factor site-1 protease
LOC100650072	-0.470623891	0.001117135	Down-regulated	centrosomal protein of 104 kDa
LOC110120229	-0.409315328	0.001120524	Down-regulated	vesicular acetylcholine transporter
LOC100645528	-0.667987645	0.001123599	Down-regulated	pinopsin
LOC100649850	-0.279526037	0.001133033	Down-regulated	uncharacterized LOC100649850
LOC100642677	-0.52660318	0.001133999	Down-regulated	protein tipE
LOC100650548	-0.449669524	0.00114118	Down-regulated	neuronal calcium sensor 2
LOC105666825	-0.391239091	0.00114118	Down-regulated	ras-like protein family member 10B
LOC100644765	0.563850428	0.001141344	Up-regulated	CUGBP Elav-like family member 2
LOC100643558	-0.759956466	0.001144003	Down-regulated	uncharacterized LOC100643558
LOC100644539	1.542634429	0.001144003	Up-regulated	uncharacterized LOC100644539
LOC100649082	0.340583034	0.001144003	Up-regulated	vesicle-associated membrane protein 7
LOC100646536	-0.446142702	0.001148041	Down-regulated	uncharacterized LOC100646536
LOC100645642	-0.641402196	0.001149105	Down-regulated	elongation of very long chain fatty acids protein 6
LOC100646637	0.364813796	0.001151722	Up-regulated	elongation of very long chain fatty acids protein 6
LOC100643684	0.865310496	0.001164535	Up-regulated	putative phospholipase B-like lamina ancestor
LOC100648936	-0.303044541	0.001169454	Down-regulated	uncharacterized LOC100648936
LOC100645186	0.400099811	0.001172183	Up-regulated	26S proteasome non-ATPase regulatory subunit 5
LOC100644276	0.587582307	0.001174446	Up-regulated	60S ribosomal protein L28
LOC100644432	0.68870233	0.001176082	Up-regulated	uncharacterized LOC100644432
LOC100647648	0.550193425	0.001184054	Up-regulated	trehalase
LOC100648493	-0.352901456	0.001184054	Down-regulated	neurofibromin
LOC100649714	1.004434245	0.001184164	Up-regulated	calcyphosin-like protein
LOC100652288	-0.204892202	0.001185254	Down-regulated	ADP-dependent glucokinase
LOC100651688	0.235207717	0.001188416	Up-regulated	activating transcription factor of chaperone
LOC100651218	0.34511179	0.001191402	Up-regulated	methionine aminopeptidase 1
LOC100648916	-0.409663768	0.001193569	Down-regulated	N-acetyl-D-glucosamine kinase
LOC100642438	1.776650481	0.001203858	Up-regulated	uncharacterized LOC100642438
LOC100645236	-0.324772433	0.001204353	Down-regulated	protein dispatched
LOC100647075	-0.285429633	0.001207783	Down-regulated	dnaJ homolog subfamily C member 16
LOC105665624	-0.406690642	0.001213981	Down-regulated	polycomb protein EED-like
LOC100647744	-0.222874998	0.001224116	Down-regulated	transcription initiation factor TFIID subunit 1
LOC100651979	0.538025383	0.001224116	Up-regulated	protein brambleberry
LOC100649123	-0.317138904	0.001225274	Down-regulated	sodium/potassium-transporting ATPase subunit beta-2
LOC100644618	1.6417626	0.001228117	Up-regulated	facilitated trehalose transporter Tret1
LOC100644599	-0.356276846	0.001241837	Down-regulated	endothelial zinc finger protein induced by tumor necrosis factor alpha
LOC100649909	0.227778933	0.001241837	Up-regulated	5'-3' exoribonuclease 1
LOC100645343	-0.470340571	0.001243535	Down-regulated	putative fatty acyl-CoA reductase CG5065
LOC100648412	0.408822711	0.001243563	Up-regulated	putative GPI-anchored protein pfl2
LOC105666885	-1.522179869	0.001243563	Down-regulated	uncharacterized LOC105666885
LOC100645072	0.276777205	0.001250121	Up-regulated	INO80 complex subunit B
LOC100643426	-0.522055123	0.001250763	Down-regulated	myb-like protein X
LOC100645582	-0.273761765	0.001271468	Down-regulated	diphosphoinositol polyphosphate phosphohydrolase 1
LOC100643112	-0.215114269	0.001271632	Down-regulated	neuropathy target esterase sws
LOC100645392	0.257949338	0.001272255	Up-regulated	vacuolar protein sorting-associated protein 53 homolog
LOC100649727	-0.644954588	0.001272878	Down-regulated	uncharacterized LOC100649727
LOC100651229	0.395856429	0.001272878	Up-regulated	uncharacterized LOC100651229
LOC100647604	-0.487698945	0.001275942	Down-regulated	mediator of RNA polymerase II transcription subunit 11
LOC100646357	-0.41447972	0.001277474	Down-regulated	GTP-binding protein 10 homolog
LOC100643356	0.458251599	0.001277609	Up-regulated	ubiquitin-conjugating enzyme E2 H
LOC100649412	-0.397201723	0.00128733	Down-regulated	paired box protein Pax-6
LOC100642820	-0.250036157	0.001288807	Down-regulated	wolframin
LOC105666020	2.279201748	0.001288807	Up-regulated	uncharacterized LOC105666020
LOC100645057	0.332182837	0.001288865	Up-regulated	something about silencing protein 10
LOC100650888	-0.463329308	0.001288865	Down-regulated	thioredoxin domain-containing protein
LOC100644831	-0.609316317	0.00130234	Down-regulated	asparagine synthetase [glutamine-hydrolyzing]
LOC100651729	0.715343688	0.001309905	Up-regulated	uncharacterized LOC100651729
LOC100649833	0.797550672	0.001323409	Up-regulated	formin-like protein
LOC100643555	0.405316713	0.001335703	Up-regulated	UBA-like domain-containing protein 2
LOC100644998	-0.686820662	0.001338285	Down-regulated	nose resistant to fluoxetine protein 6
LOC100644655	-0.375633123	0.001347987	Down-regulated	unconventional myosin-Va
LOC100648082	0.347558587	0.001347987	Up-regulated	exonuclease 1
LOC100651798	-0.300896761	0.001347987	Down-regulated	dihydrolipoyllysine-residue succinyltransferase of 2-oxoglutarate dehydrogenase
LOC100650850	-0.495695686	0.001355688	Down-regulated	tudor domain-containing protein 7
LOC100650406	-0.588329335	0.001357836	Down-regulated	leucine-rich repeat and immunoglobulin domain-containing nogo receptor
LOC100643827	0.44036855	0.001374617	Up-regulated	dual specificity protein phosphatase 3
LOC100649989	0.25812368	0.001374617	Up-regulated	probable tyrosyl-DNA phosphodiesterase
LOC100644387	-0.421826297	0.001381075	Down-regulated	inosine-5'-monophosphate dehydrogenase 1b
LOC100646238	-0.220594351	0.001396204	Down-regulated	transcription elongation factor B polypeptide 3
LOC100643945	-0.504179121	0.001408268	Down-regulated	syntaxin-1A
LOC100650022	0.243820569	0.001413643	Up-regulated	probable ATP-dependent RNA helicase kurz
LOC100644062	0.375725472	0.001415723	Up-regulated	eukaryotic translation initiation factor 2 subunit 1
LOC100644147	-0.529729762	0.001434256	Down-regulated	segmentation protein Runt

LOC100644939	-0.433656531	0.001442469	Down-regulated	uncharacterized LOC100644939
LOC100645362	-0.232358258	0.001442508	Down-regulated	uncharacterized LOC100645362
LOC100646128	-0.957762236	0.001451133	Down-regulated	homeobox protein Hmx
LOC110119144	-0.318744805	0.001451133	Down-regulated	zinc finger and BTB domain-containing protein 17
LOC100645615	-0.633695269	0.001455421	Down-regulated	serine-protein kinase ATM
LOC100651565	0.322444996	0.001465595	Up-regulated	60S ribosomal protein L10a
LOC100644486	-0.452400134	0.001466609	Down-regulated	insulin gene enhancer protein ISL-1
LOC100648766	-0.433132397	0.001472096	Down-regulated	basement membrane-specific heparan sulfate proteoglycan core protein
LOC100643729	0.492599086	0.00147574	Up-regulated	death-associated protein 1
LOC105666941	0.807381786	0.001482277	Up-regulated	leucine-rich repeat-containing protein 34
LOC100642621	0.26309923	0.001487339	Up-regulated	protein cereblon
LOC110120209	0.831492822	0.001497318	Up-regulated	uncharacterized LOC110120209
LOC100649134	-0.316689331	0.00149893	Down-regulated	uroporphyrinogen-III synthase
LOC100647106	0.238537948	0.00152558	Up-regulated	CCHC-type zinc finger nucleic acid binding protein
LOC105666009	-0.372468051	0.001540639	Down-regulated	coiled-coil domain-containing protein 112-like
LOC100643831	-0.418595264	0.001548306	Down-regulated	dynamin
LOC100647668	-0.561367613	0.001548306	Down-regulated	putative thiamine transporter SLC35F3
LOC100652181	0.263772549	0.001548306	Up-regulated	egalitarian protein homolog
LOC110119498	-0.334942537	0.001548306	Down-regulated	translation initiation factor IF-2-like
LOC100646468	-0.41008581	0.001575625	Down-regulated	zinc finger protein 189
LOC100651764	0.31188528	0.00157829	Up-regulated	2-oxoglutarate and iron-dependent oxygenase domain-containing protein 3
LOC100652254	0.359041794	0.001589717	Up-regulated	protein dj-1beta
LOC100646370	0.256388693	0.001592273	Up-regulated	zinc finger protein ZFP2
LOC100645006	-0.673802741	0.001599924	Down-regulated	uncharacterized LOC100645006
LOC105665972	-1.084140823	0.00160911	Down-regulated	glycine-rich cell wall structural protein
LOC100651961	0.313105676	0.001611879	Up-regulated	ADP-ribosylation factor 2
LOC100646583	-0.440290329	0.001614142	Down-regulated	multidrug resistance-associated protein 1
LOC100644249	2.077799589	0.001617105	Up-regulated	uncharacterized LOC100644249
LOC100650712	-0.252398897	0.00162259	Down-regulated	nuclear RNA export factor 1
LOC100651099	-0.384993988	0.00163079	Down-regulated	protein ARV1
LOC100631058	0.725952592	0.001643993	Up-regulated	small heat shock protein
LOC100645414	-0.683869348	0.001648978	Down-regulated	B-cell lymphoma/leukemia 11A
LOC100643577	1.366023388	0.001654663	Up-regulated	protein takeout
LOC100645224	1.242027493	0.001654663	Up-regulated	CD63 antigen
LOC100648257	1.031369821	0.001654663	Up-regulated	probable cytochrome P450 6a14 LOC100648257
LOC105666325	1.441794037	0.001654663	Up-regulated	protein lethal(2)essential for life
LOC100642234	-0.35336087	0.001655029	Down-regulated	zinc finger protein 543
LOC100643070	0.622463929	0.001655029	Up-regulated	ryanodine receptor
LOC100643300	0.470827678	0.001655029	Up-regulated	uncharacterized LOC100643300
LOC100644604	-0.526986285	0.001655029	Down-regulated	ankyrin repeat and BTB/POZ domain-containing protein BTBD11
LOC100644874	0.754785447	0.001658041	Up-regulated	uncharacterized protein CG43427
LOC100644319	-0.535574036	0.001677974	Down-regulated	potassium voltage-gated channel protein Shal
LOC100649897	0.629792351	0.001677974	Up-regulated	TBC1 domain family member 31
LOC100649863	0.259715081	0.001679924	Up-regulated	WW domain-containing oxidoreductase
LOC100642447	-0.541558022	0.001680327	Down-regulated	homeobox protein Nkx-2.4
LOC100651185	-0.439879132	0.001689947	Down-regulated	alpha-mannosidase 2
LOC100642889	0.268339841	0.001697534	Up-regulated	ubiquitin domain-containing protein UBFD1
LOC100644635	-0.601285683	0.001697534	Down-regulated	chorion peroxidase
LOC100648867	-0.766430163	0.001697534	Down-regulated	uncharacterized LOC100648867
LOC110120086	1.349326337	0.001697534	Up-regulated	uncharacterized LOC110120086
LOC100646541	0.280992024	0.001697597	Up-regulated	large subunit GTPase 1 homolog
LOC100645138	0.263570583	0.001699526	Up-regulated	SET and MYND domain-containing protein 4
LOC105666736	-0.307255682	0.001703726	Down-regulated	probable phosphatase phospho2
LOC100645912	-0.314362401	0.001713914	Down-regulated	DNA-directed RNA polymerase III subunit RPC8
LOC105665646	-0.359995827	0.001718253	Down-regulated	GPI mannosyltransferase 3
LOC100642521	0.525944791	0.0017264	Up-regulated	peroxisome biogenesis factor 10
LOC100648661	0.566334502	0.0017264	Up-regulated	slit homolog 1 protein
LOC100647189	-0.422075368	0.001732032	Down-regulated	oxysterol-binding protein-related protein 2
LOC100648676	-0.327641907	0.00173467	Down-regulated	uncharacterized LOC100648676
LOC100648246	-0.195333989	0.00173543	Down-regulated	target of rapamycin complex subunit lst8
LOC105666521	0.425649174	0.00173543	Up-regulated	uncharacterized LOC105666521
LOC100642190	0.312630614	0.001738272	Up-regulated	sorbin and SH3 domain-containing protein 1 homolog
LOC100643080	0.589548295	0.001738272	Up-regulated	adenylate kinase isoenzyme 1
LOC100645783	-0.344696752	0.001740692	Down-regulated	phosphatidylinositol-binding clathrin assembly protein LAP
LOC100643457	0.217730314	0.001741153	Up-regulated	RAC serine/threonine-protein kinase
LOC100647127	-0.697050708	0.001747293	Down-regulated	BTB/POZ domain-containing protein KCTD16
LOC100651084	-0.250022369	0.001747293	Down-regulated	gamma-1-syntrophin
LOC100651508	-0.391711718	0.001756245	Down-regulated	uncharacterized LOC100651508
LOC100642796	-0.571550144	0.00176041	Down-regulated	uncharacterized LOC100642796
LOC100647634	0.320663101	0.001793716	Up-regulated	probable U2 small nuclear ribonucleoprotein A'
LOC100646764	1.328119207	0.001817054	Up-regulated	replication factor C subunit 2
LOC105666961	1.387552728	0.001831502	Up-regulated	fatty acyl-CoA reductase 1-like
LOC100652073	-0.349280226	0.001832269	Down-regulated	uncharacterized LOC100652073
LOC105666026	1.264538244	0.001839858	Up-regulated	uncharacterized LOC105666026
LOC100648432	-0.453623305	0.001847647	Down-regulated	glutamine--fructose-6-phosphate aminotransferase [isomerizing] 2
LOC100643883	-0.319263731	0.001887572	Down-regulated	armadillo repeat-containing protein 5
LOC100644348	-0.394469223	0.001893614	Down-regulated	uncharacterized LOC100644348
LOC105665827	0.492094485	0.001904798	Up-regulated	uncharacterized LOC105665827
LOC100647789	0.295060294	0.001909971	Up-regulated	signal transducer and activator of transcription 5A
LOC100651711	0.405371794	0.00191162	Up-regulated	adenylyltransferase and sulfurtransferase MOCS3
LOC100646284	-0.194011555	0.001914074	Down-regulated	histidine--tRNA ligase

LOC100650491	-0.335715974	0.001921017	Down-regulated	pre-mRNA-processing factor 6
LOC100643060	-0.311355099	0.001923213	Down-regulated	inositol polyphosphate-5-phosphatase A
LOC100647322	1.542604237	0.001923213	Up-regulated	gustatory receptor for sugar taste 64f-like
LOC100643695	-0.434191234	0.001923703	Down-regulated	vesicular inhibitory amino acid transporter
LOC100648123	-0.262055187	0.001927046	Down-regulated	Golgi membrane protein 1
LOC100643331	0.459505553	0.001929655	Up-regulated	tropomodulin
LOC100650121	0.244265842	0.001931747	Up-regulated	serine/threonine-protein phosphatase 4 regulatory subunit 2
LOC100646656	1.669803882	0.001932062	Up-regulated	uncharacterized LOC100646656
LOC100644156	0.42149662	0.001933107	Up-regulated	S-adenosylmethionine sensor upstream of mTORC1
LOC100645587	0.323819801	0.001938557	Up-regulated	peptidyl-prolyl cis-trans isomerase H
LOC100647792	-0.268842429	0.001941256	Down-regulated	uncharacterized LOC100647792
LOC100651693	-0.499130899	0.001944428	Down-regulated	cytochrome P450 6B5 LOC100651693
LOC100648722	0.33340513	0.001945604	Up-regulated	negative elongation factor A
LOC100649744	1.500457767	0.001945604	Up-regulated	alpha-amylase
LOC105666363	1.079672715	0.001945604	Up-regulated	amine oxidase [flavin-containing]
LOC100643914	-0.225676356	0.001947275	Down-regulated	calcium-binding mitochondrial carrier protein Aralar1
LOC100643895	-0.379705854	0.001951368	Down-regulated	sorting nexin-27
LOC100646187	0.378074572	0.001953833	Up-regulated	2-oxoglutarate dehydrogenase
LOC100651083	-0.342960079	0.001971621	Down-regulated	uncharacterized LOC100651083
LOC100650042	1.329245498	0.001974027	Up-regulated	uncharacterized LOC100650042
LOC100651324	0.386333569	0.001984225	Up-regulated	1-acylglycerol-3-phosphate O-acyltransferase Pnpla3
LOC100648005	-0.171885833	0.001987249	Down-regulated	uncharacterized LOC100648005
LOC105667084	-0.754443581	0.001987249	Down-regulated	uncharacterized LOC105667084
LOC100642291	-0.33842529	0.002004368	Down-regulated	phosphatidylserine synthase
LOC100647376	-0.189891377	0.002009104	Down-regulated	peptidyl-prolyl cis-trans isomerase cyp11
LOC100643119	-0.229100763	0.002035564	Down-regulated	coiled-coil domain-containing protein 102A
LOC100651985	-0.698468773	0.002037406	Down-regulated	uncharacterized LOC100651985
LOC100646483	0.230070372	0.002044835	Up-regulated	E3 ubiquitin-protein ligase Topors
LOC100643243	-1.418398293	0.002074833	Down-regulated	dipeptidase 1
LOC100650168	0.26715978	0.002080832	Up-regulated	ubiquitin carboxyl-terminal hydrolase CYLD
LOC100648369	0.57115821	0.002089123	Up-regulated	uncharacterized LOC100648369
LOC100645804	-0.418863615	0.002123486	Down-regulated	prolyl 4-hydroxylase subunit alpha-1
LOC100643728	-0.351685507	0.002125918	Down-regulated	laccase-1
LOC100651053	-0.804744238	0.002132028	Down-regulated	hyaluronidase
LOC100646805	-0.544527707	0.002137478	Down-regulated	transmembrane protein 205
LOC100650736	-0.511328127	0.002137478	Down-regulated	metabotropic glutamate receptor
LOC100646070	0.48173045	0.002139462	Up-regulated	40S ribosomal protein S6
LOC100645708	-0.212125469	0.002160896	Down-regulated	dnaJ homolog subfamily A member 1
LOC100651277	-0.16668378	0.002164688	Down-regulated	3-oxoacyl-[acyl-carrier-protein] synthase
LOC100651131	0.307553562	0.00219839	Up-regulated	cytosolic Fe-S cluster assembly factor NUBP1 homolog
LOC100649101	0.201380823	0.002221754	Up-regulated	E3 ubiquitin-protein ligase ariadne-1
LOC100642975	-0.344746569	0.002222686	Down-regulated	bridging integrator 3
LOC100646555	-0.536068446	0.002222686	Down-regulated	EGF domain-specific O-linked N-acetylglucosamine transferase
LOC100644558	0.334714722	0.00222778	Up-regulated	ankyrin repeat domain-containing protein 40
LOC100651042	-0.232493304	0.002234839	Down-regulated	peptide-N(4)-(N-acetyl-beta-glucosaminyl)asparagine amidase
LOC100647620	0.561370368	0.002241378	Up-regulated	uncharacterized LOC100647620
LOC100644405	0.262920195	0.0022626	Up-regulated	flap endonuclease 1
LOC100646196	0.884370457	0.002271861	Up-regulated	diacylglycerol O-acyltransferase 1
LOC105666550	-0.356469262	0.002290654	Down-regulated	nucleoporin Nup43
LOC100649382	-0.671746131	0.002293491	Down-regulated	alpha-N-acetylglucosaminidase
LOC100642653	1.060286395	0.002302138	Up-regulated	scavenger receptor class B member 1
LOC100649972	0.341603899	0.002307922	Up-regulated	kinesin-like protein KIF23
LOC100643803	0.209801885	0.002309082	Up-regulated	eukaryotic initiation factor 4A-III
LOC100651500	1.924608884	0.002309473	Up-regulated	uncharacterized LOC100651500
LOC100643107	-0.327467205	0.002317653	Down-regulated	glycerol kinase
LOC100643638	-0.412377416	0.002319508	Down-regulated	uncharacterized LOC100643638
LOC100645819	-0.617976431	0.002331574	Down-regulated	heat shock protein 83
LOC100647087	0.41082136	0.002341672	Up-regulated	dystrobrevin beta
LOC100647581	-0.48476027	0.002341672	Down-regulated	iodotyrosine deiodinase 1
LOC100651896	-0.246653395	0.002341672	Down-regulated	protein PELPK1
LOC105666395	-0.536962422	0.002351176	Down-regulated	uncharacterized LOC105666395
LOC100652300	-0.291838578	0.002353811	Down-regulated	ankyrin repeat domain-containing protein 49
LOC100646957	0.314358847	0.002360719	Up-regulated	activating signal cointegrator 1 complex subunit 3
LOC105666547	-0.282145577	0.002369128	Down-regulated	aldo-keto reductase family 1 member B1
LOC100643432	0.233786693	0.002375539	Up-regulated	1-phosphatidylinositol 3-phosphate 5-kinase
LOC100646418	0.484367047	0.00238998	Up-regulated	leucine-rich melanocyte differentiation-associated protein
LOC100648842	-0.341932359	0.00238998	Down-regulated	serine/threonine-protein kinase PLK1
LOC100646538	0.265216006	0.002392352	Up-regulated	protein phosphatase 1 regulatory subunit 12A
LOC100644400	-0.289750541	0.002399176	Down-regulated	steroid hormone receptor ERR1
LOC100651593	0.297379255	0.002399176	Up-regulated	probable galactose-1-phosphate uridylyltransferase
LOC100648347	0.230292723	0.002421279	Up-regulated	zinc finger RNA-binding protein
LOC100649847	0.662704084	0.002426521	Up-regulated	NPC intracellular cholesterol transporter 2 homolog a
LOC100650719	-0.218723139	0.002428461	Down-regulated	protein vav
LOC100650913	0.377624001	0.002428811	Up-regulated	rRNA-processing protein FCF1 homolog
LOC100651396	-0.635769049	0.002437931	Down-regulated	allergen Tha p 1
LOC100643237	-0.227618634	0.00243868	Down-regulated	active breakpoint cluster region-related protein
LOC100645793	0.250120209	0.002439106	Up-regulated	lon protease homolog
LOC100642592	-0.595536977	0.002456294	Down-regulated	basic helix-loop-helix transcription factor amos
LOC100649811	-0.394184227	0.002461243	Down-regulated	plexin-B
LOC100645342	-0.582984955	0.002472357	Down-regulated	uncharacterized LOC100645342
LOC100642856	0.912506098	0.002497852	Up-regulated	endoribonuclease LACTB2

LOC100647509	0.295685176	0.002498164	Up-regulated	protein Smaug homolog 1
LOC100645013	1.018831536	0.002498582	Up-regulated	PI-PLC X domain-containing protein 1
LOC100650387	-0.50942522	0.002498582	Down-regulated	GTP-binding protein Di-Ras2
LOC100643688	0.352590459	0.002501871	Up-regulated	zinc finger protein 830
LOC100645134	-0.323081227	0.002501871	Down-regulated	transmembrane protein 19
LOC100650156	-0.347123143	0.002501871	Down-regulated	U1 small nuclear ribonucleoprotein 70 kDa
LOC100650349	-0.529245425	0.002501871	Down-regulated	immunoglobulin superfamily DCC subclass member 4
LOC100651089	0.442203761	0.002501871	Up-regulated	uncharacterized LOC100651089
LOC100651406	0.311305345	0.002522147	Up-regulated	E3 ubiquitin-protein ligase HUWE1
LOC100645091	-0.361704555	0.002528622	Down-regulated	class E basic helix-loop-helix protein 23
LOC100649370	-0.819616475	0.002528622	Down-regulated	homeobox protein MSX-2
LOC100651694	0.288322357	0.002528622	Up-regulated	6-phosphofructo-2-kinase/fructose-2
LOC105666918	-0.318270246	0.002528622	Down-regulated	dipeptidase 1
LOC100648891	-0.245388481	0.002567754	Down-regulated	protein SON
LOC100645354	0.211288437	0.002584046	Up-regulated	T-complex protein 1 subunit theta
LOC100642456	0.189909148	0.002587267	Up-regulated	protein Daple
LOC100646199	-0.405817265	0.002587267	Down-regulated	R3H domain-containing protein 2
LOC100649276	-0.396411893	0.00258766	Down-regulated	activator of 90 kDa heat shock protein ATPase homolog 1
LOC100646750	-0.237725095	0.002598365	Down-regulated	DENN domain-containing protein 1B
LOC100644717	0.668483058	0.002609687	Up-regulated	terminal nucleotidyltransferase 5C
LOC100648907	-0.30158354	0.002616629	Down-regulated	DNA-directed RNA polymerase II subunit RPB3
LOC100646673	-0.186675758	0.002624499	Down-regulated	mitochondrial Rho GTPase
LOC100647514	0.226078076	0.002641677	Up-regulated	histone-lysine N-methyltransferase eggless
LOC100642457	-0.369624608	0.002651458	Down-regulated	sodium/potassium-transporting ATPase subunit beta-1-interacting protein
LOC100645784	0.921130968	0.002653344	Up-regulated	spindle and kinetochore-associated protein 1
LOC100649396	0.291727104	0.002662492	Up-regulated	autophagy-related protein 9A
LOC100648784	0.3895682	0.002662515	Up-regulated	2-(3-amino-3-carboxypropyl)histidine synthase subunit 1
LOC100644295	0.416211081	0.002740046	Up-regulated	reticulon-4-interacting protein 1 homolog
LOC100645981	-0.155961084	0.002740046	Down-regulated	ankyrin repeat domain-containing protein 13D
LOC100646260	0.451390904	0.002740046	Up-regulated	receptor expression-enhancing protein 5
LOC100646827	-0.537275014	0.002740046	Down-regulated	potassium voltage-gated channel subfamily KQT member 1
LOC100650023	-0.644273588	0.002740046	Down-regulated	uncharacterized LOC100650023
LOC100650664	0.388589132	0.002740046	Up-regulated	glucose dehydrogenase [FAD
LOC100651912	0.737899406	0.002740046	Up-regulated	serine/threonine-protein kinase NLK
LOC100647650	-0.208184454	0.002778193	Down-regulated	hsp90 co-chaperone Cdc37
LOC100647213	0.250814395	0.002778198	Up-regulated	RNA-splicing ligase RtcB homolog
LOC100651447	-0.381546185	0.002790395	Down-regulated	tachykinins
LOC100643680	-0.373035729	0.002791731	Down-regulated	cyclin-dependent kinase inhibitor 2
LOC100651048	1.110749281	0.002806155	Up-regulated	aurora kinase B
LOC100642726	0.338205405	0.002828501	Up-regulated	eukaryotic translation initiation factor 3 subunit A
LOC100649657	0.367106897	0.002828501	Up-regulated	apoptosis-inducing factor 3
LOC100648362	-0.203216163	0.002836293	Down-regulated	pre-mRNA-processing-splicing factor 8
LOC100645828	0.687938646	0.002860362	Up-regulated	dystroglycan 1
LOC100646154	-0.330537427	0.002860362	Down-regulated	tau-tubulin kinase homolog Asator
LOC100650797	-0.563710379	0.002873569	Down-regulated	uncharacterized LOC100650797
LOC100649170	1.101887493	0.002875462	Up-regulated	uncharacterized LOC100649170
LOC105665861	0.32455928	0.002898106	Up-regulated	DDB1- and CUL4-associated factor 11
LOC100651639	-0.268218481	0.002901735	Down-regulated	E3 ubiquitin-protein ligase TRIM33
LOC100642217	-0.216741874	0.002912892	Down-regulated	pseudouridine-metabolizing bifunctional protein C1861.05
LOC100643663	0.217624868	0.002912892	Up-regulated	periodic tryptophan protein 1 homolog
LOC100647101	-0.143845588	0.002912892	Down-regulated	threonine--tRNA ligase 1
LOC100647782	-0.527097552	0.002912892	Down-regulated	homeobox protein aristaless-like 4
LOC100644130	0.221808311	0.002923046	Up-regulated	uncharacterized protein PF3D7_1120600
LOC100643919	-0.244420444	0.002971553	Down-regulated	uncharacterized LOC100643919
LOC110119166	-0.320574409	0.002971553	Down-regulated	uncharacterized LOC110119166
LOC100647567	-0.897890745	0.002975929	Down-regulated	semaphorin-1A
LOC100643472	0.366313494	0.002980107	Up-regulated	protein cordon-bleu
LOC100647385	-0.19580521	0.002994528	Down-regulated	fumarate hydratase
LOC100648034	-0.275305578	0.002999685	Down-regulated	F-box only protein 9
LOC100649968	-0.308407807	0.00300747	Down-regulated	dual specificity mitogen-activated protein kinase kinase dSOR1
LOC100645719	-0.257371574	0.003041168	Down-regulated	uncharacterized LOC100645719
LOC100643808	-0.46270948	0.003043775	Down-regulated	acyl-coenzyme A thioesterase 13
LOC100642518	-0.4130653	0.003050766	Down-regulated	DNA polymerase interacting tetratricopeptide repeat-containing
LOC100650781	0.15376646	0.00306642	Up-regulated	secretory carrier-associated membrane protein 5A
LOC100643219	-0.211016368	0.003075681	Down-regulated	putative pre-mRNA-splicing factor ATP-dependent RNA helicase PRP1
LOC100643643	-0.439896747	0.003075681	Down-regulated	nucleoredoxin
LOC100644811	-0.401947468	0.003075681	Down-regulated	neuroligin-4
LOC100650818	0.429902772	0.003100978	Up-regulated	transmembrane protein 18
LOC100645045	-0.395800157	0.003109082	Down-regulated	ecotropic viral integration site 5 ortholog
LOC100652137	0.439018504	0.003109082	Up-regulated	28 kDa heat- and acid-stable phosphoprotein
LOC100642207	0.220886284	0.003126898	Up-regulated	CDK-activating kinase assembly factor MAT1
LOC100642673	0.299839911	0.003126898	Up-regulated	proteasome subunit alpha type-1
LOC100649801	1.182991584	0.003126898	Up-regulated	uncharacterized LOC100649801
LOC100651290	0.201316197	0.003190157	Up-regulated	ralBP1-associated Eps domain-containing protein 1
LOC100643299	-0.186547364	0.003213227	Down-regulated	KAT8 regulatory NSL complex subunit 2
LOC100647004	0.462116291	0.003218321	Up-regulated	octopamine receptor beta-2R
LOC100646391	-0.311315091	0.003242072	Down-regulated	vacuolar protein sorting-associated protein 13B
LOC100650073	0.361676242	0.003242072	Up-regulated	double-strand break repair protein MRE11
LOC105666469	0.349295375	0.003242072	Up-regulated	signal recognition particle subunit SRP68
LOC100645047	1.225606851	0.003247318	Up-regulated	alpha-(1
LOC105666514	-0.455891483	0.003247318	Down-regulated	tetraspanin-11

LOC100642897	0.618748852	0.003266361	Up-regulated	cytochrome P450 18a1 LOC100642897
LOC100649983	1.242008416	0.003268573	Up-regulated	uncharacterized LOC100649983
LOC105665759	-0.379435171	0.003268573	Down-regulated	uncharacterized LOC105665759
LOC100644418	0.256886306	0.003269994	Up-regulated	ATP-dependent RNA helicase abstrakt
LOC100649998	0.184459893	0.00327079	Up-regulated	mediator of RNA polymerase II transcription subunit 23
LOC100652092	-0.279987928	0.00327079	Down-regulated	nucleoporin p58/p45
LOC105667083	0.729612123	0.00327079	Up-regulated	transcriptional regulator ATRX
LOC100644764	-0.191067542	0.003289978	Down-regulated	cytoplasmic protein NCK1
LOC100645698	-0.153245068	0.003296324	Down-regulated	zinc finger MYND domain-containing protein 11
LOC100643163	0.42013487	0.003316383	Up-regulated	SET and MYND domain-containing protein 4
LOC100646522	-0.483347446	0.003316567	Down-regulated	tetratricopeptide repeat protein 12
LOC100650395	-0.165729416	0.003324969	Down-regulated	splicing factor 3B subunit 1
LOC110119814	-0.950671206	0.003330089	Down-regulated	uncharacterized LOC110119814
LOC100649670	0.397583443	0.003338546	Up-regulated	nuclear autoantigenic sperm protein
LOC100644540	-0.38033298	0.003350697	Down-regulated	dynamin
LOC100644018	-0.407418461	0.003351743	Down-regulated	F-box/LRR-repeat protein 16
LOC100642837	-0.174119951	0.003384888	Down-regulated	F-box only protein 42
LOC100643155	0.281646557	0.003384888	Up-regulated	alcohol dehydrogenase class-3
LOC100644832	0.518330667	0.003419161	Up-regulated	farnesol dehydrogenase
LOC100652159	0.518335425	0.003423876	Up-regulated	protein YAE1
LOC100643660	-0.487821554	0.003426713	Down-regulated	kelch-like protein 10
LOC100647149	-0.480794215	0.003426713	Down-regulated	protein still life
LOC100644658	0.751723428	0.003441158	Up-regulated	muscle M-line assembly protein unc-89
LOC105666542	0.358046296	0.003441998	Up-regulated	rRNA N6-adenosine-methyltransferase ZCCHC4
LOC105666886	1.260303811	0.003454126	Up-regulated	facilitated trehalose transporter Tret1-like
LOC100643839	-0.286834208	0.00345554	Down-regulated	armadillo repeat-containing protein 6 homolog
LOC100642394	-0.411900944	0.003456582	Down-regulated	probable serine/threonine-protein kinase DDB_G0282963
LOC100642855	-0.337437476	0.003456582	Down-regulated	E3 ubiquitin-protein ligase HECW2
LOC100649500	-0.610881284	0.003460647	Down-regulated	dexamethasone-induced Ras-related protein 1
LOC110119729	0.81162137	0.003460647	Up-regulated	uncharacterized LOC110119729
LOC100645499	-0.322127798	0.003462455	Down-regulated	palmitoyl-protein thioesterase 1
LOC100651870	-0.40980314	0.003462455	Down-regulated	limbic system-associated membrane protein
LOC105667207	1.007590324	0.003462455	Up-regulated	uncharacterized LOC105667207
LOC100642880	0.340863337	0.003465947	Up-regulated	exocyst complex component 4
LOC100644451	-0.223338846	0.003465947	Down-regulated	calcium channel flower
LOC100647294	-0.211274023	0.003465947	Down-regulated	ribosomal protein S6 kinase alpha-2
LOC100643416	-0.246974123	0.00347532	Down-regulated	rabankyrin-5
LOC100648192	1.005221863	0.00347532	Up-regulated	glucose dehydrogenase [FAD
LOC100649310	0.245423333	0.00347532	Up-regulated	protein D2
LOC100651622	-0.225430101	0.00347532	Down-regulated	dnaJ homolog subfamily C member 7
LOC100643850	0.619030745	0.003488434	Up-regulated	lipid storage droplets surface-binding protein 2
LOC100646007	0.231891652	0.003520941	Up-regulated	ras association domain-containing protein 8
LOC100651253	0.389798325	0.003523971	Up-regulated	40S ribosomal protein S10
LOC100642763	-0.321302653	0.003528161	Down-regulated	histone deacetylase HDAC1
LOC100651379	0.274910974	0.00353072	Up-regulated	glycogen debranching enzyme
LOC100645646	-0.398204848	0.003542841	Down-regulated	FK506-binding protein 4
LOC100646376	-0.45253483	0.003548972	Down-regulated	dynein axonemal light chain 4
LOC100649246	0.313201345	0.003548972	Up-regulated	putative GTP-binding protein 6
LOC100642245	0.31460777	0.003551004	Up-regulated	zinc finger protein 16
LOC100642392	0.435279629	0.003551004	Up-regulated	uncharacterized LOC100642392
LOC100646150	-0.322457133	0.00357406	Down-regulated	malate dehydrogenase
LOC100646024	-0.395182994	0.003575882	Down-regulated	mannose-P-dolichol utilization defect 1 protein homolog
LOC100643618	-0.287236036	0.003593317	Down-regulated	coiled-coil domain-containing protein 28A
LOC100648562	-0.689807255	0.003602218	Down-regulated	gem-associated protein 6
LOC100648571	-0.35264482	0.003607866	Down-regulated	uncharacterized LOC100648571
LOC100650281	-0.228635333	0.003607866	Down-regulated	fatty acid hydroxylase domain-containing protein 2
LOC110119463	-0.299510906	0.003610855	Down-regulated	uncharacterized LOC110119463
LOC100651270	0.409136902	0.003623486	Up-regulated	EARP-interacting protein homolog
LOC100647997	-0.179552205	0.003631421	Down-regulated	beta-TrCP
LOC100651570	-0.201501168	0.003631421	Down-regulated	probable E3 ubiquitin-protein ligase RNF144A
LOC105667087	-0.294473714	0.003631421	Down-regulated	protein sly1 homolog
LOC100650452	0.337646613	0.003637284	Up-regulated	angiomotin-like protein 1
LOC100643464	-0.298607893	0.003667769	Down-regulated	ubiquitin carboxyl-terminal hydrolase 48
LOC100642481	-0.34824218	0.003672137	Down-regulated	THO complex subunit 6 homolog
LOC105666989	0.377845195	0.003672137	Up-regulated	serine/threonine-protein phosphatase 2A regulatory subunit B" subunit gamma
LOC100650756	-0.489742097	0.003685312	Down-regulated	adenosine receptor A2b
LOC100651176	-0.340059345	0.003689787	Down-regulated	double-stranded RNA-specific editase Adar
LOC100642973	1.324872337	0.003705616	Up-regulated	pro-resilin
LOC100651886	-1.169698885	0.003718784	Down-regulated	putative gustatory receptor 28b
LOC100650311	0.51131736	0.003720298	Up-regulated	protein 60A
LOC100645971	-0.387845973	0.00373205	Down-regulated	low-density lipoprotein receptor-related protein 2
LOC100642511	0.323485665	0.003733458	Up-regulated	peptidyl-prolyl cis-trans isomerase
LOC100651077	0.266128774	0.003752389	Up-regulated	JNK-interacting protein 3
LOC100646921	-0.882476371	0.003772521	Down-regulated	brain-specific homeobox protein homolog
LOC100648348	0.299587527	0.00377483	Up-regulated	ATP-dependent RNA helicase DDX55
LOC110120352	-0.638928479	0.003776621	Down-regulated	uncharacterized LOC110120352
LOC100647889	0.289990697	0.00379237	Up-regulated	vascular endothelial growth factor A-A
LOC100644581	0.89822693	0.003794924	Up-regulated	uncharacterized LOC100644581
LOC100648044	0.308234026	0.003794924	Up-regulated	transcription factor Dp-1
LOC100651956	-0.41438147	0.003806426	Down-regulated	uncharacterized LOC100651956
LOC105665924	-0.324038913	0.003889486	Down-regulated	SOSS complex subunit B homolog

LOC100647939	1.487269449	0.003915245	Up-regulated	tyrosine aminotransferase
LOC100646133	-0.293328327	0.00392027	Down-regulated	nucleoporin Nup188
LOC100642565	-0.404224075	0.00392461	Down-regulated	uncharacterized LOC100642565
LOC100644543	-0.416959116	0.00392461	Down-regulated	YLP motif-containing protein 1
LOC100645949	-0.329608669	0.003933905	Down-regulated	signal peptide peptidase-like 3
LOC100647583	-0.455926385	0.003933905	Down-regulated	lysine-specific histone demethylase 1A
LOC100645772	0.674756544	0.003941303	Up-regulated	putative fatty acyl-CoA reductase CG5065
LOC100648325	0.79455873	0.003941303	Up-regulated	prolactin-releasing peptide receptor
LOC100651397	-0.163489317	0.003955089	Down-regulated	saccharopine dehydrogenase-like oxidoreductase
LOC100651487	-1.576403726	0.003955089	Down-regulated	transcription factor 21
LOC105666353	0.55995427	0.004028708	Up-regulated	gamma-interferon-inducible lysosomal thiol reductase
LOC105666944	0.30774032	0.004028708	Up-regulated	nucleoporin Nup35
LOC100648196	0.275991272	0.004060593	Up-regulated	uncharacterized LOC100648196
LOC100643283	0.592403286	0.004063174	Up-regulated	pyruvate dehydrogenase E1 component subunit beta
LOC100644774	-0.318016801	0.004071103	Down-regulated	probable methyltransferase-like protein 23
LOC100650317	-1.006178146	0.004075434	Down-regulated	tubulin alpha-2/alpha-4 chain
LOC100642950	0.44082157	0.004083697	Up-regulated	uncharacterized LOC100642950
LOC100644403	0.311508962	0.004089586	Up-regulated	ATP-binding cassette subfamily G member 4
LOC100644955	-0.773953394	0.004093071	Down-regulated	LIM/homeobox protein Lhx5
LOC100643353	1.692478952	0.004094692	Up-regulated	IQ and ubiquitin-like domain-containing protein
LOC100648072	0.345776325	0.004116012	Up-regulated	meteorin-like protein
LOC100650663	1.47380876	0.00412083	Up-regulated	protein yellow
LOC100642423	0.258559673	0.004133943	Up-regulated	probable 28S rRNA (cytosine(4447)-C(5))-methyltransferase
LOC105666021	0.401392206	0.004151187	Up-regulated	lipid storage droplets surface-binding protein 2
LOC105665702	0.615704246	0.004172254	Up-regulated	uncharacterized LOC105665702
LOC100648289	-0.275805655	0.004175446	Down-regulated	D-glucuronyl C5-epimerase B
LOC100652034	-0.20682427	0.004175446	Down-regulated	exosome complex component RRP41
LOC100648016	0.214663843	0.004179197	Up-regulated	helicase SKI2W
LOC100650857	0.597300839	0.004194647	Up-regulated	ETS-related transcription factor Elf-5
LOC110120119	1.362490309	0.004194647	Up-regulated	uncharacterized LOC110120119
LOC100644145	-0.637537902	0.004203345	Down-regulated	L-threonine 3-dehydrogenase
LOC100651097	-0.383966757	0.004204156	Down-regulated	sphingomyelin phosphodiesterase
LOC100650287	0.51525983	0.004211849	Up-regulated	replication protein A 32 kDa subunit
LOC100651960	-0.366193517	0.004229633	Down-regulated	breast cancer anti-estrogen resistance protein 3 homolog
LOC100647865	0.512942952	0.004230131	Up-regulated	BRCA1-associated RING domain protein 1
LOC100649762	-0.516127834	0.004249347	Down-regulated	ovarian-specific serine/threonine-protein kinase Lok
LOC100650431	0.51440239	0.004277454	Up-regulated	jmjC domain-containing histone demethylation protein 1
LOC105666478	-0.320818688	0.004352299	Down-regulated	zinc finger protein GLI4
LOC100644510	1.292061863	0.004362772	Up-regulated	odorant receptor 82a
LOC100651843	-0.378615156	0.004378421	Down-regulated	voltage-dependent T-type calcium channel subunit alpha-1G
LOC100647383	0.344347713	0.004378568	Up-regulated	tryptophan--tRNA ligase
LOC100644747	-0.346323437	0.004384294	Down-regulated	integrator complex subunit 14
LOC100651948	1.211494396	0.004401779	Up-regulated	calcium and integrin-binding family member 3
LOC100652284	0.220546214	0.004424407	Up-regulated	F-box/WD repeat-containing protein 5
LOC100647767	-0.184324255	0.004447733	Down-regulated	oxysterol-binding protein-related protein 8
LOC100645471	0.400134346	0.004459578	Up-regulated	uncharacterized LOC100645471
LOC100649037	0.324341156	0.004469807	Up-regulated	uncharacterized LOC100649037
LOC100651426	-0.354104939	0.004483662	Down-regulated	CCAAT/enhancer-binding protein gamma
LOC100649795	-0.610940548	0.004504267	Down-regulated	glutamate receptor ionotropic
LOC100642935	0.263654965	0.004504303	Up-regulated	exosome complex exonuclease RRP44
LOC100651361	0.204545496	0.004521157	Up-regulated	restin homolog
LOC100649556	-0.238682796	0.004528781	Down-regulated	uncharacterized LOC100649556
LOC100650488	-0.641293046	0.004528781	Down-regulated	PR domain zinc finger protein 1
LOC105666578	0.782328259	0.004528781	Up-regulated	uncharacterized LOC105666578
LOC100648434	0.178804679	0.004553309	Up-regulated	NSFL1 cofactor p47
LOC100648557	0.595407044	0.004553309	Up-regulated	uncharacterized LOC100648557
LOC100650448	0.267649488	0.004553309	Up-regulated	FUN14 domain-containing protein 1
LOC100642460	-0.631482289	0.004562742	Down-regulated	netrin receptor UNC5B
LOC100646996	-0.284362823	0.004573889	Down-regulated	zinc finger-containing ubiquitin peptidase 1
LOC100647229	1.387784322	0.004581351	Up-regulated	troponin C
LOC100648738	0.352332973	0.004581351	Up-regulated	protein charybde
LOC105666351	0.19522435	0.004581351	Up-regulated	uncharacterized LOC105666351
LOC100648333	1.308776947	0.004585995	Up-regulated	uncharacterized LOC100648333
LOC100648350	0.31408097	0.004591317	Up-regulated	ferritin-3
LOC100652259	-0.182032586	0.004596349	Down-regulated	hippocampus abundant transcript 1 protein
LOC100642838	1.591012141	0.004614925	Up-regulated	ras-responsive element-binding protein 1
LOC100643492	-0.463392677	0.004614925	Down-regulated	protein kinase C
LOC100648226	0.288536292	0.004630367	Up-regulated	meiosis regulator and mRNA stability factor 1
LOC100645219	-0.40232348	0.004642757	Down-regulated	ADP-ribosylation factor-like protein 4C
LOC100649354	1.015696624	0.004642757	Up-regulated	uncharacterized LOC100649354
LOC100650216	-0.420500627	0.004649413	Down-regulated	uncharacterized LOC100650216
LOC100643678	2.657582974	0.004683996	Up-regulated	probable cytochrome P450 LOC100643678
LOC100646218	0.238198753	0.004683996	Up-regulated	uncharacterized LOC100646218
LOC100646073	-0.622087222	0.004692412	Down-regulated	uncharacterized LOC100646073
LOC105665849	-0.797999906	0.004695161	Down-regulated	uncharacterized LOC105665849
LOC100647030	0.259548526	0.004699076	Up-regulated	mitochondrial ribosome-associated GTPase 2
LOC100643971	-0.34527143	0.004705771	Down-regulated	synaptosomal-associated protein 25
LOC100644777	-0.331265046	0.004724524	Down-regulated	breast cancer metastasis-suppressor 1-like protein
LOC100649189	0.344778112	0.004746287	Up-regulated	60S ribosomal protein L24
LOC100651308	0.229868589	0.004746287	Up-regulated	ubiquitin-like protein 4A
LOC100651179	0.205338132	0.004749036	Up-regulated	cell division cycle and apoptosis regulator protein 1

LOC100650262	0.662359628	0.004757992	Up-regulated	outer dense fiber protein 3
LOC100648459	-0.626711664	0.004820253	Down-regulated	uncharacterized LOC100648459
LOC100649133	0.303037436	0.004820253	Up-regulated	actin-related protein 2/3 complex subunit 3
LOC100642604	-0.474886796	0.004820288	Down-regulated	uncharacterized LOC100642604
LOC100644159	-0.237357662	0.004820288	Down-regulated	mitochondrial-processing peptidase subunit beta
LOC100644856	-0.896244192	0.004820288	Down-regulated	uncharacterized LOC100644856
LOC100646551	-0.215207593	0.004820288	Down-regulated	uncharacterized protein CG5098
LOC105666367	2.552946526	0.004820288	Up-regulated	cytochrome P450 6k1 LOC105666367
LOC100644713	2.140899692	0.004856741	Up-regulated	laccase-1
LOC100649947	0.229460612	0.004856914	Up-regulated	short/branched chain specific acyl-CoA dehydrogenase
LOC100649385	0.375675224	0.004867424	Up-regulated	GPN-loop GTPase 3
LOC100650952	0.912173859	0.004867424	Up-regulated	insulin-like peptide receptor
LOC110119865	-0.693775604	0.004867424	Down-regulated	protein PFC0760c
LOC100649029	-0.197090544	0.004895398	Down-regulated	reticulocalbin-2
LOC100646822	1.215596496	0.004896476	Up-regulated	clavesin-2
LOC100648501	-0.633388979	0.004896476	Down-regulated	single-minded homolog 2
LOC100649716	-0.221304656	0.004904523	Down-regulated	serine/threonine-protein phosphatase 4 catalytic subunit
LOC100642278	0.251275818	0.004914823	Up-regulated	uncharacterized LOC100642278
LOC100645763	-0.285948212	0.004915242	Down-regulated	REST corepressor 3
LOC100645003	0.21907957	0.004918953	Up-regulated	A-kinase anchor protein 10
LOC110119489	-0.553406523	0.0049226	Down-regulated	uncharacterized LOC110119489
LOC100644480	-0.256598932	0.004940363	Down-regulated	histone H2A
LOC100647717	0.701981307	0.004944606	Up-regulated	cytochrome b-c1 complex subunit Rieske
LOC100644891	0.373228221	0.004971933	Up-regulated	probable phosphorylase b kinase regulatory subunit beta
LOC100646002	0.572130648	0.005088209	Up-regulated	organic cation transporter protein
LOC100648677	-0.536483618	0.00511354	Down-regulated	C-terminal-binding protein
LOC100644700	-0.167861091	0.005116469	Down-regulated	ubiquitin-like domain-containing CTD phosphatase 1
LOC100649435	-0.232452154	0.005156739	Down-regulated	synaptojanin-1
LOC100648316	0.243722343	0.005178635	Up-regulated	integrator complex subunit 8
LOC105666541	-0.483473735	0.005178635	Down-regulated	U-scoloptoxin(05)-Sm1a
LOC100647048	0.27212084	0.00517883	Up-regulated	ATP-dependent Clp protease ATP-binding subunit clpX-like
LOC100645008	0.37780391	0.005188042	Up-regulated	pre-mRNA-splicing factor CWC25 homolog
LOC100646339	0.821573216	0.005188042	Up-regulated	sestrin homolog
LOC100647357	0.473505321	0.005188042	Up-regulated	uncharacterized LOC100647357
LOC100647524	1.126726636	0.005188042	Up-regulated	UDP-glycosyltransferase UGT5
LOC100642814	-0.564992908	0.005213132	Down-regulated	uncharacterized LOC100642814
LOC100646267	0.203618869	0.005214443	Up-regulated	ell-associated factor Eaf
LOC100649352	0.521209642	0.005226126	Up-regulated	IQ motif-containing protein H
LOC100644215	0.222543725	0.005240736	Up-regulated	regulator of nonsense transcripts 1
LOC100646927	-0.239553002	0.005245944	Down-regulated	cullin-associated NEDD8-dissociated protein 1
LOC100647327	0.158310058	0.005245944	Up-regulated	protein Mo25
LOC100644449	-1.140895831	0.005252164	Down-regulated	paired box protein Pax-1
LOC100646943	0.21780931	0.005273686	Up-regulated	fatty-acid amide hydrolase 2-A
LOC100650246	-0.140405848	0.005273686	Down-regulated	ubiquitin-protein ligase E3C
LOC100645218	0.272436619	0.005277927	Up-regulated	E3 ubiquitin-protein ligase RNF113A
LOC100644283	0.413251893	0.005281184	Up-regulated	RPA-interacting protein
LOC100647104	0.303930069	0.005281184	Up-regulated	zinc finger protein 549
LOC100648475	0.28673819	0.005281184	Up-regulated	DNA replication factor Cdt1
LOC100647317	0.808334868	0.00532395	Up-regulated	uncharacterized LOC100647317
LOC100648637	0.443478152	0.00532395	Up-regulated	uncharacterized LOC100648637
LOC100650645	0.29246791	0.00532395	Up-regulated	syntaxin-17
LOC100651060	1.279962393	0.00532395	Up-regulated	uncharacterized LOC100651060
LOC100643859	-0.89956123	0.005380819	Down-regulated	anoctamin-4
LOC100648367	-0.273452208	0.005380819	Down-regulated	histone-arginine methyltransferase CARMER
LOC100651102	-0.387950689	0.005380819	Down-regulated	fumarylacetoacetate hydrolase domain-containing protein 2A
LOC100644312	-0.293284223	0.005391528	Down-regulated	putative uncharacterized protein DDB_G0282133
LOC100645331	-0.13108099	0.005401658	Down-regulated	probable elongation factor 1-delta
LOC100642696	0.321637017	0.005414998	Up-regulated	uncharacterized protein C7orf26 homolog
LOC100651937	0.230635603	0.005414998	Up-regulated	zinc finger protein 425
LOC110119285	-0.853072256	0.005414998	Down-regulated	cytochrome b-c1 complex subunit 9
LOC100644995	-1.300595828	0.005419375	Down-regulated	uncharacterized LOC100644995
LOC100643813	0.283198386	0.005435283	Up-regulated	V-type proton ATPase subunit D
LOC100644282	0.29837543	0.005435283	Up-regulated	WD repeat-containing protein 89
LOC100645020	0.346861741	0.00543823	Up-regulated	caskin-1
LOC100642316	1.004909674	0.005444117	Up-regulated	SET domain-containing protein SmydA-8
LOC100644042	-0.473463374	0.005445535	Down-regulated	excitatory amino acid transporter
LOC100642204	-0.264108956	0.005451055	Down-regulated	solute carrier family 35 member G1
LOC100647914	0.310126669	0.005480282	Up-regulated	dual specificity protein kinase CLK2
LOC100651800	-0.329767823	0.005486835	Down-regulated	death-associated protein kinase 1
LOC100652094	0.202218574	0.005510334	Up-regulated	WD repeat-containing protein 91
LOC100646465	0.369564988	0.005533264	Up-regulated	protein LLP homolog
LOC100647260	-0.365369283	0.005546662	Down-regulated	Down syndrome cell adhesion molecule-like protein Dscam2
LOC100642779	-0.232375738	0.005583662	Down-regulated	G protein-coupled receptor kinase 2
LOC100643454	0.33316937	0.005583662	Up-regulated	5'-deoxynucleotidase HDDC2
LOC100647998	0.333489089	0.005584582	Up-regulated	ras-related protein Rab-8A
LOC100644110	-0.510448687	0.005651114	Down-regulated	uncharacterized LOC100644110
LOC100644685	0.19404641	0.005651114	Up-regulated	centrosomal protein of 290 kDa
LOC100650717	-0.169645691	0.005654756	Down-regulated	nonsense-mediated mRNA decay factor SMG7-like
LOC100648840	-0.83389824	0.005677497	Down-regulated	ATP-binding cassette sub-family G member 5
LOC100646610	0.356862375	0.005680932	Up-regulated	60S ribosomal protein L7a
LOC100651987	0.930308602	0.005680932	Up-regulated	homogentisate 1

LOC100650086	0.656408354	0.005689957	Up-regulated	cytoplasmic dynein 2 light intermediate chain 1
LOC100650834	-0.547084911	0.005766424	Down-regulated	transcription factor collier
LOC105666957	-0.449670907	0.005772918	Down-regulated	uncharacterized LOC105666957
LOC100650016	0.457221851	0.005783489	Up-regulated	DNA repair and recombination protein RAD54-like
LOC100643847	0.210450477	0.005784542	Up-regulated	beclin 1-associated autophagy-related key regulator
LOC100647105	0.203538901	0.005788443	Up-regulated	notchless protein homolog 1
LOC100643444	0.226532744	0.005796996	Up-regulated	protein phosphatase inhibitor 2
LOC100644561	0.303538221	0.005798597	Up-regulated	eukaryotic translation initiation factor 3 subunit I
LOC100646582	0.984905257	0.005798597	Up-regulated	4-hydroxyphenylpyruvate dioxygenase
LOC100650486	-0.250449061	0.005798597	Down-regulated	lysosomal alpha-glucosidase
LOC105665725	-0.386293187	0.005798597	Down-regulated	uncharacterized LOC105665725
LOC100650828	-0.4587337	0.005811711	Down-regulated	alanine--tRNA ligase
LOC100645103	-0.299767171	0.005818567	Down-regulated	protein CLP1 homolog
LOC100648301	0.608245529	0.005818567	Up-regulated	uncharacterized LOC100648301
LOC100643148	0.362707708	0.005851411	Up-regulated	zinc transporter ZIP13 homolog
LOC100643585	0.579861561	0.005851411	Up-regulated	dipeptidyl aminopeptidase-like protein 6
LOC100648351	-0.231464135	0.005851411	Down-regulated	transmembrane protein 39A
LOC100652045	-0.181320492	0.00588162	Down-regulated	alsin
LOC100648201	0.154129325	0.00588274	Up-regulated	stromal membrane-associated protein 1
LOC100649073	-0.386125867	0.005904207	Down-regulated	ADP-ribose pyrophosphatase
LOC100644235	-0.49193911	0.005913787	Down-regulated	uncharacterized LOC100644235
LOC100643738	-0.339281966	0.005929328	Down-regulated	DNA-binding protein RFX7
LOC100652239	-0.46412328	0.005969245	Down-regulated	transcriptional activator protein Pur-beta
LOC110120284	1.174015962	0.005969245	Up-regulated	uncharacterized LOC110120284
LOC100643379	-0.370442741	0.005994967	Down-regulated	tubulin alpha-1 chain
LOC100642473	0.180615469	0.006004841	Up-regulated	ATP-dependent RNA helicase Ddx1
LOC100652086	0.221256011	0.006004841	Up-regulated	probable RNA-binding protein 19
LOC100647977	0.259220959	0.006070349	Up-regulated	uncharacterized LOC100647977
LOC100647602	0.472546851	0.006077132	Up-regulated	smad nuclear interacting protein 1-like
LOC100650017	-0.369100386	0.006094794	Down-regulated	PDZ domain-containing protein GIPC3
LOC100643048	-0.354170743	0.006111668	Down-regulated	protein sprint
LOC100644533	-0.123072995	0.006111668	Down-regulated	protein brunelleschi
LOC100644894	-0.479504454	0.006111668	Down-regulated	uncharacterized LOC100644894
LOC100646868	-0.141258782	0.006111668	Down-regulated	ATP-dependent helicase brm
LOC100649738	-0.353682548	0.006111668	Down-regulated	gem-associated protein 7
LOC105666371	-0.492734536	0.006111668	Down-regulated	uncharacterized LOC105666371
LOC100642313	0.241508486	0.006128033	Up-regulated	ubiquitin carboxyl-terminal hydrolase 46
LOC100648435	0.249186611	0.006192843	Up-regulated	26S proteasome non-ATPase regulatory subunit 9
LOC100648631	0.249186611	0.006192843	Up-regulated	26S proteasome non-ATPase regulatory subunit 9
LOC100650718	1.511369389	0.006291059	Up-regulated	uncharacterized LOC100650718
LOC100642375	-0.35137783	0.006293305	Down-regulated	nuclear pore complex protein Nup93
LOC105665638	1.079046597	0.006293305	Up-regulated	putative WEB family protein At1g65010
LOC105666229	1.082162075	0.006336744	Up-regulated	uncharacterized LOC105666229
LOC100644410	0.349771866	0.006343916	Up-regulated	protein transport protein Sec61 subunit gamma
LOC100648433	1.007139892	0.006346057	Up-regulated	uncharacterized aarF domain-containing protein kinase 2
LOC100645223	0.767613132	0.006354721	Up-regulated	peroxiredoxin-5
LOC100648296	-0.746714241	0.006354721	Down-regulated	protein split ends
LOC100650954	-0.454133051	0.006376374	Down-regulated	phosrestin-2
LOC100651474	-0.675655775	0.006376374	Down-regulated	neuroligin-1
LOC100644257	-0.770597441	0.0063841	Down-regulated	uncharacterized LOC100644257
LOC100648293	0.331187202	0.006395977	Up-regulated	phosphoribosylformylglycinamidine synthase
LOC110119579	-0.424865484	0.006395977	Down-regulated	zinc finger protein 350
LOC100650189	0.425071246	0.006438235	Up-regulated	UPF0235 protein C15orf40 homolog
LOC100646168	0.360705189	0.00644455	Up-regulated	cdc42 homolog
LOC100646964	-0.423427639	0.00644455	Down-regulated	ornithine aminotransferase
LOC100643510	-0.51720689	0.006450004	Down-regulated	cAMP-dependent protein kinase catalytic subunit 1
LOC100644653	-0.308357201	0.006453179	Down-regulated	rapamycin-insensitive companion of mTOR
LOC100651538	-0.552998456	0.006464769	Down-regulated	lachesin
LOC100646857	-0.339287615	0.006470185	Down-regulated	tricalbin-1
LOC100643401	0.264688512	0.006495588	Up-regulated	WW domain-binding protein 4
LOC100650333	-0.792955087	0.006501299	Down-regulated	uncharacterized LOC100650333
LOC100647456	0.510850279	0.006517023	Up-regulated	dynein axonemal assembly factor 10
LOC100649710	0.320446318	0.006517023	Up-regulated	B-cell receptor-associated protein 31
LOC100651266	-0.231966137	0.006517023	Down-regulated	S-adenosylmethionine mitochondrial carrier protein
LOC100652277	0.19875569	0.006517023	Up-regulated	bromodomain adjacent to zinc finger domain protein 1A
LOC100650649	-0.538412511	0.006518826	Down-regulated	protein artichoke
LOC100643003	-0.447108757	0.006530502	Down-regulated	leucine-rich repeat-containing protein 58
LOC100643348	0.357483688	0.006549466	Up-regulated	membrane-associated progesterone receptor component 1
LOC100649188	-0.766680833	0.006636457	Down-regulated	collagen alpha-1(IV) chain
LOC100651837	-0.788963528	0.006645941	Down-regulated	protein SCAI
LOC100644651	0.222697544	0.006647643	Up-regulated	NAD-dependent protein deacetylase sirtuin-7
LOC100650201	0.140413944	0.006699808	Up-regulated	putative protein FAM10A4
LOC100648991	-0.308547178	0.006713391	Down-regulated	calcium and integrin-binding protein 1
LOC100650920	-0.242981763	0.006858733	Down-regulated	NAD-dependent protein deacetylase sirtuin-1
LOC100644871	0.219873232	0.00686777	Up-regulated	uncharacterized LOC100644871
LOC100651036	-0.498795568	0.006872129	Down-regulated	glutamate receptor ionotropic
LOC100648054	-0.224951347	0.006874492	Down-regulated	serine/threonine-protein kinase PRP4 homolog
LOC100651273	0.238032836	0.006884343	Up-regulated	exportin-1
LOC100643453	0.531782286	0.00689553	Up-regulated	GAS2-like protein pickled eggs
LOC100645967	-0.520415028	0.006911951	Down-regulated	formin-2
LOC100646444	0.775618326	0.006957992	Up-regulated	uncharacterized LOC100646444

LOC100649336	1.655159614	0.006957992	Up-regulated	putative mediator of RNA polymerase II transcription subunit 26
LOC100645422	-0.683125234	0.006972672	Down-regulated	reversion-inducing cysteine-rich protein with Kazal motifs
LOC100651905	-0.312495858	0.007036283	Down-regulated	low density lipoprotein receptor adapter protein 1-B
LOC100644056	-0.250440257	0.007036962	Down-regulated	FERM domain-containing protein 8
LOC100643840	-0.261414649	0.007059201	Down-regulated	poly(A) polymerase type 3
LOC100643552	0.318738206	0.007084934	Up-regulated	outer mitochondrial transmembrane helix translocase
LOC100642737	-0.454079132	0.007101635	Down-regulated	agrin
LOC100651887	0.814645652	0.007102571	Up-regulated	extracellular matrix protein 2
LOC105665781	0.42782918	0.007102571	Up-regulated	transmembrane ascorbate-dependent reductase CYB561
LOC100644600	-0.616296129	0.00710587	Down-regulated	facilitated trehalose transporter Tret1
LOC100643844	-0.545786047	0.007107216	Down-regulated	calmodulin
LOC100642418	1.327202007	0.007115364	Up-regulated	receptor-type tyrosine-protein phosphatase delta
LOC100647117	0.615549561	0.007136311	Up-regulated	ATP-sensitive inward rectifier potassium channel 12
LOC100647302	0.227104058	0.007159849	Up-regulated	phospholipid-transporting ATPase ABCA1
LOC100648446	-0.159877347	0.007177716	Down-regulated	microtubule-associated protein RP/EB family member 1
LOC100650614	0.446497557	0.007177716	Up-regulated	translocon-associated protein subunit gamma
LOC100650358	-0.32446759	0.007227634	Down-regulated	1-acyl-sn-glycerol-3-phosphate acyltransferase alpha
LOC100649093	-0.32953454	0.007243797	Down-regulated	b(0
LOC100643343	0.331319761	0.00726269	Up-regulated	DNA replication licensing factor Mcm6
LOC100643790	-0.614536465	0.00726269	Down-regulated	gamma-aminobutyric acid type B receptor subunit 1
LOC100646134	-0.321548751	0.00726269	Down-regulated	DNA-binding protein RFXANK
LOC100647306	0.179147603	0.00726269	Up-regulated	actin-related protein 10
LOC100648953	-0.405781932	0.00726269	Down-regulated	116 kDa U5 small nuclear ribonucleoprotein component
LOC100648961	-0.445633376	0.00726269	Down-regulated	uncharacterized LOC100648961
LOC100649113	-0.364956849	0.00726269	Down-regulated	zinc transporter ZIP11
LOC105667058	2.463732493	0.007267674	Up-regulated	putative fatty acyl-CoA reductase CG5065
LOC100651088	1.618643492	0.007279227	Up-regulated	GATA-binding factor A
LOC100650560	-0.508617052	0.007281782	Down-regulated	triple functional domain protein
LOC100645002	-0.37110559	0.00731407	Down-regulated	dnaJ protein homolog 1
LOC100645822	1.432279816	0.007340419	Up-regulated	protein dead ringer homolog
LOC100652139	-0.318488919	0.007386333	Down-regulated	PRADC1-like protein
LOC100645222	-0.249871463	0.007398037	Down-regulated	aminoacylase-1
LOC100647807	0.242927895	0.007398037	Up-regulated	transmembrane and coiled-coil domains protein 2
LOC100650279	0.658166297	0.007398037	Up-regulated	RNA polymerase II elongation factor Ell
LOC100652315	-0.454041908	0.007398037	Down-regulated	uncharacterized LOC100652315
LOC105665778	-0.231108834	0.007407031	Down-regulated	regulator of microtubule dynamics protein 1-like
LOC100646567	0.312795013	0.007432365	Up-regulated	uncharacterized LOC100646567
LOC100645001	-0.254221964	0.007435519	Down-regulated	ribosome-releasing factor 2
LOC100648056	0.354039504	0.007435519	Up-regulated	UPF0488 protein CG14286
LOC100649255	-0.500116198	0.007435519	Down-regulated	protein unc-79 homolog
LOC110119509	0.409309495	0.007435519	Up-regulated	L-aminoadipate-semialdehyde dehydrogenase-phosphopantetheinyl transferase
LOC100644714	-0.339043003	0.007435646	Down-regulated	nuclear cap-binding protein subunit 2
LOC100650814	0.338343984	0.007435646	Up-regulated	uncharacterized LOC100650814
LOC100649851	0.408638526	0.007447964	Up-regulated	60S ribosomal protein L17
LOC100642282	-0.218202395	0.007450979	Down-regulated	transmembrane protein 256 homolog
LOC100643209	-0.308816428	0.007474702	Down-regulated	protein stoned-B
LOC100645890	-0.5033806	0.007474849	Down-regulated	uncharacterized LOC100645890
LOC100649350	-0.355592751	0.007510181	Down-regulated	uncharacterized LOC100649350
LOC100648492	-0.239863734	0.007510995	Down-regulated	protein singed
LOC100648252	2.220627703	0.007523939	Up-regulated	paternally-expressed gene 3 protein
LOC100642976	0.158648856	0.007526931	Up-regulated	E3 ubiquitin-protein ligase KCMF1
LOC100644355	-0.442820271	0.007526931	Down-regulated	chondroitin sulfate proteoglycan 4
LOC100645275	0.78597028	0.00758383	Up-regulated	GDP-D-glucose phosphorylase 1
LOC100651633	0.225780571	0.007593425	Up-regulated	BRO1 domain-containing protein BROX
LOC100649202	0.791718999	0.007606364	Up-regulated	neuropeptides capa receptor
LOC100642867	0.384086578	0.007606712	Up-regulated	serine/threonine-protein kinase OSR1
LOC105666990	1.696211016	0.007615861	Up-regulated	ATPase family AAA domain-containing protein 2
LOC100643796	-0.583217883	0.00761666	Down-regulated	uncharacterized LOC100643796
LOC100651336	1.645521565	0.00761666	Up-regulated	hornerin
LOC100650463	-0.361532741	0.007640518	Down-regulated	uncharacterized LOC100650463
LOC100642213	0.236833702	0.007647332	Up-regulated	cullin-4B
LOC100643240	-0.207158189	0.007667554	Down-regulated	zinc finger protein 267
LOC100643298	-0.251548104	0.007667554	Down-regulated	metaxin-2
LOC100648017	0.343525927	0.007667554	Up-regulated	39S ribosomal protein L11
LOC105666153	0.311487246	0.007667554	Up-regulated	3-oxoacyl-[acyl-carrier-protein] reductase FabG
LOC110119314	0.632865909	0.007667554	Up-regulated	cell wall protein DAN4-like
LOC100646512	-0.289726767	0.007696892	Down-regulated	nuclear pore complex protein Nup133
LOC100647956	-0.433644014	0.0077212	Down-regulated	synaptotagmin-7
LOC100651777	0.265266071	0.0077212	Up-regulated	xaa-Pro aminopeptidase ApepP
LOC100645620	0.271827533	0.00772348	Up-regulated	lysosomal Pro-X carboxypeptidase
LOC100646385	-0.570566725	0.007724139	Down-regulated	lipid storage droplets surface-binding protein 1
LOC100649939	-0.570233189	0.007772696	Down-regulated	uncharacterized LOC100649939
LOC100650685	0.535634321	0.007772696	Up-regulated	rho guanine nucleotide exchange factor 10
LOC100647630	-0.337070011	0.007803775	Down-regulated	eukaryotic translation initiation factor 4E-1A
LOC100648646	-0.302797166	0.007803775	Down-regulated	uncharacterized LOC100648646
LOC100642254	0.291839135	0.007821097	Up-regulated	general transcription factor IIE subunit 1
LOC100647411	-0.396319307	0.007844858	Down-regulated	60S ribosomal protein L11
LOC100650783	1.192443342	0.007864319	Up-regulated	transcription factor Sp9
LOC100651169	-0.351777514	0.007880973	Down-regulated	chromatin modification-related protein eaf-1
LOC100648311	0.794681379	0.007882167	Up-regulated	T-cell leukemia homeobox protein 3
LOC100649395	-0.262951019	0.007882167	Down-regulated	elongation of very long chain fatty acids protein 6

LOC100645713	0.86550548	0.007885322	Up-regulated	uncharacterized LOC100645713
LOC100645391	-0.276460095	0.007901297	Down-regulated	midasin
LOC100647726	0.22367253	0.007984298	Up-regulated	cytosolic carboxypeptidase-like protein 5
LOC100650290	0.644411207	0.007995403	Up-regulated	tetratricopeptide repeat protein 19 homolog
LOC100644290	-0.406885069	0.008058359	Down-regulated	uncharacterized LOC100644290
LOC100643417	0.336273643	0.008063651	Up-regulated	uncharacterized LOC100643417
LOC100645369	0.527687121	0.008098749	Up-regulated	excitatory amino acid transporter 1
LOC100650393	0.260600957	0.008110302	Up-regulated	cyclic AMP-dependent transcription factor ATF-6 alpha
LOC105665683	0.212916908	0.008110302	Up-regulated	transmembrane and ubiquitin-like domain-containing protein 1
LOC100651517	1.139416067	0.008127467	Up-regulated	RNA-binding protein fusilli
LOC105666656	4.157515825	0.008166575	Up-regulated	synaptonemal complex protein 1
LOC105666292	0.291883096	0.0081674	Up-regulated	dihydrolipoyllysine-residue acetyltransferase component pyruvate dehydrogenase
LOC100645187	-0.230675551	0.008265071	Down-regulated	rab3 GTPase-activating protein catalytic subunit
LOC100644999	-0.280344271	0.008280393	Down-regulated	protein retinal degeneration B
LOC100649067	-0.288279126	0.008280393	Down-regulated	phosphatidate phosphatase LPIN3
LOC100642426	-0.154924458	0.008316217	Down-regulated	liprin-beta-1
LOC100650658	-0.397972018	0.008317262	Down-regulated	uncharacterized LOC100650658
LOC100643529	0.187632289	0.00833181	Up-regulated	nudC domain-containing protein 3
LOC100644950	0.272053549	0.00833181	Up-regulated	CXXC-type zinc finger protein 1
LOC100643884	-1.163176541	0.008358966	Down-regulated	DNA translocase FtsK
LOC100649490	-0.656257946	0.008358966	Down-regulated	uncharacterized LOC100649490
LOC100646963	0.485318643	0.008363394	Up-regulated	testicular acid phosphatase homolog
LOC110120070	-0.549503555	0.008363394	Down-regulated	leucine-rich repeat-containing protein 15-like
LOC100647501	-0.618794149	0.008392597	Down-regulated	uncharacterized LOC100647501
LOC100648841	0.353453241	0.008454603	Up-regulated	protein Gawky
LOC100646388	0.240310237	0.008498249	Up-regulated	prenylated Rab acceptor protein 1
LOC100648984	0.248995486	0.008498249	Up-regulated	probable ATP-dependent RNA helicase DDX47
LOC100643266	-0.251251389	0.008499352	Down-regulated	chromatin-remodeling complex ATPase chain Iswi
LOC100644759	-0.21918923	0.008509868	Down-regulated	transmembrane protein 179
LOC100646221	-0.549478622	0.008509868	Down-regulated	glutamate receptor ionotropic
LOC100646560	0.431586814	0.008510296	Up-regulated	transcription factor 15
LOC100650302	0.31334943	0.008510296	Up-regulated	zinc transporter 1
LOC100650177	-0.449284621	0.008513029	Down-regulated	uncharacterized LOC100650177
LOC100643086	-0.315388895	0.008516845	Down-regulated	uncharacterized LOC100643086
LOC110119233	-0.296134798	0.008547137	Down-regulated	uncharacterized LOC110119233
LOC100642807	0.310373982	0.008569406	Up-regulated	icarapin-like
LOC100647355	-0.410546353	0.008656078	Down-regulated	uncharacterized LOC100647355
LOC100644396	0.916448761	0.008661975	Up-regulated	2-oxoisovalerate dehydrogenase subunit beta
LOC100648388	-0.249775042	0.008693308	Down-regulated	polynucleotide 5'-hydroxyl-kinase NOL9
LOC100647136	-0.526626763	0.008721274	Down-regulated	thioredoxin domain-containing protein 17
LOC100650735	0.165805698	0.008804277	Up-regulated	tyrosine-protein phosphatase non-receptor type 23
LOC100647232	0.635848363	0.008984282	Up-regulated	uncharacterized LOC100647232
LOC100647573	0.200595885	0.009007205	Up-regulated	protein mahjong
LOC100643982	-0.355610223	0.009017954	Down-regulated	disks large 1 tumor suppressor protein
LOC100650109	0.554046107	0.009017954	Up-regulated	bicaudal D-related protein homolog
LOC100644112	0.511732065	0.009065118	Up-regulated	fructose-bisphosphate aldolase
LOC100645756	-0.435665729	0.009082125	Down-regulated	gamma-aminobutyric acid type B receptor subunit 2
LOC100647265	-0.461033858	0.009082125	Down-regulated	globin
LOC100644797	0.253561103	0.009114447	Up-regulated	iron-sulfur cluster assembly scaffold protein IscU
LOC100649080	0.324538362	0.009150866	Up-regulated	rab11 family-interacting protein 2
LOC105666131	1.101032086	0.009157128	Up-regulated	uncharacterized LOC105666131
LOC110119219	-0.375480871	0.009173573	Down-regulated	glutathione synthetase
LOC100647131	0.228160861	0.009181969	Up-regulated	sodium-dependent transporter bedraggled
LOC100648466	-0.183015221	0.009182612	Down-regulated	geranylgeranyl transferase type-1 subunit beta
LOC100643689	0.903505393	0.009188907	Up-regulated	synaptogenesis protein syg-2
LOC100645309	-0.313740295	0.009188907	Down-regulated	ATP-dependent RNA helicase vasa
LOC100647485	0.209906431	0.009196707	Up-regulated	multiple epidermal growth factor-like domains protein 8
LOC100643261	0.366296355	0.009207141	Up-regulated	m7GpppX diphosphatase
LOC100643812	0.236652729	0.009221891	Up-regulated	zinc finger protein 37
LOC100646704	-0.197330391	0.009254792	Down-regulated	essential MCU regulator
LOC100643959	-0.504995186	0.009276599	Down-regulated	uncharacterized LOC100643959
LOC100646660	0.342765708	0.009276599	Up-regulated	transcriptional protein SWT1
LOC100650142	-0.594347987	0.009322576	Down-regulated	furin-like protease 1
LOC100644074	-0.434168434	0.009322658	Down-regulated	uncharacterized LOC100644074
LOC100642470	0.232386574	0.009328385	Up-regulated	traB domain-containing protein
LOC100648156	-0.30999485	0.009329656	Down-regulated	COP9 signalosome complex subunit 9
LOC100645734	0.173578953	0.009372991	Up-regulated	acidic leucine-rich nuclear phosphoprotein 32 family member A
LOC100648040	-0.322558161	0.009376991	Down-regulated	brefeldin A-inhibited guanine nucleotide-exchange protein 1
LOC105667088	0.432250031	0.009380273	Up-regulated	uncharacterized LOC105667088
LOC100649737	-0.363433853	0.009387908	Down-regulated	zwei Ig domain protein zig-8
LOC100645660	-0.228940343	0.009423171	Down-regulated	endoplasmic reticulum resident protein 29
LOC100651090	0.265436968	0.009423171	Up-regulated	inositol polyphosphate 5-phosphatase E
LOC100648204	0.597667876	0.009487009	Up-regulated	FH1/FH2 domain-containing protein 3
LOC105665793	0.972740923	0.009493502	Up-regulated	uncharacterized LOC105665793
LOC100644386	-0.261745566	0.009499127	Down-regulated	tumor necrosis factor receptor superfamily member 4
LOC100645681	0.550159681	0.009499127	Up-regulated	pancreatic lipase-related protein 2
LOC100631059	0.338700679	0.009506538	Up-regulated	NF-kappa-B inhibitor cactus 2
LOC100651226	0.175725889	0.009538955	Up-regulated	exocyst complex component 8
LOC100651351	0.192121456	0.009551047	Up-regulated	large proline-rich protein BAG6
LOC100646480	0.288555907	0.009568019	Up-regulated	peroxisomal biogenesis factor 19
LOC100651624	0.198070044	0.009568019	Up-regulated	uncharacterized LOC100651624

LOC100647328	-0.271676902	0.009568395	Down-regulated	homer protein homolog 2
LOC100647971	-0.346189665	0.009574824	Down-regulated	discoidin domain-containing receptor 2
LOC100643756	-0.408900248	0.00963912	Down-regulated	uncharacterized LOC100643756
LOC100643981	-0.546827633	0.00965384	Down-regulated	high-affinity choline transporter 1
LOC100649072	0.174776635	0.009743871	Up-regulated	uncharacterized LOC100649072
LOC100651550	-0.414016873	0.009743876	Down-regulated	prominin-1-A
LOC100648382	-0.555444938	0.009752705	Down-regulated	cysteine sulfinic acid decarboxylase
LOC110119153	0.404634772	0.009760302	Up-regulated	HIG1 domain family member 2A
LOC100650097	0.288096984	0.009784402	Up-regulated	adhesion G protein-coupled receptor A3
LOC100648721	0.343877384	0.009814256	Up-regulated	NF-kappa-B essential modulator
LOC100644609	-0.147049014	0.009826863	Down-regulated	V-type proton ATPase 116 kDa subunit a 1
LOC100643963	0.187343967	0.009830762	Up-regulated	protein phosphatase methylesterase 1
LOC100645027	-0.285839574	0.009830762	Down-regulated	spermine oxidase-like
LOC100647607	-0.308561982	0.009830762	Down-regulated	RNA-binding protein 1
LOC100649588	-0.268159507	0.009830762	Down-regulated	ribitol 5-phosphate transferase FKRP
LOC100650864	0.640334149	0.009830762	Up-regulated	uncharacterized LOC100650864
LOC100651235	-0.354308117	0.009830762	Down-regulated	G-protein coupled receptor Mth2
LOC105666147	-0.499803469	0.009830762	Down-regulated	adenylate kinase isoenzyme 1
LOC105667054	0.904099807	0.009830762	Up-regulated	uncharacterized LOC105667054
LOC100646108	-0.222794848	0.009862269	Down-regulated	polyadenylate-binding protein 2-B
LOC100651272	0.268245424	0.009862269	Up-regulated	uncharacterized protein C1orf198 homolog
LOC100648322	0.333418751	0.009886889	Up-regulated	peptidoglycan-recognition protein LC
LOC100645608	0.742466959	0.009893339	Up-regulated	protein snakeskin
LOC100648913	-0.553505155	0.00996504	Down-regulated	uncharacterized LOC100648913
LOC100651368	-0.295123844	0.009997983	Down-regulated	scaffold protein salvador
LOC100650840	0.177112356	0.010016153	Up-regulated	ribonuclease 3
LOC100651717	-0.271411891	0.010023827	Down-regulated	spastin
LOC100651923	1.894324269	0.010023827	Up-regulated	protein yellow
LOC100644531	0.228267293	0.010034635	Up-regulated	rho GTPase-activating protein 44
LOC100649864	-0.585123794	0.010034635	Down-regulated	DNA-directed RNA polymerase III subunit RPC7-like
LOC100645830	0.54258129	0.010064081	Up-regulated	unconventional myosin ID
LOC100651035	-0.317744371	0.010064081	Down-regulated	glutathione S-transferase
LOC100646120	1.027007316	0.010069088	Up-regulated	uncharacterized LOC100646120
LOC100645897	0.421307206	0.010082262	Up-regulated	CDK5 regulatory subunit-associated protein 3
LOC100645679	0.493285176	0.010105885	Up-regulated	peptide transporter family 1
LOC100645348	-0.472575095	0.01016257	Down-regulated	collagen alpha chain CG42342
LOC100642968	0.861932277	0.010172479	Up-regulated	probable tubulin polyglutamylase TTLL2
LOC100646338	-0.296840998	0.010174957	Down-regulated	protein sidekick
LOC100652002	-0.83781325	0.010278731	Down-regulated	EF-hand calcium-binding domain-containing protein 1
LOC100643385	-0.257530411	0.010284023	Down-regulated	tetraspanin-1
LOC100650362	-0.322721401	0.010313931	Down-regulated	uncharacterized LOC100650362
LOC100648620	-0.444246477	0.010331698	Down-regulated	leucine-rich repeat and immunoglobulin domain-containing nogo receptor
LOC100650632	0.55631732	0.010358298	Up-regulated	sentrin-specific protease 8
LOC100642844	-0.463175091	0.01036326	Down-regulated	mycosubtilin synthase subunit C
LOC100647414	0.276156289	0.01036326	Up-regulated	small integral membrane protein 14
LOC100647774	-0.237935092	0.01038932	Down-regulated	GPI ethanolamine phosphate transferase 3
LOC100643652	-0.247226674	0.010390231	Down-regulated	endothelial differentiation-related factor 1 homolog
LOC100645384	-0.421406506	0.010390231	Down-regulated	UDP-glucosyltransferase 2
LOC100651882	-0.369356041	0.010390231	Down-regulated	apolipoprotein D
LOC100649884	0.702460798	0.010400594	Up-regulated	myocyte-specific enhancer factor 2
LOC100648980	-0.44166957	0.010412104	Down-regulated	insulin-like growth factor I
LOC100650883	-0.47874567	0.010468414	Down-regulated	anion exchange protein 2
LOC100644479	-0.245776694	0.010512783	Down-regulated	density-regulated protein homolog
LOC100651073	-0.290302574	0.01053448	Down-regulated	E3 SUMO-protein ligase ZBED1
LOC100644028	-0.30211802	0.01054741	Down-regulated	endophilin-A
LOC100649612	-0.259069986	0.010602622	Down-regulated	acetylcholine receptor subunit beta-like 1
LOC100646142	0.576541155	0.010605514	Up-regulated	glycine receptor subunit alpha-1
LOC100646246	1.219268318	0.010616206	Up-regulated	arylsulfatase B
LOC100642408	1.192219632	0.010714645	Up-regulated	serine/threonine-protein kinase ICK
LOC100644164	-0.82043474	0.010714645	Down-regulated	uncharacterized LOC100644164
LOC100645597	1.074870457	0.010714645	Up-regulated	uncharacterized LOC100645597
LOC100652155	0.237291194	0.010714645	Up-regulated	cytoplasmic tRNA 2-thiolation protein 1
LOC100642253	-0.349930962	0.010780641	Down-regulated	succinate--hydroxymethylglutarate CoA-transferase
LOC100647345	-0.153526188	0.010780641	Down-regulated	serine/threonine-protein phosphatase PP1-beta catalytic subunit
LOC105667201	0.970432857	0.010780641	Up-regulated	uncharacterized LOC105667201
LOC100646997	-0.181603866	0.010965839	Down-regulated	BLOC-1-related complex subunit 5
LOC100648409	-0.216605979	0.010977859	Down-regulated	amyloid protein-binding protein 2
LOC100644168	1.780747506	0.010983263	Up-regulated	potassium/sodium hyperpolarization-activated cyclic nucleotide-gated channel 1
LOC100647051	0.722292396	0.010983263	Up-regulated	beta-ureidopropionase
LOC100642806	0.382082813	0.010991213	Up-regulated	protein SHQ1 homolog
LOC100647135	-0.612139768	0.010995541	Down-regulated	voltage-dependent calcium channel type A subunit alpha-1
LOC100649964	0.189694085	0.010995541	Up-regulated	vacuolar protein sorting-associated protein 54
LOC100642308	-0.22959281	0.010997121	Down-regulated	protein JTB
LOC100644742	-0.434337117	0.010997121	Down-regulated	glycine dehydrogenase (decarboxylating)
LOC100647010	-0.209247535	0.010997121	Down-regulated	elongation factor G
LOC100649775	-0.365949047	0.010997121	Down-regulated	protein numb
LOC105665737	-0.446732149	0.011003837	Down-regulated	centrosomal and chromosomal factor
LOC100642385	-0.459049209	0.011057838	Down-regulated	fl(2)d-associated complex component
LOC100647333	-0.396572219	0.01106076	Down-regulated	sodium/potassium-transporting ATPase subunit alpha
LOC100642432	-0.241869038	0.011079656	Down-regulated	anaphase-promoting complex subunit 7
LOC100648126	0.306425871	0.011095477	Up-regulated	angiotensin-converting enzyme

LOC100645318	-0.233196993	0.011102951	Down-regulated	progestin and adipoQ receptor family member 3
LOC100648829	-0.177231751	0.011108957	Down-regulated	ras-related protein Rab6
LOC100651251	0.280669388	0.011111011	Up-regulated	cytochrome c oxidase assembly protein COX11
LOC100642636	0.449377349	0.01112757	Up-regulated	probable cytosolic iron-sulfur protein assembly protein Ciao1
LOC105665751	-0.453916702	0.01112757	Down-regulated	transcription and mRNA export factor ENY2
LOC100644721	-0.377753423	0.011186249	Down-regulated	teneurin-a
LOC100647338	0.177008333	0.011190922	Up-regulated	GDP-mannose 4
LOC100650046	0.297878416	0.011193678	Up-regulated	protein catecholamines up
LOC100644779	0.413713608	0.011203526	Up-regulated	insulin receptor substrate 1
LOC105665872	0.147152194	0.01126954	Up-regulated	beta-parvin
LOC100648626	0.174505292	0.011299252	Up-regulated	mediator of RNA polymerase II transcription subunit 17
LOC100648079	-0.97561149	0.011307457	Down-regulated	endoglucanase E-4
LOC100651551	-0.369679113	0.011426908	Down-regulated	thrombospondin type-1 domain-containing protein 4
LOC100642425	-0.239075692	0.011436747	Down-regulated	GPI transamidase component PIG-S
LOC100642647	-0.383958306	0.011436747	Down-regulated	cationic amino acid transporter 2
LOC100649284	0.754670846	0.011436747	Up-regulated	invertebrate-type lysozyme 3
LOC100642769	-0.476568988	0.011460179	Down-regulated	elongation of very long chain fatty acids protein AAEL008004
LOC100643860	-0.397706854	0.011460179	Down-regulated	uncharacterized LOC100643860
LOC100645572	-0.243648902	0.011460179	Down-regulated	threonylcarbamoyladenosine tRNA methylthiotransferase
LOC100651405	0.811275702	0.011461235	Up-regulated	juvenile hormone esterase-like
LOC100646192	0.393574098	0.011487222	Up-regulated	probable Bax inhibitor 1
LOC100650512	0.361576619	0.011487954	Up-regulated	mitochondrial RNA pseudouridine synthase rpusd4
LOC100644500	0.194527796	0.011512643	Up-regulated	isoleucine--tRNA ligase
LOC105666679	1.501315228	0.011512643	Up-regulated	homeobox protein abdominal-B
LOC100642686	0.28277033	0.011544648	Up-regulated	ankyrin-3
LOC100646734	-0.157440766	0.011544648	Down-regulated	FYVE
LOC100646781	-0.757599036	0.011544648	Down-regulated	protein mab-21
LOC100649216	0.354041344	0.011544648	Up-regulated	breast cancer type 1 susceptibility protein homolog
LOC100650501	-0.17446153	0.011544648	Down-regulated	growth factor receptor-bound protein 2
LOC100651984	-0.372129652	0.011544648	Down-regulated	centrosomal protein of 152 kDa
LOC100647342	-0.313663034	0.011548477	Down-regulated	BTB/POZ domain-containing protein 7
LOC100649707	-0.142803769	0.01155068	Down-regulated	cyclic AMP-dependent transcription factor ATF-2
LOC100650392	0.43193319	0.011648887	Up-regulated	ATP-binding cassette sub-family A member 13
LOC100650721	-0.383311906	0.011656154	Down-regulated	XK-related protein 6
LOC100644875	-0.280539115	0.011682304	Down-regulated	solute carrier family 41 member 1
LOC100650363	-0.292387009	0.0117265	Down-regulated	conserved oligomeric Golgi complex subunit 8
LOC100650269	-0.225773386	0.011735233	Down-regulated	cysteine-rich PDZ-binding protein
LOC105665736	-0.252070592	0.011735233	Down-regulated	uncharacterized LOC105665736
LOC100645473	0.340478783	0.011746386	Up-regulated	codanin-1
LOC100650367	-0.232418739	0.011746386	Down-regulated	muskelin
LOC100651986	0.21121517	0.011746386	Up-regulated	chromodomain-helicase-DNA-binding protein 1
LOC100642357	0.446772766	0.011754544	Up-regulated	uncharacterized LOC100642357
LOC110119976	-0.481021413	0.011804888	Down-regulated	GPI mannosyltransferase 1
LOC100647549	0.290711526	0.011807718	Up-regulated	probablee cytochrome P450 LOC100647549
LOC100642615	0.677284459	0.011831697	Up-regulated	uncharacterized LOC100642615
LOC100644430	-0.20755269	0.011831697	Down-regulated	peptidyl-alpha-hydroxyglycine alpha-amidating lyase 1
LOC100647534	0.40300224	0.011831697	Up-regulated	proteasome assembly chaperone 1
LOC100651771	-0.406486046	0.01186699	Down-regulated	fez family zinc finger protein 2
LOC100648084	-0.284398432	0.011881276	Down-regulated	sprouty-related
LOC100650933	1.594673532	0.01188358	Up-regulated	clavesin-1
LOC100651019	0.406361731	0.01188358	Up-regulated	cadherin-99C
LOC100642630	-0.390330474	0.011919308	Down-regulated	zinc finger protein 385B
LOC100651348	0.494543093	0.011919308	Up-regulated	vascular endothelial growth factor receptor 3
LOC100648567	-0.415198964	0.011954512	Down-regulated	zwei Ig domain protein zig-8
LOC105667042	-0.577729657	0.011969571	Down-regulated	uncharacterized LOC105667042
LOC100642478	1.100861536	0.011975692	Up-regulated	myogenic-determination protein
LOC100650612	-0.204304938	0.011975692	Down-regulated	RUN domain-containing protein 1
LOC105666865	0.447301198	0.01202543	Up-regulated	protein tramtrack
LOC100652162	0.34919328	0.012085148	Up-regulated	uncharacterized LOC100652162
LOC100642568	0.341860548	0.012102298	Up-regulated	DNA-directed RNA polymerase I subunit RPA1
LOC100643341	-0.350740622	0.012103104	Down-regulated	uncharacterized LOC100643341
LOC100651367	0.279628951	0.012103104	Up-regulated	translation initiation factor IF-2
LOC100646171	0.274806082	0.012111468	Up-regulated	ribonuclease H1
LOC100647394	-0.401808475	0.012111468	Down-regulated	uncharacterized LOC100647394
LOC100651315	-0.292988855	0.012130467	Down-regulated	slowpoke-binding protein
LOC100648534	0.489518835	0.012184509	Up-regulated	uncharacterized LOC100648534
LOC100642857	0.427820053	0.012186876	Up-regulated	protein angel
LOC100645668	-0.239239397	0.012186999	Down-regulated	SWI/SNF-related matrix-associated actin-dependent regulator of chromatin
LOC100648815	0.273248569	0.012186999	Up-regulated	TRPL translocation defect protein 14
LOC100650809	-0.24060484	0.012204916	Down-regulated	clathrin light chain
LOC100645023	1.018451187	0.012208528	Up-regulated	serine protease filzig
LOC100646574	-0.262932408	0.012280286	Down-regulated	dynamin-1-like protein
LOC100650118	-0.353740917	0.0124348	Down-regulated	pyrimidodiazepine synthase
LOC100646726	-0.424557911	0.012441083	Down-regulated	protein neuralized
LOC100631066	0.300909971	0.012465481	Up-regulated	ribosomal protein S5a
LOC100646623	0.239134457	0.012510649	Up-regulated	calcium-binding mitochondrial carrier protein SCaMC-2
LOC100645101	0.757245594	0.01256651	Up-regulated	inducible metalloproteinase inhibitor protein
LOC100642655	-0.381259255	0.012572018	Down-regulated	facilitated trehalose transporter Tret1
LOC100649907	1.740773818	0.012577932	Up-regulated	general odorant-binding protein 56d
LOC100651805	-0.259709237	0.012607825	Down-regulated	laminin subunit gamma-1
LOC100644126	2.539973808	0.012713738	Up-regulated	uncharacterized LOC100644126

LOC100646087	0.350760751	0.012719084	Up-regulated	succinate dehydrogenase assembly factor 2
LOC100646177	0.177306087	0.012719084	Up-regulated	N-acetylgalactosaminyltransferase 6
LOC105667074	0.333924703	0.012719084	Up-regulated	glutathione S-transferase 1
LOC100652279	0.789652142	0.01272488	Up-regulated	uncharacterized LOC100652279
LOC100642429	-0.15675656	0.012755355	Down-regulated	protein EFR3 homolog cmp44E
LOC100644427	-0.389799361	0.012761017	Down-regulated	RNA-binding protein Musashi homolog 2
LOC100647641	0.430297826	0.012761017	Up-regulated	tumor protein p53-inducible nuclear protein 2
LOC100650271	-0.204743833	0.012761017	Down-regulated	glycerol kinase
LOC105666516	1.142936372	0.012761017	Up-regulated	carbonic anhydrase 2
LOC100631072	0.240226049	0.012802885	Up-regulated	NF-kappa-B transcription factor relish
LOC100649411	1.029434097	0.012804	Up-regulated	uncharacterized LOC100649411
LOC100646282	-0.433818755	0.012823536	Down-regulated	transmembrane protein 42
LOC100647777	0.140125978	0.012834624	Up-regulated	sporulation-specific protein 15
LOC100644910	-0.213215487	0.012851268	Down-regulated	phosphatidylinositol transfer protein
LOC100647716	-0.353952812	0.012851268	Down-regulated	uncharacterized LOC100647716
LOC100651262	0.697525743	0.012857049	Up-regulated	homeobox protein ARX
LOC100645095	0.394320584	0.012866117	Up-regulated	mitochondrial ribonuclease P protein 1 homolog
LOC100644615	-0.219966841	0.012875341	Down-regulated	CDK5RAP1-like protein
LOC100648481	-0.28144543	0.012875341	Down-regulated	kinesin-like protein unc-104
LOC100642752	-0.262433533	0.012884365	Down-regulated	uncharacterized LOC100642752
LOC100644919	-0.30083102	0.012884365	Down-regulated	guanine deaminase
LOC100647283	-0.311696103	0.012884365	Down-regulated	bidirectional sugar transporter N3
LOC100650804	-0.477273705	0.012884365	Down-regulated	uncharacterized LOC100650804
LOC100643306	0.300574017	0.012902959	Up-regulated	la-related protein 7
LOC100643809	-0.236763985	0.012902959	Down-regulated	sister chromatid cohesion protein PDS5 homolog B
LOC100645555	1.47251273	0.012902959	Up-regulated	nose resistant to fluoxetine protein 6
LOC100645675	-0.240450264	0.012902959	Down-regulated	beta-1
LOC100651691	0.174438056	0.012902959	Up-regulated	nuclear cap-binding protein subunit 1
LOC110119755	0.378213289	0.012902959	Up-regulated	uncharacterized LOC110119755
LOC100642969	0.255804693	0.012953879	Up-regulated	polycomb protein Sfmbt
LOC100644601	0.241940391	0.012997211	Up-regulated	replication termination factor 2
LOC100647011	0.157645674	0.012997211	Up-regulated	caseinolytic peptidase B protein homolog
LOC100645631	1.201041872	0.01299783	Up-regulated	sodium-independent sulfate anion transporter
LOC100643935	-0.229962306	0.013031593	Down-regulated	transcription elongation regulator 1
LOC100652006	-0.243285446	0.013036692	Down-regulated	serine/threonine-protein kinase Pak
LOC100645371	-0.233195438	0.013043769	Down-regulated	G-protein coupled receptor 143
LOC100644646	-0.192800315	0.013097093	Down-regulated	protein transport protein Sec23A
LOC100643264	0.555153035	0.013135921	Up-regulated	uncharacterized LOC100643264
LOC105665613	0.332632163	0.013135921	Up-regulated	sorting nexin-4
LOC100644716	1.621957394	0.013136038	Up-regulated	proton-coupled amino acid transporter-like protein pathetic
LOC100646898	-0.309629275	0.013184929	Down-regulated	intraflagellar transport protein 88 homolog
LOC100644378	-0.333225297	0.013210591	Down-regulated	brefeldin A-inhibited guanine nucleotide-exchange protein 3
LOC100650180	0.245439653	0.013215909	Up-regulated	TATA-box-binding protein
LOC100643398	0.254498207	0.013305428	Up-regulated	probable ATP-dependent RNA helicase DHX35
LOC100645590	0.34287299	0.013360676	Up-regulated	ras-related C3 botulinum toxin substrate 1
LOC100650297	-0.387059813	0.013360676	Down-regulated	synaptic vesicle membrane protein VAT-1 homolog-like
LOC100647770	-0.162849626	0.013458433	Down-regulated	nucleoporin SEH1
LOC100650855	-0.182498818	0.013458681	Down-regulated	serrate RNA effector molecule homolog
LOC100647577	-0.602645897	0.013472893	Down-regulated	RNA-binding protein MEX3B
LOC100648548	-0.525968332	0.013475501	Down-regulated	uncharacterized LOC100648548
LOC100648258	-0.501041068	0.013494352	Down-regulated	serine/threonine-protein kinase meng-po
LOC100649619	-0.21128624	0.013494352	Down-regulated	programmed cell death protein 2-like
LOC100645929	-0.276026516	0.013501314	Down-regulated	CDGSH iron-sulfur domain-containing protein 2 homolog
LOC100644058	-0.323114136	0.013556671	Down-regulated	uncharacterized LOC100644058
LOC100644580	-0.160553513	0.013637096	Down-regulated	4-hydroxybutyrate coenzyme A transferase
LOC100650334	-0.540370445	0.013665243	Down-regulated	LIM/homeobox protein Awh
LOC100650251	0.519088299	0.013697435	Up-regulated	plexin domain-containing protein 2
LOC105665636	0.962803408	0.013697435	Up-regulated	uncharacterized LOC105665636
LOC100643985	0.226762861	0.013703058	Up-regulated	DNA-directed RNA polymerase III subunit RPC5
LOC110119783	0.531660937	0.01371258	Up-regulated	uncharacterized LOC110119783
LOC100647643	-0.21179484	0.013752921	Down-regulated	UDP-glucose 6-dehydrogenase
LOC100648857	-0.276878336	0.01378719	Down-regulated	microtubule-associated protein Jupiter
LOC100650654	0.707942944	0.013848984	Up-regulated	allatotropin
LOC100645682	-0.278328069	0.013856099	Down-regulated	transcription initiation factor TFIID subunit 5
LOC100648241	-0.376540632	0.013856099	Down-regulated	HAUS augmin-like complex subunit 3
LOC100648602	0.536927441	0.013878647	Up-regulated	antichymotrypsin-2
LOC100645677	-0.400645541	0.013881622	Down-regulated	synaptonemal complex protein 1
LOC100646063	-0.459339012	0.013881622	Down-regulated	uncharacterized LOC100646063
LOC100650397	-0.259513613	0.013889943	Down-regulated	uncharacterized protein C4B3.18
LOC100644818	-0.216000004	0.013943867	Down-regulated	MOB kinase activator-like 1
LOC100647539	1.538384765	0.013948721	Up-regulated	cytochrome b5-related protein
LOC100643478	0.346837814	0.013954019	Up-regulated	peroxisomal membrane protein 2
LOC100645366	-0.475606788	0.013954189	Down-regulated	uncharacterized LOC100645366
LOC100644183	-0.301732341	0.013975871	Down-regulated	chondroitin sulfate synthase 2
LOC100643967	0.407163921	0.013991206	Up-regulated	cell cycle checkpoint protein RAD1
LOC100647288	-0.420736351	0.013991206	Down-regulated	protein THEM6
LOC100651552	0.734087143	0.013991206	Up-regulated	thyroid receptor-interacting protein 11
LOC100650580	0.937794068	0.013995719	Up-regulated	pickpocket protein 28
LOC100642459	0.688836089	0.014015823	Up-regulated	carbohydrate sulfotransferase 11
LOC105665754	0.296956138	0.014015823	Up-regulated	replication stress response regulator SDE2
LOC105666851	-0.693627705	0.014046525	Down-regulated	uncharacterized LOC105666851

LOC100649798	-0.194615753	0.014072201	Down-regulated	serine/arginine repetitive matrix protein 2
LOC100642401	-0.223302924	0.014076995	Down-regulated	cytochrome c oxidase subunit 5A
LOC100645635	-0.376033092	0.014076995	Down-regulated	uncharacterized LOC100645635
LOC100644921	0.290510686	0.014102151	Up-regulated	proton-coupled amino acid transporter-like protein CG1139
LOC100643547	-0.386732248	0.014103425	Down-regulated	protein hairy
LOC100648675	-0.281537648	0.014103425	Down-regulated	sodium/hydrogen exchanger 8
LOC100649567	-0.364284709	0.014103425	Down-regulated	guanine nucleotide-binding protein subunit beta-like protein 1
LOC100650656	-1.186268052	0.014103425	Down-regulated	uncharacterized LOC100650656
LOC100646889	-0.733846788	0.014121139	Down-regulated	collagen alpha-3(IX) chain
LOC100650936	0.389557289	0.01414254	Up-regulated	protein roadkill
LOC100643828	0.197163524	0.014180444	Up-regulated	voltage-dependent calcium channel gamma-7 subunit
LOC100645041	0.179549915	0.014183583	Up-regulated	coatomer subunit delta
LOC100648825	0.283936061	0.01419505	Up-regulated	protein KIAA0100
LOC100642551	0.199451494	0.014221619	Up-regulated	lipoyl synthase
LOC100647713	-0.392854666	0.014221619	Down-regulated	UPF0605 protein CG18335
LOC100644439	0.272629867	0.014225294	Up-regulated	myosin regulatory light chain sqh
LOC100650375	-0.212653493	0.014252043	Down-regulated	integrator complex subunit 2
LOC100647588	-0.214929579	0.014255045	Down-regulated	long-chain fatty acid transport protein 1
LOC100646409	0.332947262	0.014275903	Up-regulated	mannosyl-oligosaccharide 1
LOC100652120	1.508971366	0.014371764	Up-regulated	protein cortex
LOC100642601	-0.192304239	0.014380687	Down-regulated	pre-mRNA-splicing factor syf1 homolog
LOC100642191	0.224440719	0.014386458	Up-regulated	PH-interacting protein
LOC100650529	-0.417895635	0.014395533	Down-regulated	putative carbonic anhydrase-like protein 2
LOC100645243	0.257746389	0.014415662	Up-regulated	putative 28S ribosomal protein S5
LOC100643071	-0.737491855	0.014417315	Down-regulated	uncharacterized LOC100643071
LOC100642901	0.202716561	0.014538469	Up-regulated	NADH-quinone oxidoreductase subunit I
LOC100647833	0.775548003	0.014636147	Up-regulated	protein Wnt-6
LOC100642552	0.322340423	0.014644471	Up-regulated	60S ribosomal protein L13a
LOC100650341	-0.240691436	0.014644471	Down-regulated	polyamine-transporting ATPase 13A3
LOC100650790	0.244659149	0.014644471	Up-regulated	cytoplasmic dynein 2 intermediate chain 2
LOC100651536	-0.322485603	0.014644471	Down-regulated	follistatin-related protein 5
LOC100644192	0.213144554	0.014649063	Up-regulated	mothers against decapentaplegic homolog 4
LOC100652212	-0.289176901	0.014649063	Down-regulated	methionine--tRNA ligase
LOC100645283	-0.319899302	0.014689381	Down-regulated	uncharacterized protein C594.04c
LOC100648769	0.355995672	0.014689381	Up-regulated	uncharacterized LOC100648769
LOC100643719	0.230241946	0.014739923	Up-regulated	protein BCCIP homolog
LOC100649782	-0.156220369	0.014775147	Down-regulated	pleckstrin homology domain-containing family M member 2
LOC105665862	-0.246972076	0.01481142	Down-regulated	ras-related protein Rab-8A
LOC100646059	-0.432933349	0.014842123	Down-regulated	receptor-type guanylate cyclase gcy-14
LOC100648652	1.645169293	0.014876386	Up-regulated	uncharacterized LOC100648652
LOC100650945	1.044610969	0.014889678	Up-regulated	phospholipase A1
LOC100645495	-0.472481398	0.01489939	Down-regulated	bolA-like protein DDB_G0274169
LOC105665659	0.380153078	0.01489939	Up-regulated	coiled-coil domain-containing protein 97
LOC100646748	-0.218576803	0.01496559	Down-regulated	GTP-binding nuclear protein Ran
LOC100649050	-0.181042821	0.01496559	Down-regulated	zinc finger CCCH-type with G patch domain-containing protein
LOC100643087	0.256154196	0.015002493	Up-regulated	40S ribosomal protein S4
LOC100650499	-0.196570153	0.015002493	Down-regulated	gamma-tubulin complex component 3 homolog
LOC100645833	0.218445833	0.015005231	Up-regulated	DNA-directed RNA polymerase III subunit RPC3
LOC100642627	0.239786116	0.015006269	Up-regulated	rho guanine nucleotide exchange factor 7
LOC100651054	-0.481014837	0.015018507	Down-regulated	major facilitator superfamily domain-containing protein 6
LOC100644315	-0.378277592	0.015037582	Down-regulated	mitochondrial ornithine transporter 1
LOC100650780	-0.271129821	0.015054045	Down-regulated	sodium leak channel NALCN
LOC100646061	-0.34474088	0.015194716	Down-regulated	hemicentin-2
LOC100649676	-0.290003081	0.015299774	Down-regulated	WD repeat-containing protein on Y chromosome
LOC100648354	-0.326707812	0.015371163	Down-regulated	uncharacterized LOC100648354
LOC100631077	0.312283425	0.015381155	Up-regulated	ribosomal protein L13
LOC100646149	-0.403914668	0.01544097	Down-regulated	uncharacterized LOC100646149
LOC100643584	-0.213680319	0.015447765	Down-regulated	protein kinase C and casein kinase substrate in neurons protein 1
LOC110120016	1.134569182	0.015463018	Up-regulated	uncharacterized LOC110120016
LOC100650255	-0.288333433	0.015477519	Down-regulated	integrator complex subunit 13
LOC100649236	0.490420995	0.015489339	Up-regulated	ATP-dependent DNA helicase Q5
LOC100652040	-0.471184259	0.015516305	Down-regulated	calpain-C
LOC100644471	-0.191299902	0.015533631	Down-regulated	S-adenosylmethionine decarboxylase proenzyme
LOC100649452	0.322132106	0.015533631	Up-regulated	uncharacterized LOC100649452
LOC100646777	0.504795174	0.015549734	Up-regulated	transient receptor potential channel pyrexia
LOC100649759	0.390411644	0.015559349	Up-regulated	REPTOR-binding partner
LOC105666342	0.311389781	0.015572053	Up-regulated	60S ribosomal protein L18
LOC100647007	-0.386220362	0.015579029	Down-regulated	uncharacterized LOC100647007
LOC100642281	0.321432635	0.015583895	Up-regulated	mediator of DNA damage checkpoint protein 1
LOC100646892	1.076310003	0.015587543	Up-regulated	homeobox protein aristaless
LOC100648275	0.431685939	0.015587543	Up-regulated	DNA topoisomerase 2-binding protein 1
LOC100648118	-0.265531072	0.015615126	Down-regulated	bcl-2-related ovarian killer protein
LOC100643805	0.287380854	0.015666538	Up-regulated	arrestin domain-containing protein 17
LOC100649384	-0.376526179	0.015687288	Down-regulated	neprilysin-4
LOC100651414	0.164117956	0.015688186	Up-regulated	uncharacterized LOC100651414
LOC100647537	0.38218094	0.015706855	Up-regulated	uncharacterized LOC100647537
LOC100644610	-0.34563016	0.015720552	Down-regulated	UDP-glucose 4-epimerase
LOC100647195	0.189387482	0.015808802	Up-regulated	glycogen [starch] synthase
LOC105665675	-0.532166801	0.015844184	Down-regulated	uncharacterized LOC105665675
LOC100643579	0.22306181	0.015885129	Up-regulated	zinc finger protein 622
LOC105666621	0.304734999	0.015893269	Up-regulated	27 kDa hemolymph protein

LOC100644251	-0.195351196	0.015960999	Down-regulated	coiled-coil domain-containing protein 43
LOC100649572	-0.189662308	0.015960999	Down-regulated	kinesin-like protein KIF21A
LOC100646067	-0.270893508	0.015971504	Down-regulated	serine/arginine repetitive matrix protein 1
LOC100642347	-0.262293792	0.016087845	Down-regulated	transcription factor MafG
LOC100648833	0.18600686	0.016087845	Up-regulated	antichymotrypsin-2
LOC100649855	0.754268588	0.016087845	Up-regulated	putative polypeptide N-acetylgalactosaminyltransferase 9
LOC100643795	-0.173329481	0.016112396	Down-regulated	nucleolar complex protein 4 homolog B
LOC100642620	-0.297742113	0.0161156	Down-regulated	O-phosphoseryl-tRNA(Sec) selenium transferase
LOC100643195	0.556350112	0.0161156	Up-regulated	aprataxin and PNK-like factor
LOC100647132	0.110331244	0.0161156	Up-regulated	bone morphogenetic protein receptor type-2
LOC105665630	0.268536049	0.0161156	Up-regulated	uncharacterized LOC105665630
LOC100648778	-0.164666636	0.016135375	Down-regulated	huntingtin-interacting protein 1
LOC100650270	-0.228147252	0.016147499	Down-regulated	protein mini spindles
LOC100643912	-0.230926288	0.016220933	Down-regulated	nuclear pore complex protein Nup160 homolog
LOC100645257	0.527992841	0.016220933	Up-regulated	mucin-3A
LOC100650768	-0.228660016	0.016220933	Down-regulated	lactosylceramide 4-alpha-galactosyltransferase
LOC105667183	0.235456346	0.016220933	Up-regulated	splicing factor 3B subunit 5
LOC100648858	0.144913413	0.016228211	Up-regulated	V-type proton ATPase subunit E
LOC100647300	-0.209910369	0.016257666	Down-regulated	F-box/SPRY domain-containing protein 1
LOC105666190	0.200283787	0.016313619	Up-regulated	protein YIPF5 homolog
LOC100643877	-0.258348055	0.016434074	Down-regulated	leucine-rich repeats and immunoglobulin-like domains protein 3
LOC100643143	-0.497371496	0.016527322	Down-regulated	protein borderless
LOC100651544	0.304520279	0.016527322	Up-regulated	40S ribosomal protein S7
LOC100644464	1.238628534	0.016583464	Up-regulated	fibrillin-2
LOC100651117	-0.338385541	0.016631293	Down-regulated	mitochondrial fission process protein 1
LOC100648603	0.313133658	0.016636811	Up-regulated	putative phosphoenolpyruvate synthase
LOC100646081	-0.239962375	0.016662678	Down-regulated	serine/threonine-protein kinase D3
LOC100644202	0.923642639	0.016674594	Up-regulated	uncharacterized LOC100644202
LOC100644864	-0.399155976	0.016686396	Down-regulated	carboxypeptidase Q
LOC100648149	0.33157454	0.016686396	Up-regulated	dnaJ homolog subfamily B member 6
LOC100643824	0.443116898	0.016696459	Up-regulated	long-chain-fatty-acid--CoA ligase 1
LOC100648588	-0.228435644	0.016696459	Down-regulated	8-oxo-dGDP phosphatase NUDT18
LOC100645875	0.227892991	0.01672748	Up-regulated	mucin-17
LOC100647114	0.730662737	0.01672748	Up-regulated	scavenger receptor class B member 1
LOC100648461	0.373711872	0.01672748	Up-regulated	60S ribosomal protein L26
LOC100644524	-0.473967709	0.016783955	Down-regulated	uncharacterized LOC100644524
LOC100643212	0.51571405	0.016864835	Up-regulated	dynein axonemal heavy chain 3
LOC100644632	-0.362045001	0.016875263	Down-regulated	solute carrier family 41 member 1
LOC110119937	0.450093101	0.016903185	Up-regulated	nuclear pore complex protein DDB_G0274915-like
LOC100644020	-0.195512505	0.016934572	Down-regulated	protein abrupt
LOC100644518	0.567391567	0.016934572	Up-regulated	homeobox protein homothorax
LOC100642445	-0.234190324	0.016954789	Down-regulated	tRNA-specific adenosine deaminase 1
LOC100648958	-0.216941054	0.016954789	Down-regulated	inositol-pentakisphosphate 2-kinase
LOC100649209	0.275855821	0.016969782	Up-regulated	transmembrane protein 216
LOC100651542	-0.675004896	0.016969782	Down-regulated	dendritic arbor reduction protein 1
LOC100652033	0.211675585	0.017060926	Up-regulated	mitochondrial E3 ubiquitin protein ligase 1
LOC100649677	-0.244705314	0.017077844	Down-regulated	uncharacterized LOC100649677
LOC100645931	0.207632423	0.017150734	Up-regulated	eukaryotic translation initiation factor 4 gamma 3
LOC100642341	0.364054657	0.017177129	Up-regulated	zinc finger protein 511
LOC100642333	0.312069068	0.017181025	Up-regulated	G1/S-specific cyclin-D2
LOC100643391	-0.444087228	0.017181025	Down-regulated	zwei Ig domain protein zig-8
LOC100648416	0.283795102	0.017200754	Up-regulated	adenosine kinase 1
LOC100644817	-0.328365806	0.017242552	Down-regulated	heat shock protein 60A
LOC100647013	-0.376083251	0.017242552	Down-regulated	gamma-aminobutyric acid type B receptor subunit 1
LOC100648255	-0.365870343	0.017250015	Down-regulated	uncharacterized LOC100648255
LOC100645946	-0.485833419	0.017264474	Down-regulated	hemicentin-2
LOC100649271	0.182273165	0.017264474	Up-regulated	NADH dehydrogenase [ubiquinone] 1 beta subcomplex subunit 5
LOC100648212	0.941020254	0.017269583	Up-regulated	putative inorganic phosphate cotransporter
LOC105666596	-0.786898864	0.017346203	Down-regulated	uncharacterized LOC105666596
LOC100645383	0.191071858	0.01739941	Up-regulated	vacuolar protein sorting-associated protein 52 homolog
LOC100651150	0.141663654	0.017454953	Up-regulated	rab3 GTPase-activating protein non-catalytic subunit
LOC100647766	-0.31992918	0.017477577	Down-regulated	mitochondrial import inner membrane translocase subunit Tim13
LOC100650554	0.381220224	0.017477577	Up-regulated	ribonuclease H2 subunit B
LOC100646891	0.490203277	0.017551582	Up-regulated	SET and MYND domain-containing protein 4-like
LOC100651334	-0.778578191	0.01757719	Down-regulated	solute carrier organic anion transporter family member 74D
LOC100643270	0.241012683	0.017608103	Up-regulated	phosphorylated adapter RNA export protein
LOC100645216	0.360178385	0.017608124	Up-regulated	uncharacterized LOC100645216
LOC100650105	-0.239421295	0.017642551	Down-regulated	Golgi integral membrane protein 4
LOC100649534	0.45277082	0.017661705	Up-regulated	torso-like protein
LOC100647869	-0.217252706	0.017720168	Down-regulated	uncharacterized LOC100647869
LOC100652116	0.226672819	0.017755409	Up-regulated	serine/threonine-protein phosphatase PGAM5
LOC100648943	-0.134273956	0.017823308	Down-regulated	syntaxin-7
LOC100647779	-1.360428738	0.017826444	Down-regulated	pickpocket protein 28
LOC100650480	-0.446332233	0.017831015	Down-regulated	E3 ubiquitin-protein ligase TRIM9
LOC100649295	0.136676065	0.017831649	Up-regulated	protoheme IX farnesyltransferase
LOC100643388	-0.244371014	0.017858952	Down-regulated	polyphosphoinositide phosphatase
LOC100648392	-0.144606064	0.017942744	Down-regulated	ral GTPase-activating protein subunit beta
LOC100648780	-0.390075873	0.017942744	Down-regulated	proton channel OtopLc
LOC100645117	0.774490822	0.017958423	Up-regulated	uncharacterized LOC100645117
LOC100647861	-0.288835034	0.017972676	Down-regulated	fasciculation and elongation protein zeta-2
LOC100644462	-0.612523818	0.017972987	Down-regulated	facilitated trehalose transporter Tret1

LOC100646003	-0.348378468	0.018034274	Down-regulated	10 kDa heat shock protein
LOC100645825	0.244899189	0.018035474	Up-regulated	dynactin subunit 2
LOC100646053	1.448800628	0.018042891	Up-regulated	uncharacterized LOC100646053
LOC100647293	0.318315762	0.018045547	Up-regulated	uncharacterized LOC100647293
LOC100651857	0.231076853	0.018061339	Up-regulated	uncharacterized LOC100651857
LOC100644204	0.218103882	0.018090766	Up-regulated	protein SPT2 homolog
LOC100649982	0.249590895	0.018090766	Up-regulated	molybdenum cofactor biosynthesis protein 1
LOC100647169	0.268390958	0.018103596	Up-regulated	AN1-type zinc finger protein 5
LOC110119158	0.495110903	0.018115239	Up-regulated	uncharacterized LOC110119158
LOC100646854	0.239773541	0.018133329	Up-regulated	formylglycine-generating enzyme
LOC100645579	-0.512339766	0.018135267	Down-regulated	stimulator of interferon genes protein homolog
LOC100643175	-0.335265617	0.018159128	Down-regulated	telomerase Cajal body protein 1
LOC100649721	-0.4271286	0.018220093	Down-regulated	lysophosphatidylserine lipase ABHD12
LOC100650108	0.517392811	0.018220093	Up-regulated	multidrug resistance protein homolog 49
LOC100644266	-0.134568989	0.018231319	Down-regulated	protein Aster-B
LOC100648038	-0.12245379	0.018236275	Down-regulated	dnaJ homolog subfamily B member 12
LOC100648183	-0.504004391	0.018236275	Down-regulated	enhancer of split m7 protein
LOC100650151	0.224090967	0.018236275	Up-regulated	F-box/WD repeat-containing protein 9
LOC100651466	-0.259936179	0.018247667	Down-regulated	nonsense-mediated mRNA decay factor SMG9
LOC105667123	1.819941348	0.018423559	Up-regulated	uncharacterized LOC105667123
LOC105666304	-0.63576204	0.018426853	Down-regulated	pre-mRNA-splicing factor CWC22 homolog
LOC100648266	-0.213771135	0.018501496	Down-regulated	alanine aminotransferase 1
LOC100650940	-0.216783747	0.018647132	Down-regulated	uncharacterized LOC100650940
LOC100644446	-0.195102817	0.018707541	Down-regulated	melanotransferrin
LOC105666631	0.3434408	0.018866356	Up-regulated	activating signal cointegrator 1 complex subunit 1
LOC100646029	-0.313609337	0.019050816	Down-regulated	uncharacterized LOC100646029
LOC100650976	-0.209574147	0.019050816	Down-regulated	WD repeat
LOC105666191	0.258783332	0.019050816	Up-regulated	nucleolar protein 58-like
LOC100651047	0.319290552	0.019054247	Up-regulated	MKI67 FHA domain-interacting nucleolar phosphoprotein
LOC100648623	0.654144842	0.019076811	Up-regulated	uncharacterized LOC100648623
LOC100648890	0.303261732	0.019076811	Up-regulated	60S ribosomal protein L18a
LOC100650459	-0.11909363	0.019076811	Down-regulated	membrane-associated protein Hem
LOC100650633	1.002481743	0.019076811	Up-regulated	probable DNA replication complex GINS protein PSF2
LOC100652009	0.380853197	0.019076811	Up-regulated	DNA polymerase alpha catalytic subunit
LOC105666583	-0.414825952	0.019076811	Down-regulated	MAP kinase-activating death domain protein
LOC100650559	0.212042457	0.019258981	Up-regulated	ankyrin repeat and LEM domain-containing protein 2
LOC100646144	0.726310072	0.019477158	Up-regulated	histone H2A
LOC100645994	-0.200391133	0.019481264	Down-regulated	uncharacterized LOC100645994
LOC105665631	-0.278893505	0.019481264	Down-regulated	iron-sulfur protein NUBPL
LOC100651576	0.292384112	0.019496466	Up-regulated	RING finger protein 121
LOC100649141	0.216392301	0.01950878	Up-regulated	citron Rho-interacting kinase
LOC100643590	-1.145339727	0.019606057	Down-regulated	uncharacterized LOC100643590
LOC100650793	0.181669476	0.019606057	Up-regulated	WAS/WASL-interacting protein family member 1
LOC100646802	-0.13705716	0.019629841	Down-regulated	lipoma-preferred partner homolog
LOC100642450	-0.347642295	0.019644796	Down-regulated	lysophospholipid acyltransferase 6
LOC100648479	-0.424596141	0.019651542	Down-regulated	G-protein coupled receptor moody
LOC100642899	-0.221986972	0.019664116	Down-regulated	protein artichoke
LOC100645382	-0.232059894	0.019673664	Down-regulated	Golgi SNAP receptor complex member 2
LOC100650742	-0.223746721	0.019673867	Down-regulated	lysophospholipid acyltransferase 7
LOC100646372	-0.391927875	0.019681127	Down-regulated	leukocyte tyrosine kinase receptor
LOC100643061	0.262427514	0.019806908	Up-regulated	WD40 repeat-containing protein SMU1
LOC100650549	-0.455429504	0.019834298	Down-regulated	high-affinity choline transporter 1
LOC100643597	-0.24952393	0.019868669	Down-regulated	synembryn-A
LOC100651434	0.408594188	0.019872971	Up-regulated	40S ribosomal protein S3-like
LOC100647490	-0.181974386	0.019955155	Down-regulated	uncharacterized LOC100647490
LOC100648993	0.213471283	0.019955155	Up-regulated	galactose mutarotase
LOC100645585	0.674501444	0.020017333	Up-regulated	uncharacterized LOC100645585
LOC100644686	0.212030365	0.02003009	Up-regulated	la protein homolog
LOC100649028	-0.224651254	0.02004462	Down-regulated	32 kDa beta-galactoside-binding lectin
LOC100648710	0.185547806	0.020121294	Up-regulated	ubiquitin-conjugating enzyme E2 W
LOC100645040	-0.443724917	0.020168481	Down-regulated	paired box protein Pax-6
LOC100652301	1.656415011	0.0202223	Up-regulated	serine protease inhibitor 3/4
LOC100651767	-0.364056665	0.02032436	Down-regulated	uncharacterized LOC100651767
LOC100643268	-0.407023403	0.020333494	Down-regulated	zwei Ig domain protein zig-8
LOC100645705	-0.180745387	0.020337035	Down-regulated	TBC1 domain family member 9
LOC100642611	-0.370107086	0.020370684	Down-regulated	retinal guanylyl cyclase 2
LOC100645846	-0.406305559	0.02038439	Down-regulated	ephrin type-B receptor 1-B
LOC100648189	0.466167537	0.020496212	Up-regulated	RNA/RNP complex-1-interacting phosphatase
LOC100649312	0.214292983	0.020496212	Up-regulated	max-like protein X
LOC100652093	-0.298934102	0.020496212	Down-regulated	DNA replication complex GINS protein SLD5
LOC105666115	-0.415348095	0.020496212	Down-regulated	uncharacterized LOC105666115
LOC100651620	-0.199965597	0.02055537	Down-regulated	golgin subfamily A member 7
LOC100652325	0.228344207	0.02055537	Up-regulated	ras-related protein rab-6.2
LOC100648430	-0.367880726	0.020558542	Down-regulated	GTPase-activating Rap/Ran-GAP domain-like protein 3
LOC100643325	-0.367719611	0.020572602	Down-regulated	enhancer of rudimentary homolog
LOC100648060	-0.157761395	0.020572602	Down-regulated	protein pellino
LOC100648143	-0.206815456	0.020580773	Down-regulated	ATP-dependent (S)-NAD(P)H-hydrate dehydratase
LOC100648220	-0.22407162	0.020632634	Down-regulated	putative RNA-binding protein Luc7-like 2
LOC100648408	0.153546699	0.02071854	Up-regulated	lethal(3)malignant brain tumor-like protein 3
LOC100645920	-0.326388105	0.020728124	Down-regulated	probable helicase with zinc finger domain
LOC105666362	-1.284825998	0.020729564	Down-regulated	uncharacterized LOC105666362

LOC100647000	0.200225949	0.020768865	Up-regulated	dedicator of cytokinesis protein 1
LOC100650074	0.497031627	0.020773534	Up-regulated	heat shock factor 2-binding protein
LOC100649287	0.170081983	0.02081308	Up-regulated	ubiquitin-conjugating enzyme E2-17 kDa
LOC110119167	0.161826193	0.02081308	Up-regulated	uncharacterized LOC110119167
LOC100649687	0.396805333	0.020820198	Up-regulated	uncharacterized LOC100649687
LOC100644887	0.194731742	0.020827332	Up-regulated	5'-3' exoribonuclease 2 homolog
LOC105666103	-0.814390971	0.020827332	Down-regulated	uncharacterized LOC105666103
LOC100645625	-0.272249217	0.020837384	Down-regulated	tax1-binding protein 3 homolog
LOC100645706	-0.187796082	0.020927343	Down-regulated	receptor-mediated endocytosis protein 6 homolog
LOC100645142	0.170887417	0.020950665	Up-regulated	translation initiation factor eIF-2B subunit delta
LOC100648151	-0.20064373	0.021081562	Down-regulated	ataxin-2-like protein
LOC100646590	-0.369491432	0.021082508	Down-regulated	GPI mannosyltransferase 4
LOC100631060	-0.511025091	0.021100376	Down-regulated	period
LOC100645297	0.326534178	0.021153455	Up-regulated	nitric oxide-associated protein 1
LOC100645982	0.225210342	0.021187858	Up-regulated	solute carrier family 12 member 8
LOC100648049	-0.155821311	0.021187858	Down-regulated	protein MTO1 homolog
LOC100651055	0.260487315	0.021187858	Up-regulated	selenocysteine insertion sequence-binding protein 2-like
LOC100643404	-0.257944325	0.021204905	Down-regulated	calcium/calmodulin-dependent 3'
LOC100650877	0.233110444	0.021247338	Up-regulated	protein CNPPD1
LOC100650050	-0.241271538	0.021320511	Down-regulated	eukaryotic translation initiation factor 5B
LOC100651992	-0.139181223	0.021332198	Down-regulated	FACT complex subunit Ssrp1
LOC100642995	0.670521661	0.021364401	Up-regulated	1-acyl-sn-glycerol-3-phosphate acyltransferase gamma
LOC100646608	0.198965368	0.021409689	Up-regulated	extended synaptotagmin-2
LOC100649054	0.720094432	0.021409689	Up-regulated	uncharacterized LOC100649054
LOC100646649	0.211264825	0.021439752	Up-regulated	ragulator complex protein LAMTOR1
LOC110119636	-0.755483462	0.021439752	Down-regulated	uncharacterized LOC110119636
LOC100642402	-0.382757745	0.021525156	Down-regulated	uncharacterized LOC100642402
LOC100652110	-0.184756711	0.021561652	Down-regulated	NEDD8-activating enzyme E1 catalytic subunit
LOC100644840	0.166835411	0.021680397	Up-regulated	exportin-2
LOC100650001	-0.428880222	0.021705089	Down-regulated	forkhead box protein D3-like
LOC100649165	-0.458610095	0.021717072	Down-regulated	rho GTPase-activating protein 20
LOC100645184	-0.301984141	0.021739578	Down-regulated	protein Cep78 homolog
LOC105666529	0.785867112	0.021739578	Up-regulated	aquaporin-11
LOC110119859	1.053684118	0.021739578	Up-regulated	uncharacterized LOC110119859
LOC100645752	0.622315523	0.021751123	Up-regulated	uncharacterized protein YER152C-like
LOC105665786	0.52645689	0.021844346	Up-regulated	chymotrypsin-2
LOC100648741	0.586077818	0.021972076	Up-regulated	protein PF3D7_1417600
LOC100652132	0.204551864	0.02199027	Up-regulated	importin-11
LOC105666405	-0.464330617	0.02199027	Down-regulated	uncharacterized LOC105666405
LOC100651254	1.259880322	0.021998887	Up-regulated	clavesin-1
LOC100645542	-0.126632057	0.02206338	Down-regulated	vacuolar protein sorting-associated protein 37A
LOC100647547	-0.447653659	0.02206338	Down-regulated	uncharacterized LOC100647547
LOC100645213	-0.273854903	0.022152337	Down-regulated	protein tramtrack
LOC100650989	-0.262268606	0.022207523	Down-regulated	uncharacterized LOC100650989
LOC100646373	-0.894473222	0.022236504	Down-regulated	inositol oxygenase
LOC100642788	0.128760024	0.022270774	Up-regulated	uncharacterized LOC100642788
LOC100650957	-0.229209813	0.022283248	Down-regulated	heterogeneous nuclear ribonucleoprotein R
LOC100647307	-0.175063151	0.022316038	Down-regulated	blood vessel epicardial substance
LOC100647622	0.302627918	0.022342647	Up-regulated	IQ motif and SEC7 domain-containing protein 2
LOC100649673	0.089319737	0.022365296	Up-regulated	cytoplasmic dynein 1 light intermediate chain 1
LOC100649104	-0.361117031	0.022387491	Down-regulated	electron transfer flavoprotein beta subunit lysine methyltransferase
LOC100645279	-0.295468462	0.022421227	Down-regulated	retinol dehydrogenase 11
LOC100642279	0.763457824	0.022447208	Up-regulated	uncharacterized LOC100642279
LOC100642532	-0.164175294	0.022496598	Down-regulated	polycomb protein Asx
LOC100643384	0.441996231	0.022546956	Up-regulated	eukaryotic translation initiation factor 4E-binding protein
LOC100647256	0.59647969	0.022589392	Up-regulated	DNA replication licensing factor Mcm2
LOC100644077	0.300599895	0.022640371	Up-regulated	proteasome subunit beta type-4
LOC100647346	0.237859677	0.022641445	Up-regulated	DCN1-like protein 1
LOC100649025	-0.364496774	0.022694985	Down-regulated	uncharacterized LOC100649025
LOC100631067	-0.505005604	0.022709742	Down-regulated	toll like receptor 6
LOC100646212	-0.124211036	0.022862459	Down-regulated	twinfilin
LOC100646141	-0.49690885	0.022869253	Down-regulated	hemicentin-1
LOC100646086	0.229589637	0.022907997	Up-regulated	mediator of RNA polymerase II transcription subunit 15
LOC100643697	0.167776694	0.022943707	Up-regulated	rabenosyn-5
LOC100649061	0.199435272	0.023000554	Up-regulated	corepressor interacting with RBPJ 1
LOC100652233	-0.227270177	0.023094556	Down-regulated	cysteine--tRNA ligase
LOC100647578	0.509895215	0.023112685	Up-regulated	probable cytochrome P450 305a1 LOC100647578
LOC100651316	0.194854435	0.023145203	Up-regulated	ras-related protein Rab-9A
LOC100650010	0.551193913	0.02314824	Up-regulated	echinoderm microtubule-associated protein-like 2
LOC100644541	0.20177805	0.02318391	Up-regulated	probable ATP-dependent RNA helicase DDX49
LOC100650007	0.180508454	0.02318391	Up-regulated	NCK-interacting protein with SH3 domain
LOC100647334	-0.213679006	0.023199775	Down-regulated	ribonuclease P protein subunit p14
LOC100651245	-0.301436418	0.023199775	Down-regulated	glutathione S-transferase
LOC100651629	0.194133623	0.023199775	Up-regulated	RNA polymerase II subunit A C-terminal domain phosphatase
LOC100642757	0.317640079	0.023251596	Up-regulated	proteasome subunit beta type-1
LOC100644380	-0.258372717	0.023297909	Down-regulated	syndecan
LOC100650442	0.189972685	0.023359615	Up-regulated	zinc finger protein on ecdysone puffs
LOC100644975	0.213305466	0.023425219	Up-regulated	peroxiredoxin-6
LOC100647589	0.400839589	0.02344679	Up-regulated	Y-box-binding protein 1
LOC100652107	-0.282609901	0.023463983	Down-regulated	integrator complex subunit 7
LOC100649659	-0.182055845	0.02350824	Down-regulated	histone deacetylase complex subunit SAP130-A

LOC100646078	1.159575072	0.023614631	Up-regulated	trehalase
LOC100649290	0.336409098	0.023868972	Up-regulated	leucine-rich repeat-containing protein 49
LOC100647478	0.364975395	0.023875877	Up-regulated	baculoviral IAP repeat-containing protein 5
LOC100642640	-0.254793626	0.023915273	Down-regulated	myotubularin-related protein 14
LOC100642693	0.207920508	0.023915273	Up-regulated	CCR4-NOT transcription complex subunit 1
LOC100645062	3.057433102	0.023915273	Up-regulated	uncharacterized LOC100645062
LOC100651252	-0.171940627	0.023915273	Down-regulated	ATP-dependent RNA helicase WM6
LOC100651926	-0.26585033	0.023951481	Down-regulated	corrinoid adenosyltransferase
LOC100646731	0.282136137	0.024062866	Up-regulated	RNA-binding protein 8A
LOC100647941	0.545887458	0.024115907	Up-regulated	uncharacterized protein DDB_G0283697
LOC100648960	-0.421204805	0.024123809	Down-regulated	dynein axonemal heavy chain 7
LOC100650895	-0.224266942	0.02413868	Down-regulated	reactive oxygen species modulator 1
LOC100643245	0.245169727	0.024154531	Up-regulated	RAB6A-GEF complex partner protein 2
LOC100642355	-0.327843676	0.024214536	Down-regulated	cell adhesion molecule 2
LOC100646840	-0.308926258	0.024253449	Down-regulated	DNA polymerase theta
LOC100651147	0.328036668	0.024273722	Up-regulated	uncharacterized LOC100651147
LOC100647624	-0.524648339	0.024299032	Down-regulated	acetylcholine receptor subunit alpha-like 1
LOC100646721	0.415278077	0.024299472	Up-regulated	venom acid phosphatase Acph-1
LOC100647392	-0.162007858	0.024299472	Down-regulated	myotrophin
LOC100643224	-0.701706444	0.02434972	Down-regulated	homeotic protein Sex combs reduced
LOC100644534	-0.325370326	0.02434972	Down-regulated	DET1- and DDB1-associated protein 1
LOC100645295	0.329152221	0.024419603	Up-regulated	sialin
LOC100646730	0.503771032	0.024465347	Up-regulated	serine protease snake
LOC100642339	0.379455274	0.024474806	Up-regulated	endoribonuclease CG2145
LOC100644368	-0.199809211	0.024474806	Down-regulated	protein HBS1
LOC105666177	-0.335818373	0.024587784	Down-regulated	uncharacterized LOC105666177
LOC100646812	0.260506451	0.024598237	Up-regulated	TAR DNA-binding protein 43
LOC100649802	-0.245557116	0.024604999	Down-regulated	trichohyalin
LOC100646913	0.243711108	0.024708252	Up-regulated	uncharacterized LOC100646913
LOC100645960	0.661330451	0.024725422	Up-regulated	protein APCDD1
LOC100643084	0.180104332	0.024807272	Up-regulated	U3 small nucleolar ribonucleoprotein protein MPP10
LOC100644487	0.23956409	0.024879488	Up-regulated	nucleolar protein 14 homolog
LOC100648477	0.303479574	0.024879488	Up-regulated	major facilitator superfamily domain-containing protein 6
LOC100648133	1.278503138	0.024880677	Up-regulated	protein mesh
LOC105666029	0.471066822	0.024880677	Up-regulated	putative zinc finger protein 702
LOC100647405	-0.322012641	0.0249533	Down-regulated	actin-binding LIM protein 2
LOC100649774	0.113552608	0.02501274	Up-regulated	calcium permeable stress-gated cation channel 1
LOC100645322	0.74881087	0.025069944	Up-regulated	doublesex- and mab-3-related transcription factor A2
LOC100647976	-0.262654549	0.025107957	Down-regulated	phosphatidylinositol-glycan biosynthesis class X protein
LOC100650185	-0.345922822	0.025107957	Down-regulated	phospholipase B1
LOC105666455	-1.184513925	0.025134494	Down-regulated	uncharacterized LOC105666455
LOC100651294	-0.271689553	0.025145035	Down-regulated	HIV Tat-specific factor 1 homolog
LOC105665986	-0.137597034	0.025193969	Down-regulated	charged multivesicular body protein 5
LOC100651994	0.205121506	0.025230771	Up-regulated	SPRY domain-containing SOCS box protein 3
LOC100642631	0.281871125	0.025237188	Up-regulated	uncharacterized protein PF3D7_1120600
LOC100649171	-0.329412655	0.025278124	Down-regulated	synaptotagmin-14
LOC100646461	0.166377163	0.025336312	Up-regulated	ribosomal protein S6 kinase beta-1
LOC100643569	-0.48636955	0.02558136	Down-regulated	nephrin
LOC100652178	0.161784515	0.02558136	Up-regulated	guanine nucleotide-binding protein-like 3 homolog
LOC105665848	0.23426593	0.02558136	Up-regulated	serine/threonine-protein phosphatase PGAM5
LOC105666963	-0.777982831	0.02559835	Down-regulated	uncharacterized LOC105666963
LOC100642809	-0.157995295	0.025599692	Down-regulated	actin-related protein 2/3 complex subunit 1A-A
LOC110119206	-0.347404359	0.025634615	Down-regulated	uncharacterized LOC110119206
LOC100642584	-0.313899986	0.02564654	Down-regulated	uncharacterized LOC100642584
LOC100651437	-0.24509681	0.02564654	Down-regulated	cytochrome b5
LOC100644364	0.311465885	0.02573945	Up-regulated	uncharacterized LOC100644364
LOC100650519	0.19647858	0.025802616	Up-regulated	cytochrome c oxidase subunit NDUFA4
LOC100649820	0.190724853	0.025828757	Up-regulated	protein cramped-like
LOC100643082	0.653716927	0.025884466	Up-regulated	UPF0489 protein C5orf22 homolog
LOC100652303	0.195533821	0.025884466	Up-regulated	nonsense-mediated mRNA decay factor SMG8
LOC100642307	-0.223570976	0.025937069	Down-regulated	tyrosine-protein kinase hopscotch
LOC100645212	-0.250596107	0.025937069	Down-regulated	intersectin-1
LOC100646413	-0.47006394	0.025937069	Down-regulated	growth arrest-specific protein 2
LOC100650784	-0.215834277	0.025937069	Down-regulated	deformed epidermal autoregulatory factor 1
LOC100645026	0.954157566	0.026052969	Up-regulated	protein zerknuellt 1
LOC100646360	-0.254393891	0.026052969	Down-regulated	ATP synthase subunit beta
LOC100642913	-0.243149076	0.026063777	Down-regulated	myophilin
LOC100644521	0.303453981	0.026078163	Up-regulated	LON peptidase N-terminal domain and RING finger protein 3
LOC100650833	0.38310205	0.026078163	Up-regulated	translocon-associated protein subunit beta
LOC100651822	-0.143743622	0.026177965	Down-regulated	probable protein S-acyltransferase 23
LOC100644233	0.927290453	0.026196941	Up-regulated	citrate synthase
LOC100644745	-0.180925631	0.026212223	Down-regulated	polycomb group RING finger protein 3
LOC100651802	-0.164240474	0.026212223	Down-regulated	uncharacterized LOC100651802
LOC100651196	-0.300941224	0.026219196	Down-regulated	voltage-dependent calcium channel subunit alpha-2/delta-3
LOC100650786	0.370162547	0.026288396	Up-regulated	RING finger protein unkempt
LOC100652249	-0.315486869	0.026288396	Down-regulated	protein disulfide-isomerase A6 homolog
LOC100646831	0.235420179	0.026301958	Up-regulated	GPN-loop GTPase 1
LOC100648166	0.437204637	0.026301958	Up-regulated	trimeric intracellular cation channel type 1B.1
LOC105666092	0.239886083	0.026524508	Up-regulated	transmembrane protein adipocyte-associated 1 homolog
LOC100649471	0.150151236	0.026531722	Up-regulated	B-cell receptor-associated protein 31
LOC100645987	-0.196794441	0.026553714	Down-regulated	transmembrane reductase CYB561D2

LOC100650125	0.522479355	0.026561704	Up-regulated	uncharacterized LOC100650125
LOC100651187	0.994634802	0.026597524	Up-regulated	uncharacterized LOC100651187
LOC100648438	-0.435845161	0.026696373	Down-regulated	potassium voltage-gated channel protein Shaker
LOC100644556	0.980688553	0.0267499	Up-regulated	carbohydrate sulfotransferase 11
LOC100648163	0.321240039	0.026830958	Up-regulated	E3 ubiquitin-protein ligase TRAIP
LOC100647308	-0.456571422	0.026908516	Down-regulated	armadillo repeat-containing protein 3
LOC110119505	0.647553156	0.026908516	Up-regulated	uncharacterized LOC110119505
LOC100648285	-0.271843266	0.026932565	Down-regulated	lissencephaly-1 homolog
LOC105666359	0.42153326	0.027037677	Up-regulated	CB1 cannabinoid receptor-interacting protein 1
LOC100651456	0.191172889	0.027065013	Up-regulated	ubiquitin-conjugating enzyme E2 L3
LOC100650035	1.325636295	0.027075404	Up-regulated	insulin-like growth factor-binding protein complex acid labile subunit
LOC105666060	0.590387879	0.027083304	Up-regulated	uncharacterized LOC105666060
LOC100644903	-0.228707292	0.027130367	Down-regulated	sodium-coupled monocarboxylate transporter 1
LOC100650278	-0.218149595	0.027267843	Down-regulated	pre-mRNA-splicing factor 38
LOC100650934	0.241637094	0.027275445	Up-regulated	tRNA pseudouridine synthase Pus10
LOC100651689	0.142854674	0.027275445	Up-regulated	transport and Golgi organization protein 11
LOC100648380	-0.238580264	0.027335997	Down-regulated	protein BTG2
LOC100650479	0.235863475	0.027335997	Up-regulated	glutamyl aminopeptidase
LOC100646843	0.400691547	0.027359152	Up-regulated	EP300-interacting inhibitor of differentiation 3
LOC105666652	1.258043474	0.027359953	Up-regulated	uncharacterized LOC105666652
LOC100648981	-0.357688629	0.027369218	Down-regulated	uncharacterized LOC100648981
LOC100644152	0.572783097	0.027455945	Up-regulated	solute carrier family 35 member E3
LOC100644162	0.370383397	0.027472825	Up-regulated	sodium/calcium exchanger regulatory protein 1
LOC100650758	0.135631301	0.027655782	Up-regulated	succinate dehydrogenase [ubiquinone] iron-sulfur subunit
LOC105666340	0.35343158	0.027685222	Up-regulated	uncharacterized LOC105666340
LOC100647040	0.189358942	0.027721099	Up-regulated	abhydrolase domain-containing protein 2
LOC100646969	-0.236502975	0.027744309	Down-regulated	dual specificity protein phosphatase Mpk3
LOC100644807	0.209782036	0.027874324	Up-regulated	probable 39S ribosomal protein L45
LOC100648360	0.185934197	0.027874324	Up-regulated	sorting nexin-29
LOC100651809	-0.208446928	0.027900387	Down-regulated	inactive hydroxysteroid dehydrogenase-like protein 1
LOC100646905	0.128828731	0.02794927	Up-regulated	3'-5' RNA helicase YTHDC2
LOC105665983	-0.268261051	0.027974812	Down-regulated	coatomer subunit beta
LOC100650451	-0.187078265	0.027987583	Down-regulated	VWFA and cache domain-containing protein 1
LOC100642409	0.310838208	0.028009671	Up-regulated	programmed cell death protein 2
LOC100650339	0.353880888	0.028009671	Up-regulated	chloride channel protein 2
LOC100650350	-0.26511156	0.028009671	Down-regulated	cysteine-rich with EGF-like domain protein 2
LOC100651046	0.846427557	0.028009671	Up-regulated	uncharacterized LOC100651046
LOC100642681	0.316224436	0.028038081	Up-regulated	tRNA N(3)-methylcytidine methyltransferase METTL2
LOC100648327	0.398693568	0.028038081	Up-regulated	5-hydroxytryptamine receptor 2A
LOC100646941	-0.603352165	0.028076491	Down-regulated	uncharacterized LOC100646941
LOC100649524	0.317090283	0.028081064	Up-regulated	40S ribosomal protein S6
LOC100648195	0.218245277	0.028131327	Up-regulated	cytosolic carboxypeptidase 1
LOC100649736	-1.237970512	0.028131327	Down-regulated	cyclin-dependent kinase inhibitor 1C-like
LOC110120345	-0.610599435	0.028131327	Down-regulated	origin recognition complex subunit 5
LOC100650606	-0.82987754	0.028146142	Down-regulated	cell wall protein RBR3
LOC100649783	0.200048254	0.028317153	Up-regulated	solute carrier family 35 member F6
LOC100649732	-0.22882693	0.028337033	Down-regulated	WD repeat-containing protein 13
LOC100643231	-0.378937113	0.028413866	Down-regulated	probable nuclear hormone receptor HR3
LOC100648167	0.117015732	0.028423464	Up-regulated	E3 ubiquitin-protein transferase MAEA
LOC100652290	0.230555961	0.02844176	Up-regulated	TWiK family of potassium channels protein 7
LOC100647326	0.184708498	0.028467704	Up-regulated	coatomer subunit alpha-like
LOC100645911	-0.169583003	0.028475104	Down-regulated	E3 ubiquitin-protein ligase NRDP1
LOC100651859	0.289454496	0.028475104	Up-regulated	transmembrane protein 41B
LOC105666642	-0.137205115	0.028477736	Down-regulated	rho GDP-dissociation inhibitor 2
LOC105666632	-0.341726646	0.028505681	Down-regulated	proteasome assembly chaperone 4
LOC100645845	-0.164840636	0.028532172	Down-regulated	mediator of RNA polymerase II transcription subunit 19
LOC100645511	-0.359436583	0.028708781	Down-regulated	inositol-3-phosphate synthase 1-B
LOC100647041	-0.83304659	0.028708781	Down-regulated	probable cytochrome P450 6a14 LOC100647041
LOC100648168	-0.795245303	0.028708781	Down-regulated	uncharacterized LOC100648168
LOC100649039	0.301419831	0.028708781	Up-regulated	uncharacterized LOC100649039
LOC100643265	-0.610651497	0.028866451	Down-regulated	protein turtle
LOC100643436	0.228473955	0.02892763	Up-regulated	cofilin/actin-depolymerizing factor homolog
LOC100648644	-0.202510204	0.02892763	Down-regulated	uncharacterized LOC100648644
LOC100649587	-0.245442689	0.02892763	Down-regulated	interference hedgehog
LOC100651322	-0.238910236	0.028939157	Down-regulated	scm-like with four MBT domains protein 1
LOC100650521	0.537457995	0.028981902	Up-regulated	maltase A3
LOC100646481	-0.157764936	0.028983775	Down-regulated	homeobox protein PKNOX2
LOC105666381	0.175376034	0.028983775	Up-regulated	protein FAM210A
LOC110120252	0.259007484	0.028999523	Up-regulated	uncharacterized LOC110120252
LOC100649948	-0.437113793	0.029091843	Down-regulated	uncharacterized LOC100649948
LOC105665618	0.211386673	0.02918599	Up-regulated	anaphase-promoting complex subunit 2
LOC100650318	0.434468078	0.029224609	Up-regulated	alanine--glyoxylate aminotransferase 2-like
LOC100648511	-0.18436879	0.029231844	Down-regulated	male-enhanced antigen 1
LOC100651120	-0.414918997	0.029236029	Down-regulated	4-coumarate--CoA ligase 1
LOC100650015	-0.941977428	0.029285698	Down-regulated	uncharacterized LOC100650015
LOC100651638	-0.235486458	0.029285698	Down-regulated	putative uncharacterized protein DDB_G0289263
LOC100650561	1.014736534	0.029306887	Up-regulated	alanine--glyoxylate aminotransferase
LOC100642736	0.241955669	0.029393194	Up-regulated	ski oncogene
LOC100647121	-0.757022996	0.029470106	Down-regulated	PCNA-associated factor
LOC100649515	-0.471093654	0.029560927	Down-regulated	neuronal acetylcholine receptor subunit alpha-10
LOC100643078	-0.385200683	0.029593215	Down-regulated	heart- and neural crest derivatives-expressed protein 1

LOC100650816	0.214913103	0.029593215	Up-regulated	integrator complex subunit 6
LOC100651966	-0.247607737	0.029601635	Down-regulated	popeye domain-containing protein 3
LOC105667133	0.557558359	0.029635348	Up-regulated	uncharacterized LOC105667133
LOC100649828	0.448765552	0.029636228	Up-regulated	cilia- and flagella-associated protein 65
LOC100649962	-0.266275495	0.029686951	Down-regulated	ribosomal protein 63
LOC100650655	-0.16344913	0.029686951	Down-regulated	mitochondrial chaperone BCS1
LOC100650937	-0.320851908	0.029715985	Down-regulated	putative ammonium transporter 3
LOC100648971	-0.129035524	0.029731039	Down-regulated	phosphatidylinositol-3-phosphatase SAC1
LOC100642368	1.137868192	0.029749225	Up-regulated	thyrotropin-releasing hormone receptor
LOC100647475	0.234327582	0.029756393	Up-regulated	CTD nuclear envelope phosphatase 1 homolog
LOC100651321	0.306482915	0.029763489	Up-regulated	mitochondrial import inner membrane translocase subunit Tim21
LOC100644553	0.36966042	0.02988981	Up-regulated	high affinity copper uptake protein 1
LOC100644798	-0.307657669	0.029988439	Down-regulated	LIM homeobox transcription factor 1-beta.1
LOC100646152	0.362098904	0.03012269	Up-regulated	26S proteasome non-ATPase regulatory subunit 1
LOC100647407	0.265296935	0.03012269	Up-regulated	L-galactose dehydrogenase
LOC105665717	-0.330995656	0.03012269	Down-regulated	muscle M-line assembly protein unc-89-like
LOC100650140	0.220879538	0.030180148	Up-regulated	26S proteasome regulatory subunit 10B
LOC100645563	-0.267873746	0.030335617	Down-regulated	octopamine receptor beta-1R
LOC100650370	-0.330920691	0.030335617	Down-regulated	uncharacterized LOC100650370
LOC100652150	0.670839567	0.030336418	Up-regulated	BMP-binding endothelial regulator protein
LOC100644329	0.207764953	0.030359348	Up-regulated	poly [ADP-ribose] polymerase
LOC100650275	0.211718609	0.030359348	Up-regulated	tRNA modification GTPase GTPBP3
LOC100651258	0.187547623	0.030415633	Up-regulated	charged multivesicular body protein 2a
LOC100642698	-0.460014074	0.030443494	Down-regulated	chymotrypsinogen B
LOC100645152	0.277229776	0.030454702	Up-regulated	nucleolar protein 14 homolog
LOC100645129	0.313435986	0.030498592	Up-regulated	60S ribosomal protein L35a
LOC100647851	0.227951778	0.030498592	Up-regulated	ribosomal protein S6 kinase alpha-5
LOC100644224	-0.503695798	0.030511861	Down-regulated	putative 3-methyladenine DNA glycosylase
LOC100643127	0.378243511	0.030524152	Up-regulated	ras-related protein Rab-10
LOC105666894	2.321846277	0.030524152	Up-regulated	uclacyanin-3
LOC100642586	-0.308848655	0.030699628	Down-regulated	dopamine receptor 1
LOC100642528	0.293099315	0.030745452	Up-regulated	developmentally-regulated GTP-binding protein 2
LOC100642725	0.586038505	0.030785347	Up-regulated	endoribonuclease CG2145
LOC105665656	-0.324714733	0.030800769	Down-regulated	ADP-ribosylation factor-like protein 6
LOC100644993	-0.14502149	0.030858699	Down-regulated	membrane-associated guanylate kinase
LOC100646164	-0.664963651	0.030983406	Down-regulated	pyrokinin-1 receptor
LOC100647066	0.619728505	0.031049181	Up-regulated	sterol carrier protein 2
LOC100649066	-0.652652566	0.031049181	Down-regulated	uncharacterized LOC100649066
LOC100649262	0.337267107	0.031049181	Up-regulated	18S rRNA aminocarboxypropyltransferase
LOC100646869	-0.207341926	0.031080032	Down-regulated	U1 small nuclear ribonucleoprotein C
LOC100642849	-0.317761837	0.031087024	Down-regulated	Werner Syndrome-like exonuclease
LOC100645172	-0.156775769	0.031116526	Down-regulated	probable phosphoglycerate kinase
LOC100644494	-0.540653455	0.031136711	Down-regulated	ankyrin repeat domain-containing protein 6
LOC100643132	0.179758473	0.031155131	Up-regulated	transmembrane 9 superfamily member 3
LOC100646203	-0.165886722	0.031155131	Down-regulated	succinyl-CoA:3-ketoacid coenzyme A transferase 1
LOC100649185	0.147753957	0.031298116	Up-regulated	pre-mRNA-processing factor 40 homolog A
LOC100644236	0.244343929	0.031306778	Up-regulated	NADH dehydrogenase [ubiquinone] 1 alpha subcomplex subunit 12
LOC105666737	0.383090687	0.031444017	Up-regulated	G patch domain-containing protein 11
LOC100644723	0.579258513	0.031452186	Up-regulated	ATP-binding cassette sub-family G member 1
LOC100646320	-0.926037173	0.031452186	Down-regulated	uncharacterized LOC100646320
LOC100650911	-0.290817861	0.031457306	Down-regulated	uncharacterized LOC100650911
LOC100645424	1.442014265	0.031480272	Up-regulated	zinc finger and SCAN domain-containing protein 31
LOC100645616	1.536959931	0.031590559	Up-regulated	histone H2B
LOC100646157	0.278461323	0.031616883	Up-regulated	palmitoyltransferase ZDHHC16A
LOC100645015	-0.23575986	0.031792817	Down-regulated	snRNA-activating protein complex subunit 3
LOC100646075	0.317146797	0.031815388	Up-regulated	60S ribosomal protein L34
LOC105666983	0.646576961	0.031863405	Up-regulated	GDP-D-glucose phosphorylase 1
LOC100645253	-0.15298751	0.031883844	Down-regulated	uncharacterized LOC100645253
LOC100645973	0.245213361	0.031883844	Up-regulated	F-box only protein 28
LOC100648885	0.181438301	0.031883844	Up-regulated	THO complex subunit 2
LOC100643700	-0.226507955	0.031899621	Down-regulated	cleavage and polyadenylation specificity factor subunit 4
LOC100651118	-0.275454202	0.032011499	Down-regulated	synapse-associated protein 1
LOC100652217	-0.314017192	0.032038589	Down-regulated	basement membrane-specific heparan sulfate proteoglycan core protein
LOC105666912	-0.353751294	0.032064654	Down-regulated	methyltransferase-like 26
LOC100650852	-0.207778152	0.032086304	Down-regulated	inositol polyphosphate 1-phosphatase
LOC100647140	-0.208228704	0.032095555	Down-regulated	uncharacterized LOC100647140
LOC100644450	0.387664975	0.032117149	Up-regulated	serine protease inhibitor 88Ea
LOC105665913	-0.212366104	0.032162945	Down-regulated	abasic site processing protein HMCES
LOC100643364	-0.164091825	0.03232698	Down-regulated	adenylate cyclase type 2
LOC100646021	-0.168398205	0.03232698	Down-regulated	periodic tryptophan protein 2 homolog
LOC100649607	0.143015718	0.032336022	Up-regulated	polymerase delta-interacting protein 3
LOC100646025	-0.403264008	0.032349731	Down-regulated	uncharacterized LOC100646025
LOC100643757	-0.347844164	0.032421075	Down-regulated	NADPH:adrenodoxin oxidoreductase
LOC100648873	-0.140448329	0.032472352	Down-regulated	rab GTPase-binding effector protein 1
LOC100643956	-0.147230202	0.032534029	Down-regulated	tetraspanin-13
LOC100649460	-0.336558648	0.032668298	Down-regulated	ubiquitin-like-conjugating enzyme ATG10
LOC100642910	-0.153074978	0.032725043	Down-regulated	BTB/POZ domain-containing protein 9
LOC100646040	0.193948127	0.032748691	Up-regulated	transmembrane protein 161B
LOC100650680	-0.290615292	0.032754643	Down-regulated	5-oxoprolinase
LOC100645603	0.142980334	0.032887071	Up-regulated	soluble calcium-activated nucleotidase 1
LOC100648730	0.266704905	0.032887071	Up-regulated	CDP-diacylglycerol--inositol 3-phosphatidyltransferase

LOC100649941	0.212681813	0.032887071	Up-regulated	ATP synthase mitochondrial F1 complex assembly factor 2
LOC100643722	0.279062191	0.032991896	Up-regulated	protein eva-1
LOC100648605	0.285743254	0.033074178	Up-regulated	putative uncharacterized protein DDB_G0284213
LOC110119461	0.887677886	0.033194724	Up-regulated	solute carrier family 2
LOC100646807	0.34475023	0.033222507	Up-regulated	nose resistant to fluoxetine protein 6
LOC100650087	-0.149959588	0.033345367	Down-regulated	UDP-glucose:glycoprotein glucosyltransferase
LOC100642309	0.160599524	0.033400986	Up-regulated	transcription factor BTF3 homolog 4
LOC100642293	-0.427769069	0.033441294	Down-regulated	DNA N6-methyl adenine demethylase
LOC100648918	0.147440241	0.033445359	Up-regulated	mucin-3A
LOC105665841	0.467610495	0.033465986	Up-regulated	muscle-specific protein 300 kDa
LOC110119695	-0.259118234	0.033564676	Down-regulated	uncharacterized LOC110119695
LOC105667080	0.28544944	0.033628821	Up-regulated	uncharacterized LOC105667080
LOC100649466	-0.198840376	0.033694186	Down-regulated	ATPase family AAA domain-containing protein 3A homolog
LOC100649653	0.442807623	0.033694186	Up-regulated	valacyclovir hydrolase
LOC100646484	-0.302946426	0.03369906	Down-regulated	atrial natriuretic peptide receptor 1
LOC100643553	0.132039699	0.033967842	Up-regulated	ethanolaminephosphotransferase 1
LOC100647242	-0.171247221	0.033967842	Down-regulated	2-phosphoxylose phosphatase 1
LOC100645289	-0.894185009	0.033996011	Down-regulated	uncharacterized LOC100645289
LOC100642829	0.334825372	0.034019797	Up-regulated	peroxisome assembly protein 12
LOC100646122	0.370681884	0.034111495	Up-regulated	P protein
LOC100645516	-0.343866696	0.034139157	Down-regulated	CD151 antigen
LOC100643110	0.33559502	0.034167599	Up-regulated	sodium channel and clathrin linker 1
LOC100643659	-0.232502839	0.034282442	Down-regulated	F-box/LRR-repeat protein fbxl-1
LOC100644080	-0.266694195	0.034282442	Down-regulated	histone-lysine N-methyltransferase Suv4-20
LOC100650084	-0.400350592	0.034282442	Down-regulated	uncharacterized LOC100650084
LOC100651835	0.235151276	0.034282442	Up-regulated	tubulin polyglutamylase TTLL5
LOC110119919	-0.474357243	0.034282442	Down-regulated	uncharacterized LOC110119919
LOC100643135	-0.139631263	0.034381262	Down-regulated	protein l(2)37Cc
LOC100650613	-0.168257822	0.034405573	Down-regulated	protein real-time
LOC100649822	0.27036705	0.034443198	Up-regulated	protein eyes shut
LOC100650858	-0.384751516	0.034541226	Down-regulated	innexin inx3
LOC100645446	1.356715105	0.034570148	Up-regulated	dnaJ homolog subfamily C member 22
LOC100648169	0.386255502	0.03459864	Up-regulated	putative aldehyde dehydrogenase family 7 member A1 homolog
LOC100651829	0.281886186	0.03459864	Up-regulated	proteasome subunit alpha type-6
LOC100645947	-0.215343758	0.034599346	Down-regulated	regulator of microtubule dynamics protein 1
LOC100646145	1.193111081	0.034624812	Up-regulated	ejaculatory bulb-specific protein 3
LOC100650846	-0.160716326	0.034639586	Down-regulated	zinc finger Ran-binding domain-containing protein 2
LOC110119243	-0.367336879	0.034658481	Down-regulated	uncharacterized LOC110119243
LOC100631092	0.158014087	0.034687528	Up-regulated	HEM-protein
LOC100648485	0.16176962	0.034690539	Up-regulated	lamin Dm0
LOC100643425	-0.337026858	0.034773947	Down-regulated	uncharacterized LOC100643425
LOC100648832	-0.295074955	0.034773947	Down-regulated	uncharacterized LOC100648832
LOC100645720	-0.167344152	0.034812692	Down-regulated	phosphatidylinositol 4-kinase alpha
LOC100651569	-0.337437812	0.034812692	Down-regulated	COMM domain-containing protein 4
LOC105666129	0.492512992	0.034837033	Up-regulated	uncharacterized LOC105666129
LOC100642246	0.20870291	0.034868629	Up-regulated	ataxin-7-like protein 3
LOC100648384	0.641679853	0.034877255	Up-regulated	octopamine receptor beta-3R
LOC100651533	0.330309825	0.034967881	Up-regulated	EH domain-binding protein 1
LOC100651940	0.278577886	0.03499441	Up-regulated	uncharacterized LOC100651940
LOC100650575	0.189706483	0.035205945	Up-regulated	isochorismatase domain-containing protein 2
LOC100650182	0.305971081	0.035209145	Up-regulated	protein croquemort
LOC100647115	-0.311159029	0.03525035	Down-regulated	mRNA-decapping enzyme 1A
LOC105665655	0.279444736	0.035280051	Up-regulated	NAD(P)H-hydrate epimerase
LOC100642466	-0.417957716	0.035315855	Down-regulated	transmembrane protein 17
LOC100644834	-0.386009872	0.035331054	Down-regulated	putative tyramine receptor 2
LOC100649015	0.135356425	0.035331054	Up-regulated	N-alpha-acetyltransferase 35
LOC100650605	0.28576602	0.0353793	Up-regulated	DNA repair protein RAD50
LOC100652202	-0.476990176	0.0353793	Down-regulated	UNC93-like protein
LOC100649034	-0.171514578	0.035503093	Down-regulated	synaptic functional regulator FMR1
LOC100643857	-1.014009824	0.035554966	Down-regulated	caspase-1
LOC100650045	0.65428482	0.035606778	Up-regulated	cuticlin-4
LOC100644470	0.90559174	0.035642318	Up-regulated	inositol 2-dehydrogenase
LOC105666404	0.67870756	0.035683739	Up-regulated	troponin C
LOC100646381	0.147614835	0.035752941	Up-regulated	G patch domain-containing protein 1 homolog
LOC100644906	-0.200415133	0.035773141	Down-regulated	histone H3.3A
LOC100645465	0.342469981	0.035773141	Up-regulated	60S ribosomal protein L22
LOC100648591	0.19482065	0.035781813	Up-regulated	helicase domino
LOC100642384	-0.253791354	0.035796108	Down-regulated	N-alpha-acetyltransferase 60
LOC100644660	-0.417476804	0.035836548	Down-regulated	octopamine receptor Oamb
LOC100648137	0.25489336	0.035836548	Up-regulated	alpha-1
LOC100645537	-0.22676735	0.035858594	Down-regulated	potassium voltage-gated channel protein Shaw
LOC100650625	-0.084477729	0.035998283	Down-regulated	serine/threonine-protein phosphatase 2A 65 kDa regulatory subunit A
LOC100647178	-1.259617513	0.036091141	Down-regulated	venom acid phosphatase Acph-1
LOC100642724	0.162691103	0.036099055	Up-regulated	probable isocitrate dehydrogenase [NAD] subunit alpha
LOC100643028	0.292394546	0.036099055	Up-regulated	60S ribosomal protein L21
LOC100649621	0.831583828	0.036099055	Up-regulated	insulin-like growth factor-binding protein complex acid labile subunit
LOC100643540	0.296865503	0.036380846	Up-regulated	MATH and LRR domain-containing protein PFE0570w
LOC100649031	-0.324338809	0.036389774	Down-regulated	facilitated trehalose transporter Tret1
LOC100649344	0.174109819	0.036405324	Up-regulated	katanin p80 WD40 repeat-containing subunit B1
LOC100647093	-0.235997411	0.036449218	Down-regulated	probable nuclear transport factor 2
LOC100652035	0.286389136	0.03647571	Up-regulated	AP-1 complex subunit sigma-2

LOC105667138	-0.18594093	0.036478445	Down-regulated	acyl carrier protein
LOC100648108	-0.152779806	0.036535162	Down-regulated	SUMO-conjugating enzyme UBC9-B
LOC100646688	-0.577234083	0.036558374	Down-regulated	protein ATP6V1FNB
LOC100644385	0.256018698	0.036567749	Up-regulated	gem-associated protein 8
LOC100643776	-0.102800554	0.036638953	Down-regulated	exocyst complex component 2
LOC100642398	-0.193322119	0.036640894	Down-regulated	dynein regulatory complex protein 1
LOC100649270	-0.390943103	0.03671516	Down-regulated	MD-2-related lipid-recognition protein
LOC100651274	0.213427816	0.03671516	Up-regulated	bromodomain-containing protein 4
LOC100644927	0.372669845	0.036737121	Up-regulated	ubiquitin carboxyl-terminal hydrolase 47
LOC105666297	-0.839517023	0.036770315	Down-regulated	uncharacterized LOC105666297
LOC100648061	0.2983491	0.036777689	Up-regulated	crossover junction endonuclease MUS81
LOC100650824	0.252169128	0.036910515	Up-regulated	SRR1-like protein
LOC100650542	-0.284276088	0.036964983	Down-regulated	metaxin-1
LOC100642878	0.407154493	0.036978561	Up-regulated	atlastin
LOC100647388	0.112578332	0.03700585	Up-regulated	E3 ubiquitin-protein ligase RNF13
LOC100649264	-0.323813476	0.03700585	Down-regulated	neuroligin-4
LOC100651561	-0.138670619	0.03700585	Down-regulated	NEDD8
LOC100643000	0.628738556	0.037028798	Up-regulated	potassium channel subfamily K member 16
LOC100643041	0.340292646	0.037028798	Up-regulated	uncharacterized LOC100643041
LOC100651415	0.189178658	0.037028798	Up-regulated	erlin-1
LOC105665719	0.293467156	0.037028798	Up-regulated	uncharacterized LOC105665719
LOC100643411	-0.565815825	0.037049979	Down-regulated	D-beta-hydroxybutyrate dehydrogenase
LOC100650026	-0.175246773	0.037182192	Down-regulated	phosphatidylinositol 4-phosphate 5-kinase type-1 alpha
LOC100651579	0.322851502	0.03721498	Up-regulated	COUP transcription factor 1-like
LOC100650787	-0.420789793	0.037249363	Down-regulated	tachykinin-like peptides receptor 99D
LOC100643898	0.268487412	0.037279338	Up-regulated	deoxyribodipyrimidine photo-lyase
LOC100646037	0.30400409	0.037283256	Up-regulated	F-box/LRR-repeat protein 2
LOC100648660	0.23164623	0.037311847	Up-regulated	ubiquitin recognition factor in ER-associated degradation protein 1
LOC100650484	0.877346829	0.037316992	Up-regulated	putative uncharacterized protein DDB_G0271606
LOC100642714	-0.132924555	0.037320092	Down-regulated	formin-binding protein 4
LOC100647245	0.297148188	0.037320092	Up-regulated	mucolipin-3
LOC100647565	0.299682789	0.037320092	Up-regulated	eukaryotic translation initiation factor 3 subunit K
LOC100648405	-0.381403936	0.037355232	Down-regulated	mitochondrial import inner membrane translocase subunit Tim9
LOC100650383	-0.624885154	0.03741836	Down-regulated	uncharacterized LOC100650383
LOC100646356	0.251097019	0.03751148	Up-regulated	uncharacterized LOC100646356
LOC100646667	0.967317481	0.03751148	Up-regulated	circadian clock-controlled protein daywake
LOC100646769	-0.268600627	0.037569335	Down-regulated	uncharacterized LOC100646769
LOC100648317	-0.315027406	0.037668616	Down-regulated	deoxynucleoside kinase
LOC100650159	0.831522087	0.037677152	Up-regulated	uncharacterized LOC100650159
LOC100650891	0.463740862	0.03769618	Up-regulated	uncharacterized LOC100650891
LOC100642436	-0.311645627	0.037749801	Down-regulated	glutamate receptor ionotropic
LOC100651962	-0.104680291	0.037749801	Down-regulated	ubiquitin carboxyl-terminal hydrolase calypso
LOC100652188	-0.176361684	0.037856989	Down-regulated	copper chaperone for superoxide dismutase
LOC100647335	-0.326985128	0.037949155	Down-regulated	guanylate cyclase soluble subunit beta-1
LOC100647535	0.261515759	0.037996089	Up-regulated	ribosome production factor 2 homolog
LOC100642854	-0.139783142	0.038031181	Down-regulated	uncharacterized LOC100642854
LOC100647192	-0.124659575	0.038031181	Down-regulated	E3 ubiquitin-protein ligase RBBP6
LOC100647989	-0.23466342	0.038031181	Down-regulated	ubiquitin-like protein 7
LOC100645558	0.235749408	0.038068804	Up-regulated	protein UXT homolog
LOC100647958	-0.357385793	0.038149207	Down-regulated	arylsulfatase J
LOC100648636	-0.35420415	0.038196247	Down-regulated	2-amino-3-ketobutyrate coenzyme A ligase
LOC105667146	1.645513117	0.038253837	Up-regulated	uncharacterized LOC105667146
LOC100647184	-0.216367086	0.038286054	Down-regulated	6-phosphogluconolactonase
LOC100643029	-0.300009998	0.038325321	Down-regulated	multifunctional methyltransferase subunit TRM112-like protein
LOC100649338	0.290772864	0.038404813	Up-regulated	proteoglycan 4
LOC100651880	0.434236886	0.038422953	Up-regulated	probable kinetochore protein nuf2
LOC100647113	-0.228394748	0.038440961	Down-regulated	splicing factor 1
LOC105666410	-0.321189054	0.038440961	Down-regulated	translation machinery-associated protein 7 homolog
LOC100645678	0.77747465	0.038571916	Up-regulated	laminin subunit alpha
LOC100644566	0.208506011	0.038578989	Up-regulated	translationally-controlled tumor protein homolog
LOC100647455	-0.175048765	0.038748991	Down-regulated	SR-related and CTD-associated factor 4
LOC100645390	0.145562478	0.038810628	Up-regulated	brahma-associated protein of 60 kDa
LOC100648276	0.305924525	0.038811113	Up-regulated	40S ribosomal protein S11
LOC100647912	0.351941016	0.038931027	Up-regulated	exonuclease 3'-5' domain-containing protein 2
LOC100643173	-0.362453529	0.039003253	Down-regulated	bone morphogenetic protein 1
LOC100646482	-0.3233814	0.039003253	Down-regulated	potassium voltage-gated channel subfamily H member 8
LOC100645626	0.192258687	0.039203408	Up-regulated	eukaryotic translation initiation factor 2-alpha kinase 1
LOC100651595	0.698949953	0.0392347	Up-regulated	uncharacterized LOC100651595
LOC100647498	-0.307609306	0.039310712	Down-regulated	maltase A2
LOC100642986	-0.135098582	0.039354777	Down-regulated	F-box-like/WD repeat-containing protein TBL1XR1
LOC100646095	1.191340861	0.039410539	Up-regulated	uncharacterized protein PF3D7_1120600
LOC100645028	0.261907678	0.039437642	Up-regulated	60S ribosomal protein L19
LOC100645259	-0.434271119	0.039437642	Down-regulated	pancreatic triacylglycerol lipase
LOC100649079	-0.397272804	0.039437642	Down-regulated	ELAV-like protein 2
LOC100645466	-0.161332058	0.039445869	Down-regulated	regulation of nuclear pre-mRNA domain-containing protein 1B
LOC100645419	0.227734125	0.039466771	Up-regulated	triosephosphate isomerase
LOC100645858	1.639557488	0.039472493	Up-regulated	histone H3
LOC100643901	-0.136777332	0.039658973	Down-regulated	cleft lip and palate transmembrane protein 1-like protein
LOC105666599	-0.76159739	0.039691927	Down-regulated	signal transducer and activator of transcription C-like
LOC100645926	0.204585526	0.039758395	Up-regulated	serine protease snake
LOC100647420	-0.117520932	0.039824107	Down-regulated	armadillo repeat-containing protein 8

LOC100645667	-0.183416305	0.039847782	Down-regulated	haloacid dehalogenase-like hydrolase domain-containing 5
LOC100644557	0.544618608	0.039853498	Up-regulated	sushi
LOC100645607	-0.162471582	0.039880652	Down-regulated	WD repeat-containing protein 37
LOC100645918	-0.211241059	0.039880652	Down-regulated	HEAT repeat-containing protein 1
LOC100643183	0.317103286	0.040046594	Up-regulated	Bardet-Biedl syndrome 7 protein homolog
LOC100643533	-0.279367393	0.040071906	Down-regulated	fasciclin-1
LOC100645487	0.615014295	0.04025605	Up-regulated	uncharacterized LOC100645487
LOC100647823	-0.319570914	0.04025605	Down-regulated	poly(rC)-binding protein 3
LOC100642830	-0.622944266	0.040284027	Down-regulated	putative thiamine transporter SLC35F3
LOC100650714	-0.439272141	0.040295213	Down-regulated	RNA-binding protein Musashi homolog Rbp6
LOC100647511	0.265495514	0.040296147	Up-regulated	C2 domain-containing protein 5
LOC100648115	0.41210948	0.04040239	Up-regulated	dynein regulatory complex subunit 4
LOC100646621	0.206172148	0.040408016	Up-regulated	28S ribosomal protein S18a
LOC100651854	0.239271771	0.040424724	Up-regulated	sphingosine-1-phosphate lyase
LOC100643754	-0.513642418	0.040430148	Down-regulated	PDZ domain-containing RING finger protein 4
LOC100642439	-0.214435687	0.040608659	Down-regulated	enhancer of mRNA-decapping protein 3
LOC100644622	-0.172812322	0.040681533	Down-regulated	integrator complex subunit 5
LOC100652020	0.274246092	0.040681533	Up-regulated	60S ribosomal protein L23a
LOC100650677	0.113621648	0.040689799	Up-regulated	gametogenetin-binding protein 2-like
LOC100644076	2.552475011	0.040750363	Up-regulated	ubiquitin-40S ribosomal protein S27a
LOC100647728	0.223753734	0.040761607	Up-regulated	beclin-1-like protein
LOC100644897	-0.175097914	0.040786987	Down-regulated	protein O-mannosyl-transferase TMTC4
LOC100644907	-0.3135336	0.040786987	Down-regulated	uncharacterized LOC100644907
LOC100648799	0.525614774	0.040786987	Up-regulated	facilitated trehalose transporter Tret1
LOC100643921	-0.456874775	0.040789466	Down-regulated	peroxidasin
LOC100642321	0.846349216	0.040910591	Up-regulated	D-galactonate transporter
LOC100642315	0.202334793	0.04094697	Up-regulated	nucleolar protein 56
LOC100644360	1.597118737	0.04094697	Up-regulated	nose resistant to fluoxetine protein 6
LOC100648402	-0.556763688	0.04094697	Down-regulated	phospholipid-transporting ATPase IF
LOC100644365	0.574270053	0.040952431	Up-regulated	uncharacterized LOC100644365
LOC100643456	-0.491703644	0.040961829	Down-regulated	cell adhesion molecule 3
LOC100650972	0.499339502	0.040961829	Up-regulated	lipoamide acyltransferase - branched-chain alpha-keto acid dehydrogenase
LOC100652173	-0.348902893	0.040961829	Down-regulated	uncharacterized LOC100652173
LOC100649166	0.529377434	0.041053294	Up-regulated	uncharacterized LOC100649166
LOC100645350	-0.18705426	0.041073107	Down-regulated	nicotinamide/nicotinic acid mononucleotide adenylyltransferase 3
LOC100645778	-0.228314661	0.041161547	Down-regulated	rho-related BTB domain-containing protein 1
LOC100642936	1.00961927	0.041172418	Up-regulated	cytochrome P450 6k1 LOC100642936
LOC100648745	0.206837689	0.041222082	Up-regulated	pre-mRNA-splicing factor ISY1 homolog
LOC100650711	0.242553414	0.041222082	Up-regulated	sterol regulatory element-binding protein cleavage-activating protein
LOC100644476	0.205261196	0.041279666	Up-regulated	transforming growth factor-beta-induced protein ig-h3
LOC100646132	-0.187507103	0.041279666	Down-regulated	UPF0047 protein YjbQ
LOC100647360	-0.418784799	0.041321529	Down-regulated	patched domain-containing protein 3
LOC110119613	0.637881042	0.041321529	Up-regulated	uncharacterized LOC110119613
LOC100649375	-0.180658956	0.04135112	Down-regulated	PRKC apoptosis WT1 regulator protein
LOC100652085	-0.31411844	0.041554202	Down-regulated	estradiol 17-beta-dehydrogenase 11
LOC100643330	0.356737194	0.041688961	Up-regulated	nipped-B-like protein
LOC100651736	-0.140504263	0.041742153	Down-regulated	docking protein 1
LOC105666056	-0.199471363	0.041905923	Down-regulated	S-formylglutathione hydrolase
LOC100650747	0.170002776	0.042008648	Up-regulated	protein CASC3
LOC100652310	-0.354215368	0.042008648	Down-regulated	protein asteroid-like
LOC100647694	-0.268486306	0.042090292	Down-regulated	methyl-CpG-binding domain protein 2
LOC100647237	0.339911963	0.042205487	Up-regulated	CDGSH iron-sulfur domain-containing protein 3
LOC100650235	-0.248823952	0.042237432	Down-regulated	BET1 homolog
LOC100647039	0.819768971	0.042493265	Up-regulated	acetyl-CoA carboxylase
LOC105665993	0.416927575	0.042534024	Up-regulated	uncharacterized LOC105665993
LOC100645643	0.126767592	0.04263095	Up-regulated	zinc finger protein 236
LOC100648132	0.154778871	0.04267562	Up-regulated	egl nine homolog 1
LOC100649041	-0.42058991	0.042704304	Down-regulated	uncharacterized LOC100649041
LOC100651739	0.388293868	0.042704304	Up-regulated	uncharacterized LOC100651739
LOC100650134	0.112638621	0.042762497	Up-regulated	ras-related protein Rac1
LOC100651344	0.380208046	0.042762497	Up-regulated	thyrotroph embryonic factor
LOC100642319	0.159430095	0.042906945	Up-regulated	nuclear pore complex protein Nup214
LOC100644932	0.302942872	0.042906945	Up-regulated	AP-1 complex subunit mu-1-like
LOC100648955	1.508479106	0.042906945	Up-regulated	probable peroxisomal acyl-coenzyme A oxidase 1
LOC100651207	-0.182781147	0.042974189	Down-regulated	leukocyte receptor cluster member 8 homolog
LOC100646005	0.280488016	0.042989969	Up-regulated	40S ribosomal protein S3a
LOC100647961	0.695902857	0.042989969	Up-regulated	uncharacterized LOC100647961
LOC110120015	0.578876245	0.043049768	Up-regulated	uncharacterized LOC110120015
LOC100642585	0.118215302	0.043201079	Up-regulated	T-complex protein 1 subunit delta
LOC100644947	-0.143980665	0.043259358	Down-regulated	protein max
LOC100644529	-0.340255605	0.043300465	Down-regulated	cAMP-dependent protein kinase type II regulatory subunit
LOC110119561	1.02524557	0.043349827	Up-regulated	uncharacterized LOC110119561
LOC100643997	-0.532584346	0.043380916	Down-regulated	prion-like-(Q/N-rich) domain-bearing protein 25
LOC100651531	0.934481184	0.043380916	Up-regulated	uncharacterized LOC100651531
LOC100642657	-0.492812786	0.043411288	Down-regulated	tubulin glycylase 3A
LOC100650008	0.209440486	0.04342947	Up-regulated	luc7-like protein 3
LOC100647211	-0.129668461	0.043458198	Down-regulated	methylosome subunit pICln
LOC110119575	0.357311913	0.043458198	Up-regulated	peroxiredoxin-2-like
LOC105665828	1.234898212	0.043485674	Up-regulated	uncharacterized LOC105665828
LOC100651623	-0.313278486	0.043503364	Down-regulated	tumor necrosis factor receptor superfamily member wengen
LOC100642785	-0.13230145	0.043535305	Down-regulated	transportin-3

LOC110119847	0.664631976	0.043535305	Up-regulated	protein lethal(2)essential for life
LOC100643378	-0.174736076	0.043616644	Down-regulated	WD repeat and FYVE domain-containing protein 3
LOC105667085	-0.672032397	0.043616644	Down-regulated	uncharacterized LOC105667085
LOC100650524	-0.403787627	0.043655902	Down-regulated	protein unc-13 homolog B
LOC100643725	-0.221687053	0.043668985	Down-regulated	dTTP/UTP pyrophosphatase
LOC100644628	0.362798776	0.043668985	Up-regulated	uncharacterized LOC100644628
LOC100649651	0.2628486	0.043679773	Up-regulated	uncharacterized LOC100649651
LOC105666182	0.349914015	0.043679773	Up-regulated	glutamyl-tRNA(Gln) amidotransferase subunit C
LOC100642393	-0.46353739	0.043750084	Down-regulated	protein tipE
LOC100650237	-0.224147722	0.043754704	Down-regulated	protein transport protein Sec31A
LOC100648759	-0.149609586	0.043758582	Down-regulated	nuclear receptor-binding protein homolog
LOC100644718	-0.330419197	0.043897895	Down-regulated	uncharacterized LOC100644718
LOC100648068	-1.007928006	0.043984557	Down-regulated	neprilysin-1-like
LOC100645857	-0.235050008	0.044035617	Down-regulated	centromere-associated protein E
LOC100649724	0.15594944	0.044035617	Up-regulated	protein transport protein Sec16A
LOC110119826	0.790028357	0.04406338	Up-regulated	uncharacterized LOC110119826
LOC100648464	-0.126195924	0.044066497	Down-regulated	ran-binding protein 3
LOC100642549	0.193063377	0.044095945	Up-regulated	RPII140-upstream gene protein
LOC110119347	-0.280968623	0.044095945	Down-regulated	uncharacterized LOC110119347
LOC100651291	0.341670647	0.044122439	Up-regulated	cytochrome P450 6a2 LOC100651291
LOC100649615	-0.250041374	0.044127331	Down-regulated	ataxin-7-like protein 1
LOC100651627	-0.246710178	0.044148125	Down-regulated	PHD finger-like domain-containing protein 5A
LOC100646650	-0.247332314	0.044160404	Down-regulated	uncharacterized LOC100646650
LOC100646708	-0.118512698	0.044161811	Down-regulated	ubiquitin-conjugating enzyme E2-22 kDa
LOC100642524	0.463226545	0.044270561	Up-regulated	uncharacterized LOC100642524
LOC100652044	-0.335516781	0.044278067	Down-regulated	probable RNA-binding protein 18
LOC100650540	-0.171043979	0.044385492	Down-regulated	protein O-mannosyl-transferase Tmtc3
LOC100647653	-1.065650846	0.044397086	Down-regulated	uncharacterized LOC100647653
LOC100646011	0.580926331	0.044549215	Up-regulated	dynein axonemal heavy chain 2
LOC100642642	0.94595345	0.044572007	Up-regulated	uncharacterized LOC100642642
LOC100646422	-0.725066268	0.044572007	Down-regulated	putative glycerol kinase 5
LOC100647453	-0.472190109	0.044790727	Down-regulated	zinc finger protein ZIC 1
LOC100644548	-0.225332529	0.044880407	Down-regulated	DNA mismatch repair protein Msh2
LOC100651127	0.155806062	0.045004593	Up-regulated	broad-complex core protein
LOC100645292	-0.194293884	0.045377928	Down-regulated	dehydrogenase/reductase SDR family member 4
LOC100642303	0.323137016	0.045381931	Up-regulated	pantothenate kinase 3
LOC100647166	-0.988069896	0.045396187	Down-regulated	histidine-rich glycoprotein-like
LOC100643434	0.137231323	0.045432028	Up-regulated	serine/threonine-protein kinase PAK mbt
LOC100645254	0.183854848	0.045432028	Up-regulated	uncharacterized LOC100645254
LOC100649839	-0.290960685	0.045432028	Down-regulated	G-protein coupled receptor dmsr-1
LOC100651391	0.193274521	0.045435715	Up-regulated	facilitated trehalose transporter Tret1-like
LOC100650995	-0.21701969	0.04543621	Down-regulated	solute carrier family 23 member 1
LOC110119307	-0.222019689	0.045734271	Down-regulated	uncharacterized LOC110119307
LOC100643702	-0.283675251	0.045844774	Down-regulated	choline transporter-like protein 1
LOC100646749	-0.174001049	0.045844774	Down-regulated	mitogen-activated protein kinase kinase kinase 10
LOC105665954	0.477934663	0.045844774	Up-regulated	selenoprotein K
LOC100648941	0.216617135	0.04586746	Up-regulated	elongator complex protein 4
LOC100651706	-0.188531822	0.046227433	Down-regulated	far upstream element-binding protein 3
LOC100642271	0.109662362	0.046291239	Up-regulated	integrator complex subunit 11
LOC100645170	-0.095419512	0.046291239	Down-regulated	protein lifeguard 1
LOC100646181	-0.250731247	0.046291239	Down-regulated	aldehyde dehydrogenase
LOC100649242	0.163320269	0.046291239	Up-regulated	lysine--tRNA ligase
LOC100648742	0.327976052	0.046392129	Up-regulated	E3 ubiquitin-protein ligase RNF8
LOC100648033	0.976411572	0.046453475	Up-regulated	uncharacterized LOC100648033
LOC100649703	0.289899294	0.046453475	Up-regulated	lutropin-choriogonadotropic hormone receptor
LOC100647945	-0.393394897	0.046766372	Down-regulated	farnesol dehydrogenase
LOC100643096	-0.109389847	0.046804958	Down-regulated	vacuolar protein sorting-associated protein 11 homolog
LOC105667206	0.487373874	0.046804958	Up-regulated	uncharacterized LOC105667206
LOC100648335	-0.319624885	0.046898544	Down-regulated	LIM/homeobox protein Lhx3
LOC100645521	0.401704245	0.047195477	Up-regulated	epidermal growth factor receptor
LOC100645522	-0.163024178	0.047195477	Down-regulated	(E3-independent) E2 ubiquitin-conjugating enzyme UBE2O
LOC105666539	0.824289583	0.047298455	Up-regulated	uncharacterized LOC105666539
LOC100646535	-0.34428023	0.047357482	Down-regulated	neurobeachin
LOC100647526	-0.153602026	0.047560534	Down-regulated	dynein light chain 2
LOC100647866	-0.262450774	0.047560534	Down-regulated	neuronal PAS domain-containing protein 4
LOC100648188	-0.145486396	0.04759683	Down-regulated	uncharacterized LOC100648188
LOC100649062	-0.11941927	0.04759683	Down-regulated	TOM1-like protein 2
LOC100649257	-0.158820783	0.04759683	Down-regulated	cationic amino acid transporter 3
LOC100647893	-0.259649344	0.04760826	Down-regulated	coiled-coil domain-containing protein 115
LOC100649622	-0.191833696	0.047692775	Down-regulated	NADH-cytochrome b5 reductase 3
LOC100643222	-0.165560507	0.047698725	Down-regulated	protein OPI10 homolog
LOC100650777	-0.194306758	0.047716733	Down-regulated	zinc finger CCCH domain-containing protein 18
LOC100643900	1.732108403	0.047741945	Up-regulated	15-hydroxyprostaglandin dehydrogenase [NAD(+)]
LOC100648808	0.208690922	0.047831626	Up-regulated	protein FAM133B
LOC100646559	0.19448117	0.047854241	Up-regulated	60S ribosomal protein L3
LOC100648211	-0.232850818	0.047874053	Down-regulated	autophagy-related protein 101
LOC100646214	0.260700129	0.047954885	Up-regulated	uncharacterized LOC100646214
LOC100649307	-0.485946628	0.047954885	Down-regulated	collagen alpha-1(IV) chain
LOC100650629	0.259903089	0.047983029	Up-regulated	dnaJ homolog subfamily C member 21
LOC100644381	-0.362863131	0.048010187	Down-regulated	basic-leucine zipper transcription factor A
LOC100645067	0.128597419	0.048052954	Up-regulated	NADH dehydrogenase [ubiquinone] 1 alpha subcomplex subunit 8

LOC110120243	-1.004333509	0.048188655	Down-regulated	inner centromere protein-like
LOC100645928	1.121871747	0.048456418	Up-regulated	uncharacterized LOC100645928
LOC100649624	-0.202457438	0.048524733	Down-regulated	rho-associated protein kinase 2
LOC100649250	-0.310579656	0.048571398	Down-regulated	protein Fe65 homolog
LOC100651126	-0.148828167	0.048660422	Down-regulated	ARF GTPase-activating protein GIT1
LOC100642421	-0.197698572	0.04866216	Down-regulated	galactoside 2-alpha-L-fucosyltransferase Sec1
LOC100650315	-0.371561197	0.04866216	Down-regulated	division abnormally delayed protein
LOC100649635	-0.327551397	0.048819077	Down-regulated	uncharacterized LOC100649635
LOC100652174	0.406434656	0.048836934	Up-regulated	hydroxymethylglutaryl-CoA lyase
LOC105667005	-0.269310324	0.04891792	Down-regulated	transmembrane inner ear expressed protein
LOC100652160	1.378409374	0.04908641	Up-regulated	modular serine protease
LOC100652047	0.966116599	0.049113532	Up-regulated	chitin deacetylase 1
LOC100643730	0.213944922	0.04911825	Up-regulated	queuosine salvage protein
LOC100649132	0.630090535	0.04931299	Up-regulated	pyruvate kinase
LOC100652046	0.248435056	0.04940236	Up-regulated	activator of basal transcription 1
LOC100649645	-0.15365182	0.049423569	Down-regulated	SH2B adapter protein 1
LOC100644106	0.198957822	0.049546057	Up-regulated	Hermansky-Pudlak syndrome 1 protein homolog
LOC100648238	0.285912398	0.049546626	Up-regulated	surfeit locus protein 6 homolog
LOC110119909	-0.754064237	0.049774257	Down-regulated	ELMO domain-containing protein C-like
LOC100651224	0.672294494	0.049809796	Up-regulated	uncharacterized LOC100651224
LOC105665770	-0.298346428	0.049829399	Down-regulated	interaptin

ACUTE ACETAMIPRID

Gene ID	log2Fold Change	Adjusted P-value (FDR)	Expression pattern	Gene annotation
LOC100648995	8.614530008	7.19E-07	Up-regulated	cytochrome P450 6k1 LOC100648995
LOC100646259	1.100640297	7.19E-07	Up-regulated	trichohyalin
LOC100643462	3.251137169	3.07E-06	Up-regulated	RING finger protein nhl-1
LOC100645959	2.044212839	3.25E-06	Up-regulated	uncharacterized LOC100645959
LOC100650582	2.802159977	4.64E-06	Up-regulated	uncharacterized LOC100650582
LOC100645530	3.135363473	9.01E-06	Up-regulated	E3 ubiquitin-protein ligase lubel
LOC100647860	2.529751517	1.27E-05	Up-regulated	uncharacterized LOC100647860
LOC105665934	2.013632718	1.32E-05	Up-regulated	uncharacterized LOC105665934
LOC100642937	2.607498979	1.60E-05	Up-regulated	actin-binding Rho-activating protein
LOC100646382	2.440818963	1.60E-05	Up-regulated	ras-related and estrogen-regulated growth inhibitor
LOC100649033	2.55821804	1.74E-05	Up-regulated	cyclin-dependent kinase-like 4
LOC100648814	1.62322985	1.74E-05	Up-regulated	uncharacterized LOC100648814
LOC100652247	2.915795875	1.89E-05	Up-regulated	cAMP-dependent protein kinase catalytic subunit PRKX
LOC100649151	2.40099643	1.89E-05	Up-regulated	zinc finger CCHC domain-containing protein 24
LOC100643103	2.062948665	1.89E-05	Up-regulated	homeotic protein proboscipedia
LOC100644880	3.017038609	1.97E-05	Up-regulated	uncharacterized LOC100644880
LOC100649328	2.450254766	1.97E-05	Up-regulated	obscurin
LOC100650291	1.487699728	1.97E-05	Up-regulated	four and a half LIM domains protein 2
LOC100648003	4.634985382	2.17E-05	Up-regulated	UDP-glycosyltransferase UGT5
LOC100642680	1.725312049	2.27E-05	Up-regulated	leucine-rich repeat-containing protein 20
LOC100645262	1.186179526	2.42E-05	Up-regulated	uncharacterized LOC100645262
LOC100648579	2.659410152	2.65E-05	Up-regulated	uncharacterized LOC100648579
LOC100643073	3.205631294	2.82E-05	Up-regulated	uncharacterized LOC100643073
LOC105666016	1.771214268	3.01E-05	Up-regulated	uncharacterized LOC105666016
LOC100649207	2.602458196	3.07E-05	Up-regulated	flagellar attachment zone protein 1
LOC100645933	2.509261931	3.07E-05	Up-regulated	homeobox protein six1
LOC105665950	2.388946598	3.07E-05	Up-regulated	titin
LOC100643406	2.336521768	3.07E-05	Up-regulated	titin homolog
LOC100651289	2.937514033	3.21E-05	Up-regulated	uncharacterized LOC100651289
LOC100650553	1.933755858	3.21E-05	Up-regulated	glutamic acid-rich protein
LOC100644118	2.293103073	5.54E-05	Up-regulated	twitchin
LOC100651210	0.320499716	6.40E-05	Up-regulated	methionine-R-sulfoxide reductase B1
LOC100643338	1.316147926	6.53E-05	Up-regulated	leucine-rich repeat extensin-like protein 5
LOC100648162	1.238402827	6.53E-05	Up-regulated	uncharacterized LOC100648162
LOC100651225	0.64079761	6.53E-05	Up-regulated	coronin-1C-A
LOC100652071	7.293828896	6.63E-05	Up-regulated	venom carboxylesterase-6
LOC100650077	2.751085788	7.19E-05	Up-regulated	translation initiation factor IF-2
LOC105665724	1.294257498	9.77E-05	Up-regulated	transcription factor kayak
LOC100642776	2.869650839	1.04E-04	Up-regulated	uncharacterized LOC100642776
LOC100650665	2.035427027	1.05E-04	Up-regulated	uncharacterized LOC100650665
LOC100643570	0.715811398	1.19E-04	Up-regulated	40S ribosomal protein S29
LOC100643066	2.752159787	1.22E-04	Up-regulated	bestrophin-4
LOC100645301	1.570834048	1.33E-04	Up-regulated	filamin-A
LOC100651728	2.40808406	1.34E-04	Up-regulated	SET domain-containing protein SmydA-8
LOC100649546	1.325950157	1.34E-04	Up-regulated	uncharacterized LOC100649546
LOC100643291	2.466935995	1.64E-04	Up-regulated	RNA-binding protein 24
LOC100647457	1.129982316	1.64E-04	Up-regulated	leupaxin
LOC100642478	1.756287761	1.90E-04	Up-regulated	myogenic-determination protein
LOC100652204	1.376210148	1.96E-04	Up-regulated	alpha-actinin
LOC100645491	0.515013674	1.96E-04	Up-regulated	60S ribosomal protein L38
LOC100649306	2.780180946	1.97E-04	Up-regulated	chitin deacetylase 1
LOC100651605	0.98632554	1.97E-04	Up-regulated	microtubule-associated protein futsch
LOC100644627	0.966397958	1.97E-04	Up-regulated	heat shock protein beta-1
LOC100651024	0.929681255	1.97E-04	Up-regulated	glycerol-3-phosphate dehydrogenase
LOC110119633	1.64528398	1.98E-04	Up-regulated	myb-like protein X
LOC100649174	3.056713475	2.00E-04	Up-regulated	calcium-dependent protein kinase 16
LOC100651791	1.244292413	2.00E-04	Up-regulated	NAD(+) hydrolase sarm1
LOC100643436	0.411178095	2.00E-04	Up-regulated	cofilin/actin-depolymerizing factor homolog

LOC100647077	-0.192810419	2.00E-04	Down-regulated	protein shuttle craft
LOC100644626	0.777855255	2.13E-04	Up-regulated	40S ribosomal protein S28
LOC100648552	0.492786867	2.13E-04	Up-regulated	40S ribosomal protein S16
LOC100651217	2.178616207	2.25E-04	Up-regulated	sarcoplasmic calcium-binding protein 1
LOC100643342	1.972417331	2.48E-04	Up-regulated	zinc finger protein rotund
LOC100643891	1.239671258	2.48E-04	Up-regulated	zinc carboxypeptidase
LOC100648227	0.734811792	2.48E-04	Up-regulated	uncharacterized LOC100648227
LOC100652068	1.27230799	2.91E-04	Up-regulated	protein lethal(2)essential for life
LOC100649983	1.651558578	3.23E-04	Up-regulated	uncharacterized LOC100649983
LOC100647699	1.448964525	3.54E-04	Up-regulated	two pore potassium channel protein sup-9
LOC100651154	1.776425116	3.70E-04	Up-regulated	uncharacterized LOC100651154
LOC100646145	2.141275753	3.73E-04	Up-regulated	ejaculatory bulb-specific protein 3
LOC100650906	1.663854749	4.01E-04	Up-regulated	esterase FE4
LOC100646026	1.666820273	4.18E-04	Up-regulated	serine/arginine repetitive matrix protein 1
LOC100642652	1.775453129	4.22E-04	Up-regulated	calcium-transporting ATPase sarcoplasmic/endoplasmic reticulum type
LOC105665975	2.500806785	4.46E-04	Up-regulated	golgin subfamily A member 4
LOC100649005	1.699181936	4.63E-04	Up-regulated	homeobox protein ceh-17
LOC100646337	0.762558107	4.80E-04	Up-regulated	CBP80/20-dependent translation initiation factor
LOC100646055	0.733671247	4.80E-04	Up-regulated	ankyrin repeat domain-containing protein 29
LOC100645035	1.943954921	5.24E-04	Up-regulated	mpv17-like protein 2
LOC100649289	0.560804277	5.24E-04	Up-regulated	40S ribosomal protein S13
LOC105666342	0.484602773	5.24E-04	Up-regulated	60S ribosomal protein L18
LOC100643007	2.049710746	5.48E-04	Up-regulated	uncharacterized LOC100643007
LOC100642903	2.162065449	5.60E-04	Up-regulated	myosin regulatory light chain 2
LOC100646075	0.546713975	5.60E-04	Up-regulated	60S ribosomal protein L34
LOC100644723	0.990223942	6.11E-04	Up-regulated	ATP-binding cassette sub-family G member 1
LOC110120039	1.309098409	6.56E-04	Up-regulated	uncharacterized LOC110120039
LOC100644410	0.478645597	6.57E-04	Up-regulated	protein transport protein Sec61 subunit gamma
LOC100642816	5.212400994	7.50E-04	Up-regulated	cytochrome P450 6k1 LOC100642816
LOC105666811	0.54030089	7.50E-04	Up-regulated	40S ribosomal protein S15
LOC105665825	1.886721378	7.60E-04	Up-regulated	treacle protein
LOC100652136	-0.241268845	7.92E-04	Down-regulated	gastrulation defective protein 1 homolog
LOC100645373	0.335522268	8.39E-04	Up-regulated	actin-interacting protein 1
LOC100644990	1.084890364	8.58E-04	Up-regulated	uncharacterized LOC100644990
LOC100646983	1.508167888	9.57E-04	Up-regulated	uncharacterized LOC100646983
LOC105665640	0.403389523	0.001222801	Up-regulated	NADH dehydrogenase [ubiquinone] 1 alpha subcomplex subunit 13
LOC100645201	0.33921082	0.001243254	Up-regulated	RING finger protein 10
LOC100650251	0.753768684	0.001287871	Up-regulated	plexin domain-containing protein 2
LOC100649287	0.261317119	0.001309058	Up-regulated	ubiquitin-conjugating enzyme E2-17 kDa
LOC100648006	2.175723365	0.001484285	Up-regulated	phospholipase B1
LOC105666141	1.927990099	0.001484285	Up-regulated	15-hydroxyprostaglandin dehydrogenase [NAD(+)]
LOC100645803	1.340324887	0.001484285	Up-regulated	nuclear factor 1 X-type
LOC100649801	1.435388214	0.001495952	Up-regulated	uncharacterized LOC100649801
LOC100650837	0.558581845	0.001612126	Up-regulated	40S ribosomal protein S3
LOC100645515	-0.249540856	0.001715604	Down-regulated	kinesin-like protein KIF3A
LOC100643331	0.529557163	0.001745875	Up-regulated	tropomodulin
LOC100651253	0.474242834	0.001775107	Up-regulated	40S ribosomal protein S10
LOC100651772	0.392986056	0.001898744	Up-regulated	ras-related protein RabJ
LOC100647701	0.499551047	0.001934816	Up-regulated	60S ribosomal protein L27a
LOC100644902	1.270516624	0.001966995	Up-regulated	uncharacterized LOC100644902
LOC100644931	1.562824899	0.002053598	Up-regulated	uncharacterized LOC100644931
LOC100649554	1.745734536	0.002066049	Up-regulated	histone-lysine N-methyltransferase SETMAR
LOC100646793	1.391862575	0.002066049	Up-regulated	PDZ and LIM domain protein 3
LOC100642272	0.948153391	0.002066049	Up-regulated	very-long-chain 3-oxoacyl-CoA reductase
LOC100646980	0.219177603	0.002066049	Up-regulated	integrin-linked protein kinase homolog pat-4
LOC100642980	1.431960083	0.002074304	Up-regulated	hemocytin
LOC100643689	1.199029765	0.002117169	Up-regulated	synaptogenesis protein syg-2
LOC100644502	0.346527726	0.002117169	Up-regulated	ATPase inhibitor mai-2
LOC100646463	1.020792803	0.002278025	Up-regulated	uncharacterized LOC100646463
LOC100650573	1.504294491	0.002335386	Up-regulated	endothelin-converting enzyme homolog
LOC100648166	0.668157085	0.002335386	Up-regulated	trimeric intracellular cation channel type 1B.1
LOC105666762	1.252693184	0.00234177	Up-regulated	bone morphogenetic protein 2
LOC100651597	0.321959957	0.002413471	Up-regulated	elongator complex protein 3
LOC100643293	0.973702669	0.002414382	Up-regulated	probable inactive protein kinase DDB_G0270444
LOC100643217	0.845349988	0.00247363	Up-regulated	forkhead box protein O
LOC100652162	0.475176815	0.00247363	Up-regulated	uncharacterized LOC100652162
LOC100643509	0.412260857	0.00247363	Up-regulated	ubiquitin-60S ribosomal protein L40
LOC100647290	1.095772683	0.002503714	Up-regulated	SET and MYND domain-containing protein 4
LOC100649463	-0.413216506	0.002651958	Down-regulated	dynein intermediate chain 2
LOC100642537	0.4043753	0.002723567	Up-regulated	40S ribosomal protein S18
LOC100645631	1.638617102	0.002749082	Up-regulated	sodium-independent sulfate anion transporter
LOC100649873	0.349238493	0.002868442	Up-regulated	ubiquitin-conjugating enzyme E2 G1
LOC100648193	0.205699577	0.003013443	Up-regulated	serine/threonine-protein kinase Pink1
LOC100648516	1.288300392	0.003030443	Up-regulated	sushi
LOC100645362	-0.250436092	0.003030443	Down-regulated	uncharacterized LOC100645362
LOC100642552	0.443371646	0.003059769	Up-regulated	60S ribosomal protein L13a
LOC100649164	0.360595769	0.003059769	Up-regulated	elongation factor 1-beta'
LOC105666366	3.880841194	0.003117837	Up-regulated	cytochrome P450 6k1 LOC105666366
LOC105666782	1.084617128	0.003117837	Up-regulated	uncharacterized LOC105666782
LOC100648128	1.365487839	0.003133142	Up-regulated	uncharacterized LOC100648128
LOC100645713	1.095815744	0.00320327	Up-regulated	uncharacterized LOC100645713

LOC100643996	0.449873356	0.003207349	Up-regulated	probable enoyl-CoA hydratase
LOC100646020	0.646419408	0.003303733	Up-regulated	40S ribosomal protein S29
LOC100651156	1.367824992	0.003342381	Up-regulated	uncharacterized LOC100651156
LOC100646220	1.225944035	0.003342381	Up-regulated	dual oxidase maturation factor 1
LOC100652321	0.992637224	0.00337346	Up-regulated	protein spaetzle 5
LOC100646491	1.872113629	0.003491098	Up-regulated	probable peroxisomal acyl-coenzyme A oxidase 1
LOC105665894	1.023367715	0.003511973	Up-regulated	uncharacterized LOC105665894
LOC100646835	0.971006237	0.003511973	Up-regulated	uncharacterized LOC100646835
LOC100642337	-0.299242792	0.003604163	Down-regulated	uncharacterized LOC100642337
LOC100652147	1.802128367	0.00363634	Up-regulated	peroxisomal hydratase-dehydrogenase-epimerase
LOC100648560	0.380291742	0.003827456	Up-regulated	40S ribosomal protein S8
LOC100642473	0.218055753	0.003881825	Up-regulated	ATP-dependent RNA helicase Ddx1
LOC100648934	0.5193654	0.003968002	Up-regulated	60S ribosomal protein L15
LOC100649799	1.120146641	0.003977747	Up-regulated	transketolase
LOC105666460	0.74896013	0.003977747	Up-regulated	uncharacterized LOC105666460
LOC100647508	0.541202852	0.003977747	Up-regulated	60S ribosomal protein L30
LOC100642733	0.484563285	0.003977747	Up-regulated	60S ribosomal protein L32
LOC100642567	0.231852091	0.003977747	Up-regulated	cytochrome c oxidase subunit 4 isoform 1
LOC100652050	1.140611961	0.004150965	Up-regulated	venom dipeptidyl peptidase 4
LOC100643536	2.133787449	0.004205988	Up-regulated	protein javelin
LOC100642299	0.647451119	0.004205988	Up-regulated	unconventional myosin-XVIIIa
LOC100647894	0.355218898	0.004205988	Up-regulated	60S ribosomal protein L7
LOC100652012	0.441225091	0.004240421	Up-regulated	40S ribosomal protein S26
LOC100648013	0.421413637	0.0044685	Up-regulated	serine/threonine-protein kinase S6KL
LOC105667097	0.58472105	0.004547046	Up-regulated	mitochondrial pyruvate carrier 4
LOC100648962	0.517884369	0.004593886	Up-regulated	60S ribosomal protein L35
LOC100642708	0.361628499	0.004593886	Up-regulated	holocytochrome c-type synthase
LOC100647536	-0.236728804	0.004593886	Down-regulated	islet cell autoantigen 1-like protein
LOC100642397	0.405461488	0.004650121	Up-regulated	uncharacterized LOC100642397
LOC100650495	2.279506459	0.004757167	Up-regulated	troponin C
LOC100646995	1.727105726	0.004757167	Up-regulated	uncharacterized LOC100646995
LOC100647229	1.593608574	0.004757167	Up-regulated	troponin C
LOC100642936	1.565847717	0.004757167	Up-regulated	cytochrome P450 6k1 LOC100642936
LOC100642316	1.176496205	0.004757167	Up-regulated	SET domain-containing protein SmydA-8
LOC100649729	0.369780285	0.004757167	Up-regulated	ras-related protein Rab-43
LOC100652246	-0.316080647	0.004757167	Down-regulated	myoneurin
LOC100643802	0.855805612	0.004787103	Up-regulated	ecdysteroid-regulated 16 kDa protein
LOC100651376	1.76018551	0.004790315	Up-regulated	alpha-tocopherol transfer protein-like
LOC100651337	1.562669009	0.004822746	Up-regulated	acyl-CoA-binding protein homolog
LOC100644439	0.357183991	0.004822746	Up-regulated	myosin regulatory light chain sqh
LOC100648549	0.258035189	0.004822746	Up-regulated	cytochrome c
LOC100649977	0.873104967	0.004990888	Up-regulated	uncharacterized LOC100649977
LOC100644788	0.603505978	0.005000551	Up-regulated	60S ribosomal protein L37
LOC100652238	0.568082739	0.005053478	Up-regulated	60S ribosomal protein L29
LOC100648394	0.503580289	0.005053478	Up-regulated	40S ribosomal protein S12
LOC100643087	0.336621059	0.005053478	Up-regulated	40S ribosomal protein S4
LOC100647191	0.325987537	0.005053478	Up-regulated	eukaryotic translation initiation factor 3 subunit F
LOC100651961	0.323465962	0.005053478	Up-regulated	ADP-ribosylation factor 2
LOC100647623	0.490130867	0.005130524	Up-regulated	adenylate cyclase type 2
LOC100643733	0.446888667	0.005130524	Up-regulated	60S ribosomal protein L14
LOC100647169	0.361523539	0.005130524	Up-regulated	AN1-type zinc finger protein 5
LOC100645595	1.289778687	0.005145627	Up-regulated	retinoid-inducible serine carboxypeptidase
LOC100644819	0.527752966	0.005159996	Up-regulated	fumarylacetoacetase
LOC100645940	0.405170692	0.005159996	Up-regulated	60S ribosomal protein L27
LOC100647053	1.156864504	0.005229364	Up-regulated	hippocalcin-like protein 4
LOC100652184	0.413389606	0.005229364	Up-regulated	60S acidic ribosomal protein P1
LOC100650919	0.219388709	0.005229364	Up-regulated	peroxiredoxin-5
LOC100648426	1.381959963	0.005289757	Up-regulated	acetyl-CoA acetyltransferase
LOC100651996	0.740986789	0.005289757	Up-regulated	transaldolase
LOC100644175	0.507787165	0.005289757	Up-regulated	40S ribosomal protein S20
LOC100647664	0.352754331	0.005289757	Up-regulated	40S ribosomal protein S17
LOC100643821	0.842864962	0.005374157	Up-regulated	protein CREG1
LOC100649782	-0.203176186	0.005446306	Down-regulated	pleckstrin homology domain-containing family M member 2
LOC100643627	0.768468588	0.00546207	Up-regulated	tubulin alpha-1 chain-like
LOC100648124	0.836134449	0.005482414	Up-regulated	glucose-6-phosphate 1-dehydrogenase
LOC100650785	0.86310146	0.00562215	Up-regulated	facilitated trehalose transporter Tret1
LOC100651068	0.435747706	0.005715561	Up-regulated	60S ribosomal protein L11
LOC100643684	0.857494833	0.005800561	Up-regulated	putative phospholipase B-like lamina ancestor
LOC100648165	0.616807108	0.005883035	Up-regulated	phytanoyl-CoA dioxygenase domain-containing protein 1 homolog
LOC100645787	0.197690395	0.005883035	Up-regulated	NEDD8-conjugating enzyme Ubc12
LOC100644187	0.413171433	0.00592387	Up-regulated	60S ribosomal protein L36
LOC100645697	0.205732361	0.005932376	Up-regulated	SR-related and CTD-associated factor 8
LOC100642428	0.296025192	0.006400254	Up-regulated	OTU domain-containing protein 7B
LOC100651918	0.276291539	0.00641908	Up-regulated	26S proteasome regulatory subunit 4
LOC100642748	1.92559314	0.006446556	Up-regulated	diacylglycerol lipase-beta
LOC100645730	0.406689594	0.006446556	Up-regulated	uncharacterized LOC100645730
LOC100648608	0.341944755	0.006446556	Up-regulated	40S ribosomal protein S23
LOC100648890	0.401780382	0.006485801	Up-regulated	60S ribosomal protein L18a
LOC105666285	0.367615614	0.006839346	Up-regulated	60S ribosomal protein L6
LOC100647144	1.009244242	0.006895016	Up-regulated	uncharacterized LOC100647144
LOC100643495	0.416504277	0.006895016	Up-regulated	eukaryotic translation initiation factor 4E-binding protein Mextli

LOC105666292	0.343812573	0.006951725	Up-regulated	dihydrolipoyllysine-residue acetyltransferase component pyruvate dehydrogenase
LOC100642650	0.479309651	0.007066052	Up-regulated	elongation of very long chain fatty acids protein AAEL008004
LOC100651488	0.200525778	0.007066052	Up-regulated	ubiquitin thioesterase OTU1
npve	1.208776915	0.007182607	Up-regulated	acyl-CoA delta-9 desaturase
LOC100650532	0.491498507	0.007195647	Up-regulated	60S ribosomal protein L8
LOC100645322	1.020682926	0.007199916	Up-regulated	doublesex- and mab-3-related transcription factor A2
LOC100647299	0.484903069	0.007199916	Up-regulated	uncharacterized LOC100647299
LOC100646404	-0.211647392	0.007199916	Down-regulated	PDZ domain-containing protein 8
LOC100642321	1.253051388	0.007233781	Up-regulated	D-galactonate transporter
LOC100645678	1.13874274	0.007234326	Up-regulated	laminin subunit alpha
LOC100644295	0.434615918	0.007234326	Up-regulated	reticulon-4-interacting protein 1 homolog
LOC100649714	0.974637897	0.007273217	Up-regulated	calcyphosin-like protein
LOC100650085	1.879342791	0.007307302	Up-regulated	uncharacterized LOC100650085
LOC100651584	0.407105248	0.007423822	Up-regulated	ras-related protein Rab-18
LOC100643234	0.364493148	0.007470526	Up-regulated	RING-box protein 1A
LOC100651763	0.52265394	0.007482766	Up-regulated	40S ribosomal protein S23
LOC100645181	0.507017891	0.007482766	Up-regulated	segmentation protein cap'n'collar
LOC100650905	0.460115204	0.007556248	Up-regulated	adiponectin receptor protein
LOC100649638	0.400786115	0.007556248	Up-regulated	60S ribosomal protein L4
LOC100646012	1.830146791	0.007568608	Up-regulated	isopentenyl-diphosphate Delta-isomerase 1-like
LOC100642418	1.516600043	0.007623409	Up-regulated	receptor-type tyrosine-protein phosphatase delta
LOC100650007	-0.243318026	0.007623409	Down-regulated	NCK-interacting protein with SH3 domain
LOC100647475	0.325495016	0.007819202	Up-regulated	CTD nuclear envelope phosphatase 1 homolog
LOC100643669	2.506405946	0.007888392	Up-regulated	elongation of very long chain fatty acids protein 6
LOC100647965	0.775608893	0.007888392	Up-regulated	monocarboxylate transporter 12-B
LOC100644545	0.352403327	0.00791081	Up-regulated	proteasome subunit alpha type-2
LOC100645096	-0.259822564	0.00791081	Down-regulated	CUE domain-containing protein 2-A
LOC100646336	-0.254400994	0.007936831	Down-regulated	multiple inositol polyphosphate phosphatase 1
LOC100650566	0.821505957	0.007963683	Up-regulated	protein henna
LOC100651862	0.30846292	0.007998229	Up-regulated	3-ketoacyl-CoA thiolase
LOC100645028	0.380115013	0.008133386	Up-regulated	60S ribosomal protein L19
LOC110120071	0.696882695	0.008178712	Up-regulated	60S ribosomal protein L39
LOC100644276	0.561760617	0.008178712	Up-regulated	60S ribosomal protein L28
LOC100647377	0.442619823	0.008178712	Up-regulated	40S ribosomal protein S25
LOC100647610	0.41291673	0.008178712	Up-regulated	40S ribosomal protein S15Aa
LOC100652292	0.36470988	0.008178712	Up-regulated	prolyl 3-hydroxylase OGFOD1
LOC100646258	-0.350008725	0.008178712	Down-regulated	USP6 N-terminal-like protein
LOC100643127	0.523534221	0.008245136	Up-regulated	ras-related protein Rab-10
LOC100645695	0.445463244	0.008245136	Up-regulated	60S ribosomal protein L31
LOC105666640	1.463645147	0.008378661	Up-regulated	D-aspartate oxidase
LOC100646699	1.365713628	0.008398512	Up-regulated	tektin-1
LOC100644293	0.423079112	0.008398512	Up-regulated	60S ribosomal protein L9
LOC100646721	0.552573569	0.008399459	Up-regulated	venom acid phosphatase Acph-1
LOC100645456	1.868514592	0.008404193	Up-regulated	gonadotropin-releasing hormone receptor
LOC100648641	0.439113936	0.008447622	Up-regulated	peroxisomal multifunctional enzyme type 2
LOC100648112	0.516246043	0.008643637	Up-regulated	uncharacterized LOC100648112
LOC100647667	0.531458106	0.008658535	Up-regulated	uncharacterized LOC100647667
LOC100646610	0.392513238	0.008658535	Up-regulated	60S ribosomal protein L7a
LOC100646399	0.69966088	0.008678327	Up-regulated	uncharacterized LOC100646399
LOC100650547	0.589862215	0.008691894	Up-regulated	60S ribosomal protein L44
LOC100647269	0.506963681	0.008691894	Up-regulated	40S ribosomal protein S19
LOC100645665	0.86286393	0.008706938	Up-regulated	uncharacterized LOC100645665
LOC100652020	0.395554721	0.00890301	Up-regulated	60S ribosomal protein L23a
LOC100650427	5.286016088	0.009011207	Up-regulated	cytochrome P450 6k1 LOC100650427
LOC100645898	1.104542664	0.009011207	Up-regulated	T-box transcription factor TBX10
LOC100646339	0.889146331	0.009011207	Up-regulated	sestrin homolog
LOC100644616	0.47291883	0.009011207	Up-regulated	ankyrin repeat and SOCS box protein 16
LOC100649561	0.461757912	0.009011207	Up-regulated	laminin subunit alpha-1
LOC100652254	0.347541675	0.009011207	Up-regulated	protein dj-1beta
LOC100645941	0.272452856	0.009011207	Up-regulated	40S ribosomal protein S24
LOC100650064	-0.294280817	0.009011207	Down-regulated	mitogen-activated protein kinase kinase kinase 4
LOC110120284	1.29212452	0.00913015	Up-regulated	uncharacterized LOC110120284
LOC100650698	0.249929393	0.009264142	Up-regulated	succinate dehydrogenase [ubiquinone] cytochrome b small subunit
LOC100645227	0.267240442	0.009419939	Up-regulated	glycerophosphocholine phosphodiesterase GPCPD1
LOC100648257	0.996043576	0.009585761	Up-regulated	probable cytochrome P450 6a14 LOC100648257
LOC100649541	0.482094131	0.009585761	Up-regulated	60S acidic ribosomal protein P0
LOC100643288	0.450215923	0.009907747	Up-regulated	protein limb expression 1 homolog
LOC100645203	2.170439847	0.010688399	Up-regulated	uncharacterized LOC100645203
LOC105667110	1.105754799	0.010688399	Up-regulated	organic cation transporter protein
LOC105667204	0.300056665	0.010688399	Up-regulated	pyruvate kinase
LOC100648461	0.460174449	0.010710525	Up-regulated	60S ribosomal protein L26
LOC105666381	0.234185286	0.010710525	Up-regulated	protein FAM210A
LOC100651532	-0.202561615	0.010710525	Down-regulated	serine/threonine-protein kinase 3
LOC100645129	0.422193984	0.010711076	Up-regulated	60S ribosomal protein L35a
LOC100645452	1.140625965	0.010718774	Up-regulated	apolipoprotein D
LOC100650807	0.668498992	0.010944549	Up-regulated	uncharacterized LOC100650807
LOC100642741	0.304212608	0.011068608	Up-regulated	39S ribosomal protein L35
LOC100650372	1.629451907	0.011104357	Up-regulated	endothelin-converting enzyme 1
LOC100650045	0.899638775	0.011154862	Up-regulated	cuticlin-4
LOC100649393	1.143227623	0.011194734	Up-regulated	ABC transporter G family member 20
LOC100652137	0.441687342	0.011194734	Up-regulated	28 kDa heat- and acid-stable phosphoprotein

LOC100631066	0.353717202	0.011194734	Up-regulated	ribosomal protein S5a
LOC100650136	0.395749963	0.011407792	Up-regulated	adenylosuccinate synthetase
LOC100651544	0.371272598	0.011496309	Up-regulated	40S ribosomal protein S7
LOC100646901	-0.277617871	0.011498566	Down-regulated	probable ATP-dependent RNA helicase spindle-E
LOC100643394	1.028072488	0.011996003	Up-regulated	F-box/LRR-repeat protein fbxl-1
LOC100650613	-0.228974461	0.012115762	Down-regulated	protein real-time
LOC100649312	0.267230864	0.012162239	Up-regulated	max-like protein X
LOC100646422	1.026364945	0.012281896	Up-regulated	putative glycerol kinase 5
LOC100643255	0.241346454	0.01254472	Up-regulated	probable pyruvate dehydrogenase E1 component subunit alpha
LOC110120152	0.836512678	0.0125792	Up-regulated	vinexin-like
LOC100650936	0.460548926	0.012679689	Up-regulated	protein roadkill
LOC100644780	0.339456252	0.012793107	Up-regulated	thioredoxin
LOC100647540	1.98081584	0.012806976	Up-regulated	uncharacterized LOC100647540
LOC100651218	0.314809666	0.012948327	Up-regulated	methionine aminopeptidase 1
LOC105665982	1.163920733	0.012967781	Up-regulated	uncharacterized LOC105665982
LOC100650103	-0.256576977	0.013397703	Down-regulated	DNA fragmentation factor subunit alpha
LOC100642562	0.820378239	0.013727009	Up-regulated	CCAAT/enhancer-binding protein
LOC105665862	-0.291808604	0.013732125	Down-regulated	ras-related protein Rab-8A
LOC100644802	0.35437225	0.013763541	Up-regulated	twisted gastrulation protein homolog 1-B
LOC100651517	1.245249632	0.013842387	Up-regulated	RNA-binding protein fusilli
LOC100647765	1.466614462	0.014163973	Up-regulated	toll-like receptor 3
LOC100646892	1.275015347	0.014163973	Up-regulated	homeobox protein aristaless
LOC105666637	0.832378147	0.014163973	Up-regulated	calcium-independent protein kinase C
LOC100645442	0.875033778	0.01420256	Up-regulated	calmodulin-lysine N-methyltransferase
LOC110119673	0.556301525	0.014301806	Up-regulated	uncharacterized LOC110119673
LOC100651796	0.988608649	0.014359789	Up-regulated	organic cation transporter protein
LOC100649759	0.461693884	0.014359789	Up-regulated	REPTOR-binding partner
LOC100647074	-0.276413043	0.014359789	Down-regulated	vacuolar protein sorting-associated protein 13C
LOC100644403	0.314496541	0.014383379	Up-regulated	ATP-binding cassette subfamily G member 4
LOC110119150	0.673039784	0.014459007	Up-regulated	sperm-associated antigen 6-like
LOC100651829	0.375417927	0.014459007	Up-regulated	proteasome subunit alpha type-6
LOC100649098	-0.194973953	0.014556672	Down-regulated	DNA (cytosine-5)-methyltransferase PliMCI
LOC100649726	0.477428445	0.01459003	Up-regulated	40S ribosomal protein S14a
LOC100648276	0.415643206	0.01459003	Up-regulated	40S ribosomal protein S11
LOC100648497	0.242571405	0.01459003	Up-regulated	MIP18 family protein galla-1
LOC100649969	0.395734866	0.014631351	Up-regulated	electron transfer flavoprotein subunit beta
LOC105667135	0.166102069	0.014631351	Up-regulated	E3 ubiquitin-protein ligase RNF185
LOC105666474	-0.256258658	0.014631351	Down-regulated	pyruvate dehydrogenase phosphatase regulatory subunit
LOC100649961	0.884156962	0.014756825	Up-regulated	glycogen-binding subunit 76A
LOC100645335	0.808099684	0.014756825	Up-regulated	uncharacterized LOC100645335
LOC105666621	0.359554583	0.014756825	Up-regulated	27 kDa hemolymph protein
LOC100650019	0.225772416	0.014758725	Up-regulated	protein ILRUN
LOC100649975	0.60430568	0.014804635	Up-regulated	uncharacterized LOC100649975
LOC100644850	1.220239217	0.014819012	Up-regulated	diphosphomevalonate decarboxylase
LOC100648690	0.392071941	0.014819012	Up-regulated	CLIP-associating protein 1-A
LOC100647544	0.17819286	0.014819012	Up-regulated	elongation factor 1-gamma
LOC105666248	-0.250183476	0.014819012	Down-regulated	sulfhydryl oxidase 1-like
LOC100644818	-0.249928	0.014827679	Down-regulated	MOB kinase activator-like 1
LOC100650864	0.706591978	0.014916464	Up-regulated	uncharacterized LOC100650864
LOC100648967	-0.27215968	0.014942062	Down-regulated	uncharacterized LOC100648967
LOC100647727	0.781986064	0.015097168	Up-regulated	uncharacterized LOC100647727
LOC100647066	0.803415798	0.015452472	Up-regulated	sterol carrier protein 2
LOC100650572	1.646059081	0.015577289	Up-regulated	scavenger receptor class B member 1
LOC100645047	1.192339932	0.015662615	Up-regulated	alpha-(1
LOC100647615	0.322767147	0.015725155	Up-regulated	iroquois-class homeodomain protein IRX-6
LOC100652045	-0.187840407	0.015725155	Down-regulated	alsin
LOC100646998	-0.286439266	0.015725155	Down-regulated	gamma-tubulin complex component 6
LOC100643197	-0.263137769	0.016157384	Down-regulated	palmitoyltransferase Hip14
LOC100642305	-0.311927325	0.016163426	Down-regulated	guanine nucleotide-binding protein G(o) subunit alpha
LOC100645460	0.460608463	0.016415493	Up-regulated	uncharacterized LOC100645460
LOC100650481	0.913596285	0.016449906	Up-regulated	uncharacterized LOC100650481
LOC100650923	0.618768571	0.016490282	Up-regulated	follistatin-A
LOC100648435	0.257852545	0.016502911	Up-regulated	26S proteasome non-ATPase regulatory subunit 9
LOC100648631	0.257852545	0.016502911	Up-regulated	26S proteasome non-ATPase regulatory subunit 9
LOC100644120	-0.176286846	0.016567955	Down-regulated	protein phosphatase 1H
LOC100650319	0.601569104	0.016620717	Up-regulated	dual specificity protein phosphatase 10
LOC105666558	0.398806009	0.016644061	Up-regulated	peroxiredoxin 1
LOC100651057	0.181028631	0.016889594	Up-regulated	LIM and senescent cell antigen-like-containing domain protein 1
LOC100645775	1.023746623	0.016941364	Up-regulated	glutathione S-transferase 1-1
LOC100644920	0.389629253	0.016950305	Up-regulated	60S ribosomal protein L37a
LOC100649119	0.58869592	0.016983804	Up-regulated	phospholipase A2 inhibitor
LOC100642984	0.21523257	0.017095292	Up-regulated	anaphase-promoting complex subunit 10
LOC100648465	0.264304988	0.017261891	Up-regulated	electron transfer flavoprotein subunit alpha
LOC100649013	0.194510662	0.017261891	Up-regulated	ras-like GTP-binding protein Rho1
LOC100642781	0.206534895	0.017481304	Up-regulated	eukaryotic initiation factor 4A-I
LOC100642933	-0.456891936	0.017555331	Down-regulated	iron-sulfur clusters transporter ABCB7
LOC100645299	0.732688134	0.017660045	Up-regulated	protein bowel
LOC100645796	0.787579906	0.017795066	Up-regulated	sarcospan
LOC100643095	0.806497457	0.017916831	Up-regulated	uncharacterized LOC100643095
LOC100650555	0.754758162	0.017916831	Up-regulated	fork head domain transcription factor slp1-like
LOC100645221	1.938343429	0.018162087	Up-regulated	ABC transporter G family member 20

LOC100646544	1.467931448	0.018162087	Up-regulated	uncharacterized LOC100646544
LOC100646822	1.210796998	0.018258198	Up-regulated	clavesin-2
LOC100652331	0.655740399	0.018749391	Up-regulated	microsomal glutathione S-transferase 1
LOC100644928	0.117049715	0.018749391	Up-regulated	vesicle-associated membrane protein/synaptobrevin-binding protein
LOC100643774	-0.262159763	0.018872751	Down-regulated	protein pigeon
LOC100643454	0.33536226	0.01904843	Up-regulated	5'-deoxynucleotidase HDDC2
LOC100648473	1.420508394	0.019143112	Up-regulated	2-acylglycerol O-acyltransferase 1
LOC100649883	0.614203517	0.019143112	Up-regulated	uncharacterized LOC100649883
LOC100648322	0.357583827	0.019143112	Up-regulated	peptidoglycan-recognition protein LC
LOC100650893	0.269864898	0.019173435	Up-regulated	acyl-coenzyme A diphosphatase FITM2
LOC100646597	0.400156122	0.019353859	Up-regulated	transmembrane GTPase Marf
LOC100647557	0.300151374	0.019353859	Up-regulated	ras suppressor protein 1
LOC100642381	0.182930136	0.019353859	Up-regulated	eukaryotic translation initiation factor 3 subunit G
LOC100650926	-0.292857212	0.019353859	Down-regulated	transmembrane protein 47
LOC105667175	-0.39652655	0.019353859	Down-regulated	monocarboxylate transporter 9
LOC100646920	0.64271978	0.019421911	Up-regulated	uncharacterized LOC100646920
LOC100642892	0.309258171	0.019698302	Up-regulated	trifunctional enzyme subunit alpha
LOC100645904	0.43504137	0.019746309	Up-regulated	40S ribosomal protein S21
LOC100645052	1.20045059	0.019787013	Up-regulated	neprilysin-4
LOC100645225	0.501934132	0.019787013	Up-regulated	60S acidic ribosomal protein P2
LOC100650219	0.904405572	0.019795585	Up-regulated	ATP-citrate synthase
LOC100643482	0.211533748	0.019838753	Up-regulated	nucleolysin TIAR
LOC100647908	0.630018285	0.019841538	Up-regulated	mevalonate kinase
LOC100643050	1.53423068	0.019976215	Up-regulated	sodium channel protein Nach
LOC100642874	0.343500066	0.020063175	Up-regulated	60S ribosomal protein L5
LOC105665747	0.242243998	0.020063175	Up-regulated	NADH dehydrogenase [ubiquinone] 1 alpha subcomplex subunit 5
LOC100645621	0.434229981	0.020101007	Up-regulated	pyruvate dehydrogenase E1 component subunit beta
LOC100644590	0.291458729	0.020101007	Up-regulated	uncharacterized LOC100644590
LOC100649202	0.814639957	0.020561418	Up-regulated	neuropeptides capa receptor
LOC100648451	2.319786129	0.020625198	Up-regulated	3,4-dihydroxyphenylacetaldehyde synthase
LOC105666419	0.196635962	0.020625198	Up-regulated	ornithine decarboxylase antizyme 1
LOC100646566	0.882965519	0.020870975	Up-regulated	drebrin-like protein
LOC100645214	0.632641549	0.020870975	Up-regulated	6-phosphogluconate dehydrogenase
LOC100649507	-0.25459371	0.020870975	Down-regulated	E3 ubiquitin-protein ligase RNF123
LOC100648676	-0.290545468	0.020870975	Down-regulated	uncharacterized LOC100648676
LOC100651787	1.118503177	0.020945943	Up-regulated	3-hydroxy-3-methylglutaryl-coenzyme A reductase
LOC100644874	0.665748024	0.021121464	Up-regulated	uncharacterized protein CG43427
LOC100642544	0.34371909	0.021242977	Up-regulated	FAU ubiquitin-like and ribosomal protein S30
LOC100643421	0.297587043	0.021242977	Up-regulated	60S ribosomal protein L23
LOC100649310	0.231378608	0.021242977	Up-regulated	protein D2
LOC100648375	0.683356397	0.021431774	Up-regulated	NADP-dependent malic enzyme
LOC100651552	0.811413496	0.021460286	Up-regulated	thyroid receptor-interacting protein 11
LOC100646227	2.074212506	0.021477467	Up-regulated	peroxidase
LOC100649262	0.418669752	0.021655347	Up-regulated	18S rRNA aminocarboxypropyltransferase
LOC100651229	0.340459436	0.021655347	Up-regulated	uncharacterized LOC100651229
LOC100646649	0.247302192	0.021655347	Up-regulated	ragulator complex protein LAMTOR1
LOC105666139	1.280534825	0.021762007	Up-regulated	15-hydroxyprostaglandin dehydrogenase [NAD(+)]
LOC100631054	0.214090456	0.02177452	Up-regulated	dorsal
LOC100648381	-0.26502466	0.02177452	Down-regulated	potassium channel subfamily T member 2
LOC100649035	0.432635623	0.021863804	Up-regulated	BCL2/adenovirus E1B 19 kDa protein-interacting protein 3
LOC100651326	-0.193884374	0.021863804	Down-regulated	RING finger and CHY zinc finger domain-containing protein 1
LOC100646914	0.786937669	0.021986624	Up-regulated	circadian clock-controlled protein daywake
LOC100642716	-0.1651085	0.022045459	Down-regulated	protein PAT1 homolog 1
LOC100643507	-0.279440582	0.022045459	Down-regulated	trypsin-3
LOC100645053	0.640118669	0.022189881	Up-regulated	adenylate cyclase type 10
LOC100643177	0.201453948	0.022362573	Up-regulated	5'-AMP-activated protein kinase catalytic subunit alpha-2
LOC100646205	1.250295089	0.022400272	Up-regulated	uncharacterized LOC100646205
LOC100645503	0.74325047	0.022400272	Up-regulated	protein phosphatase 1 regulatory subunit 3C-B
LOC100648602	0.588061822	0.022400272	Up-regulated	antichymotrypsin-2
LOC100645144	0.296907767	0.022400272	Up-regulated	protein max
LOC100644418	0.23865537	0.022400272	Up-regulated	ATP-dependent RNA helicase abstrakt
LOC100652196	0.134750929	0.022400272	Up-regulated	T-complex protein 1 subunit zeta
LOC100642854	-0.17926177	0.022400272	Down-regulated	uncharacterized LOC100642854
LOC100651187	1.194694833	0.02247004	Up-regulated	uncharacterized LOC100651187
LOC100643080	0.516955497	0.022477824	Up-regulated	adenylate kinase isoenzyme 1
LOC100648453	0.551471379	0.022521715	Up-regulated	pyruvate carboxylase
LOC100645465	0.432266494	0.022521715	Up-regulated	60S ribosomal protein L22
LOC100644975	0.251032097	0.02275447	Up-regulated	peroxiredoxin-6
LOC100643824	0.496245832	0.022959372	Up-regulated	long-chain-fatty-acid--CoA ligase 1
LOC100643743	0.297845854	0.023111562	Up-regulated	40S ribosomal protein S9
LOC100648694	0.612870074	0.02327033	Up-regulated	glutamate receptor-interacting protein 1
LOC100645087	0.25397515	0.02338384	Up-regulated	transcription initiation factor TFIID subunit 9
LOC100649217	0.208829022	0.02338384	Up-regulated	cytochrome c oxidase subunit 6b-3
LOC100645140	-0.177207769	0.02373034	Down-regulated	protein phosphatase PP2A 55 kDa regulatory subunit
LOC100649851	0.411012329	0.023763843	Up-regulated	60S ribosomal protein L17
LOC100650720	0.360128281	0.023904756	Up-regulated	annexin B9
LOC100645701	-0.234180058	0.023904756	Down-regulated	protein ecdysoneless homolog
LOC100649396	0.263411518	0.023966485	Up-regulated	autophagy-related protein 9A
LOC100647581	-0.432473369	0.023966485	Down-regulated	iodotyrosine deiodinase 1
LOC100631087	0.412541442	0.023976984	Up-regulated	arginine kinase
LOC100647155	0.283254608	0.023976984	Up-regulated	eukaryotic peptide chain release factor GTP-binding subunit ERF3A

LOC100642289	-0.257208606	0.023976984	Down-regulated	equilibrative nucleoside transporter 4
LOC100647007	-0.42671333	0.023976984	Down-regulated	uncharacterized LOC100647007
LOC100648350	0.298602555	0.024188246	Up-regulated	ferritin-3
LOC100645759	0.308702873	0.02426309	Up-regulated	uncharacterized LOC100645759
LOC100650962	0.549104045	0.024373198	Up-regulated	F-actin-monooxygenase Mical
LOC100642694	0.270536761	0.024373198	Up-regulated	CKLF-like MARVEL transmembrane domain-containing protein 4
LOC100649601	1.272708252	0.024692794	Up-regulated	elongation of very long chain fatty acids protein 6
LOC100651420	0.345106819	0.025061039	Up-regulated	nuclear receptor-binding factor 2
LOC100646095	1.506841157	0.025242305	Up-regulated	uncharacterized protein PF3D7_1120600
LOC100649627	1.429074131	0.025242305	Up-regulated	4-nitrophenylphosphatase
LOC100650010	0.638271617	0.025242305	Up-regulated	echinoderm microtubule-associated protein-like 2
LOC100647931	0.379678137	0.025242305	Up-regulated	rab11 family-interacting protein 4A
LOC100652215	0.247037972	0.025242305	Up-regulated	ubiquitin-conjugating enzyme E2 R2
LOC100649297	0.197734908	0.025242305	Up-regulated	importin subunit alpha-3
LOC100650937	-0.388346447	0.025242305	Down-regulated	putative ammonium transporter 3
LOC100646669	-0.390855388	0.025242305	Down-regulated	uncharacterized LOC100646669
LOC100651799	-0.647311456	0.025242305	Down-regulated	uncharacterized LOC100651799
LOC100645654	0.275480639	0.025413927	Up-regulated	chloride intracellular channel exc-4
LOC100649919	1.706067146	0.025448536	Up-regulated	nose resistant to fluoxetine protein 6
LOC100648830	0.173369989	0.025448536	Up-regulated	glyceraldehyde-3-phosphate dehydrogenase 2
LOC100644311	-0.199569609	0.025448536	Down-regulated	regulator of G-protein signaling 7
LOC100650822	0.15777801	0.025513154	Up-regulated	hsp70-binding protein 1
LOC100643602	0.894870886	0.025683164	Up-regulated	endocuticle structural glycoprotein SgAbd-1
LOC100646662	0.372093406	0.025683164	Up-regulated	superoxide dismutase [Cu-Zn]
LOC100647345	-0.159302687	0.025683164	Down-regulated	serine/threonine-protein phosphatase PP1-beta catalytic subunit
LOC100651717	-0.279428541	0.025683164	Down-regulated	spastin
LOC100643728	-0.307705858	0.025683164	Down-regulated	laccase-1
LOC100645369	0.528814417	0.02571607	Up-regulated	excitatory amino acid transporter 1
LOC100645303	-0.234040874	0.02571607	Down-regulated	phosphatidylinositide phosphatase SAC2
LOC100650348	0.416561046	0.026057616	Up-regulated	zinc finger protein Noc
LOC100651434	0.459937356	0.026226608	Up-regulated	40S ribosomal protein S3-like
LOC100644288	0.494477241	0.026754935	Up-regulated	papilin
LOC100649189	0.323635648	0.027214065	Up-regulated	60S ribosomal protein L24
LOC100651084	-0.213765755	0.027253973	Down-regulated	gamma-1-syntrophin
LOC100644612	0.245694435	0.027381865	Up-regulated	proteasome subunit beta type-7
LOC100642862	0.341760734	0.027772769	Up-regulated	DNA-directed RNA polymerases I
LOC100644083	0.130739791	0.027921885	Up-regulated	NADH dehydrogenase [ubiquinone] 1 alpha subcomplex subunit 9
LOC100650575	0.231846166	0.027930776	Up-regulated	isochorismatase domain-containing protein 2
LOC100651257	0.195764263	0.028215091	Up-regulated	splicing factor 3A subunit 2
LOC100643869	1.119920227	0.028347862	Up-regulated	scavenger receptor class B member 1
LOC100644989	0.290960452	0.028748453	Up-regulated	thyroid transcription factor 1-associated protein 26
LOC105666376	-0.260807642	0.029263599	Down-regulated	protein tyrosine phosphatase domain-containing protein 1
LOC100644568	0.234682689	0.029595121	Up-regulated	ATP synthase-coupling factor 6
LOC100647939	1.356888096	0.029598376	Up-regulated	tyrosine aminotransferase
LOC100648519	0.244309523	0.029825211	Up-regulated	mitochondrial folate transporter/carrier
LOC105666874	-0.55281208	0.029825211	Down-regulated	uncharacterized LOC105666874
LOC100650305	-0.363382772	0.029944306	Down-regulated	cilia- and flagella-associated protein 44
LOC100645601	0.36380819	0.030129952	Up-regulated	SPRY domain-containing protein 7
LOC100645172	0.185760343	0.030328982	Up-regulated	probable phosphoglycerate kinase
LOC100643828	-0.211954536	0.030449622	Down-regulated	voltage-dependent calcium channel gamma-7 subunit
LOC100644341	0.939974687	0.030457921	Up-regulated	uncharacterized LOC100644341
LOC100642771	0.427886576	0.030457921	Up-regulated	fasciclin-3
LOC100631093	0.19260612	0.030457921	Up-regulated	TGF-beta activated kinase 1
LOC105666002	1.227309906	0.03056316	Up-regulated	uncharacterized LOC105666002
LOC100646772	0.539556162	0.03056316	Up-regulated	mediator of RNA polymerase II transcription subunit 22
LOC100644434	0.384403194	0.03056316	Up-regulated	SAGA-associated factor 29
LOC100648105	0.366629151	0.03056316	Up-regulated	filamin-A
LOC100643010	0.898255045	0.03074593	Up-regulated	transcriptional regulator ovo
LOC100644488	0.36339179	0.030864288	Up-regulated	homeobox protein 14
LOC100648661	0.475453109	0.03090424	Up-regulated	slit homolog 1 protein
LOC100650950	0.565397707	0.031027036	Up-regulated	uncharacterized LOC100650950
LOC100643044	-0.209117302	0.031422753	Down-regulated	probable serine/threonine-protein kinase roco9
LOC105666325	1.201464506	0.031558973	Up-regulated	protein lethal(2)essential for life
LOC100651987	0.873445745	0.031558973	Up-regulated	homogentisate 1
LOC100646806	0.762062638	0.031558973	Up-regulated	hepatocyte nuclear factor 4-gamma
LOC100649884	0.707006238	0.031558973	Up-regulated	myocyte-specific enhancer factor 2
LOC100646306	0.378091972	0.031558973	Up-regulated	ribulose-phosphate 3-epimerase
LOC100649625	0.241645253	0.031613982	Up-regulated	putative ATP synthase subunit f
LOC100650013	-0.252530833	0.031685839	Down-regulated	uncharacterized LOC100650013
LOC100642878	0.493783573	0.03170045	Up-regulated	atlastin
LOC105666158	1.18790763	0.031871422	Up-regulated	uncharacterized LOC105666158
LOC100649108	0.265323524	0.032430561	Up-regulated	stromal interaction molecule homolog
LOC100651887	0.780432947	0.032452043	Up-regulated	extracellular matrix protein 2
LOC100648993	0.233756929	0.032452043	Up-regulated	galactose mutarotase
LOC100643465	-0.664477132	0.032503155	Down-regulated	urea transporter 1
LOC100645101	0.775801604	0.032836173	Up-regulated	inducible metalloproteinase inhibitor protein
LOC100644069	0.31271387	0.032836173	Up-regulated	phosphorylase b kinase gamma catalytic chain
LOC100646604	0.231176767	0.03294618	Up-regulated	charged multivesicular body protein 3
LOC100644399	0.465258498	0.032979387	Up-regulated	unc-112-related protein
LOC100650199	0.467368621	0.033098868	Up-regulated	myosin-VIIa
LOC100645866	-0.182718006	0.033665316	Down-regulated	protein smoothened

LOC100649728	-0.482784935	0.03373686	Down-regulated	uncharacterized LOC100649728	
LOC100649997	0.424290954	0.03388014	Up-regulated	probable medium-chain specific acyl-CoA dehydrogenase	
LOC100649338	0.352039009	0.03388014	Up-regulated	proteoglycan 4	
LOC100644117	-0.250352195	0.033938904	Down-regulated	leishmanolysin-like peptidase	
LOC100650988	0.783973247	0.034068686	Up-regulated	uncharacterized LOC100650988	
LOC100642551	0.207249515	0.034235992	Up-regulated	lipoyl synthase	
LOC100642820	-0.201651456	0.034235992	Down-regulated	wolframin	
LOC100642983	0.731702025	0.034241888	Up-regulated	protein bicaudal C homolog 1-B	
LOC100646005	0.345914044	0.034258039	Up-regulated	40S ribosomal protein S3a	
LOC100643712	0.749990715	0.034273155	Up-regulated	dolichyldiphosphatase 1	
LOC100642504	-0.374715729	0.034273155	Down-regulated	trichohyalin	
LOC100645915	-0.276908404	0.034622341	Down-regulated	potassium/sodium hyperpolarization-activated cyclic nucleotide-gated channel 2	
LOC100643950	0.572961433	0.034635373	Up-regulated	protein hairy	
LOC100646049	0.182389691	0.035181538	Up-regulated	uncharacterized LOC100646049	
LOC105666145	0.87636232	0.035200193	Up-regulated	uncharacterized LOC105666145	
LOC100647428	0.378106812	0.035218139	Up-regulated	26S proteasome non-ATPase regulatory subunit 8	
LOC100649109	0.36749485	0.035218139	Up-regulated	tRNA (adenine(58)-N(1))-methyltransferase catalytic subunit TRMT61A	
LOC100650519	0.220888828	0.035218139	Up-regulated	cytochrome c oxidase subunit NDUFA4	
LOC100645765	0.194351812	0.035218139	Up-regulated	ATP synthase subunit delta	
LOC100649182	0.295449832	0.035254865	Up-regulated	adenylyl cyclase-associated protein 2	
LOC100645116	-0.240590345	0.035413865	Down-regulated	zinc finger protein 420	
LOC105666393	-0.711277956	0.035520618	Down-regulated	uncharacterized LOC105666393	
LOC100644189	0.351482084	0.035776016	Up-regulated	DNA-directed RNA polymerases I	
LOC100643187	0.164168472	0.035776016	Up-regulated	cobalamin trafficking protein CblD	
LOC100650189	0.396233131	0.035789593	Up-regulated	UPF0235 protein C15orf40 homolog	
LOC100648377	-0.125742045	0.035833315	Down-regulated	sorting nexin-14	
LOC100648835	-0.151801104	0.035878898	Down-regulated	two pore calcium channel protein 1	
LOC100649092	-0.403905869	0.036289227	Down-regulated	uncharacterized LOC100649092	
LOC100642931	0.367188774	0.036755238	Up-regulated	glycogen phosphorylase	
LOC100645229	0.254062041	0.036798395	Up-regulated	60S ribosomal protein L12	
LOC100650718	1.401078733	0.036976471	Up-regulated	uncharacterized LOC100650718	
LOC100643880	0.218855019	0.036976471	Up-regulated	26S proteasome non-ATPase regulatory subunit 14	
LOC100642633	0.445345383	0.037057481	Up-regulated	xylulose kinase	
LOC100644841	1.2124066	0.037691008	Up-regulated	cubilin	
LOC100647192	-0.14859877	0.037691008	Down-regulated	E3 ubiquitin-protein ligase RBBP6	
LOC100649916	0.871365246	0.037732236	Up-regulated	KN motif and ankyrin repeat domain-containing protein 2	
LOC100645565	-0.250314419	0.038099793	Down-regulated	kinesin-associated protein 3	
LOC100651735	0.2562682	0.038714164	Up-regulated		1
LOC100651413	0.347401352	0.038910273	Up-regulated	U6 snRNA-associated Sm-like protein LSm7	
LOC100647418	0.643829277	0.039008501	Up-regulated	ERAD-associated E3 ubiquitin-protein ligase HRD1B	
LOC100651658	1.647380878	0.039119719	Up-regulated	uncharacterized LOC100651658	
LOC100650221	0.727680799	0.039119719	Up-regulated	four-domain proteases inhibitor	
LOC100642852	0.322566208	0.039119719	Up-regulated	bublin coiled-coil protein	
LOC100642623	0.285344527	0.039119719	Up-regulated	26S proteasome regulatory subunit 6B	
LOC100646810	-0.244058454	0.039323685	Down-regulated	uridine phosphorylase 1	
LOC100642658	0.328552042	0.039369253	Up-regulated	cytosolic Fe-S cluster assembly factor NUBP2 homolog	
LOC100648518	-0.17670226	0.039818996	Down-regulated	VPS35 endosomal protein-sorting factor-like	
LOC100648422	-0.257724002	0.040006973	Down-regulated	protein inturned	
LOC100644292	0.49971996	0.04001199	Up-regulated	MOB kinase activator-like 2	
LOC100644765	0.440405103	0.04001199	Up-regulated	CUGBP Elav-like family member 2	
LOC100650604	0.406883842	0.04001199	Up-regulated	protein N-terminal asparagine amidohydrolase	
LOC100651637	-0.628690498	0.04001199	Down-regulated	protein phosphatase 1 regulatory subunit 42	
LOC100646261	0.232400424	0.040101054	Up-regulated	general transcription factor IIE subunit 2	
LOC105665858	-0.411844145	0.040371125	Down-regulated	receptor-type tyrosine-protein phosphatase S	
LOC100647962	0.373699921	0.040801782	Up-regulated	DDB1- and CUL4-associated factor 6	
LOC100643113	-0.297704222	0.040801782	Down-regulated	sarcosine dehydrogenase	
LOC100642217	-0.183064662	0.040810648	Down-regulated	pseudouridine-metabolizing bifunctional protein C1861.05	
LOC100650377	0.391672814	0.040991648	Up-regulated	40S ribosomal protein SA	
LOC100651074	0.314879772	0.040991648	Up-regulated	trifunctional enzyme subunit beta	
LOC100646246	1.181219358	0.041579482	Up-regulated	arylsulfatase B	
LOC100642757	0.343258511	0.041907458	Up-regulated	proteasome subunit beta type-1	
LOC100642656	0.951174281	0.042221411	Up-regulated	mannose-P-dolichol utilization defect 1 protein homolog	
LOC100648938	-0.397213461	0.042630771	Down-regulated	probable tubulin polyglutamylase ttll-15	
LOC100649127	0.220651713	0.042959421	Up-regulated	nascent polypeptide-associated complex subunit alpha	
LOC110119463	-0.255469744	0.042959421	Down-regulated	uncharacterized LOC110119463	
LOC110119460	1.139872172	0.043086663	Up-regulated	uncharacterized LOC110119460	
LOC100648212	0.970207272	0.043086663	Up-regulated	putative inorganic phosphate cotransporter	
LOC100650140	0.247654589	0.043233948	Up-regulated	26S proteasome regulatory subunit 10B	
LOC100644566	0.243996123	0.043233948	Up-regulated	translationally-controlled tumor protein homolog	
LOC100647412	0.705573125	0.043766611	Up-regulated	aquaporin AQPAn.G	
LOC100651688	0.181948746	0.044067824	Up-regulated	activating transcription factor of chaperone	
LOC100649994	-0.149362322	0.044067824	Down-regulated	pre-mRNA-processing factor 39	
LOC100651946	0.26048545	0.044176317	Up-regulated	ras-related protein Rab-39B	
LOC100649954	0.231266518	0.044188805	Up-regulated	coiled-coil domain-containing protein 124	
LOC100649504	0.948282603	0.044415202	Up-regulated	mitochondrial amidoxime-reducing component 1	
LOC100648002	1.516418425	0.044616554	Up-regulated	elongation of very long chain fatty acids protein 6	
LOC100631077	0.315246436	0.044616554	Up-regulated	ribosomal protein L13	
LOC100649326	-0.148584803	0.044616554	Down-regulated	zinc finger protein 354A	
LOC110119351	-0.285578648	0.044795657	Down-regulated	uncharacterized protein DDB_G0272718-like	
LOC100647941	0.58700951	0.044852854	Up-regulated	uncharacterized protein DDB_G0283697	
LOC100647473	-0.29482798	0.044852854	Down-regulated	U11/U12 small nuclear ribonucleoprotein 25 kDa protein	

LOC100651521	0.275871088	0.045059694	Up-regulated	ATP synthase subunit g
LOC100649704	-0.215333167	0.045519747	Down-regulated	calmodulin
LOC100644599	-0.274467852	0.045601869	Down-regulated	endothelial zinc finger protein induced by tumor necrosis factor alpha
LOC100644713	1.870514649	0.045867659	Up-regulated	laccase-1
LOC105666529	0.828982545	0.045867659	Up-regulated	aquaporin-11
LOC100645785	0.51735273	0.045867659	Up-regulated	protein obstructor-E
LOC100642832	0.373371159	0.045867659	Up-regulated	protein diaphanous
LOC100652287	0.25048817	0.045867659	Up-regulated	NADH dehydrogenase [ubiquinone] flavoprotein 1
LOC105666047	0.208579273	0.045867659	Up-regulated	eukaryotic translation initiation factor eIF1
LOC100648526	0.174111666	0.045867659	Up-regulated	protein-L-isoaspartate O-methyltransferase domain-containing protein 1
LOC100645651	0.167619574	0.045867659	Up-regulated	39S ribosomal protein L10
LOC100645157	-0.262924693	0.045867659	Down-regulated	UV radiation resistance-associated gene protein
LOC100644753	-0.43911331	0.045867659	Down-regulated	uncharacterized LOC100644753
LOC100645567	1.842550863	0.045984399	Up-regulated	UDP-glucosyltransferase 2
LOC100643225	-0.237141003	0.045984399	Down-regulated	WD repeat-containing protein 19
LOC100650951	2.159189137	0.046003907	Up-regulated	uncharacterized LOC100650951
LOC100642441	-0.19588087	0.046084606	Down-regulated	protein HID1
LOC100646787	1.021333537	0.046411462	Up-regulated	neuronal acetylcholine receptor subunit alpha-3
LOC100651386	0.398589634	0.046415786	Up-regulated	DDRGK domain-containing protein 1
LOC100651856	0.330600558	0.046415786	Up-regulated	glycerol-3-phosphate acyltransferase 3-like
LOC100650241	0.320028132	0.046415786	Up-regulated	uncharacterized LOC100650241
LOC100649532	0.197533106	0.046415786	Up-regulated	complement component 1 Q subcomponent-binding protein
LOC100644979	0.363720023	0.046474929	Up-regulated	uncharacterized LOC100644979
LOC100649080	0.303310623	0.046505566	Up-regulated	rab11 family-interacting protein 2
LOC105666469	0.291720716	0.046505566	Up-regulated	signal recognition particle subunit SRP68
LOC100648130	0.285568459	0.046785796	Up-regulated	protein SEC13 homolog
LOC100649110	2.717213844	0.046928705	Up-regulated	cytochrome P450 6k1 LOC100649110
LOC100645016	0.789219156	0.046928705	Up-regulated	elongation of very long chain fatty acids protein 6
LOC100647619	0.625031685	0.046928705	Up-regulated	putative uncharacterized protein DDB_G0277255
LOC100643432	-0.190789434	0.046928705	Down-regulated	1-phosphatidylinositol 3-phosphate 5-kinase
LOC100652314	-0.202476801	0.046928705	Down-regulated	transcriptional repressor CTCFL
LOC100642421	-0.237862773	0.046928705	Down-regulated	galactoside 2-alpha-L-fucosyltransferase Sec1
LOC100643158	0.501488454	0.046938948	Up-regulated	proline hydroxylase buaE
LOC100645439	-0.246871094	0.047033495	Down-regulated	uncharacterized LOC100645439
LOC100645908	-0.289437227	0.047033495	Down-regulated	elongation of very long chain fatty acids protein 4
LOC100644354	0.266619497	0.047068658	Up-regulated	uncharacterized LOC100644354
LOC100642294	0.28214799	0.048333436	Up-regulated	(Lyso)-N-acylphosphatidylethanolamine lipase
LOC100643956	-0.163946914	0.048396356	Down-regulated	tetraspanin-13
LOC100645719	-0.21213462	0.048396356	Down-regulated	uncharacterized LOC100645719
LOC100643297	0.528681916	0.048756281	Up-regulated	NAD kinase
LOC100647488	0.252105793	0.048756281	Up-regulated	death-associated inhibitor of apoptosis 1
LOC100647223	0.959999709	0.04900403	Up-regulated	protein yellow
LOC100643282	-0.196454717	0.049131223	Down-regulated	acetylcholine receptor subunit beta-like 2
LOC100647184	0.244169843	0.04914462	Up-regulated	6-phosphogluconolactonase
LOC100642963	1.374409473	0.04924207	Up-regulated	uncharacterized LOC100642963
LOC100645332	0.202437615	0.04924207	Up-regulated	Golgi resident protein GCP60
LOC100644383	0.330804722	0.04928668	Up-regulated	interferon-related developmental regulator 2
LOC100647326	0.200254596	0.04928668	Up-regulated	coatomer subunit alpha-like
LOC100647777	-0.135945316	0.04933264	Down-regulated	sporulation-specific protein 15
LOC100651721	-0.379642839	0.04933264	Down-regulated	ionotropic receptor 21a
LOC105666197	0.21460927	0.049907189	Up-regulated	uncharacterized LOC105666197

ACUTE SULFOXAFLOR

Gene ID	log2Fold Change	Adjusted P-value (FDR)	Expression pattern	Gene annotation
LOC100648995	10.51238799	7.71E-11	Up-regulated	cytochrome P450 6k1 LOC100648995
LOC100648003	6.50184742	8.33E-11	Up-regulated	UDP-glycosyltransferase UGT5
LOC100652071	10.1455956	2.49E-09	Up-regulated	venom carboxylesterase-6
LOC105666366	6.430792596	3.26E-08	Up-regulated	cytochrome P450 6k1 LOC105666366
LOC100642816	6.927380739	4.60E-06	Up-regulated	cytochrome P450 6k1 LOC100642816
LOC100650427	7.815094978	1.02E-04	Up-regulated	cytochrome P450 6k1 LOC100650427
LOC100642937	2.184367707	0.003429992	Up-regulated	actin-binding Rho-activating protein
LOC100647646	2.527455638	0.010127882	Up-regulated	UDP-glycosyltransferase UGT5-like
LOC100643891	1.143388083	0.010127882	Up-regulated	zinc carboxypeptidase
LOC100643342	1.820779177	0.010127882	Up-regulated	zinc finger protein rotund
LOC100645713	1.174014237	0.010379718	Up-regulated	uncharacterized LOC100645713
LOC100649033	1.987766127	0.010919826	Up-regulated	cyclin-dependent kinase-like 4
LOC100646259	0.741903641	0.013678397	Up-regulated	trichohyalin
LOC100647860	1.859838858	0.01696646	Up-regulated	uncharacterized LOC100647860
LOC100646382	1.814743271	0.017553251	Up-regulated	ras-related and estrogen-regulated growth inhibitor
LOC100648162	0.985117726	0.021699741	Up-regulated	uncharacterized LOC100648162
LOC100649312	0.30770225	0.021699741	Up-regulated	max-like protein X
LOC100645338	2.484216826	0.022000395	Up-regulated	ATP-binding cassette sub-family C member 4
LOC105666141	1.819350401	0.022000395	Up-regulated	15-hydroxyprostaglandin dehydrogenase [NAD(+)]
LOC100642983	0.960992304	0.022000395	Up-regulated	protein bicaudal C homolog 1-B
LOC100650927	1.585420986	0.025563724	Up-regulated	uncharacterized LOC100650927
LOC100652147	1.788855169	0.02673915	Up-regulated	peroxisomal hydratase-dehydrogenase-epimerase
LOC100645515	-0.232077455	0.02673915	Down-regulated	kinesin-like protein KIF3A
LOC100650251	0.687092758	0.027728979	Up-regulated	plexin domain-containing protein 2
LOC100649110	3.655867664	0.027931258	Up-regulated	cytochrome P450 6k1 LOC100649110
LOC100643462	2.117791028	0.027931258	Up-regulated	RING finger protein nhl-1
LOC100645530	2.12872167	0.028702002	Up-regulated	E3 ubiquitin-protein ligase lubel
LOC100649782	-0.205644485	0.030836874	Down-regulated	pleckstrin homology domain-containing family M member 2

LOC100646145	1.775217346	0.032317519	Up-regulated	ejaculatory bulb-specific protein 3
LOC100651337	1.551796493	0.032317519	Up-regulated	acyl-CoA-binding protein homolog
LOC100644592	2.305399574	0.032532435	Up-regulated	uncharacterized LOC100644592
LOC100645567	2.395152043	0.032740026	Up-regulated	UDP-glucosyltransferase 2
LOC100646012	1.889166674	0.032740026	Up-regulated	isopentenyl-diphosphate Delta-isomerase 1-like
LOC100650354	-2.118186014	0.032740026	Down-regulated	tropomyosin Per a 7.0102
LOC110119239	-19.17552017	0.032740026	Down-regulated	uncharacterized LOC110119239
LOC100649151	1.665899877	0.032985151	Up-regulated	zinc finger CCHC domain-containing protein 24
LOC100646667	1.423981825	0.034636388	Up-regulated	circadian clock-controlled protein daywake
LOC100649799	1.044899674	0.034636388	Up-regulated	transketolase
LOC100648124	0.813000467	0.034636388	Up-regulated	glucose-6-phosphate 1-dehydrogenase
LOC100651074	0.386109791	0.034636388	Up-regulated	trifunctional enzyme subunit beta
LOC100645203	2.281061717	0.034636388	Up-regulated	uncharacterized LOC100645203
LOC100644880	2.052446941	0.034636388	Up-regulated	uncharacterized LOC100644880
LOC100643066	2.046681457	0.034636388	Up-regulated	bestrophin-4
LOC100651289	2.045600163	0.034636388	Up-regulated	uncharacterized LOC100651289
LOC100643536	2.028529103	0.034636388	Up-regulated	protein javelin
LOC100651217	1.674246984	0.034636388	Up-regulated	sarcoplasmic calcium-binding protein 1
LOC100651376	1.668713651	0.034636388	Up-regulated	alpha-tocopherol transfer protein-like
LOC100649627	1.645595822	0.034636388	Up-regulated	4-nitrophenylphosphatase
LOC100648473	1.59192495	0.034636388	Up-regulated	2-acylglycerol O-acyltransferase 1
LOC100646983	1.273723878	0.034636388	Up-regulated	uncharacterized LOC100646983
LOC100645959	1.268644669	0.034636388	Up-regulated	uncharacterized LOC100645959
LOC100645452	1.18758282	0.034636388	Up-regulated	apolipoprotein D
LOC100648853	0.948544395	0.034636388	Up-regulated	phospholipase B1
LOC100642272	0.851228069	0.034636388	Up-regulated	very-long-chain 3-oxoacyl-CoA reductase
LOC100644626	0.614432861	0.034636388	Up-regulated	40S ribosomal protein S28
LOC100645181	0.505508199	0.034636388	Up-regulated	segmentation protein cap'n'collar
LOC100649561	0.466744502	0.034636388	Up-regulated	laminin subunit alpha-1
LOC100645201	0.287972616	0.034636388	Up-regulated	RING finger protein 10
LOC100651041	-0.200615087	0.034636388	Down-regulated	protein Lilipod
LOC100651084	-0.249176198	0.034636388	Down-regulated	gamma-1-syntrophin
LOC100644231	-2.539353546	0.034636388	Down-regulated	tropomyosin-1
LOC100643103	1.385767368	0.035031842	Up-regulated	homeotic protein proboscipedia
LOC100651531	1.352680155	0.035031842	Up-regulated	uncharacterized LOC100651531
LOC100649621	1.176517428	0.036525376	Up-regulated	insulin-like growth factor-binding protein complex acid labile subunit
LOC100644410	0.387176414	0.037080518	Up-regulated	protein transport protein Sec61 subunit gamma
LOC100648814	1.065055925	0.037540002	Up-regulated	uncharacterized LOC100648814
LOC100646721	0.546153072	0.037546679	Up-regulated	venom acid phosphatase Acph-1
LOC100646544	1.576985672	0.037546679	Up-regulated	uncharacterized LOC100646544
LOC100643293	0.852112594	0.037546679	Up-regulated	probable inactive protein kinase DDB_G0270444
LOC100646270	0.782139459	0.037546679	Up-regulated	maternal protein exuperantia
LOC105666047	0.255860753	0.037546679	Up-regulated	eukaryotic translation initiation factor eIF1
LOC100651918	0.263102953	0.038389917	Up-regulated	26S proteasome regulatory subunit 4
LOC100646227	2.2610076	0.039767378	Up-regulated	peroxidase
LOC100645304	1.662291514	0.039767378	Up-regulated	EF-hand domain-containing family member C2
LOC105666016	1.197559555	0.039767378	Up-regulated	uncharacterized LOC105666016
LOC100650811	0.447006252	0.039767378	Up-regulated	thiamin pyrophosphokinase 1
LOC100650077	1.930824839	0.040098296	Up-regulated	translation initiation factor IF-2
LOC100645631	1.421228388	0.041070796	Up-regulated	sodium-independent sulfate anion transporter
LOC100645595	1.188173793	0.041070796	Up-regulated	retinoid-inducible serine carboxypeptidase
LOC100648641	0.427542297	0.041070796	Up-regulated	peroxisomal multifunctional enzyme type 2
LOC110120360	1.047294745	0.041070796	Up-regulated	uncharacterized LOC110120360
LOC105666292	0.326075569	0.041070796	Up-regulated	dihydrolipoyllysine-residue acetyltransferase component -pyruvate dehydrogenase
LOC100645851	0.150327902	0.041152209	Up-regulated	phospholipase A-2-activating protein
LOC100643512	1.912831973	0.041341226	Up-regulated	lysosomal acid glucosylceramidase
LOC100642748	1.793756897	0.041341226	Up-regulated	diacylglycerol lipase-beta
LOC100650582	1.674147312	0.041341226	Up-regulated	uncharacterized LOC100650582
LOC100651254	1.587227534	0.041341226	Up-regulated	clavesin-1
LOC100649554	1.465498329	0.041341226	Up-regulated	histone-lysine N-methyltransferase SETMAR
LOC100646892	1.296865632	0.041341226	Up-regulated	homeobox protein aristaless
LOC100646822	1.276061437	0.041341226	Up-regulated	clavesin-2
LOC100651517	1.259876362	0.041341226	Up-regulated	RNA-binding protein fusilli
LOC100645016	0.952310265	0.041341226	Up-regulated	elongation of very long chain fatty acids protein 6
LOC100647239	0.813010915	0.041341226	Up-regulated	uncharacterized LOC100647239
LOC100648262	-0.665844154	0.041341226	Down-regulated	uncharacterized LOC100648262
LOC100649005	1.300641734	0.041950305	Up-regulated	homeobox protein ceh-17
LOC100647077	-0.138715613	0.042436808	Down-regulated	protein shuttle craft
npve	1.131014845	0.042436808	Up-regulated	acyl-CoA delta-9 desaturase
LOC100648516	1.10868669	0.042436808	Up-regulated	sushi
LOC100644819	0.476927932	0.042814984	Up-regulated	fumarylacetoacetase
LOC100652045	-0.190973329	0.042814984	Down-regulated	alsin
LOC100649919	1.862942016	0.042879401	Up-regulated	nose resistant to fluoxetine protein 6
LOC100648579	1.715606757	0.045527075	Up-regulated	uncharacterized LOC100648579
LOC100648426	1.246716674	0.045527075	Up-regulated	acetyl-CoA acetyltransferase
LOC100651772	0.321970171	0.045527075	Up-regulated	ras-related protein RabJ
LOC100644599	-0.319863529	0.047078272	Down-regulated	endothelial zinc finger protein induced by tumor necrosis factor alpha
LOC100645262	0.759657807	0.048665032	Up-regulated	uncharacterized LOC100645262
LOC100650572	1.649917384	0.0492824	Up-regulated	scavenger receptor class B member 1
LOC100651996	0.661260969	0.0492824	Up-regulated	transaldolase
LOC110119633	1.168652859	0.0492824	Up-regulated	myb-like protein X

LOC100647457	0.791593118	0.0492824	Up-regulated	leupaxin
LOC100646207	0.999963265	0.049361882	Up-regulated	serine protease gd
LOC100642963	1.614922914	0.049361882	Up-regulated	uncharacterized LOC100642963
LOC100645047	1.192443767	0.049361882	Up-regulated	alpha-(1
LOC100643073	2.046651045	0.049404411	Up-regulated	uncharacterized LOC100643073
